# A Theoretical Model of the Wnt Signaling Pathway in the Epithelial Mesenchymal Transition

**DOI:** 10.1186/s12976-017-0064-7

**Published:** 2017-10-10

**Authors:** Kelsey Gasior, Marlene Hauck, Alyson Wilson, Sudin Bhattacharya

**Affiliations:** 1 North Carolina State University Biomathematics Program, Cox Hall, 2700 Stinson Dr, Raleigh, NC 27607 USA; 2 North Carolina State University College of Veterinary Medicine, 1060 William Moore Dr, Raleigh, NC 27607 USA; 3 North Carolina State University Department of Statistics, SAS Hall, 2311 Stinson Dr, Raleigh, NC 27607 USA; 4 Department of Biomedical Engineering, Michigan State University, 775 Woodlot Drive, East Lansing, MI 48824-1226 USA; 5 Pharmacology & Toxicology, Michigan State University, 1355 Bogue Street, B305 Life Sciences Building, East Lansing, MI 48824 USA; 6 Center for Research on Ingredient Safety, Michigan State University, 1129 Farm Lane, East Lansing, MI 48824 USA; 7 Institute for Quantitative Health Science and Engineering, Michigan State University, 775 Woodlot Drive, East Lansing, MI 48824 USA; 8 Present address: University of North Carolina at Chapel Hill Department of Biology, Coker Hall CB #3280, 120 South Rd, Chapel Hill, NC 27599 USA

**Keywords:** Epithelial Mesenchymal Transition (EMT), Wnt, Bistable

## Abstract

**Background:**

Following the formation of a primary carcinoma, neoplastic cells metastasize by undergoing the epithelial mesenchymal transition (EMT), which is triggered by cues from inflammatory and stromal cells in the microenvironment. EMT allows epithelial cells to lose their highly adhesive nature and instead adopt the spindle-like appearance, as well as the invasive and migratory behavior, of mesenchymal cells. We hypothesize that a bistable switch between the epithelial and mesenchymal phenotypes governs EMT, allowing the cell to maintain its mesenchymal phenotype even after it leaves the primary tumor microenvironment and EMT-inducing extracellular signal.

**Results:**

This work presents a simple mathematical model of EMT, specifically the roles played by four key proteins in the Wnt signaling pathway: Dishevelled (Dvl), E-cadherin, β-catenin, and Slug. The model predicts that following activation of the Wnt pathway, an epithelial cell in the primary carcinoma must attain a threshold level of membrane-bound Dvl to convert to the mesenchymal-like phenotype and maintain that phenotype once it has migrated away from the primary tumor. Furthermore, sensitivity analysis of the model suggests that in both the epithelial and the mesenchymal states, the steady state behavior of E-cadherin and the transcription factor Slug are sensitive to changes in the degradation rate of Slug, while E-cadherin is also sensitive to the IC_50_ (half-maximal) concentration of Slug necessary to inhibit E-cadherin production. The steady state behavior of Slug exhibits sensitivity to changes in the rate at which it is induced by β-catenin upon activation of the Wnt pathway. In the presence of sufficient amount of Wnt ligand, E-cadherin levels are sensitive to the ratio of the rate of Slug activation via β-catenin to the IC_50_ concentration of Slug necessary to inhibit E-cadherin production.

**Conclusions:**

The sensitivity of E-cadherin to the degradation rate of Slug, as well as the IC_50_ concentration of Slug necessary to inhibit E-cadherin production, shows how the adhesive nature of the cell depends on finely-tuned regulation of Slug. By highlighting the role of β-catenin in the activation of EMT and the relationship between E-cadherin and Slug, this model identifies critical parameters of therapeutic concern, such as the threshold level of Dvl necessary to inactivate the GSK-3β complex mediating β-catenin degradation, the rate at which β-catenin translocates to the nucleus, and the IC_50_ concentration of Slug needed to inhibit E-cadherin production.

**Electronic supplementary material:**

The online version of this article (10.1186/s12976-017-0064-7) contains supplementary material, which is available to authorized users.

## Background

Epithelial tumors, or carcinomas, are the most common type of neoplasia found in humans, accounting for roughly 80% of all cancer related deaths in the Western world [1]. Characteristically, epithelial cells form polarized sheets. Within these sheets, cells are tightly adhered to each other to inhibit individual cellular movement [2]. This highly adhesive nature is attributable to molecules of E-cadherin, a transmembrane protein, on the surface of one epithelial cell binding with the E-cadherin moelcules of another epithelial cell across the extracellular space. Simultaneously, E-cadherin is bound to intracellular catenin members on its cytoplasmic tail, which stabilize the E-cadherin by linking it to the actin cytoskeleton [3, 4]. This E-cadherin-catenin complex is what gives the epithelial cells in both normal tissue and carcinomas their strong adhesive bonds and inhibits the movement of individual cells [2, 4].

In carcinomas, it is possible for neoplastic cells to undergo epithelial mesenchymal transition (EMT) [3]. Triggered by cues from the local stroma [1], EMT influences the expression of the cellular adhesion complex [5] and allows epithelial cells to acquire the spindle-like appearance of mesenchymal cells, as well as their invasive and migratory properties [5, 6]. While this process plays a key role in embryogenesis [7] and wound healing [5], the occurrence of EMT in tumor cells is thought to help drive the onset of metastasis, although the level to which EMT is involved in this process is currently debated due to the heterogeneous nature of cellular responses that can occur in the same tumor [5]. However, for some cells within a tumor, with the loss of adhesion and the acquisition of an invasive phenotype, they are able to invade the extracellular matrix around the tumor, penetrate the basement membrane of the blood vessel, and travel via the blood stream to other locations in the body where they ultimately form a metastasis [8].

There are a multitude of different pathways that can activate EMT, with significant crosstalk between these signaling systems. In mature multicellular organisms, as well as during development, the Wnt pathway helps regulate proliferation, differentiation, migration, and polarity [9, 10]. However, mutations and dysregulation of the pathway can ultimately lead to the occurrence of tumors and the activation of EMT leading to metastasis [9, 11].

The Wnt pathway has long been the focus of studies attempting to understand cellular behavior. Lee et al. [12] proposed a 15-equation mathematical model centered upon the formation of the protein complexes involved in β-catenin phosphorylation and degradation, a process that, when disrupted, leads to the accumulation of the β-catenin/ TCF complex in the nucleus. In particular, the authors attempted to explain the distinct roles that different members of the degradation complex can carry out. In addition, these authors carried out experiments in *Xenopus* egg extracts to support their hypothesized interactions [12]. Ramis-Conde et al. [13] built on the work of Lee and others with a multiscale mathematical model that examined the involvement of E-cadherin and β-catenin in the adhesion of epithelial cells to one another. Like Lee et al., Ramis-Conde et al. included the involvement of β-catenin in the degradation complex in the cytosol. However, Ramis-Conde et al. also focused on the behavior of E-cadherin. The authors considered the concentration of E-cadherin to be a constant that is then divided by sub-cellular localization: free E-cadherin at the membrane, free E-cadherin in the cytosol, and the E-cadherin-β-catenin membrane complex [13].

Basan et al. [14] also explored the adhesion complex relationship. However, where Ramis-Conde et al. considered the key component of cellular adhesion to be the binding of β-catenin and E-cadherin, which they modeled using ordinary differential equations (ODEs), Basan et al. examined the relationship between β-catenin and E-cadherin and the involvement of α-catenin in the adhesion complex by using reaction-diffusion equations [14]. The dynamics that exist between the adhesion complex and the Wnt pathway were explored further by Chen et al. [11]. In particular, the authors sought to understand the role spatial dynamics play in intracellular signaling. By employing a network model, Chen et al. found that the membrane adhesion complex not only competes with the degradation complex for free β-catenin, but also that the clustering of adhesion complexes can slow down the degradation complex and thus allow for an increase in nuclear signaling.

Other authors have continued to explore the influence noncanonical proteins can have on the dynamics of Wnt signaling, as well as its crosstalk with other pathways. One protein of interest has been Dickkopf1 (Dkk), which inhibits the Wnt pathway and cell proliferation, as well as promotes maturation in cells. By creating a multi-scale model to describe the relationship between the Wnt and Notch pathways, Agur et al. suggested that high levels of Dkk can push stem cells towards differentiation [15]. Kogan et al. also used mathematical modeling to examine the influence of Dkk on the Wnt pathway, but these authors examined its influence in combination with a secreted Frizzled-related protein (sFRP), another known Wnt inhibitor [9]. By proposing an ODE model with 13 variables and employing mass action kinetics, Kogan et al. suggested that the combination of sFRP1 and Dkk1 can work together to inhibit the accumulation of β-catenin in the cell, a direct outcome of the canonical Wnt signaling pathway.

Kim et al. highlighted the positive feedback loop that exists between the Wnt and ERK signaling pathways [16]. By extending the original model proposed by Lee et al. [12] and incorporating the influence of the ERK pathway, Kim et al. suggest that the crosstalk and positive feedback loop that exists between Wnt and ERK can lead to a bistable switch in cellular behavior. Shin et al. [17] found similar results to Kim et al. during their exploration of Wnt and ERK pathway crosstalk. Based on a system of six ODEs, the model proposed by the authors included the involvement of the adhesion complex, the complex responsible for β-catenin degradation, as well as the shuttling of β-catenin to the nucleus and its involvement with the transcription factor Slug. They also examined the changes in E-cadherin in response to different oncogenic stimuli: EGF and Wnt, and proposed a switch-like behavior in E-cadherin, allowing the cell to transition from the epithelial to the mesenchymal state [17]. The existence of this bistable switch was also discussed by Maclean et al. [18] who put forth a 19-equation model of the bistable switch between the epithelial and mesenchymal steady states due to cytoplasmic-nuclear shuttling of β-catenin.

It is worthwhile to note that the concept of a bistable switch is not unique to systems involving the activation of or the overlap with the Wnt signaling pathway. Recent work has examined the roles that the miR-200/ZEB, the LIN28/let-7, and the Ovol2-Zeb1 circuits may play in the activation of EMT, as well as possible overlap that may exist [19–21]. However, rather than an all or nothing switch, each of these proposed models accounts for at least one intermediate state between the epithelial and mesenchymal transitions, a state that Jolly et al. suggest is responsible for stemness and has the highest potential for tumor initiation [19, 20].

Due to the complex nature of the Wnt signaling pathway and intracellular signals as a whole, models are often quite intricate, featuring large sets of equations, and most only focus on a few of the molecular interactions β-catenin is involved in. Even models that sought to use minimal equations and variables to describe the Wnt signaling pathway, such as the work done by Benary et al., still resulted in the use of 5–6 differential equations and two conservation equations [22]. In this work, we, like Kim et al., Shin et al., and MacLean et al., hypothesize that the mechanism underlying EMT in a primary solid tumor is a bistable switch between the epithelial and the mesenchymal phenotypes: once transitioned, the cell will maintain its mesenchymal phenotype, even in the absence of sustained extracellular signaling. With regards to the Wnt pathway, we hypothesize that the bistable switch is centered around the behavior and interactions of β-catenin. Unlike many of the previous works mentioned, the model put forth here incorporates all three primary interactions β-catenin is involved in: the adhesion complex with E-cadherin, its degradation via the complex formed by GSK-3β, and its translocation to the nucleus to activate the transcription factor Slug. Additionally, even though we are examining all three relationships involved in the switch-like behavior of the cell, we propose a simplified theoretical model of three ODEs to describe the ultrasensitive feedback loop in the Wnt-β-catenin signaling pathway. By creating a simple model of the primary interactions of β-catenin in the Wnt pathway, we are able to show how activation of the Wnt pathway alone can drive EMT in a carcinoma cell, as well as explain the intracellular β-catenin interactions that may be causing the bistable switch. Our model could provide a scaffold upon which other, more complex models of EMT and the multistable switch could be built, as well as guide experimental exploration into which interactions could be targeted for prevention of EMT and metastasis.

## Methods

In a Wnt-absent environment, E-cadherin binds with intracellular catenin members, such as α, β, and γ- catenin [4]. Any free β-catenin that is not bound in this complex is degraded in the cytosol by a protein complex [23]. Axin, a scaffold protein that is present in epithelial cells [24], mediates the formation of this cytosolic degradation complex along with the proteins adenomatous polyposis coli (APC), glycogen synthase kinase 3β (GSK-3β), casein kinase 1 (CK1), and protein phosphatase 2A (PP2A) [25]. When β-catenin comes in contact with Axin and APC, the casein kinase 1α (CK1α) component of Axin and GSK-3β work together to phosphorylate β-catenin and mark it for degradation [23]. However, there currently exists some controversy around the degradation of β-catenin, as the process by which it occurs is poorly understood. Experimental work suggests that it may not be necessary for the complex to release β-catenin for degradation to occur. Rather, it may be possible for the degradation complex to undergo a structural change that exposes the β-catenin and allows it to be degraded [11]. Regardless of the exact mechanisms of the process, it is through this constant degradation that the cytosolic β-catenin is maintained at a low concentration in the absence of Wnt [23].

Carcinomas are associated with an oncogenic promoting stroma that is considered ‘reactive’, and is characterized, in part, by an increase in the number of fibroblasts that now associate with the ECM [26, 27]. These fibroblasts secrete the Wnt protein, a morphogen that relies on short-range signaling in order to activate the Wnt pathway in carcinoma cells [1, 28]. Once the Wnt ligand binds to its receptor, Frizzled (Frz) [12], on epithelial cells, LRP5-LRP6 receptors are phosphorylated by CK1γ and GSK-3β [23]. The messenger protein Disheveled (Dvl) is brought to the plasma membrane where it associates with Frz. Dvl then binds with Axin at the membrane to deactivate and dismantle the Axin/APC/GSK-3β complex, allowing β-catenin to accumulate in the cytosol and subsequently translocate to the nucleus [25]. In the absence of Wnt signaling, the T-cell factor and lymphoid enhancer factor (TCF/LEF) works in the nucleus with Groucho to repress Wnt target genes [29]. However, once Wnt signaling is activated, β-catenin replaces the Groucho factors and is able to form a complex with TCF and LEF1 [23], upregulating the expression of transcription factors such as Slug [17]. Slug, a zinc-finger transcription factor, then binds to E-boxes in the promoter regions of the E-cadherin gene and prevents transcription of E-cadherin [30]. This suppression of E-cadherin production limits the concentration of E-cadherin available to bind with β-catenin, further encouraging the translocation of β-catenin to the nucleus as it accumulates in the cytosol [31], thus creating a Wnt-driven feedback loop. The continued suppression of E-cadherin production by Slug and other transcription factors ultimately causes the epithelial cell to lose its adhesion with the other surrounding epithelial cells and undergo EMT.

**Fig. 1:**
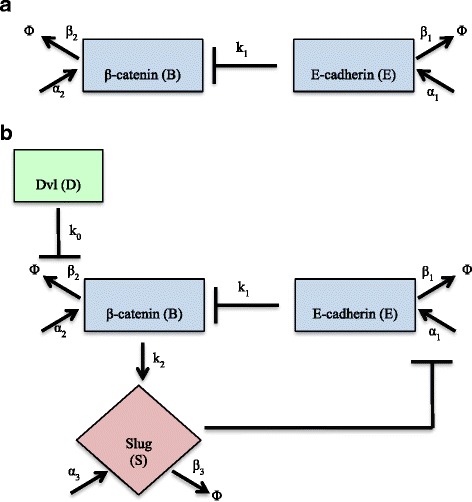
**a** β-catenin – E-cadherin relationship in a primary carcinoma tumor cell, pre-EMT. E-cadherin sequesters cytosolic β-catenin at the cell membrane where it forms a complex with other members of the catenin family to help E-cadherin attach to the cell’s cytoskeleton. **b** The upregulation of Dvl via Wnt signaling inhibits the degradation of β-catenin by deactivating the GSK-3β/Axin complex, allowing β-catenin to translocate to the nucleus and upregulate the transcription factor Slug. Slug suppresses the transcription of E-cadherin, which means there is less E-cadherin to sequester β-catenin at the membrane. β-catenin can continue to accumulate and translocate to the nucleus, thus completing the feedback the loop

Within these complex dynamics, we identify 3 key relationships centered around β-catenin, which are shown in Fig. 1. In a primary carcinoma cell, β-catenin is sequestered into a complex with E-cadherin at the membrane. Any β-catenin that is not in this complex is marked for degradation. With the activation of the Wnt pathway, the degradation complex is inactivated due to the movement of Dvl to the cellular membrane, allowing β-catenin to accumulate, translocate to the nucleus, and upregulate Slug. In order to model these β-catenin relationships at the core of the canonical Wnt pathway and the manner in which they affect the key proteins involved, we employed a system of three ODEs. The changes over time in the concentration of membrane-bound E-cadherin (E), free cytosolic β-catenin (B), and Slug (S) are described in eqs. (1)–(3) below. A complete list of the parameters and their definitions is provided in Table 1.

**Table 1:** Parameter Definitions for ODE Model

Parameter	Definition	Units	Assumed Value	Source
α_1_	Basal production of E-cadherin	$\frac{nM}{\mathit{\min}}$	0.01	estimated
α_2_	Basal production of β-catenin	$\frac{nM}{\mathit{\min}}$	0.01	[13]
α_3_	Basal production of Slug	$\frac{nM}{\mathit{\min}}$	0.001	estimated
β_1_	Basal degradation of E-cadherin	$\frac{1}{\mathit{\min}}$	0.03	estimated
β_2_	Rate at which β-catenin binds to the GSK-3β / Axin/APC complex	$\frac{1}{\mathit{\min}}$	0.03	estimated
β_3_	Basal degradation of Slug	$\frac{1}{\mathit{\min}}$	0.03	estimated
*k*_0_	Rate at which Dvl inactivates the GSK-3β/Axin/ APC complex	$\frac{nM}{\mathit{\min}}$	3.7 ∙ 10^−3^	estimated
*k*_1_	Rate at which E-cadherin sequesters β-catenin at the cell membrane	$\frac{nM}{\mathit{\min}}$	0.01	estimated
*k*_2_	Rate at which β-catenin upregulates Slug in the cell nucleus	$\frac{nM}{\mathit{\min}}$	1	estimated
*IC*_*S*_	Half maximal concentration of Slug required to inhibit E-cadherin transcription	*nM*	3.3	estimated
*IC*_*B*_	Half maximal concentration of β-catenin needed up upregulate Slug	*nM*	0.33	estimated
*IC*_*E*_	Half maximal concentration of E-cadherin needed for sequestration of β-catenin at membrane	*nM*	0.033	estimated
*IC*_*D*_	Half maximal concentration of Dvl needed to inhibit the degradation of β-catenin by GSK-3β	*nM*	0.67	estimated
*n*_1_	Hill Coefficient	–	3	estimated
*n*_2_	Hill Coefficient	–	2	estimated
*n*_3_	Hill Coefficient	–	2	estimated
*n*_4_	Hill Coefficient	–	5	estimated

1 $$ \frac{dE}{dt}=\frac{\alpha_1}{1+{\left(\frac{S}{IC_S}\right)}^{n_1}}-{\beta}_1\bullet E $$

2 $$ \frac{dB}{dt}={\alpha}_2-\frac{k_1\bullet {\left(\frac{E}{IC_E}\right)}^{n_2}}{1+{\left(\frac{E}{IC_E}\right)}^{n_2}}-\left({\beta}_2\bullet B-\frac{k_0\bullet {\left(\frac{D}{IC_D}\right)}^{n_4}}{1+{\left(\frac{D}{IC_D}\right)}^{n_4}}\right) $$

3 $$ \frac{dS}{dt}={\alpha}_3+\frac{k_2\bullet {\left(\frac{B}{IC_B}\right)}^{n_3}}{1+{\left(\frac{B}{IC_B}\right)}^{n_3}}-{\beta}_3\bullet S $$

In the epithelial steady state, the concentration of cytosolic β-catenin is very low: most β-catenin exists in complex form with other members of the catenin family and E-cadherin at the membrane. This relationship is represented in (2) with the term $\frac{k_1\bullet {\left(\frac{E}{IC_E}\right)}^{n_2}}{1+{\left(\frac{E}{IC_E}\right)}^{n_2}}$, where *k*_1_ is the rate at which membrane-bound E-cadherin sequesters β-catenin. In the model presented by Ramis-Conde et al. [13], the authors discuss two rates to describe this interaction: the rates at which β-catenin and E-cadherin bind and unbind to form this complex. However, the authors also estimate that the rate at which the two proteins bind to form the complex is 50× faster than the rate at which they unbind; hence we have used a single term to model the reduction in free cytosolic β-catenin due to the presence of E-cadherin.

Any β-catenin that is not bound to E-cadherin is subjected to phosphorylation by the GSK-3β complex and thus marked for degradation. In the model put forth by Kogan et al. [9], the authors condense the complex process of phosphorylation degradation by assuming that once β-catenin is phosphorylated, it is quickly degraded, a process they represent with a single term. Coupled with this term is another term to represent the disassociation of β-catenin from the degradation complex prior to phosphorylation. However, in the model created by Kogan et al., the rate at which β-catenin binds with the degradation complex is 10^8^x faster than the rate at which it escapes. Therefore, for simplicity, in this work, we assume that once β-catenin is bound to the GSK-3β complex in the epithelial steady state, it will be quickly phosphorylated and marked for degradation before it has the chance to unbind and exist freely in the cytosol. Thus, the basal degradation of β-catenin is represented by the term *β*_2_ ∙ *B* in (2), where *β*_2_ is the rate at which β-catenin binds to the GSK-3β complex. Once Wnt signaling is activated, Dvl works to break apart the GSK-3β complex by binding with Axin at the membrane, sparing β-catenin from phosphorylation and degradation. With Dvl (*D)* > 0, the term $\frac{k_0\bullet {\left(\frac{D}{IC_D}\right)}^{n_4}}{1+{\left(\frac{D}{IC_D}\right)}^{n_4}}>0$ in (2) where *k*_0_ is the rate at which Dvl binds to Axin and deactivates the degradation complex.

In this model, it is assumed that the cell has the ability to shuttle free β-catenin to the nucleus, as is represented by the term $\frac{k_2\bullet {\left(\frac{B}{IC_B}\right)}^{n_3}}{1+{\left(\frac{B}{IC_B}\right)}^{n_3}}$ in (3), where *k*_*2*_ is the rate at which β-catenin translocates to the nucleus and activates Slug. If free β-catenin in the cytosol is kept at a low value by the degradation complex, there is very little β-catenin available for transport. Once Wnt signaling is turned on and Dvl is upregulated, β-catenin can accumulate and translocate to the nucleus, enhancing the expression of Slug in (3). Slug then binds to E-box elements in the promoter of the E-cadherin gene, which inhibits E-cadherin production, as modeled in (1) with the term $\frac{\alpha_1}{1+{\left(\frac{S}{IC_S}\right)}^{n_1}}$ . These interactions constitute the core of the double-negative feedback loop driven by changes in the behavior of β-catenin. This double-negative feedback loop, combined with ultra-sensitivity in the interactions (represented by Hill coefficients *n*_1_ − *n*_4_ in (1)–(3)), creates the potential for a bistable switch.

Due to the number of parameters we were required to estimate for this model, as well as to continue our theoretical exploration into how these rates contribute to the underlying bistable switch, we nondimensionalized the system in (1)–(3) using the relationships

$$ {\displaystyle \begin{array}{l}E=\varepsilon \cdot e,\kern0.5em B=\lambda \cdot b,\kern0.5em S=\sigma \cdot s,\kern0.5em D=\delta \cdot d,\\ {}t=T\cdot \tau \end{array}} $$

where *e, b, s, d,* and *τ* are dimensionless variables. Substituting these relationships into (1)–(3) the constants *ε*, *λ*, *σ*, and *δ* were defined as *ε* = *IC*_*E*_, *λ* = *IC*_*B*_, *σ* = *IC*_*S*_, and *δ* = *IC*_*D*_. For the dimensional constant *T*, there were many parameter combinations that would result in the appropriate units of *minutes*. Ultimately, *T* was chosen to be the ratio of half the maximal concentration of β-catenin necessary to activate Slug to the rate at which E-cadherin sequesters β-catenin at the membrane, or $T=\frac{IC_B}{k_1}$. This ratio was chosen to reflect the time the cell would need to build up half the amount of β-catenin necessary to activate Slug, and begin the transition from the epithelial to the mesenchymal phenotype, while the pool of free β-catenin is depleted due to sequestration by E-cadherin. By using these substitutions, nondimensionalization allows for simplification and parameter grouping for further analysis. Our new nondimensional system is:

4 $$ \frac{d e}{d\tau}=\frac{A_1}{1+{s}^{n_1}}-{C}_1\bullet e $$

5 $$ \frac{d b}{d\tau}={A}_2-\frac{e^{n_2}}{1+{e}^{n_2}}-\left({C}_2\bullet b-\frac{F_1\bullet {d}^{n_4}}{1+{d}^{n_4}}\right) $$

6 $$ \frac{d s}{d\tau}={A}_3+\frac{F_2\bullet {b}^{n_3}}{1+{b}^{n_3}}-{C}_3\bullet s $$

While a range of values for each dimensionless parameter was explored (see Fig. 5), the values that were ultimately used in this model are defined in Table 2. Analysis was carried out using MATLAB software and XPPAUT [32] using the initial conditions of *e* = 10, *b* = 0, and *s* = 0 when the cell begins in the epithelial steady state.

**Table 2:** Parameter Definitions for Nondimensional ODE Model

Parameter	Definition	Value
*A*_1_	$\frac{\alpha_1\cdotp {IC}_B}{k_1\cdotp {IC}_E}$	10
*A*_2_	$\frac{\alpha_2}{k_1}$	1
*A*_3_	$\frac{\alpha_3\cdotp {IC}_B}{k_1\cdotp {IC}_S}$	0.01
*C*_1_	$\frac{\beta_1\cdotp {IC}_B}{k_1}$	1
*C*_2_	$\frac{\beta_2\cdotp {IC}_B}{k_1}$	1
*C*_3_	$\frac{\beta_3\cdotp {IC}_B}{k_1}$	1
*F*_1_	$\frac{k_0}{k_1}$	0.37
*F*_2_	$\frac{k_2\cdotp {IC}_B}{k_1\cdotp {IC}_S}$	10
*n*_1_	--	3
*n*_2_	--	2
*n*_3_	--	2
*n*_4_	--	5

## Results and Discussion

### A Bistable Switch between the Epithelial and Mesenchymal Phenotypes

**Fig. 2:**
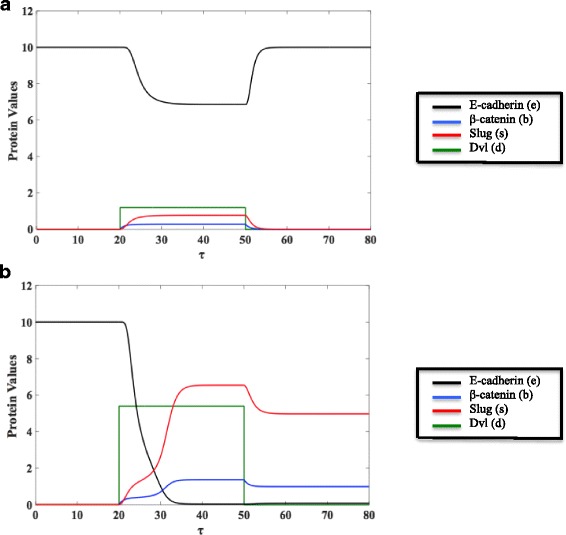
For τ = [0,20), the cell exists in the epithelial steady state where E-cadherin (*e*) level is high and both β-catenin (*b*) and Slug (*s*) are low. **a** If a small amount of Wnt signal is released by the microenvironment at *τ* = 20, a small amount of Dvl (*d* = 1.2) will accumulate at the membrane. E-cadherin will decrease slightly and both β-catenin and Slug will rise, but not enough to induce EMT. If, at *τ* = 50, the environment stops releasing Wnt signal, Dvl will detach from the membrane, meaning that the concentration of Dvl at the membrane will be 0 once again. Because EMT was not induced, the proteins all return to their initial epithelial values. **b** If enough Wnt signal is released by the microenvironment at *τ* = 20, enough Dvl will accumulate at the membrane (*d* = 5.4). The steady state values of β-catenin (*b*) and Slug (*s*) will rise and, thus, E-cadherin (*e*) will reach a very low steady state value. The lack of E-cadherin means that the cell will no longer be adhesive with the surrounding cells or its microenvironment, allowing it to move away from the primary tumor. If the concentration of membrane Dvl returns to 0 due to the cellular distance from the external Wnt signal at *τ* = 50, E-cadherin, β-catenin, and Slug will stabilize at values that allow the cell to maintain its mesenchymal state

The qualitative response of the epithelial cell to different levels of Wnt signaling is described in Fig. 2, which shows the changes in protein concentrations with respect to time. The values for each variable were included for reproducibility and the results are shown with the equivalent dimensional concentration, followed by the nondimensional parameter value. In both Fig. 2a and b, the cell exists in the epithelial steady state over the time range *τ* = [0, 20): E-cadherin level is high (0.33 nM, *e* = 10) whereas both β-catenin (*b*) and Slug (*s*) are low (< 0.05 nM, *b, s* < 0.015). At time *τ* = 20, Dvl is turned on, which implicitly models the release of Wnt protein from the surrounding environment and consequent movement of Dvl from the cytosol to the membrane. If Wnt signaling is very low, membrane Dvl will not be sufficiently high to induce EMT. This is illustrated in Fig. 2a (*d* = 1.2). The intracellular protein levels of β-catenin and Slug both increase (~0.1 nM, *b* ≈ 0.3; ~2.64 nM, *s* ≈ 0.8), but this results in only a dip in the membrane bound E-cadherin (~0.22 nM, *e* = 6.7), which allows the cell to maintain its adhesive nature and epithelial phenotype. Additionally, if the microenvironment were to then stop releasing the Wnt signal at time *τ* = 50, Dvl would detach from the membrane and return to the cytosol, meaning that the (nondimensional) concentration of membrane Dvl would return to its initial value of 0 (this is modeled by setting *d* = 0 at time *τ* = 50). Because the epithelial steady state was maintained (even with ~0.8 nM, *d* = 1.2), the proteins all go back to their original, pre-signaling epithelial levels.

If, however, the tumor microenvironment releases sufficient Wnt ligand, the amount of Dvl that accumulates at the membrane may be enough to induce significant changes in the proteins, as shown in Fig. 2b (3.62 nM, *d* = 5.4 at time *τ* = 20). With the activation of the Wnt pathway, free β-catenin accumulates, meaning that there is now a larger pool of β-catenin in the cytosol capable of translocating to the nucleus and upregulating Slug. Due to the rate limiting processes of translocation and the availability TCF/LEF, we witness the activation of Slug, as well the stabilization of β-catenin in the cytosol. Ultimately, both β-catenin and Slug reach much higher steady state values (~0.45 nM, *b* = 1.37; ~21.45 nM, *s* ≈ 6.5). The activation of the Wnt pathway and the subsequent changes in Slug and β-catenin are now enough such that there is a significant decrease in the steady state value of E-cadherin (~1.32 × 10^−3^ nM, *e* ≈ 0.04), meaning that β-catenin is not being as quickly sequestered into the adhesion complex, nor is it being as quickly degraded by the GSK-3β complex. Thus, β-catenin is able to continue to translocate to the nucleus. Even if membrane Dvl returns to 0 (as shown in Fig. 2b at *τ* = 50), the cell does not revert to the protein concentrations pertaining to the initial epithelial steady state, but has instead stabilized and maintains the protein levels of the converted mesenchymal state. In Fig. 2b, with *d* = 0 at *τ* = 50, E-cadherin is still maintained at a low value while Slug and β-catenin dip only slightly with the removal of the extracellular signal.

**Fig. 3:**
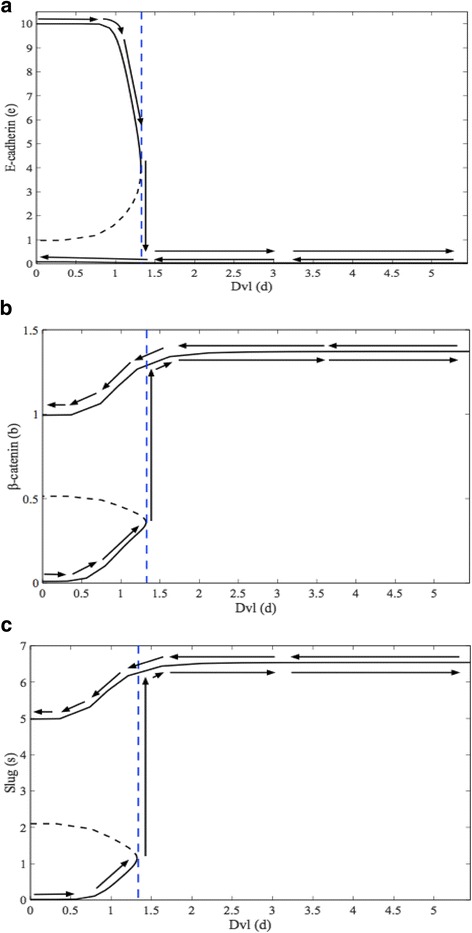
These bifurcation diagrams show a concentration-response curve of E-cadherin (Fig. 3 **a**), β-catenin (Fig. 3 **b**), and Slug (Fig. 3 **c**) with respect to membrane Dvl (*d*). If the cell starts in the epithelial steady state (*d* = 0), and the level of Dvl is steadily increased, the cell remains in the epithelial steady state until *d* = 1.33 (vertical blue dashed line). At this point, the cell undergoes EMT and transitions abruptly into a mesenchymal-like state. Once in the mesenchymal-like state, the cell (and its protein levels) will stay there, even after the level of membrane Dvl is decreased back to its initial value (*d* = 0). The bifurcation diagrams illustrate the bistable switch underlying the transition

The bifurcation diagrams in Fig. 3a-c give further insight into the behavior of E-cadherin, β-catenin, and Slug with respect to alterations in membrane-bound Dvl (*d*). These figures can be thought of as concentration-response curves of protein levels with respect to membrane Dvl. If the cell starts in the epithelial steady state, such as our system does in Fig. 3, and the level of membrane Dvl is steadily increased, the cell remains in the epithelial state, with high E-cadherin and low β-catenin and Slug levels, until Dvl reaches a threshold level of about 1.33 (blue vertical dashed line, Fig. 3a-c). At this point the cell undergoes EMT and transitions into a mesenchymal-like state with low E-cadherin and high β-catenin and Slug levels. For further increases of Dvl beyond 1.33, the cell remains in the switched mesenchymal state. However, when we start from the newly attained mesenchymal steady state and move leftward in Fig. 3 A-C, reducing the level of membrane Dvl, the cell remains in the mesenchymal steady state and does not switch back to the epithelial state. In fact, in this instance, with *d* = 0, the values that the proteins maintain are the steady state mesenchymal concentrations of the proteins at *τ* = 80 in Fig. 2b. There is thus a range of levels of Dvl (*d* = 0 to 1.33) for which the cell can exist in one of the two distinct steady states – epithelial or mesenchymal – depending on its history. This “cellular memory” mechanism generated by the bitable switch could explain how the EMT-derived mesenchymal cell is able to retain its invasive phenotype and not revert to an epithelial state even in the absence of sustained pro-EMT signaling from the microenvironment, for example in blood or lymphatic vessels. The switch mechanism also suggests that it is not the lack of an extracellular signal per se that forces the cells to revert to the epithelial phenotype in the metastatic environment (the mesenchymal to epithelial transition, MET), but rather a different extracellular signal in the new microenvironment is required to render the switch reversible.

### Sensitivity Analysis Shows E-cadherin is Influenced by Parameters Associated with Slug

To assess the sensitivity of the model to its various parameters, we carried out sensitivity analysis using a Latin Hypercube Sampling (LHS) [33] and Partial Ranked Correlation Coefficient (PRCC). LHS is a methodology that allows us to divide the range for each of our K parameters into N intervals and randomly sample a value from each interval [34]. Under the constraint that $N>\frac{4}{3}\bullet K$where *K* = 13 parameters in Eqs. (1)–(3) and *K* = 8 in Eqs. (4)–(6), it would be necessary to create at least 18 intervals to sample per parameter. However, in order to effectively sample the parameter space, 1 × 10^5^ intervals per parameter were created. Sampled values for each parameter are then randomly assembled together into N parameter sets and the model is run for each different set of parameters [35]. Once the LHS has been carried out, PRCC analysis permits us to transform our parameter input values and our outcomes into ranked values and measure the correlation between the rank-transformed input parameters and the rank-transformed outcomes [35]. As the Hill coefficients are all greater than 1 and are thus ultrasensitive terms in the model, by using LHS and PRCC, we sought to understand how the other parameters in the dimensional system influenced the steady state behaviors of E-cadherin (*E*), β-catenin (*B*), and Slug (*S*), as well as any differences that may occur when the system was nondimensionalized. Thus, the sensitivity analysis was carried out on both systems presented. By examining the sensitivity of the steady state behavior of our system to the nondimensional parameters, we were able to reduce the parameter space and highlight any hidden relationships that may exist between the parameters and the steady state behavior of the variables.

For all of the parameters in both systems, uniform distributions were used and sensitivity analysis was carried out at different values of Dvl. The ranges of the dimensional parameter values are provided in Table 3, while the ranges for the nondimensional parameters are listed in Table 4. After examining the partial correlation coefficients between each of the individual ranked parameter values at different values of Dvl, the parameters were found to be uncorrelated with each other. Scatterplots of the nondimensional steady state values for E-cadherin (*e*), β-catenin (*b*), and Slug (*s*) at different values of Dvl (*d*) in response to the nondimensional parameters are given in the Additional file 1: Fig. S1. These plots show the monotonic behavior of the variable steady states in response to changes in the different parameters, which is required to apply PRCC. Additionally, this monotonic behavior was found to hold in the dimensional system. The range of values considered for parameter *A*_*2*_ begins at 1 in Table 4. As shown in the Additional file 1, E-cadherin (*e*) and Slug (*s*) lack monotonic behavior in response to changes in *A*_*2*_. At *d = 0,* with *A*_*2*_ *ϵ* [0,1), *b* was found to be less than 0, which is not biologically realistic. While the addition of *d* to the system does reduce the range of *A*_*2*_ for which we see *b* < 0, in order to be able to compare changes in sensitivity across different levels of *d*, the entire range of *A*_*2*_ *ϵ* [0 1) was excluded from the analysis. Similar behavior was found with *α*_*2*_, and thus the parameter range *α*_*2*_ *ϵ* [0 0.01) was excluded from the analysis as well.

**Table 3:** Dimensional Parameter Ranges for Sensitivity Analysis

Parameter	Minimum Value	Maximum Value	Units
*D*	0	4	*nM*
*α*_1_	0	1.2 × 10^−1^	$\frac{nM}{\mathit{\min}}$
*α*_2_	1.0 × 10^−2^	2.0 × 10^−2^	$\frac{nM}{\mathit{\min}}$
*α*_3_	0	2.0 × 10^−1^	$\frac{nM}{\mathit{\min}}$
*β*_1_	0	1.515 × 10^−1^	$\frac{1}{\mathit{\min}}$
*β*_2_	0	7.576 × 10^−2^	$\frac{1}{\mathit{\min}}$
*β*_3_	0	7.576 × 10^−2^	$\frac{1}{\mathit{\min}}$
*k*_0_	0	2.5 × 10^−2^	$\frac{nM}{\mathit{\min}}$
*k*_1_	4.0 × 10^−3^	1.0 × 10^−2^	$\frac{nM}{\mathit{\min}}$
*k*_2_	0	2.5	$\frac{nM}{\mathit{\min}}$
*IC*_*S*_	1.65 × 10^−2^	10.0	*nM*
*IC*_*B*_	0	8.25 × 10^−1^	*nM*
*IC*_*E*_	2.75 × 10^−3^	2.0 × 10^−1^	*nM*
*IC*_*D*_	0	5.0	*nM*

**Table 4:** Nondimensional Parameter Ranges for Sensitivity Analysis

Parameter	Minimum Value	Maximum Value
*d*	0	6
*A*_*1*_	0	120
*A*_*2*_	1	2
*A*_*3*_	0	2
*C*_*1*_	0	5
*C*_*2*_	0	2.5
*C*_*3*_	0	2.5
*F*_*1*_	0	2.5
*F*_*2*_	0	25

**Fig. 4:**
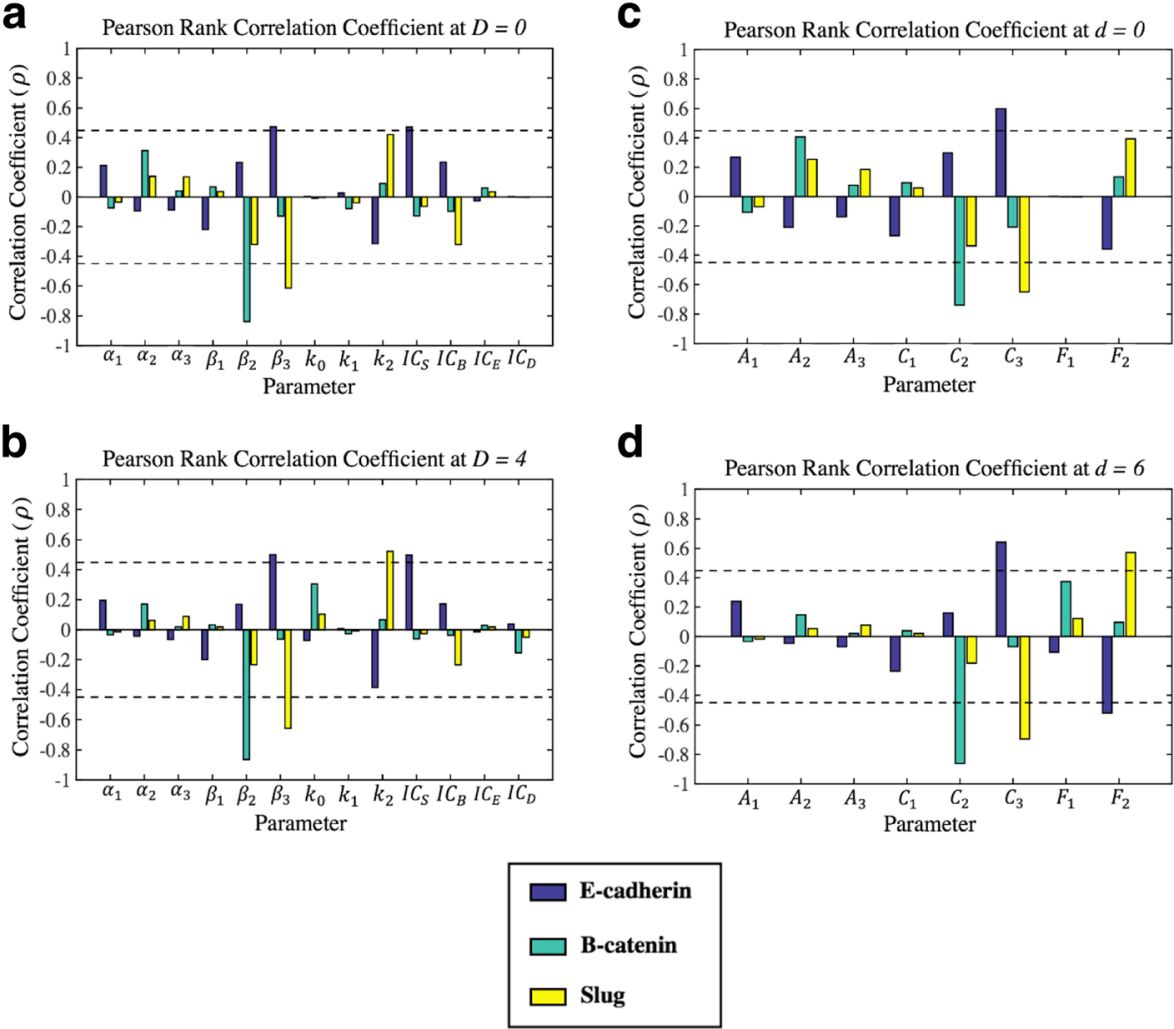
Sensitivity analysis was carried out using Latin Hypercube and Pearson’s Ranked Correlation Coefficient to understand the relationship between the steady state behavior of E-cadherin, β-catenin, and Slug and the system parameters at different levels of Dvl. The dimensional model was explored in (**a** and **b**) while the nondimensional model was explored in (**c** and **d**). A system without Wnt activation is shown in (**a ** and **c**). (Fig. 4 **a**) Only *β*_2_ is significantly correlated (correlation coefficient (*ρ*) < −0.45, *p*-value <0.05) with the steady state behavior of β-catenin (*B*), while *β*_3_ is significantly correlated (correlation coefficient (*ρ*) < −0.45 or correlation coefficient (*ρ*) > 0.45, *p*-value <0.05) with the steady state behavior of E-cadherin (*E*) and Slug (*S*). Additionally, *IC*_*S*_ is significantly correlated with the steady state behavior of E-cadherin (*E*). (Fig. 4**b**): With the activation of the Wnt pathway, the rate at which β-catenin (*B*) translocates to the nucleus and activates Slug (*S*), *k*_*2*_, becomes significantly correlated with the steady state value Slug (s). (Fig. 4**c**) Only *C*_2_ is significantly correlated (correlation coefficient (*ρ*) < −0.45, *p*-value <0.05) with β-catenin (*b*)‘s steady state behavior, while *C*_3_ is significantly correlated (correlation coefficient (*ρ*) < −0.45 or correlation coefficient (*ρ*) > 0.45, *p*-value <0.05) with the steady state behavior of E-cadherin (*e*) and Slug (*s*). (Fig. 4**d**) With the activation of the Wnt pathway, the nondimensional rate at which β-catenin (*b*) translocates to the nucleus and activates Slug (*s*), *F*_*2*_, becomes significantly correlated with the steady state values of E-cadherin (*e*) Slug (*s*). This sensitivity of E-cadherin (*e*) to *F*_*2*_ but not *k*_*2*_ in (Fig. 4b) indicates that the steady state behavior of E-cadherin may be sensitive to the ratio of the rate at which β-catenin activates Slug to the IC_50_ value of Slug needed to inhibit E-cadherin production

Figure 4 shows the PRCC analysis for the parameters with the steady state values for E-cadherin, β-catenin, and Slug both prior to and during Wnt signaling. In Fig. 4a and b, the relationship between the dimensional parameters in Eqs. (1) - (3) and the steady state values of *E, B,* and *S* at Dvl (*D*) = 0 (4A) and Dvl (*D*) = 4 (4B) are examined. For both values of Dvl (*D*), the β-catenin-GSK-3β complex binding rate (*β*_*2*_) influences the steady state concentration of free cytosolic β-catenin (*B*) while the degradation rate of Slug, *β*_*3*_, significantly influences the steady state concentrations of E-cadherin (*E*) and Slug (*S*). These sensitivity relationships are preserved after the nondimensionalization is carried out and we examine the relationship between the nondimensional parameters of Eqs. (4) – (6) to the steady state behavior of *e*, *b,* and *s*. As shown in Figs. 4c and d, the steady state behavior of β-catenin (*b*) is dependent upon the nondimensional rate at which it binds to the GSK-3β complex (*C*_*2*_), while the steady state behaviors of E-cadherin (*e*) and Slug (*s*) are both sensitive to the nondimensional degradation rate of Slug (*C*_*3*_).

One interesting difference between the parameter analysis of the dimensional model and the nondimensional model is observed when examining sensitivity changes that occur after Wnt signaling is activated. In Fig. 4a, in the absence of Wnt signaling, the parameter *k*_*2*_ is not significantly correlated with any of the dimensional variables. But, with an increase in the active membrane-bound Dvl in Fig. 4b, *k*_*2*_ significantly influences the steady state concentration of Slug. Similarly, in Fig. 4c, the concentrations of the nondimensional steady state concentrations are not significantly correlated with changes in the *F*_*2*_ parameter, which is defined as ${F}_2=\frac{k_2\cdotp {IC}_B}{k_1\cdotp {IC}_S}$ in Table 2 and thus includes the *k*_*2*_ parameter. With the activation of the Wnt pathway in Fig. 4d (*d* = 6), changes in *F*_*2*_ become significantly correlated with the steady state concentrations of Slug (*s*) *and* E-cadherin (*e*). This additional sensitivity of E-cadherin (*e*) to changes in *F*_*2*_ could be due to the underlying sensitivity to the parameter *IC*_*S*_, which exists in the denominator of *F*_*2*_ and was shown to influence the concentration of *E* in the dimensional model. However, due to the sensitivity of E-cadherin (*E*) to changes in *IC*_*S*_ in Fig. 4a, it would be expected that the nondimensional concentration of E-cadherin (*e*) would be sensitive to changes in *F*_*2*_ prior to Wnt signaling (*d* = 0), which is not the case. This sensitivity discrepancy suggests that, with the activation of the Wnt pathway and the increase in cytosolic β-catenin, the ratio of the rate at which β-catenin is able to activate Slug to the IC_50_ value of Slug necessary to inhibit E-cadherin production is influential in the steady state concentration of E-cadherin.

The sensitivity relationships in Fig. 4 suggest that the protein that is both responsible for cell-to-cell adhesion and is a characteristic marker of the epithelial phenotype, E-cadherin, is heavily influenced by changes in the concentration of Slug that is present. Even before Wnt signaling is activated, the concentration of E-cadherin present at the membrane for cell-to-cell contact is susceptible to changes in the rate at which Slug is being destroyed and the IC_50_ value of Slug necessary to inhibit production of E-cadherin. With the activation of the Wnt signaling pathway, the sensitivity of the level of Slug to the translocation of β-catenin to the nucleus changes. Now, with an external stimulus and activation of the Wnt pathway, the change in the compartmentalization of β-catenin from the cytosol to the nucleus significantly influences changes in the steady state behavior of Slug, which is then capable of overcoming the IC_50_ value necessary to inhibit E-cadherin production. This change in significance for Slug, as well as the sensitivity of E-cadherin to *IC*_*S*_ and the ratio of *k*_*2*_ to *IC*_*S*_, highlights the possibility of therapeutically targeting nuclear events for the prevention of the change from the epithelial to the mesenchymal phenotype, as well as the possibility that increasing the *IC*_*S*_ value may inhibit the switch once the pathway has been activated.

### Two-Parameter Bifurcations Highlight Cellular Rates important to EMT and the Bistable Switch

**Fig. 5:**
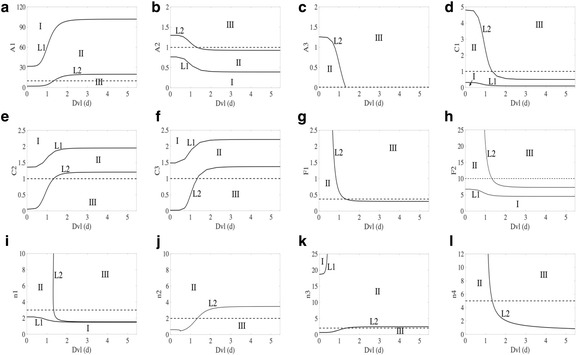
In each subfigure, Dvl (d) is varied along the x-axis. Along the y-axis, the nondimensional parameters *A*_*1*_ (**a**), *A*_*2*_ (**b**), *A*_*3*_ (**c**), *C*_*1*_ (**d**), *C*_*2*_ (**e**), *C*_*3*_ (**f**), *F*_*1*_ (**g**), *F*_*2*_ (**h**), *n*_*1*_ (**i**), *n*_*2*_ (**j**), *n*_*3*_ (**k**), *n*_*4*_ (**l**) are varied one at a time. For those parameter values where the cell begins in Region I and remains in Region I, or crosses L1 into Region II, as Dvl (d) is increased, the cell is committed to the epithelial steady state. For those parameter values where the cell begins in Region III and remains in Region III, or crosses L2 into Region II, as Dvl changes, the cell is committed to the mesenchymal steady state. For parameter values that allow the cell to begin in region two, the starting steady state depends on the system’s initial conditions. If the cell begins in the epithelial steady state and crosses L2 into Region III with a change in Dvl (d), the cell will switch to the mesenchymal steady state. If the cell begins in the mesenchymal steady state in Region II and crosses L1 into Region I with changes in Dvl (d), the cell will switch to the epithelial steady state. The parameter values used in this system are marked with a dashed line. In this model, the cell begins in Region II with epithelial initial conditions

To further understand how changes in the parameters influence the system, particularly the bistable switch behavior, nondimensional two-parameter bifurcation diagrams were generated using XPPAUT. Shown in Fig. 5, these plots depict the steady state phenotype of the simulated cell over the parameter space. Along the x-axis of each plot, Dvl (*d*) is varied. Along the y-axis, the non-dimensional parameters *A*_*1*_ *, A*_*2*_ *, A*_*3*_ *, C*_*1*_ *, C*_*2*_ *, C*_*3*_ *, F*_*1*_ *, F*_*2*_, *n*_*1*_, *n*_*2*_, *n*_*3*_, and *n*_*4*_ are varied one at a time. In each of the figures, the cell can only be in the epithelial steady state for parameter values in Region I and can only be in the mesenchymal steady state in Region III. In Region II, either steady state is possible depending on the history of the cell. If the system begins in the epithelial steady state (Region I) and crosses the L1 boundary into Region II, the cell will remain in the epithelial steady state. Similarly, if the cell begins in the mesenchymal steady state (Region III) and crosses the L2 boundary into Region II, it will remain in the mesenchymal steady state. If, based on the parameter values, the system begins in Region II, it will depend upon the initial conditions whether or not the cell assumes an epithelial or mesenchymal phenotype. The nondimensional parameter value used in this model is indicated by a dashed line in each subplot of Fig. 5. For these parameter values, the cell begins as an epithelial cell in Region II. Once the concentration of Dvl (*d*) is increased such that the L2 boundary is crossed into Region III, the cell will make the transition from epithelial to mesenchymal. If, however, the concentration of *d* is then reduced at this parameter value, the newly formed mesenchymal cell would move back into Region II and conserve its mesenchymal phenotype due to the fact that the cell was unable to cross L1. Hypothetically, if a cell begins as a mesenchymal cell in Region II and the concentration of Dvl (*d*) is decreased so that the L1 boundary is crossed, the cell will transition from a mesenchymal cell into an epithelial cell as the system transitions from Region II to Region I. The region of bistability (i.e. Region II) is seen to be fairly robust to parameter variation.

Note that, in Fig. 5g, once F_1_ overcomes a certain threshold value, the system must cross over L2 and pass from Region II to Region III with the addition of enough Dvl (*d*) to the system. This behavior indicates that an epithelial cell beginning in Region II must switch to the mesenchymal phenotype so long as enough Wnt signaling is applied. As F_1_ represents the nondimensional rate in which Dvl inactivates the GSK-3β degradation complex, this result suggests that, at if the rate at which Dvl inhibits the β-catenin degradation is high enough, so long as enough Dvl is active at the membrane, β-catenin will accumulate in the cytosol and the cell will be forced from the epithelial to the mesenchymal phenotype.

Similar behavior occurs in Fig. 5h with F_2_. For a high value of F_2_, in the presence of sufficient Wnt signaling, an epithelial cell must undergo the bistable switch and enter the mesenchymal steady state. This behavior is particularly interesting in light of what we now know about the sensitivity of the Slug and E-cadherin steady state concentrations to changes in F_2_, as well as the relationship between F_2_ and *k*_*2*_ and *IC*_*S*_. With the activation of the Wnt signal and the movement of Dvl to the membrane, if the rate at which β-catenin moves to the nucleus and binds with TCF is fast enough or the IC_50_ concentration necessary to inhibit E-cadherin production is low enough, Slug will be able to accumulate to a concentration sufficient to force the cell to switch from the epithelial to the mesenchymal phenotype.

## Conclusions

The ability of a carcinoma cell to adopt the mesenchymal phenotype due to external signals from the microenvironment and conserve the newly acquired invasive properties in the absence of an extracellular cue suggests that the switch from the epithelial to the mesenchymal state is bistable in situ. While multiple intracellular signaling pathways can stimulate EMT, we consider Wnt signaling as a case study. The Wnt signaling pathway has been under intense scrutiny from both mathematicians and biologists in order to understand how it contributes to changes in cellular behavior. Mathematical models attempting to describe the intracellular pathway are often complex and limited to one group of protein interactions. Instead of examining one subset of the Wnt pathway, the model presented in this work examines the three key relationships centered around β-catenin that comprise the Wnt signaling pathway and drive the change in cellular phenotype. Studying the system as a whole provides us a better understanding of which interactions are likely to be responsible for the bistable switch.

This model opens up many avenues for possible theoretical and practical exploration. The bistable switch proposed in this model is an all or nothing change in behavior: the cell can either occupy the epithelial or the mesenchymal steady state. However, recent work has suggested that the transition may, instead, be a gradient, whereby the cell can occupy at least one (if not more) intermediate states as it progresses towards the mesenchymal phenotype [6]. Using a scaffold model to expand upon the complicated mechanisms involved in each step of Wnt pathway activation, as well as pathway crosstalk, may indeed reveal intermediate steady states for the cell to occupy during the transition.

With the theory of a switch underlying EMT, it would also be prudent to examine how the transition is affected by the presence of neighboring cells. The stabilization of these bonds could ultimately work against the loss of adhesion in the epithelial cell and the resulting transformation. Additionally, sensitivity analysis and exploration of the response of the system to changes in parameter values as Wnt signaling is activated suggests that excellent candidates for possible therapeutic intervention are the β-catenin degradation complex, the shuttling of β-catenin to the nucleus, and the *IC*_50_ value of Slug necessary to inhibit E-cadherin production. While the steady state behavior of free β-catenin was shown to be sensitive to its own rate of degradation, the steady state behavior of E-cadherin is actually sensitive to changes in Slug. In particular, E-cadherin is sensitive to the degradation of Slug and the half maximal concentration of Slug necessary to inhibit E-cadherin production, as well as the ratio of rate of Slug activation via β-catenin to the half-maximal concentration of Slug necessary to inhibit E-cadherin production once the pathway has been activated. These insights, coupled with the behavior of the system in response to changes in both the F_2_ parameter and Dvl (*d*) activation in Fig. 5h, suggests that continued exploration of how the switch is affected by the presence of neighboring cells, as well as the formation of the E-cadherin- β-catenin complex, is necessary. The stabilization of intercellular bonds could ultimately work against the loss of adhesion in the epithelial cell and the resulting transformation. Possible avenues for practical exploration would be therapeutically raising the half-maximal concentration of Dvl necessary to inhibit β-catenin degradation or lowering the rate at which β-catenin translocates to the nucleus. Therapies targeted at the IC_50_ concentration of Slug necessary to inhibit E-cadherin production, as well as inhibiting the movement of β-catenin to the nucleus could prevent the activation of Slug and ultimately work to maintain the E-cadherin- β-catenin complex at the membrane. Pharmacological exploration of these components of the Wnt pathway could help prevent EMT prior to intravasation and metastasis, the primary cause of cancer-related mortality.

## Additional file

Additional file 1: Figure S1. Figures S1A-S1EN show the monotonic behavior for each of the three nondimensional variables (*e, b, s*) in response to changes in the 8 nondimensional parameters at select values of Dvl (*d*). Figures in the left column show the parameter varied over its parameter space in a system with epithelial initial conditions while figures in the right column show the individual parameter varied over its parameter space with mesenchymal initial conditions (DOCX 25562 kb)

## References

[1] Weinberg R. The biology of cancer: Garland science; 2013.

[2] Lee JM, Dedhar S, Kalluri R, Thompson EW (2006). The epithelial-mesenchymal transition: new insights in signaling, development, and disease. J Cell Biol.

[3] Onder TT, Gupta PB, Mani SA, Yang J, Lander ES, Weinberg RA (2008). Loss of E-cadherin promotes metastasis via multiple downstream transcriptional pathways. Cancer Res.

[4] Christofori G, Semb H (1999). The role of the cell-adhesion molecule E-cadherin as a tumour-suppressor gene. Trends Biochem Sci.

[5] Nieto MA, Huang RYJ, Jackson RA, Thiery JP (2016). EMT: 2016. Cell.

[6] Kalluri R, Weinberg RA (2009). The basics of epithelial-mesenchymal transition. J Clin Investig.

[7] Mani SA, Guo W, Liao MJ, Eaton EN, Ayyanan A, Zhou AY, Brooks M, Reinhard F, Zhang CC, Shipitsin M (2008). The epithelial-mesenchymal transition generates cells with properties of stem cells. Cell.

[8] Thiery JP (2002). Epithelial-mesenchymal transitions in tumour progression. Nat Rev Cancer.

[9] Kogan Y, Halevi-Tobias KE, Hochman G, Baczmanska AK, Leyns L, Agur Z (2012). A new validated mathematical model of the Wnt signalling pathway predicts effective combinational therapy by sFRP and Dkk. Biochem J.

[10] Lloyd-Lewis B, Fletcher AG, Dale TC, Byrne HM (2013). Toward a quantitative understanding of the Wnt/−catenin pathway through simulation and experiment. Wiley Interdiscip Rev-Syst Biol.

[11] Chen JW, Xie ZR, Wu YH (2014). Computational Modeling of the Interplay between Cadherin-Mediated Cell Adhesion and Wnt Signaling Pathway. PLoS One.

[12] Lee E, Salic A, Kruger R, Heinrich R, Kirschner MW (2003). The roles of APC and axin derived from experimental and theoretical analysis of the Wnt pathway. PLoS Biol.

[13] Ramis-Conde I, Drasdo D, Anderson ARA, Chaplain MAJ (2008). Modeling the influence of the E-cadherin-beta-catenin pathway in cancer cell invasion: A multiscale approach. Biophys J.

[14] Basan M, Idema T, Lenz M, Joanny JF, Risler T (2010). A Reaction-Diffusion Model of the Cadherin-Catenin System: A Possible Mechanism for Contact Inhibition and Implications for Tumorigenesis. Biophys J.

[15] Agur Z, Kirnasovsky OU, Vasserman G, Tencer-Hershkowicz L, Kogan Y, Harrison H, Lamb R, Clarke RB (2011). Dickkopf1 Regulates Fate Decision and Drives Breast Cancer Stem Cells to Differentiation: An Experimentally Supported Mathematical Model. PLoS One.

[16] Kim D, Rath O, Kolch W, Cho KH (2007). A hidden oncogenic positive feedback loop caused by crosstalk between Wnt and ERK Pathways. Oncogene.

[17] Shin SY, Rath O, Zebisch A, Choo SM, Kolch W, Cho KH (2010). Functional Roles of Multiple Feedback Loops in Extracellular Signal-Regulated Kinase and Wnt Signaling Pathways That Regulate Epithelial-Mesenchymal Transition. Cancer Res.

[18] MacLean AL, Rosen Z, Byrne HM, Harrington HA (2015). Parameter-free methods distinguish Wnt pathway models and guide design of experiments. Proc Natl Acad Sci U S A.

[19] Jolly MK, Tripathi SC, Jia D, Mooney SM, Celiktas M, Hanash SM, Mani SA, Pienta KJ, Ben-Jacob E, Levine H (2016). Stability of the hybrid epithelial/mesenchymal phenotype. Oncotarget.

[20] Jolly MK, Huang B, Lu MY, Mani SA, Levine H, Ben-Jacob E (2014). Towards elucidating the connection between epithelial - mesenchymal transitions and stemness. J R Soc Interface.

[21] Hong T, Watanabe K, Ta CH, Villarreal-Ponce A, Nie Q, Dai X (2015). An Ovol2-Zeb1 Mutual Inhibitory Circuit Governs Bidirectional and Multi-step Transition between Epithelial and Mesenchymal States. PLoS Comput Biol.

[22] Benary U, Kofahl B, Hecht A, Wolf J (2013). Modeling Wnt/beta-catenin target gene expression in APC and Wnt gradients under wild type and mutant conditions. Front Physiol.

[23] Heuberger J, Birchmeier W (2010). Interplay of Cadherin-Mediated Cell Adhesion and Canonical Wnt Signaling. Cold Spring Harb Perspect Biol.

[24] Schmitz Y, Rateitschak K, Wolkenhauer O (2013). Analysing the impact of nucleo-cytoplasmic shuttling of beta-catenin and its antagonists APC, Axin and GSK3 on Wnt/beta-catenin signalling. Cell Signal.

[25] Mosimann C, Hausmann G (2009). Basler K: **beta-Catenin hits chromatin: regulation of Wnt target gene activation**. Nat Rev Mol Cell Biol.

[26] Chaffer CL, Weinberg RA (2011). A Perspective on Cancer Cell Metastasis. Science.

[27] Kalluri R, Zeisberg M (2006). Fibroblasts in cancer. Nat Rev Cancer.

[28] Clevers H, Nusse R (2012). Wnt/beta-Catenin Signaling and Disease. Cell.

[29] Daniels DL, Weis WI (2005). beta-catenin directly displaces Groucho/TLE repressors from Tcf/Lef in Wnt-mediated transcription activation. Nat Struct Mol Biol.

[30] Shirley SH, Hudson LG, He J, Kusewitt DF (2010). The Skinny on Slug. Mol Carcinog.

[31] George SJ, Dwivedi A (2004). MMPs, cadhenins, and cell proliferation. Trends Cardiovasc Med.

[32] XPPAUT – the differential equations tool, version 6.10 [http://www.math.pitt.edu/~bard/xpp/xpp.html].

[33] McKay MD, Beckman RJ, Conover WJ (1979). A comparison of three methods for selecting values of input variables in the analysis of output from a computer code. Technometrics.

[34] Blower SM (1994). Dowlatabadi H: **sensitivity and uncertainty analysis of complex-models of disease transmission - an hiv model, as an example**. Int Stat Rev.

[35] Gomero B: Latin Hypercube Sampling and Partial Rank Correlation Coefficient Analysis Applied to an Optimal Control Problem. University of Tennessee; 2012.

