# Supplementary material for: A Theoretical Model of the Wnt Signaling Pathway in the Epithelial Mesenchymal Transition
Source: Theor Biol Med Model. 2017 Oct 10;14:19. doi: 10.1186/s12976-017-0064-7 (PMC5634852; doi:10.1186/s12976-017-0064-7)
Supplement: Additional file 1: Figure S1. — Figures S1A-S1EN show the monotonic behavior for each of the three nondimensional variables (e, b, s) in response to changes in the 8 nondimensional parameters at select values of Dvl (d). Figures in the left column show the parameter varied over its parameter space in a system with epithelial initial conditions while figures in the right column show the individual parameter varied over its parameter space with mesenchymal initial conditions (DOCX 25562 kb) [file 12976_2017_64_MOESM1_ESM.docx]

| 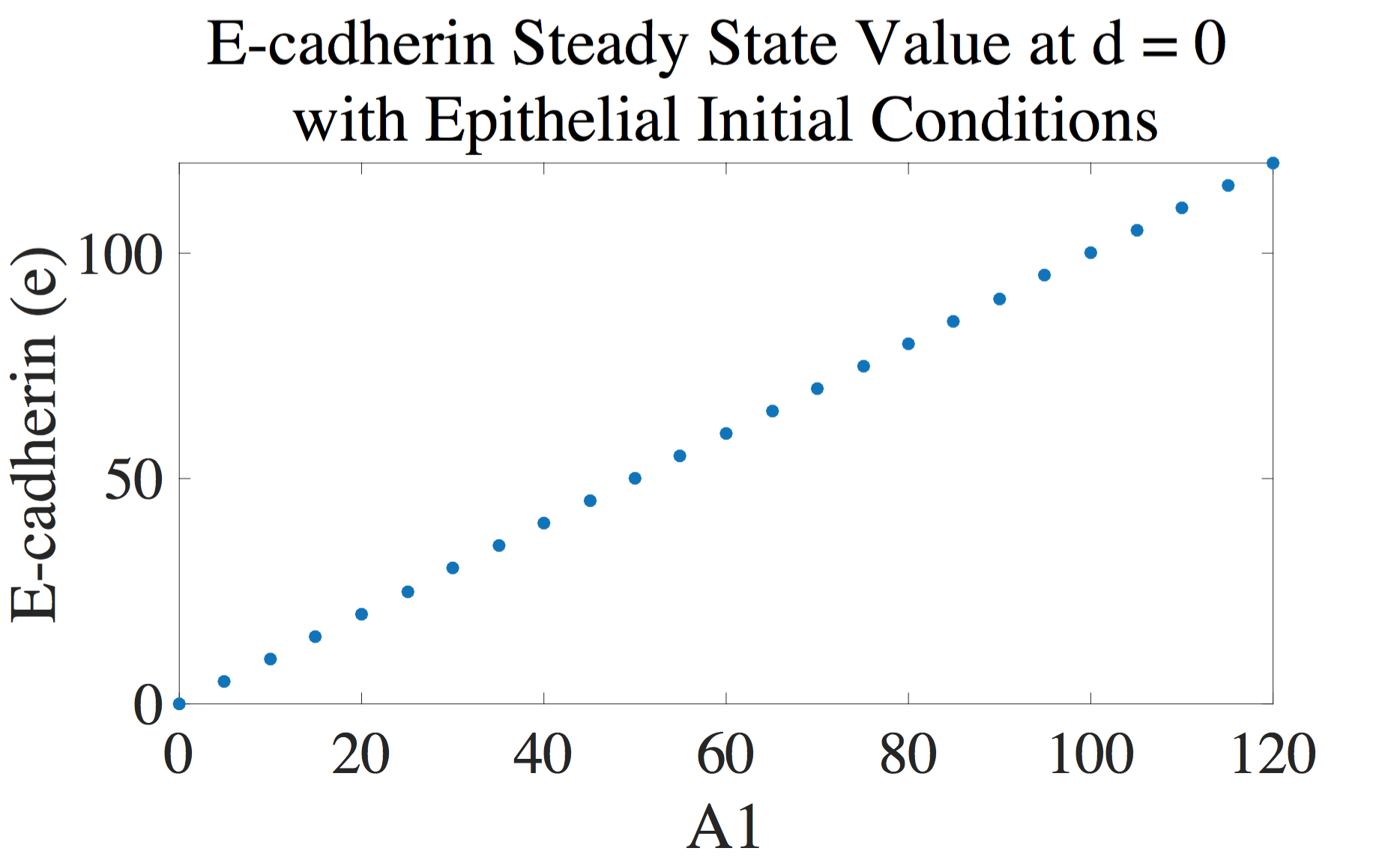 | 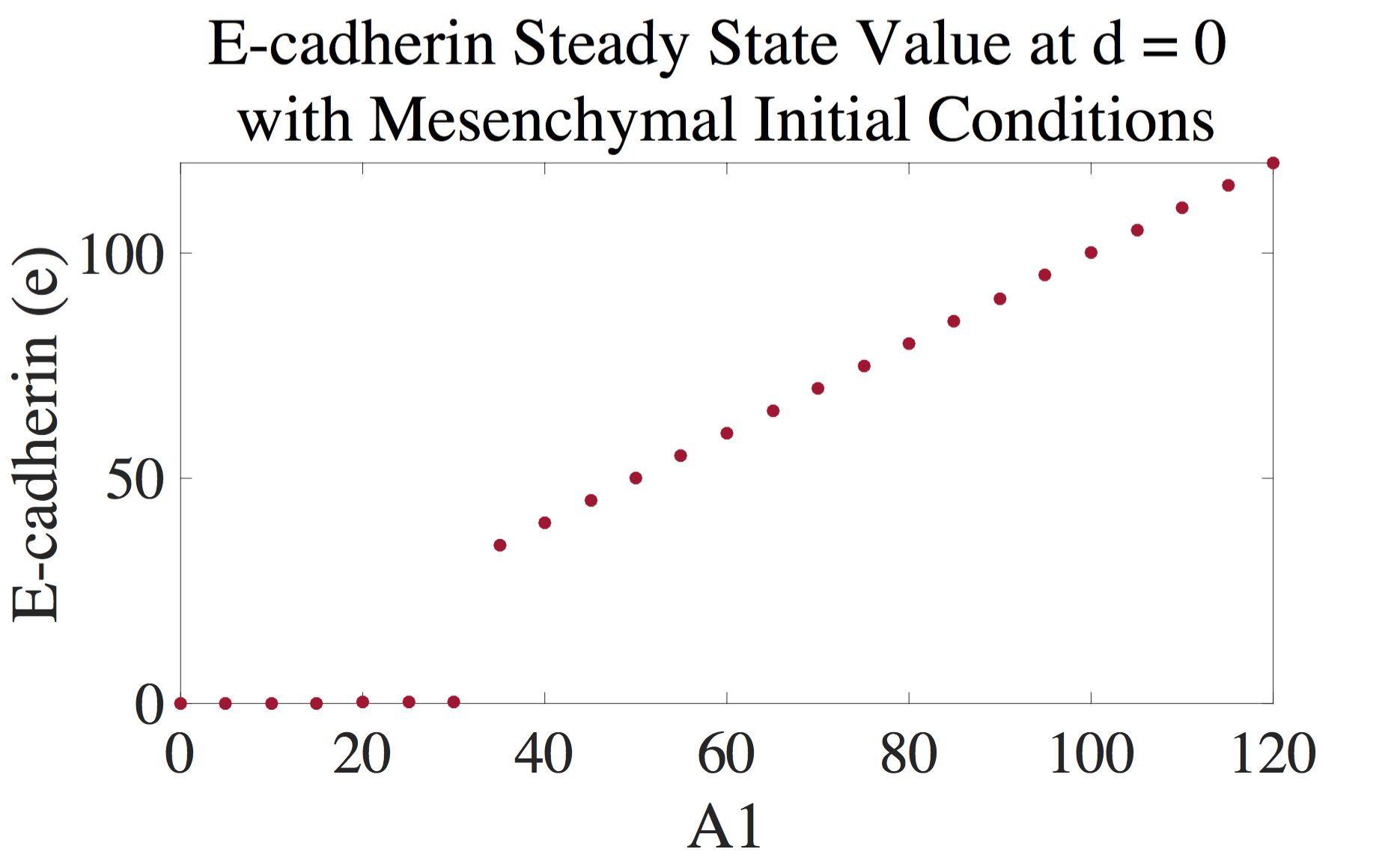 |
| --- | --- |
| Figure S1A | Figure S1B |
|  |  |
| 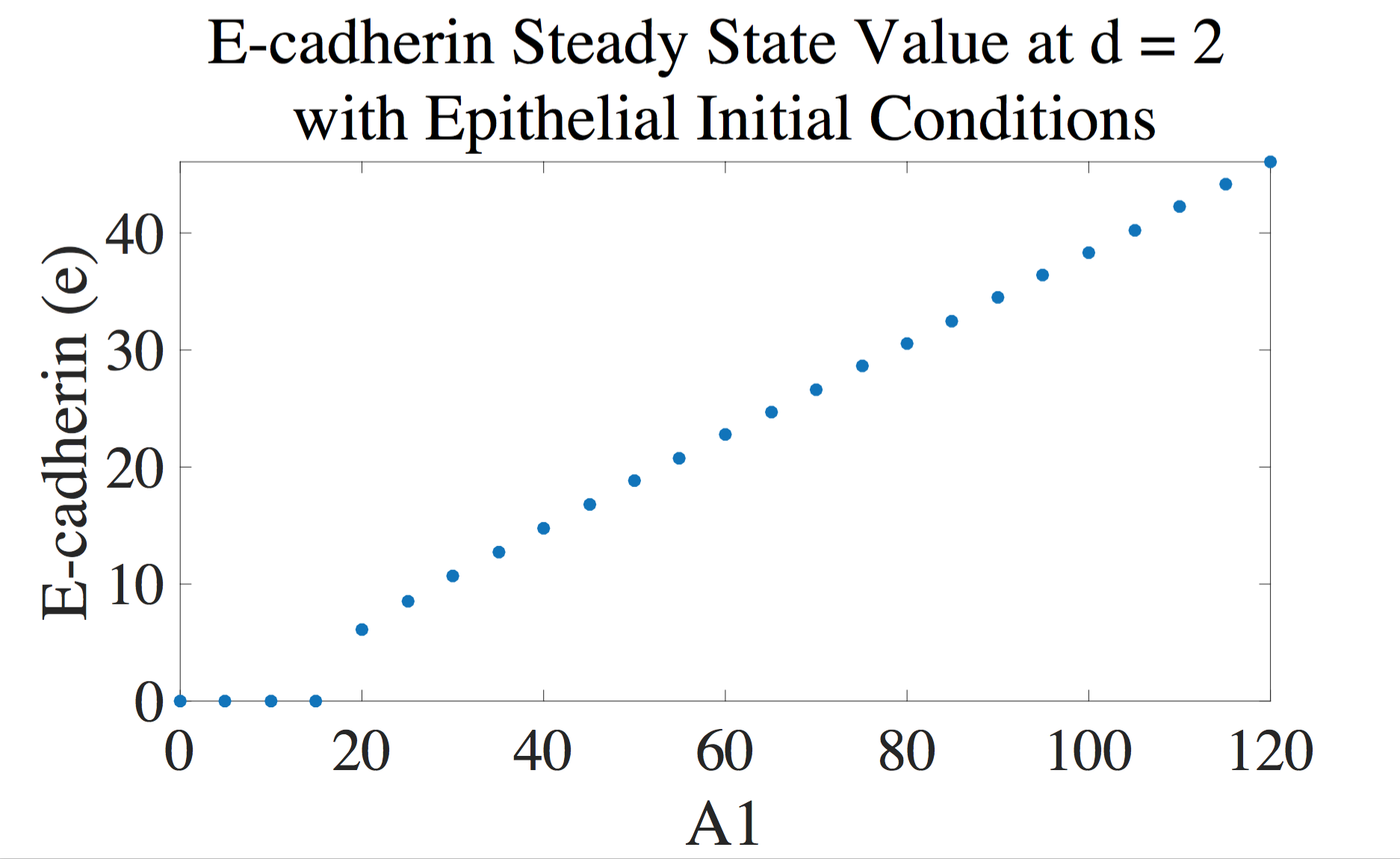 | 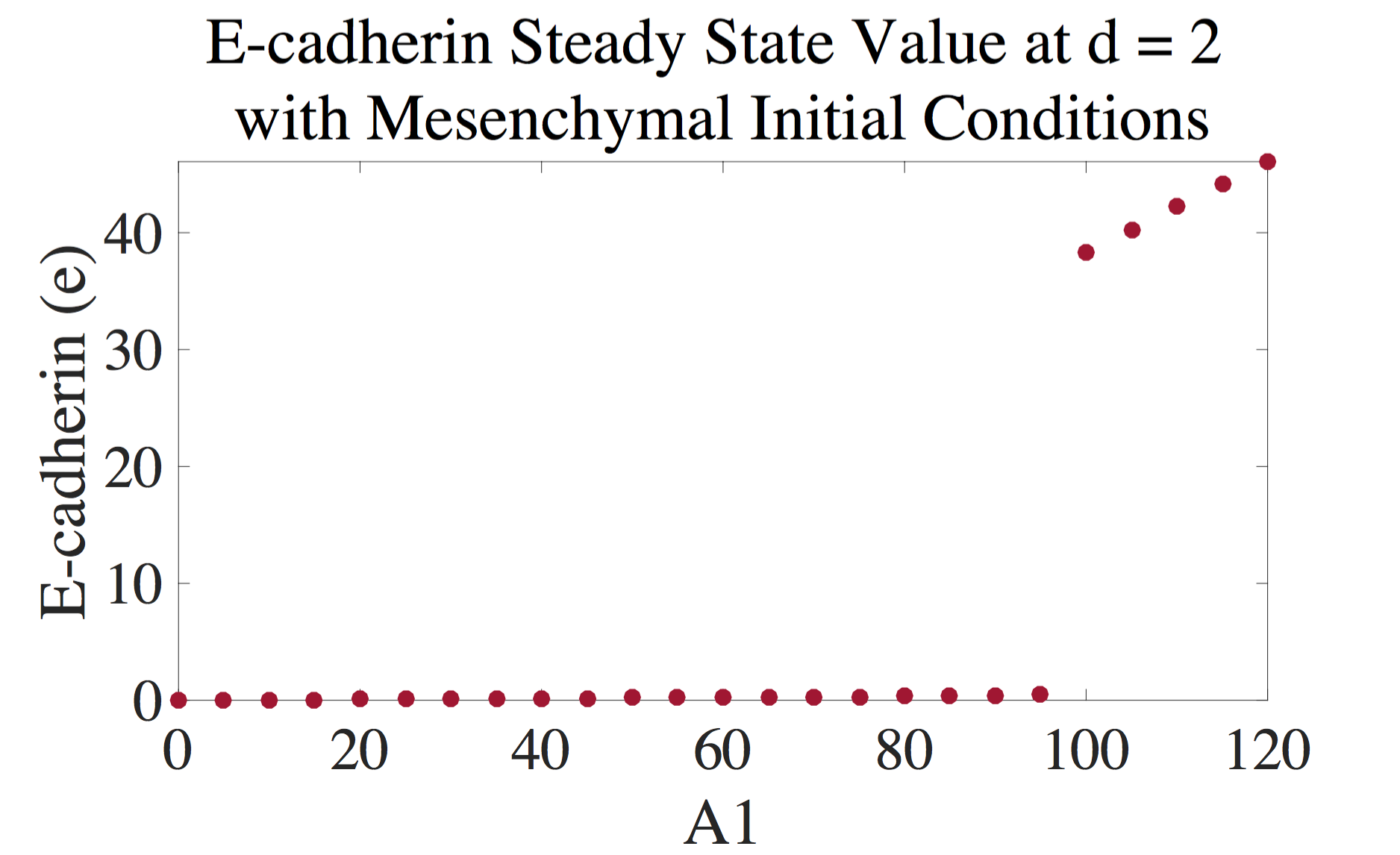 |
| Figure S1C | Figure S1D |
|  |  |
| 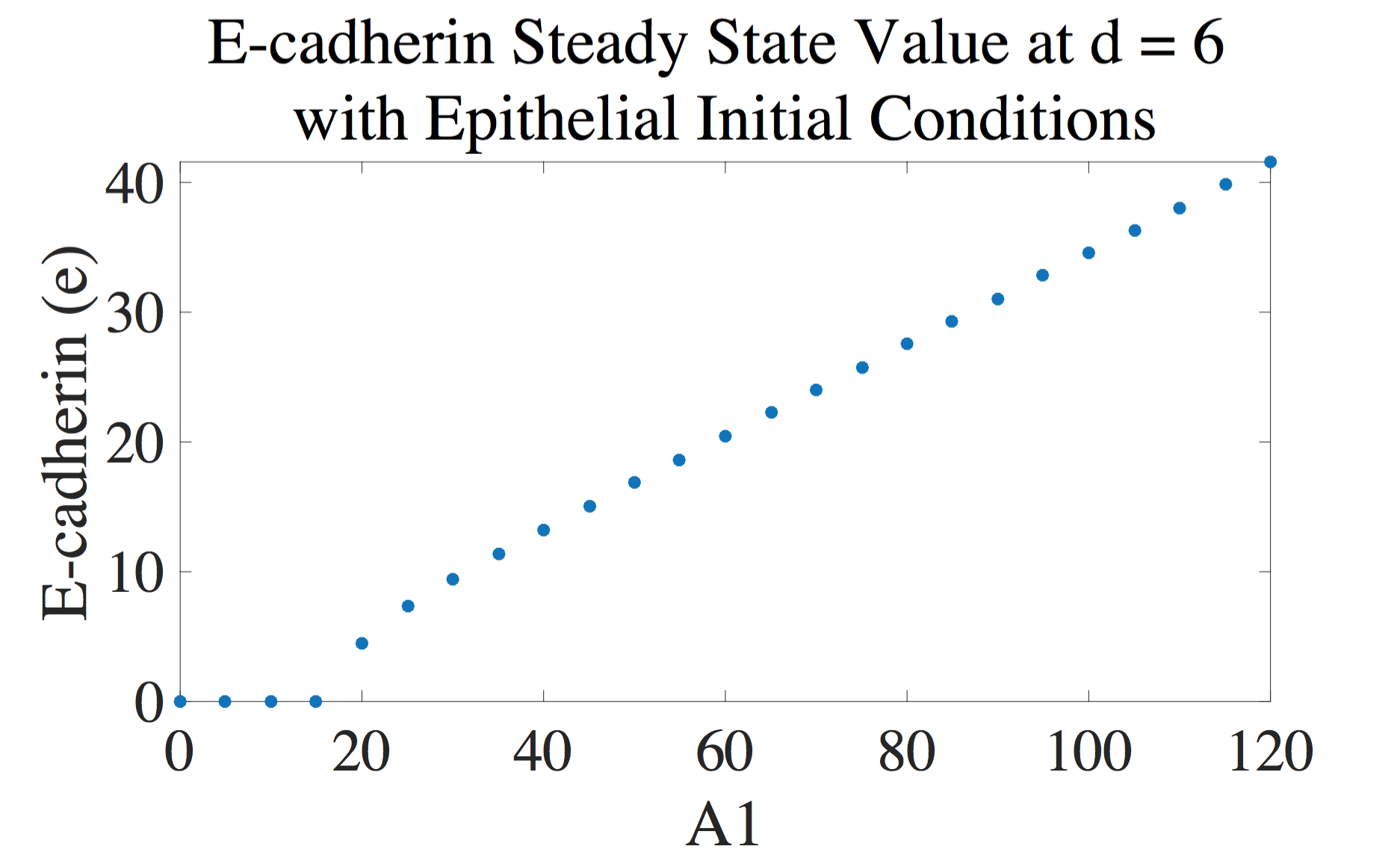 | 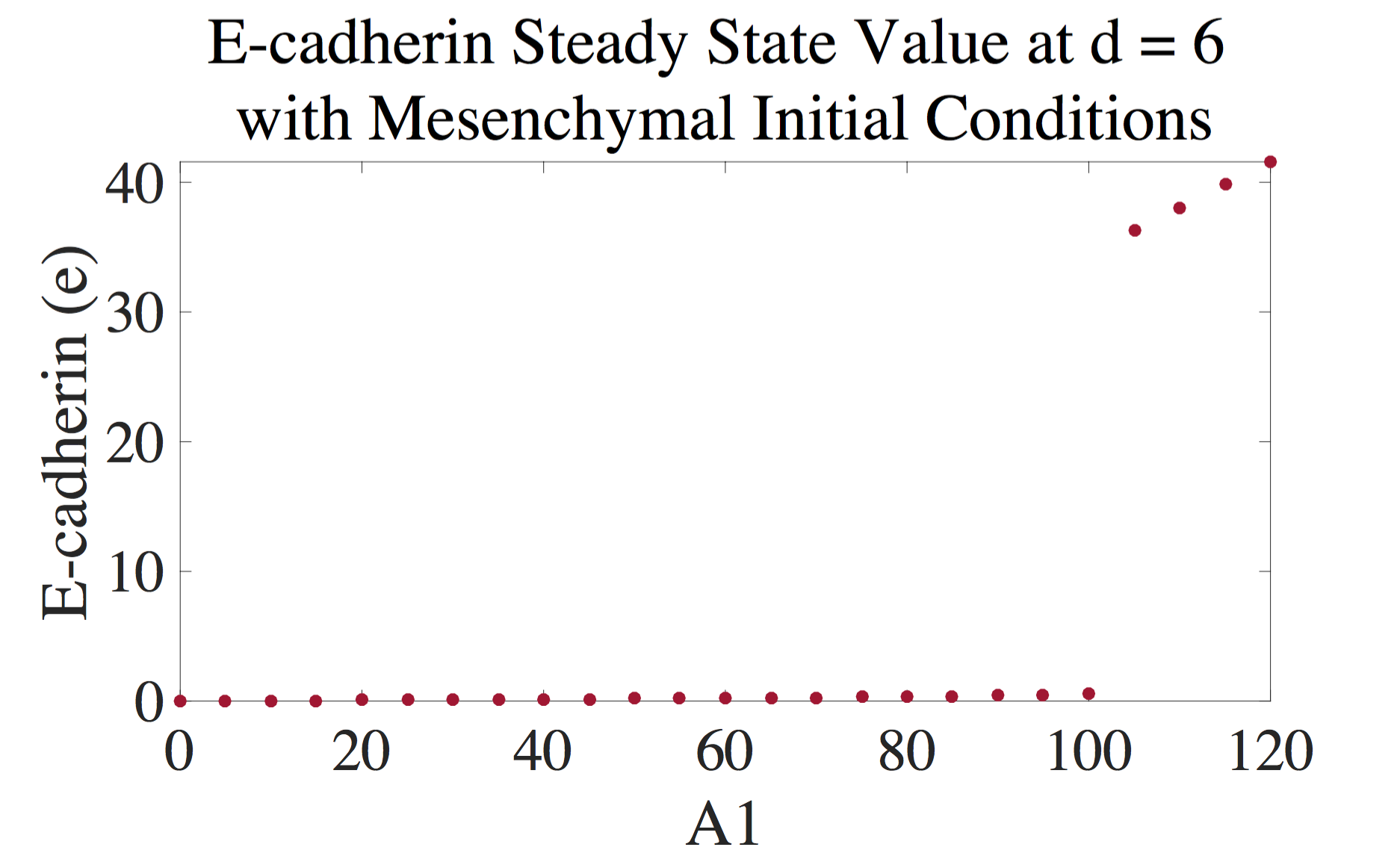 |
| Figure S1E | Figure S1F |
|  |  |
| 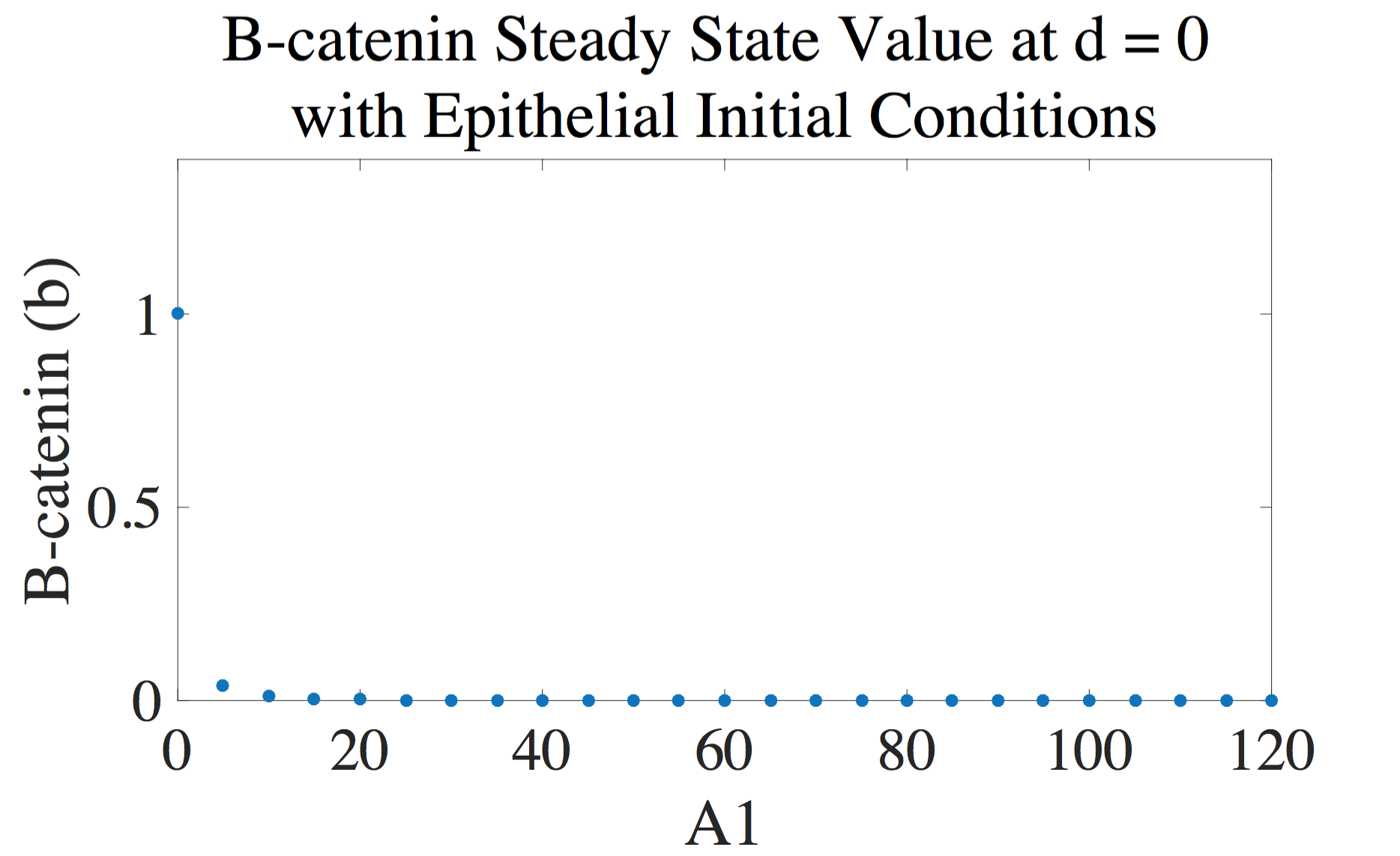 | 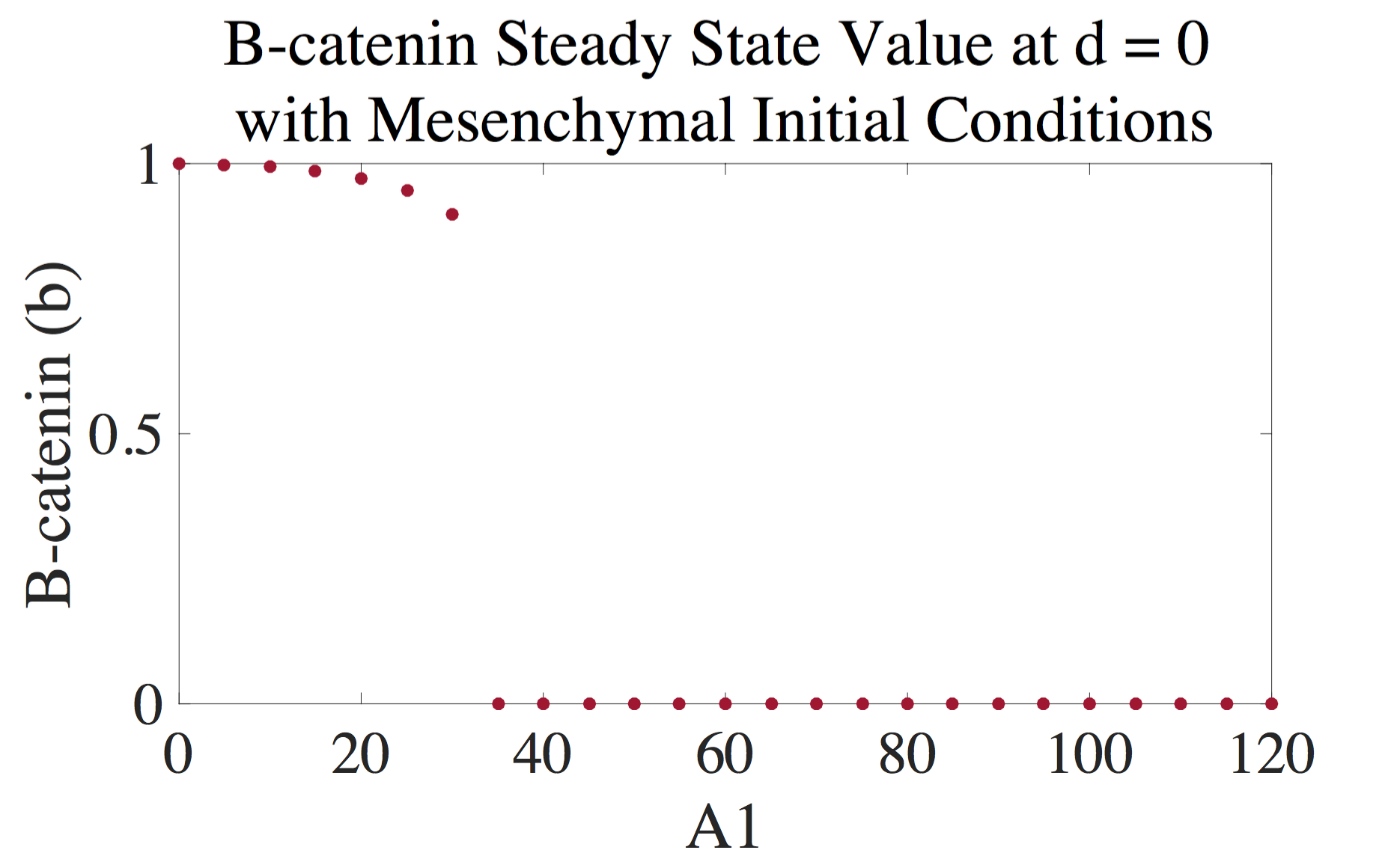 |
| Figure S1G | Figure S1H |
|  |  |
| 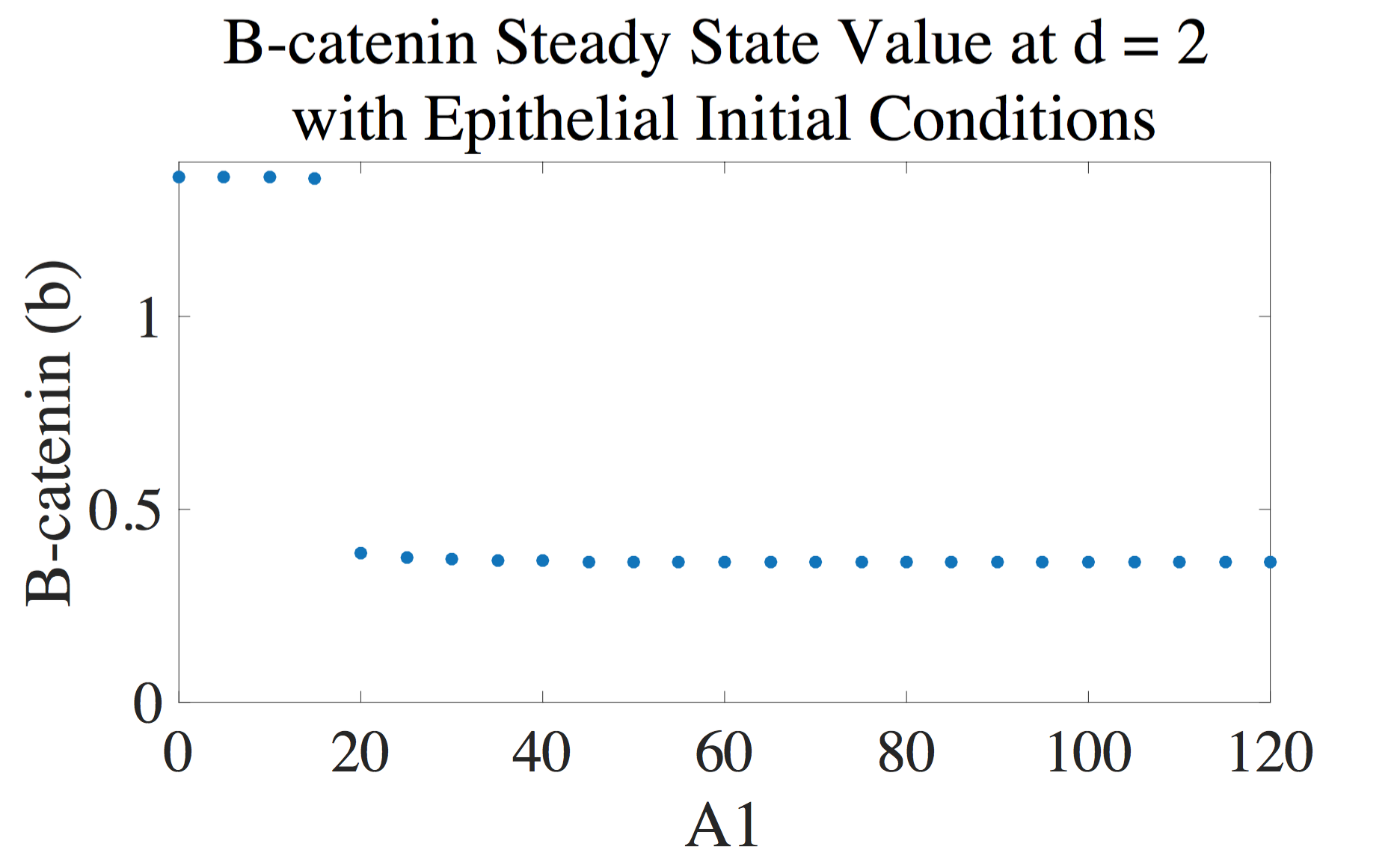 | 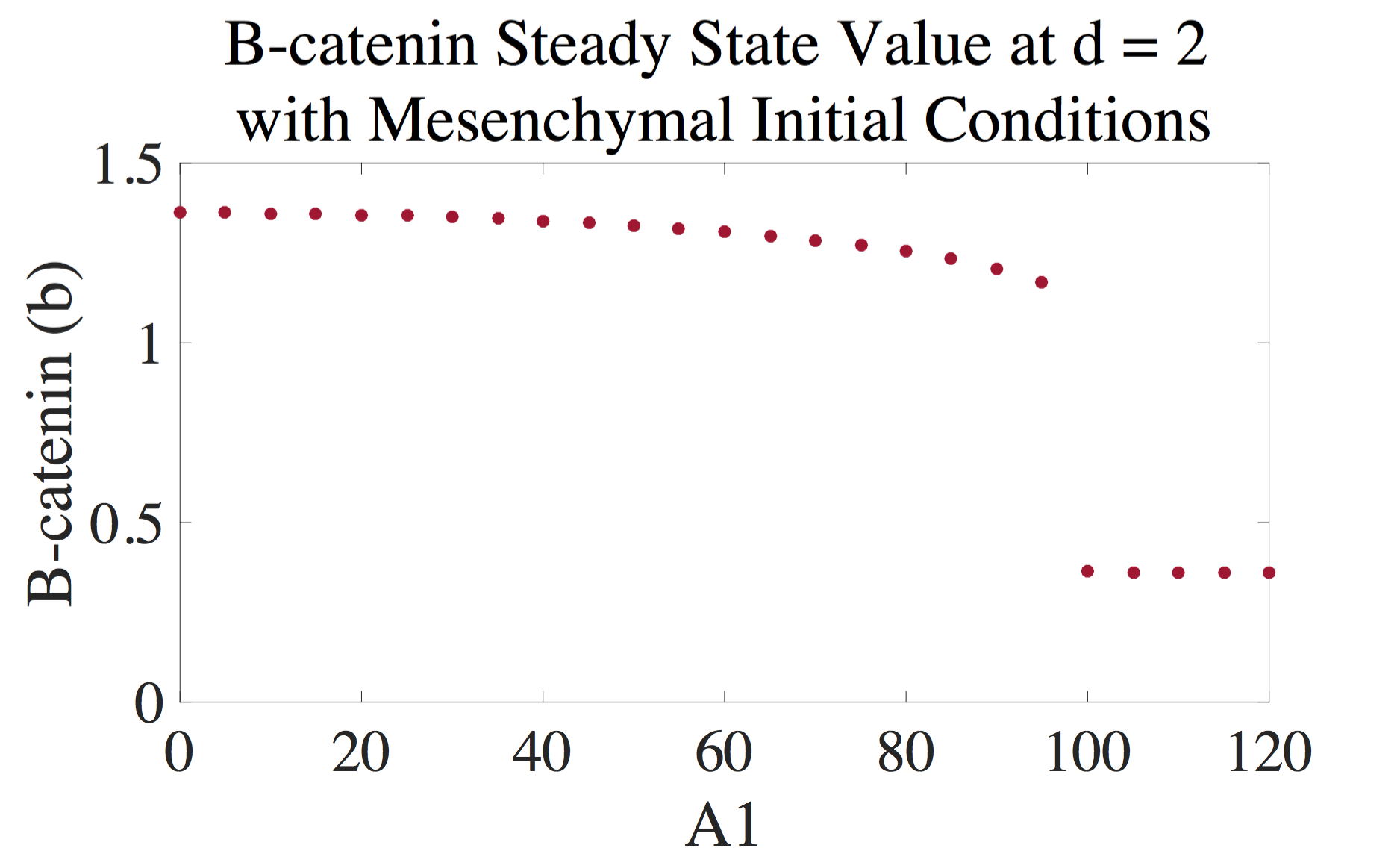 |
| Figure S1I | Figure S1J |
|  |  |
| 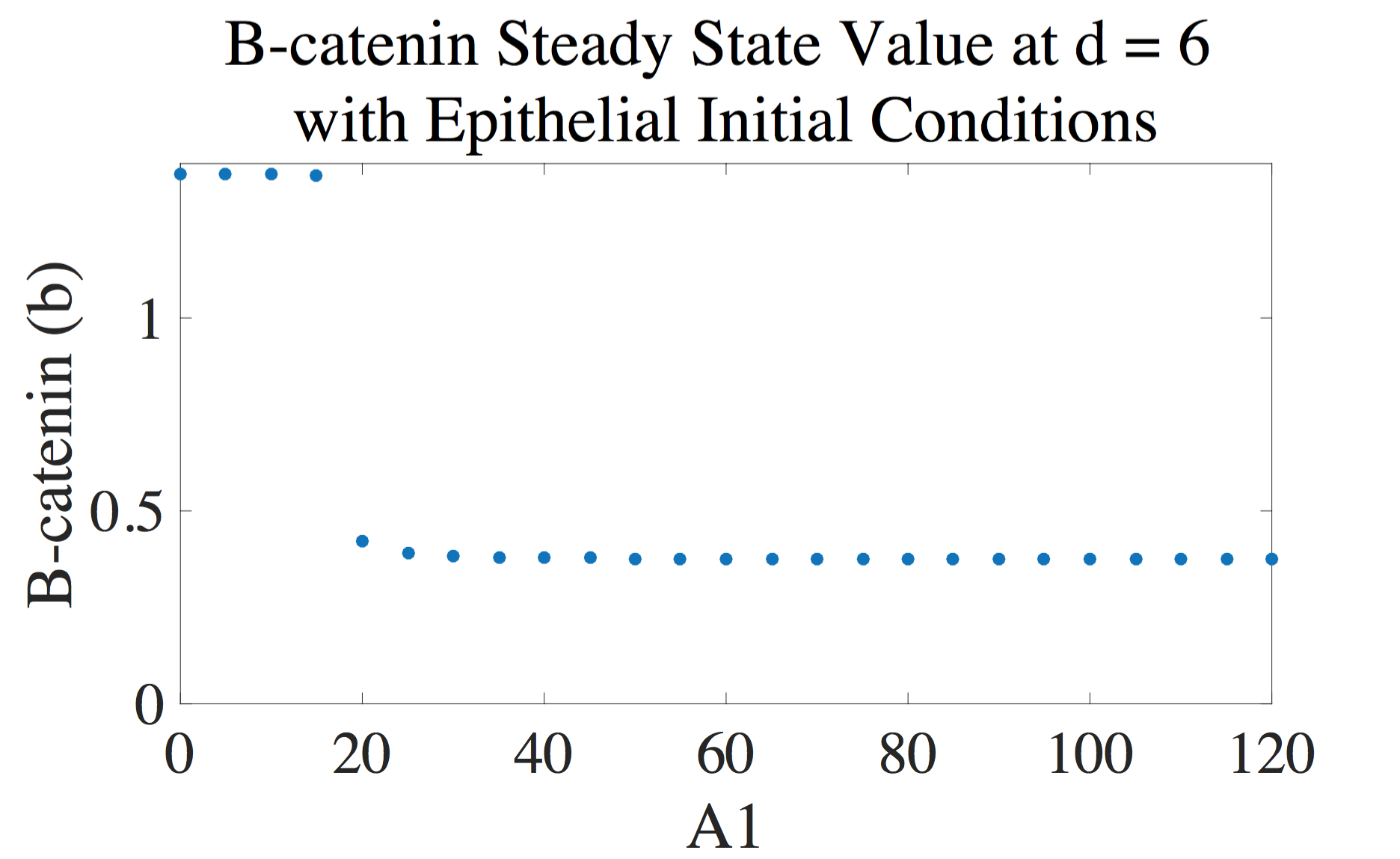 | 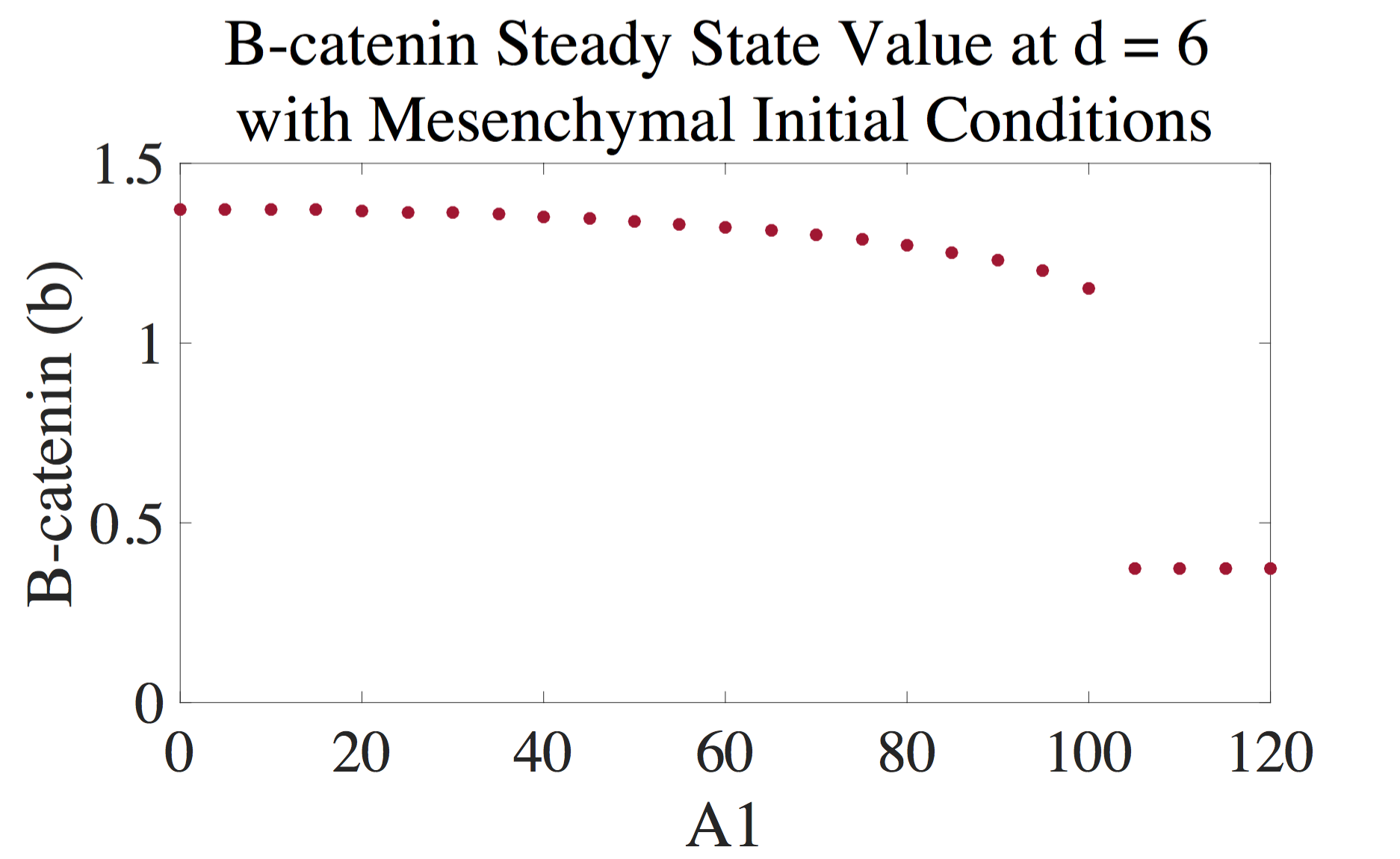 |
| Figure S1K | Figure S1L |
|  |  |
| 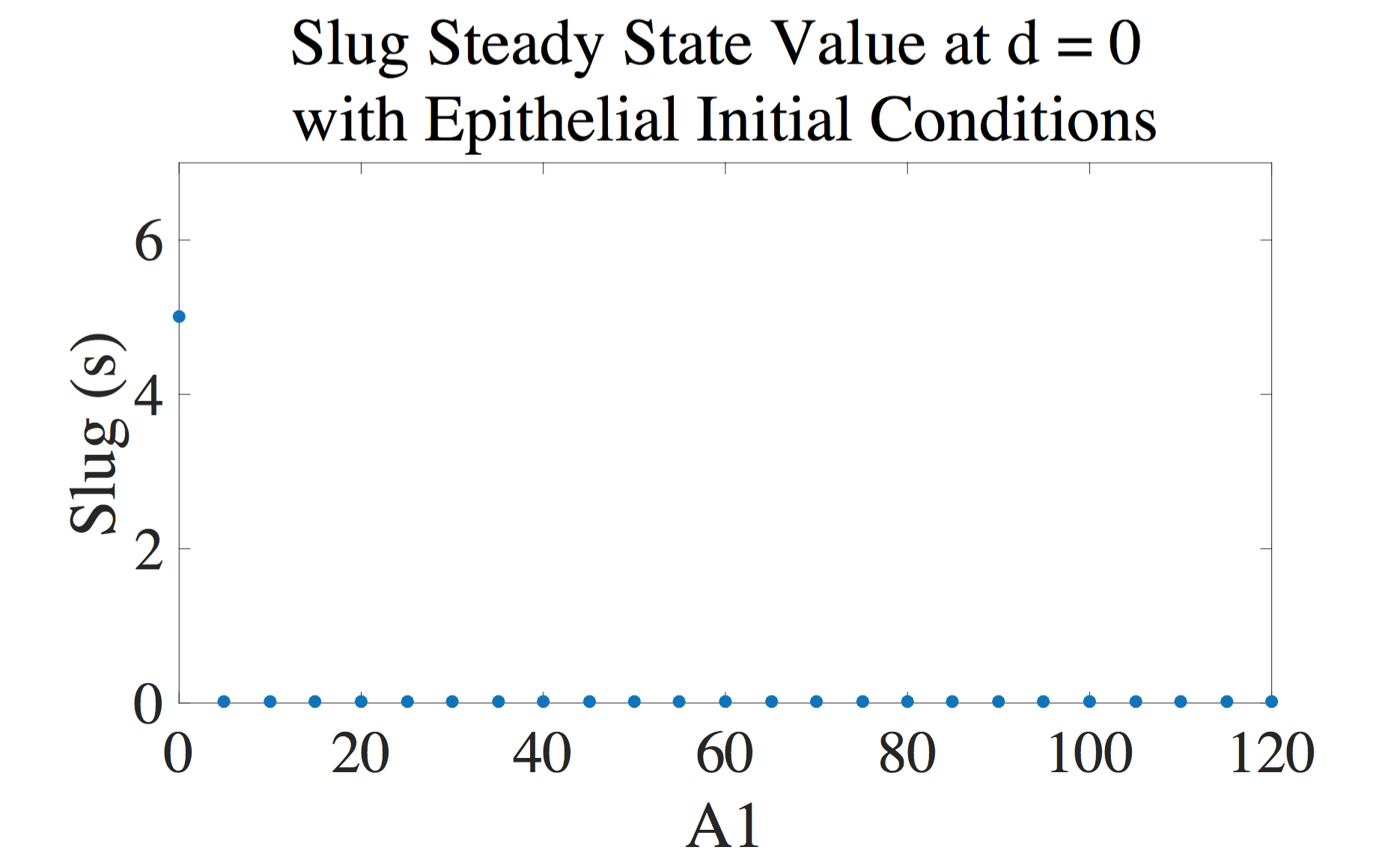 | 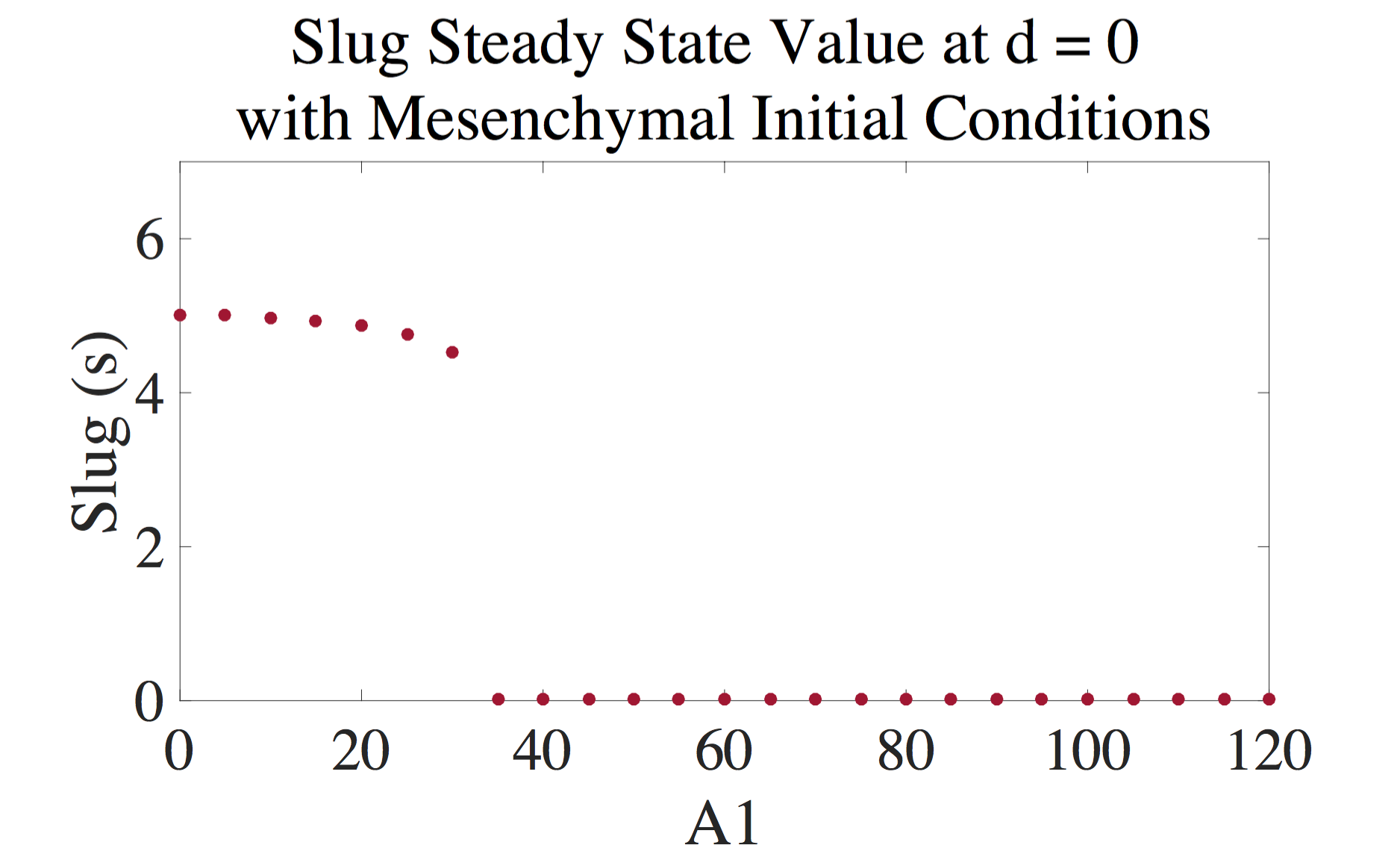 |
| Figure S1M | Figure S1N |
|  |  |
| 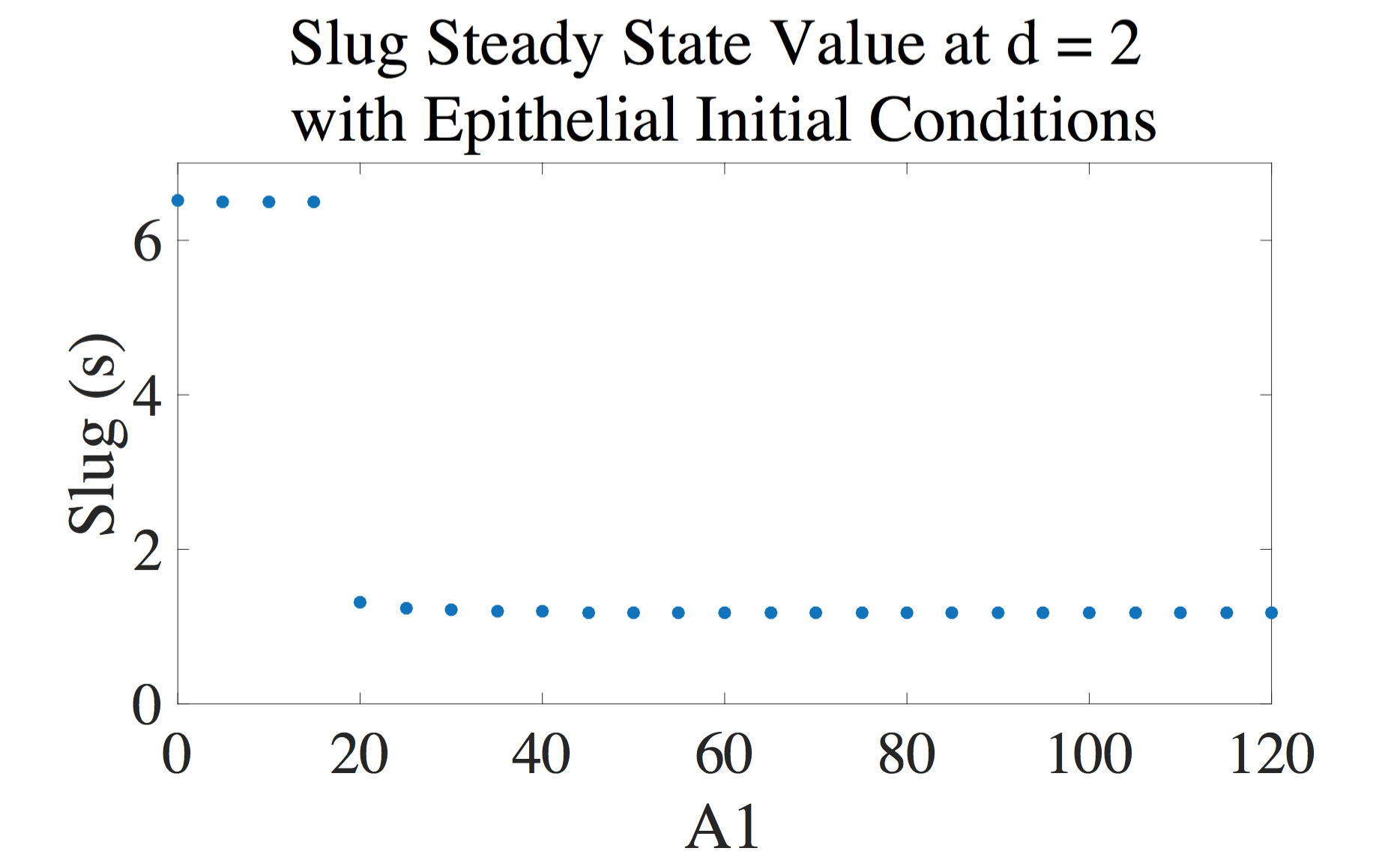 | 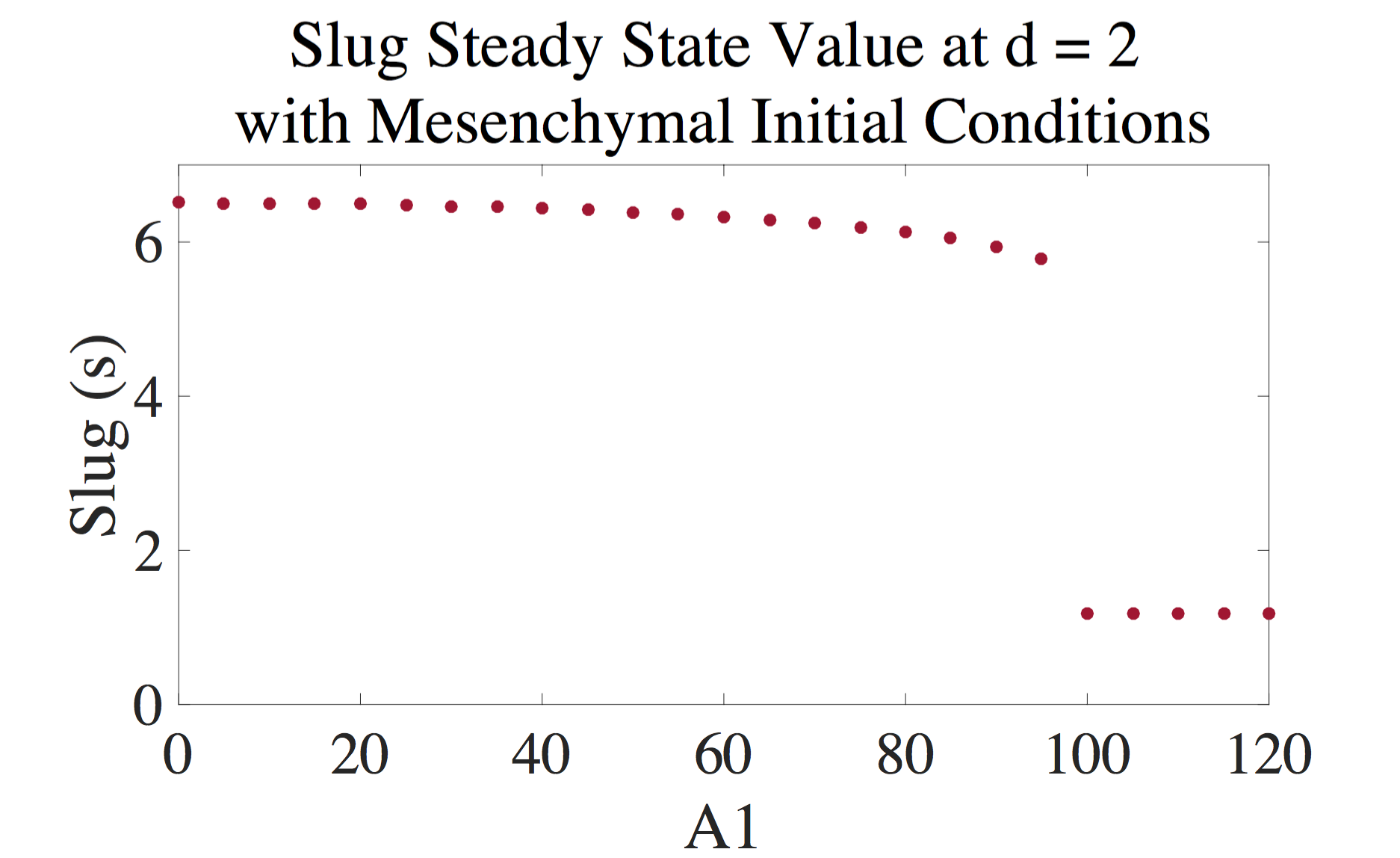 |
| Figure S1O | Figure S1P |
|  |  |
| 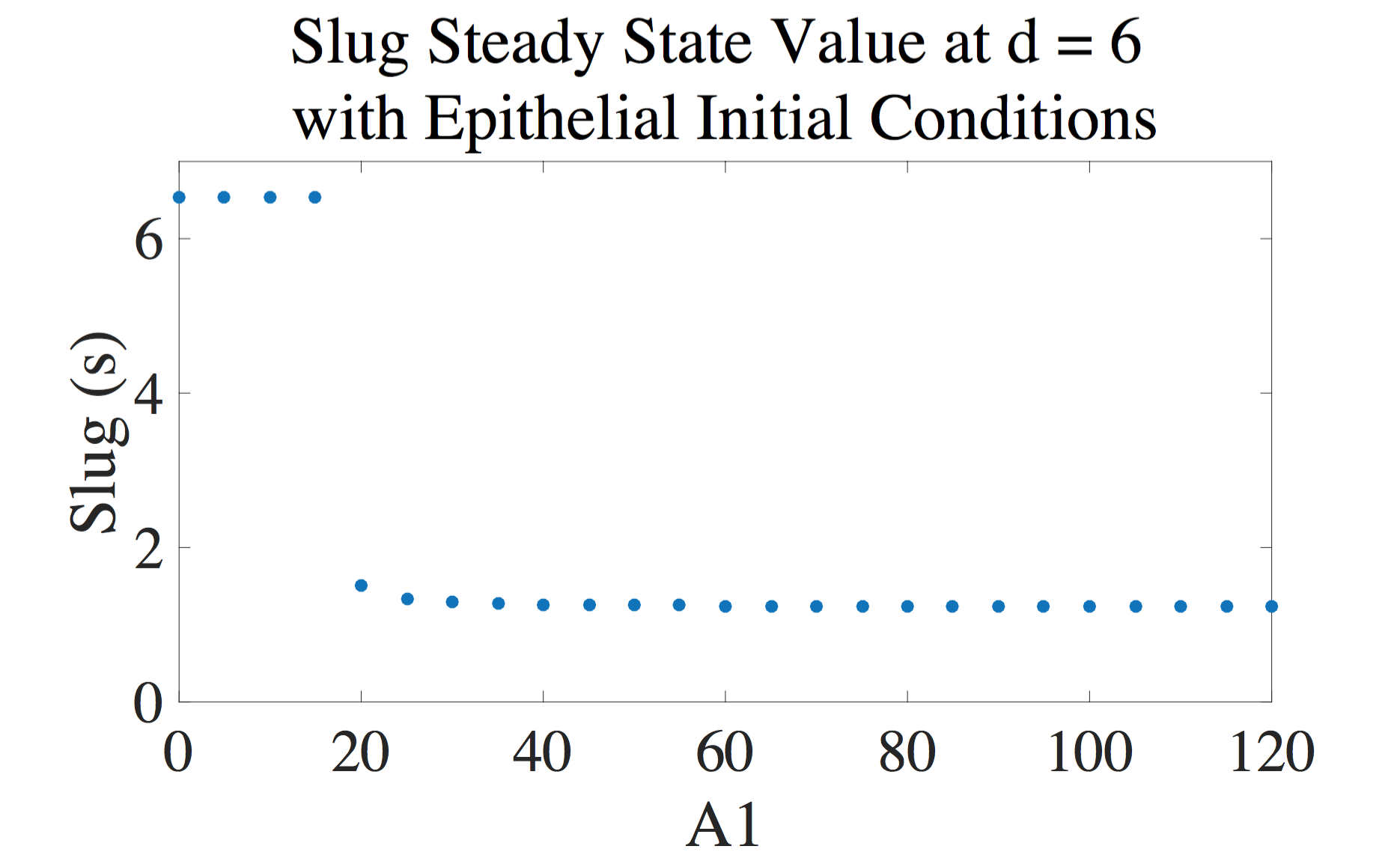 | 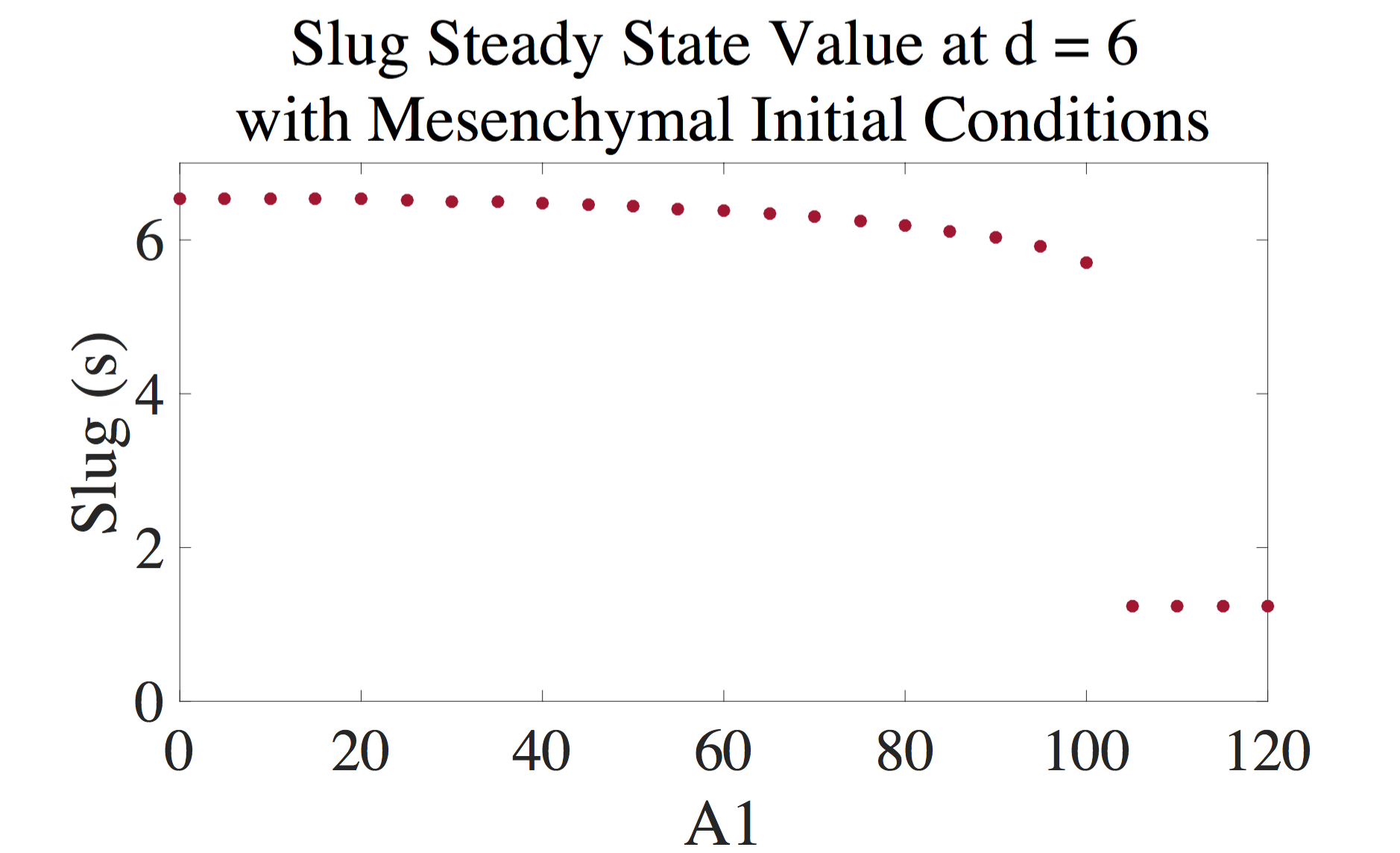 |
| Figure S1Q | Figure S1R |
|  |  |
| 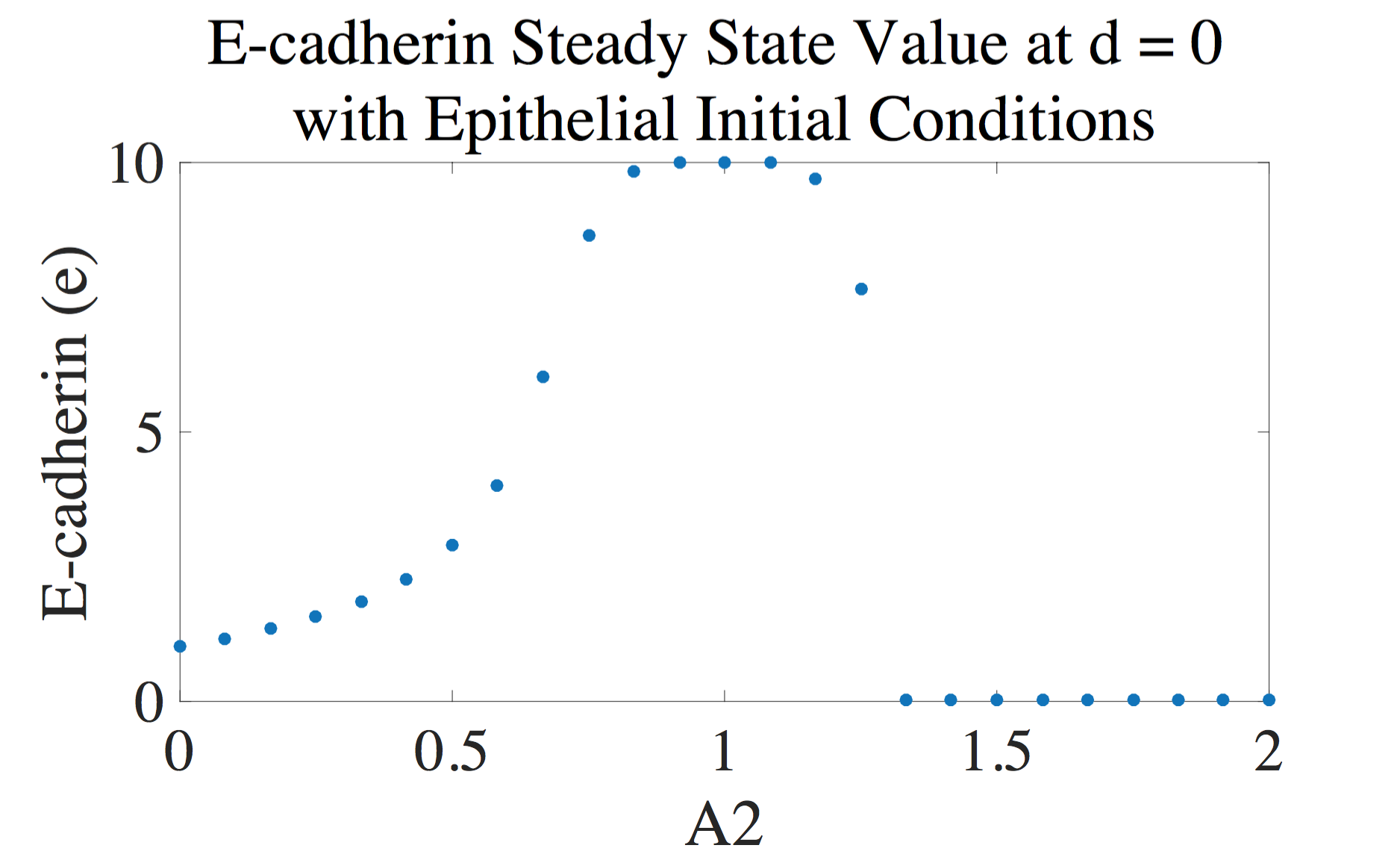 | 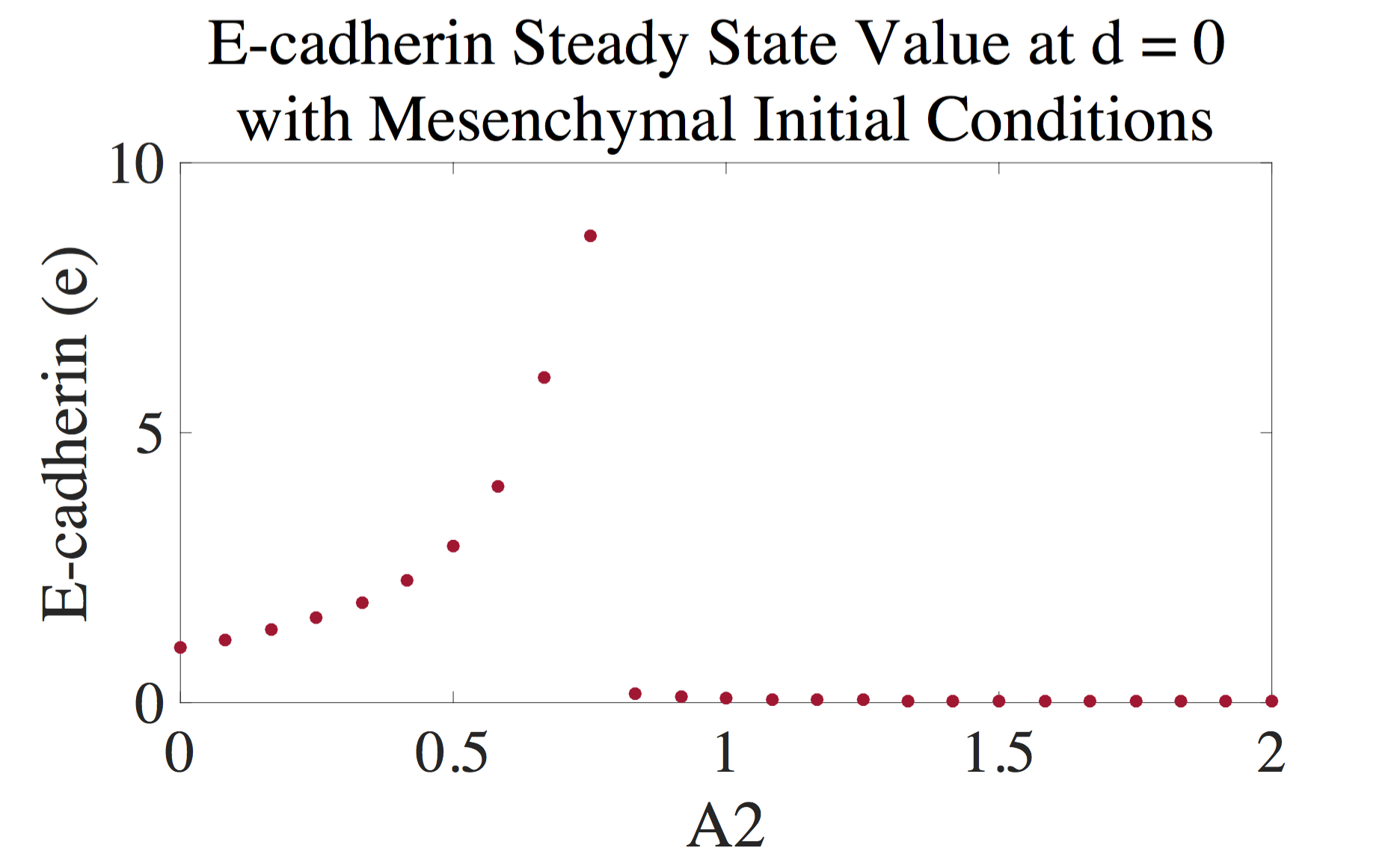 |
| Figure S1S | Figure S1T |
|  |  |
| 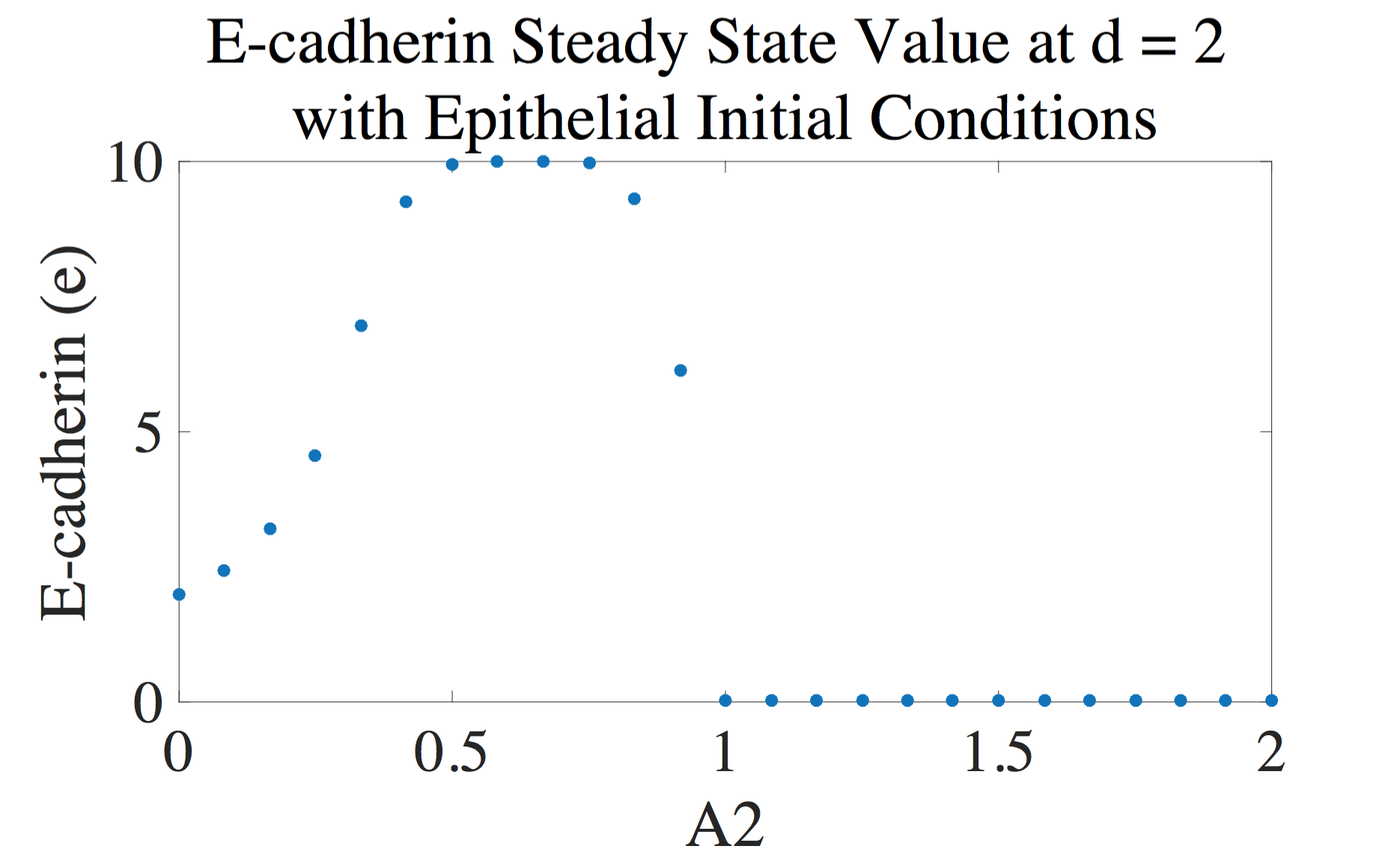 | 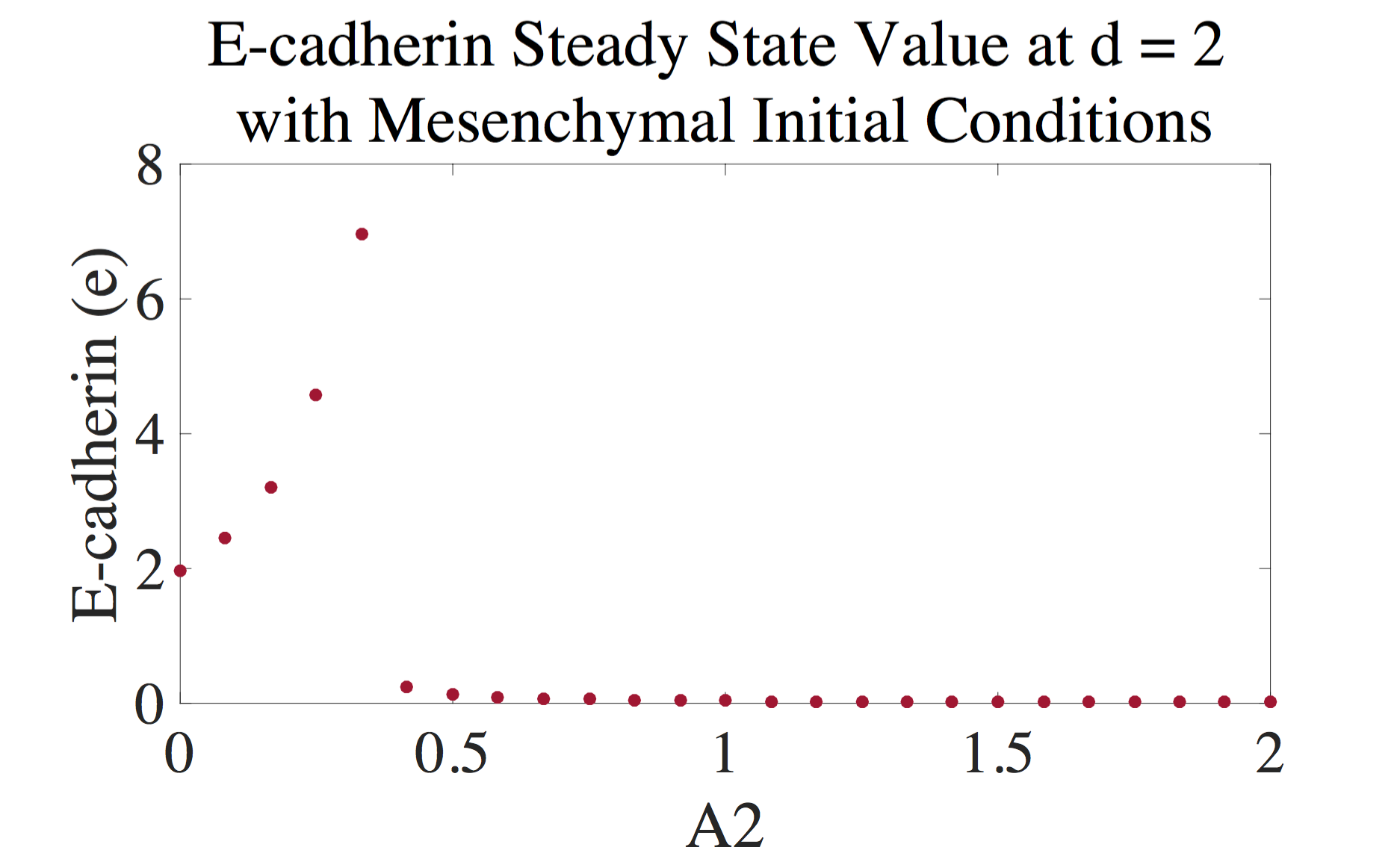 |
| Figure S1U | Figure S1V |
|  |  |
| 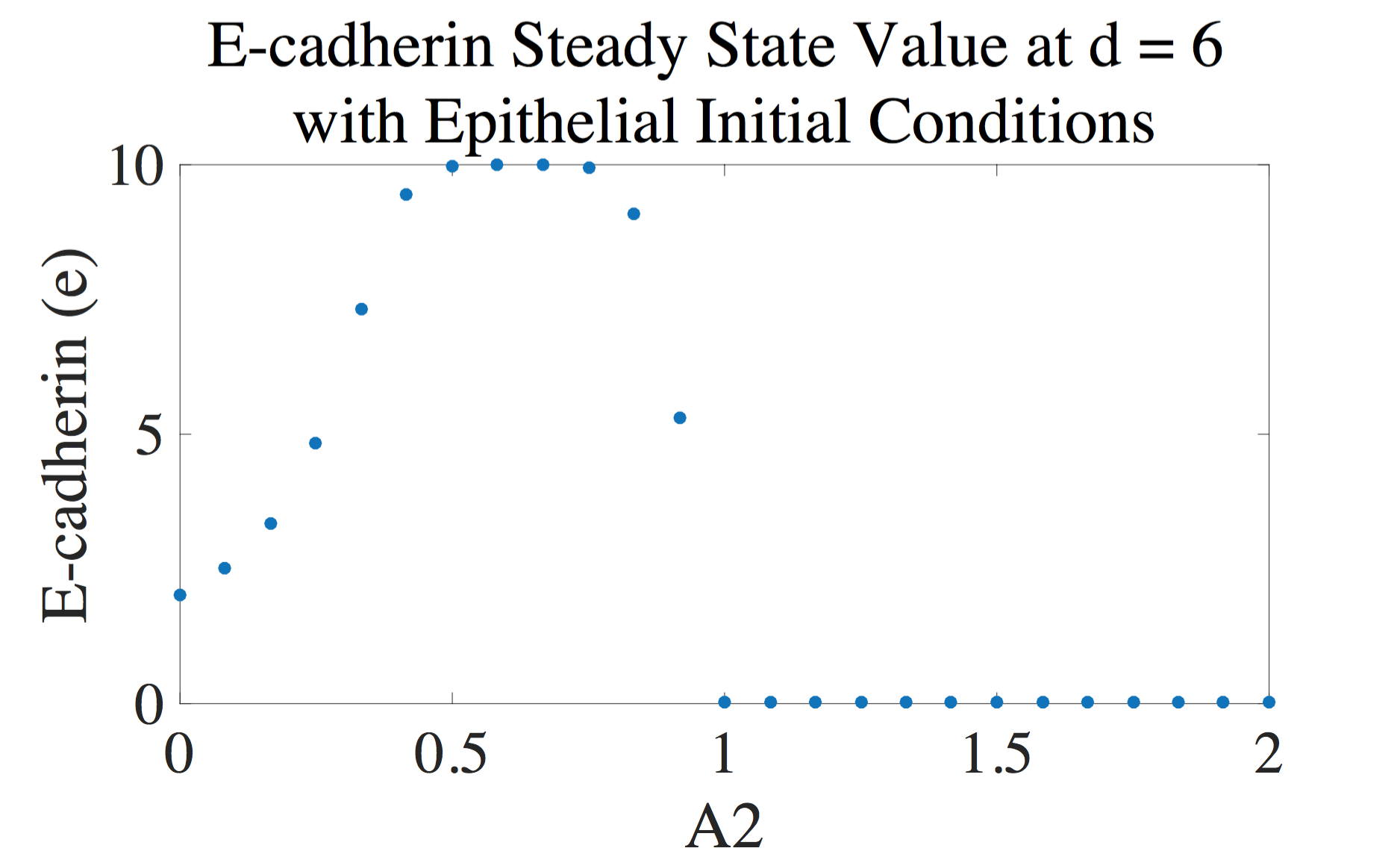 | 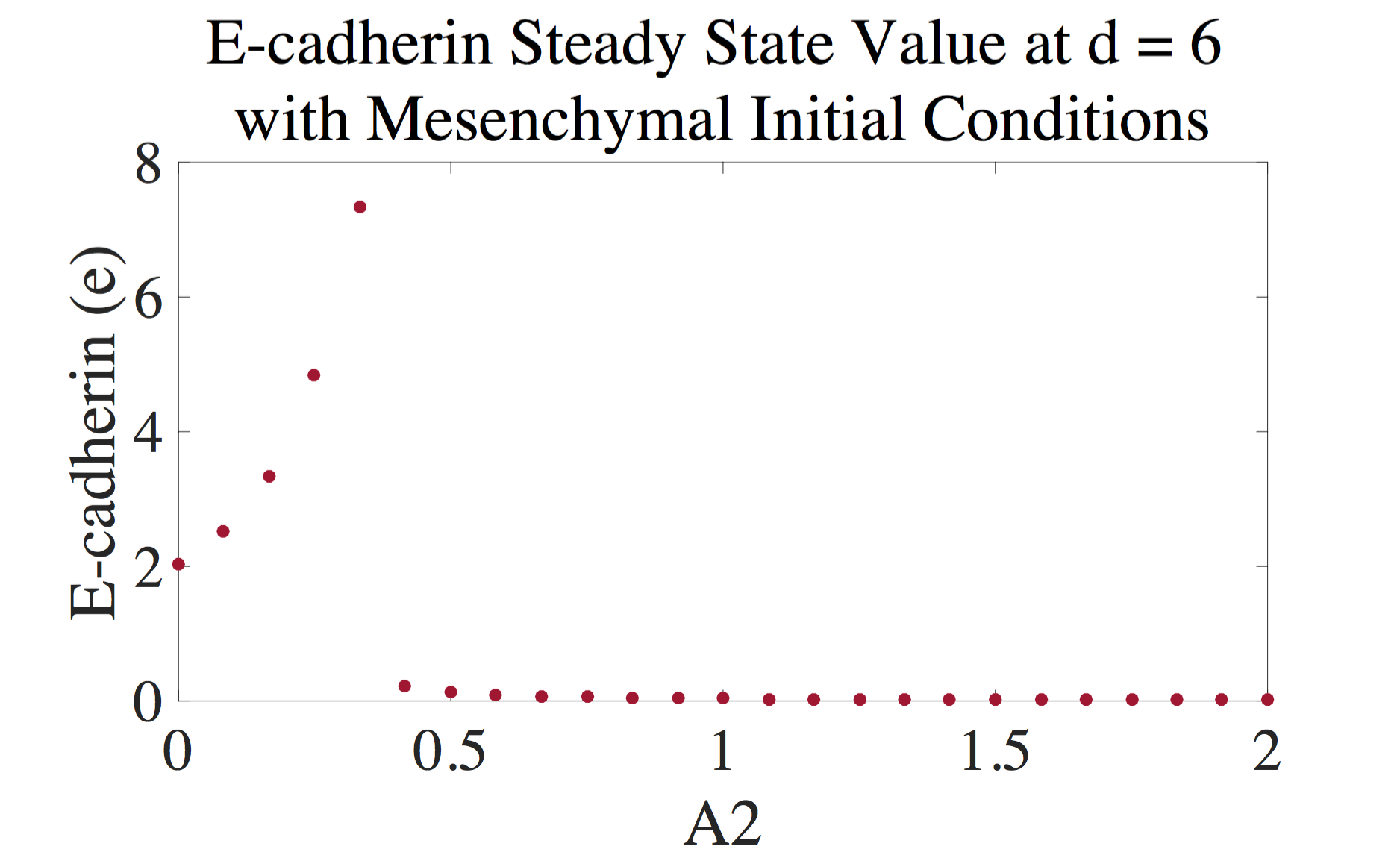 |
| Figure S1W | Figure S1X |
|  |  |
| 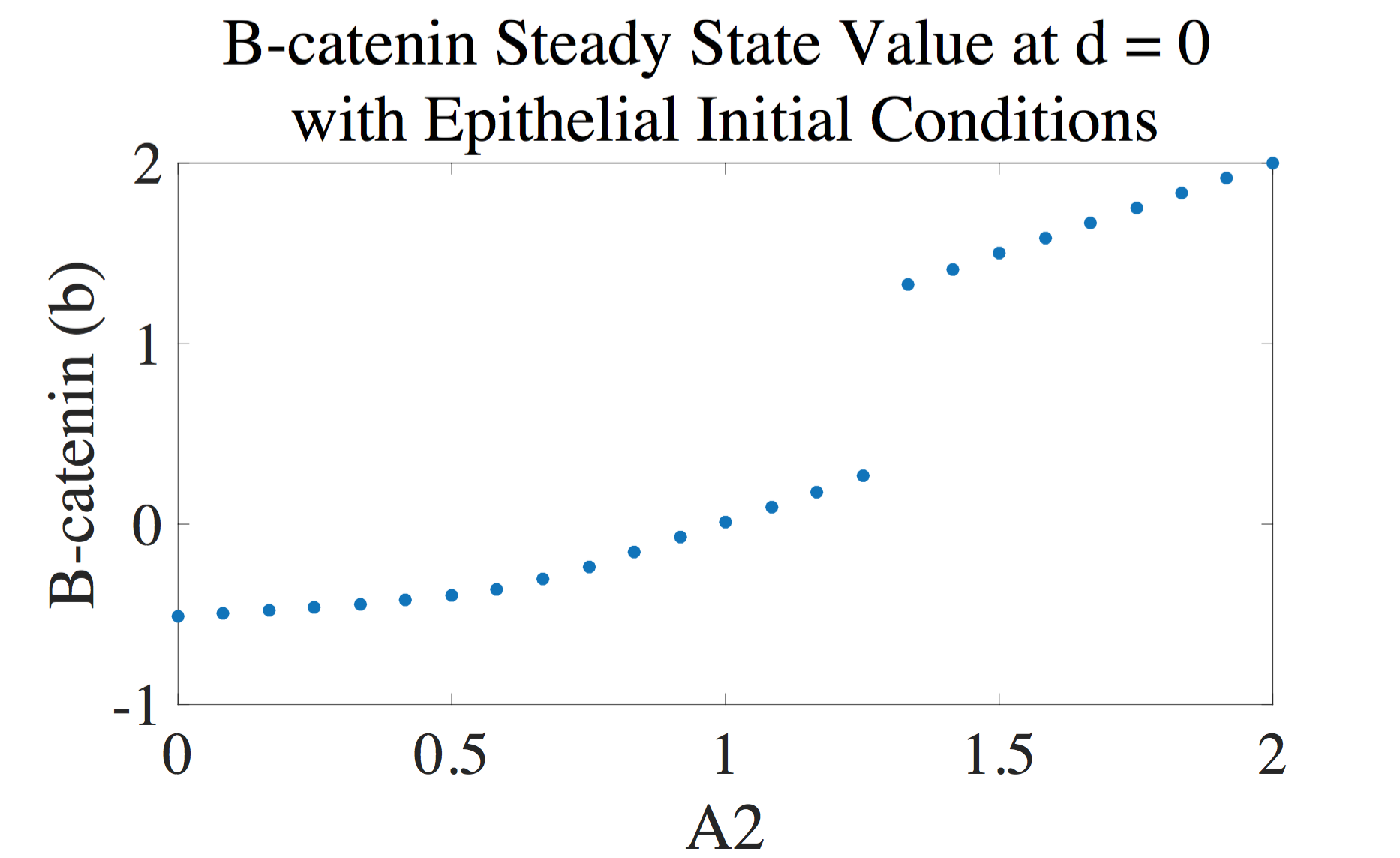 | 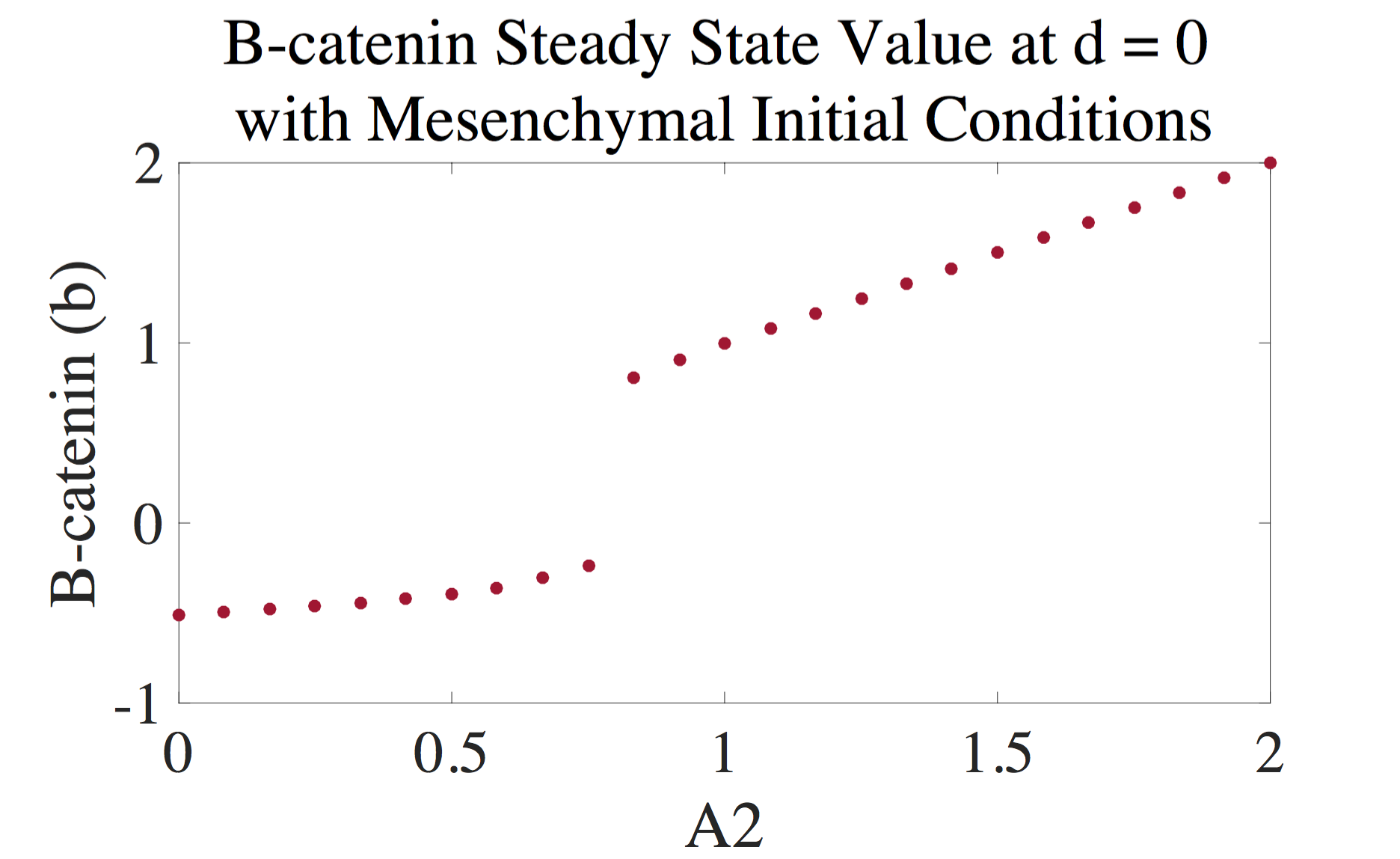 |
| Figure S1Y | Figure S1Z |
|  |  |
| 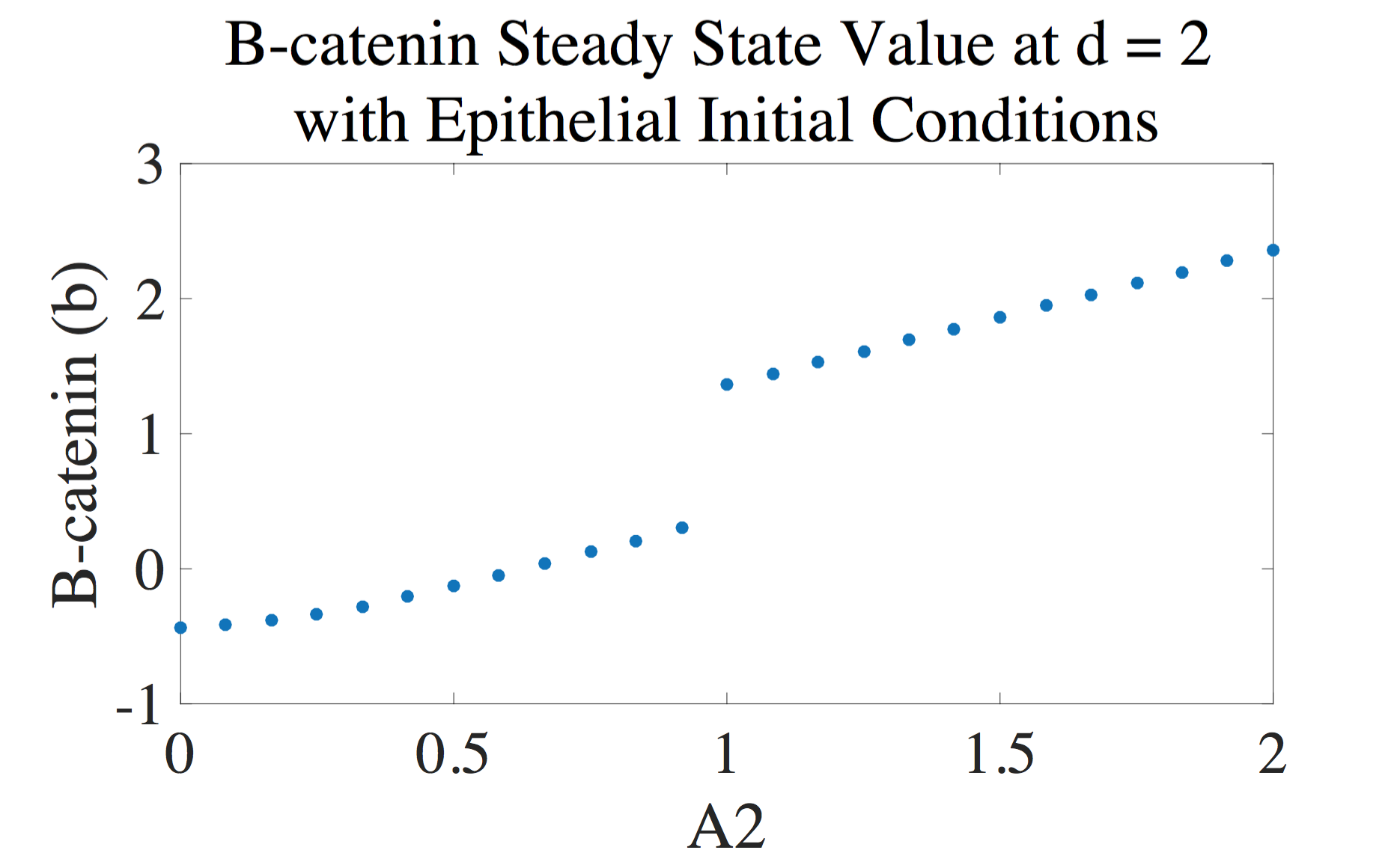 | 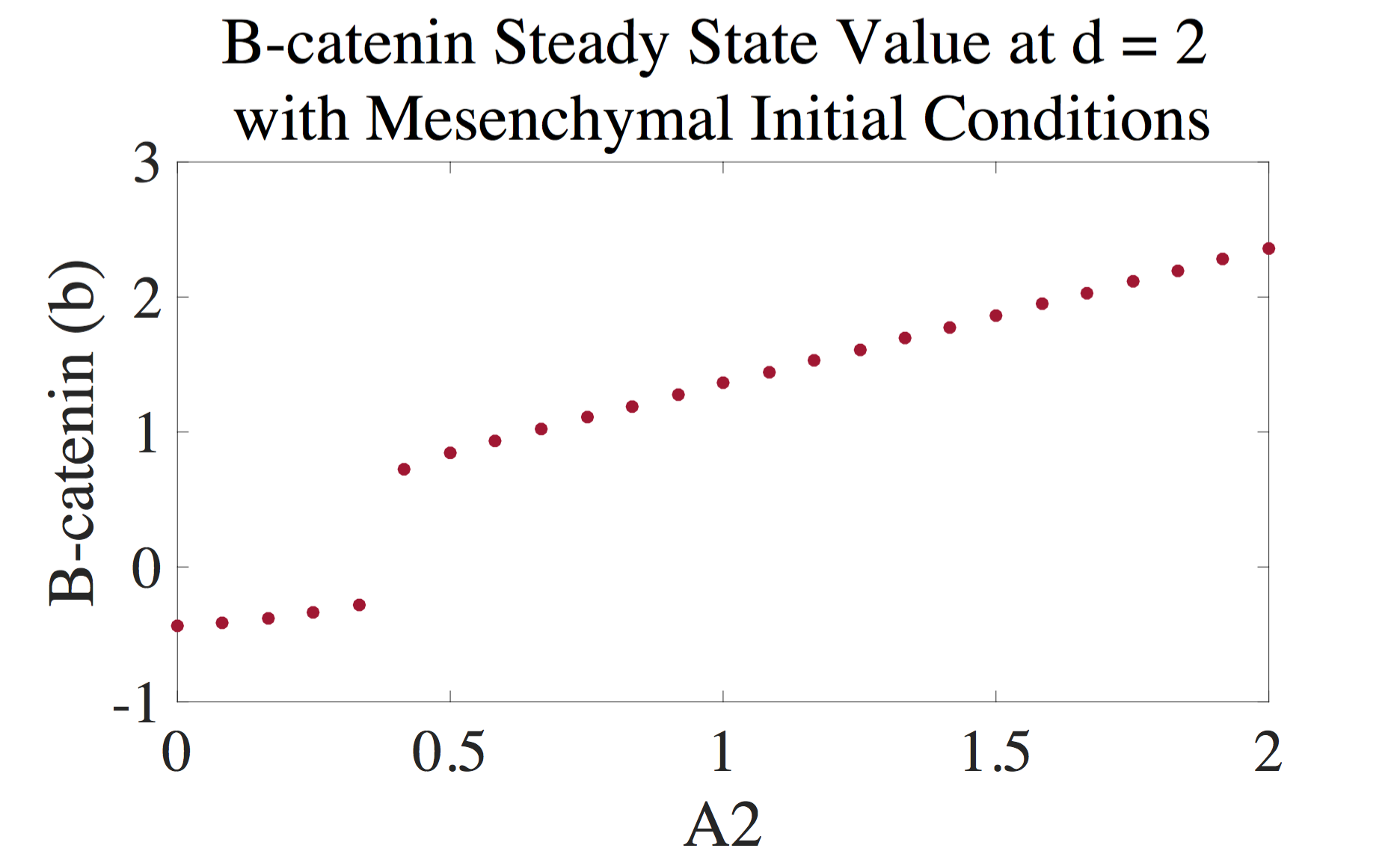 |
| Figure S1AA | Figure S1AB |
|  |  |
| 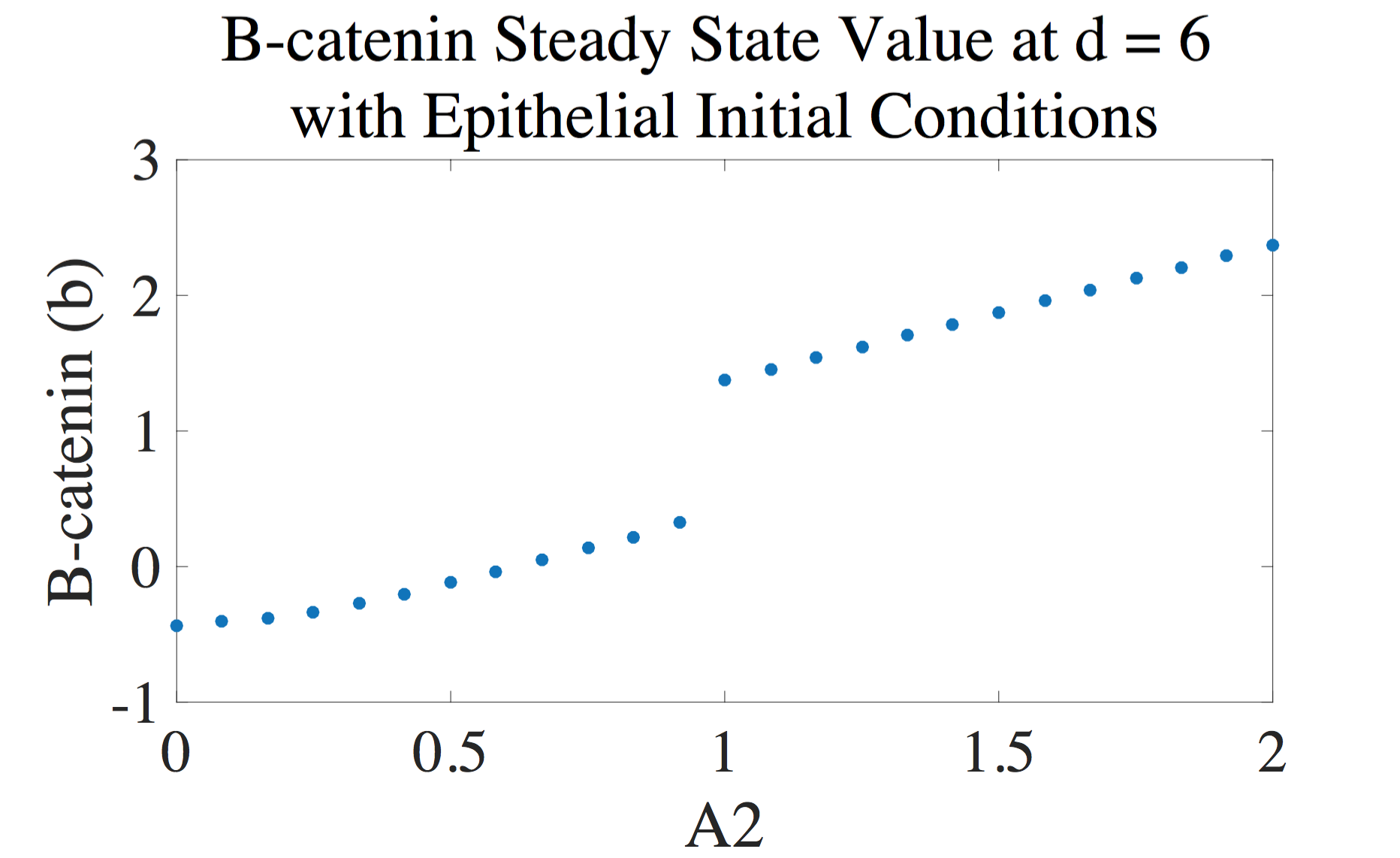 | 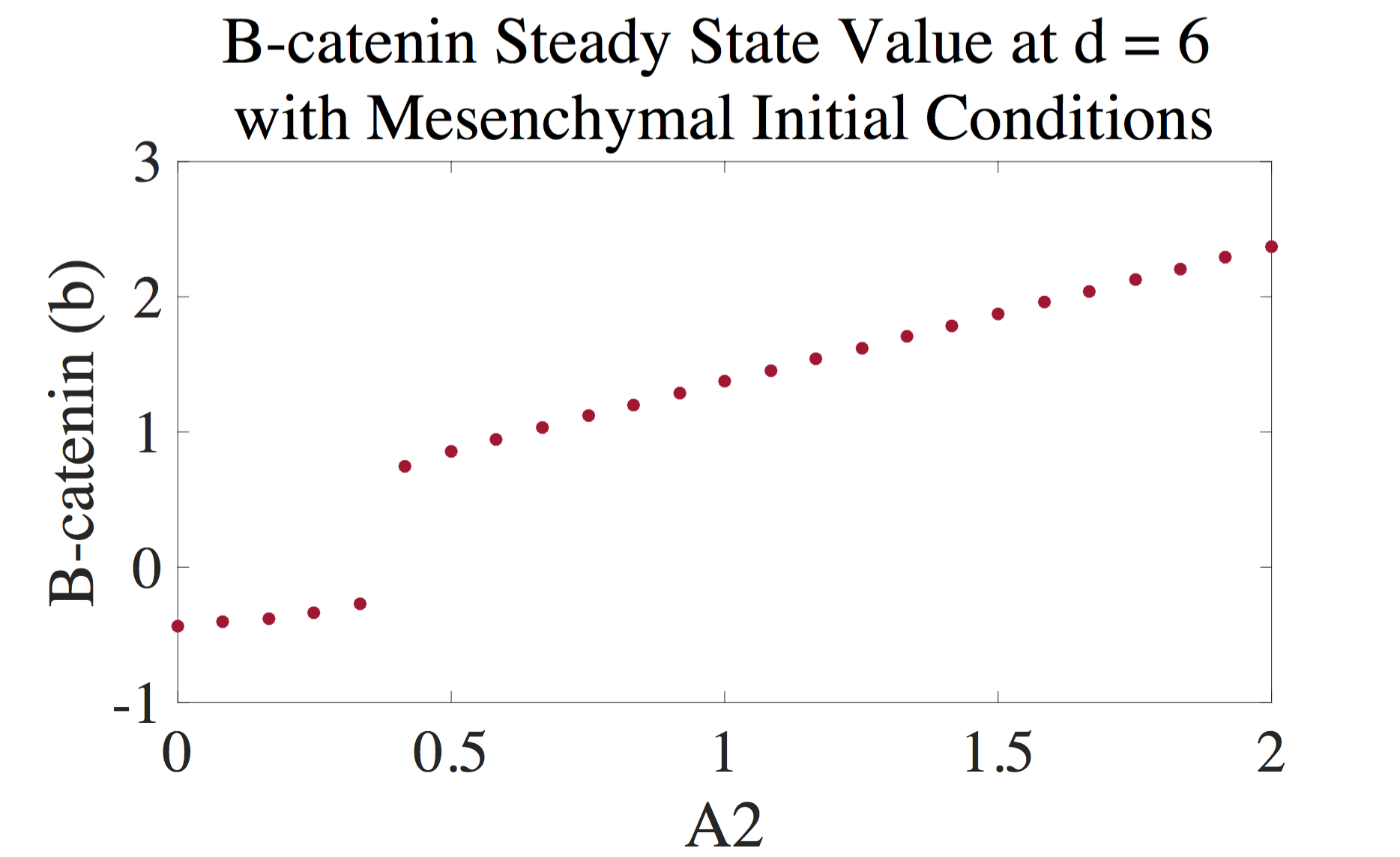 |
| Figure S1AC | Figure S1AD |
|  |  |
| 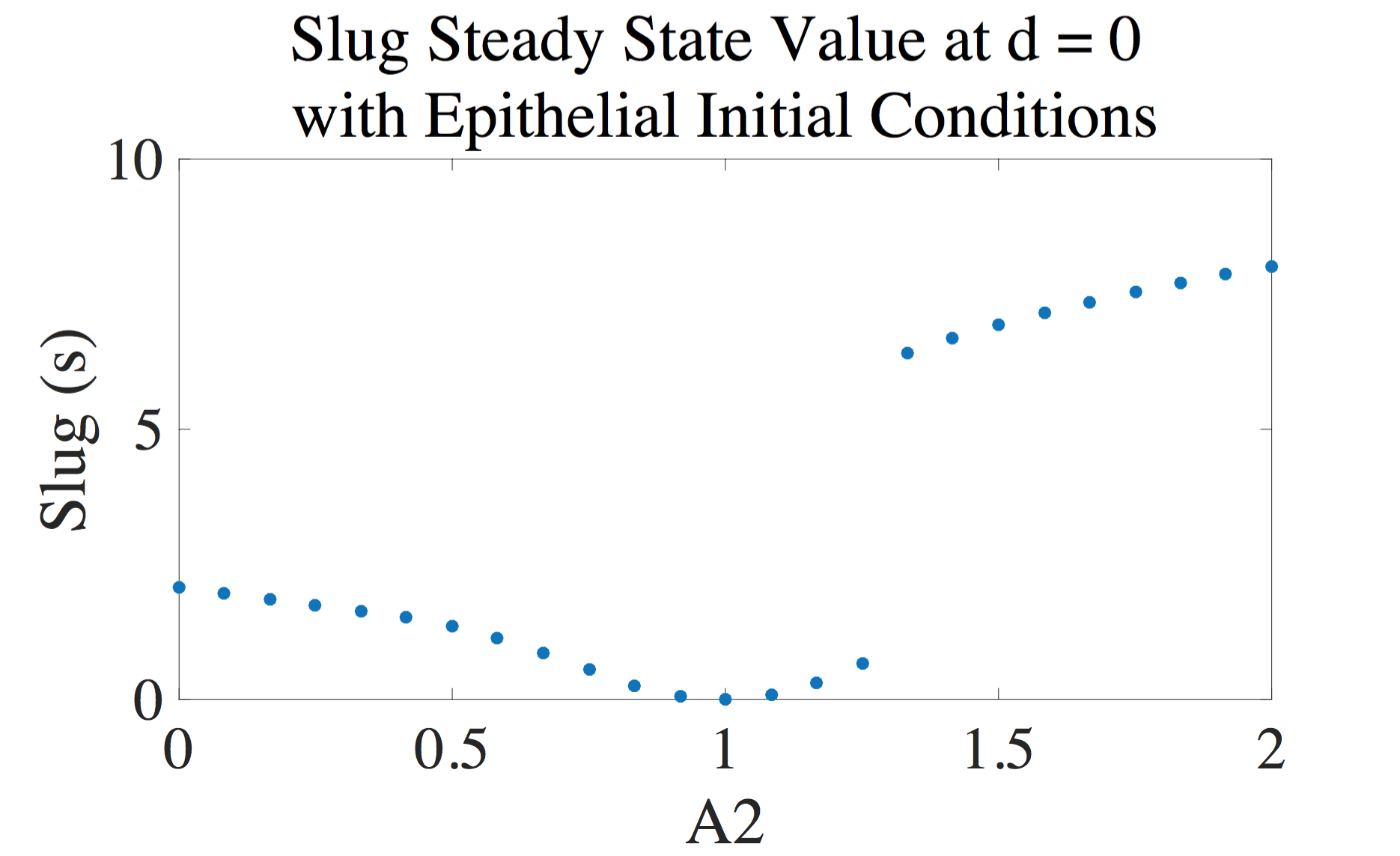 | 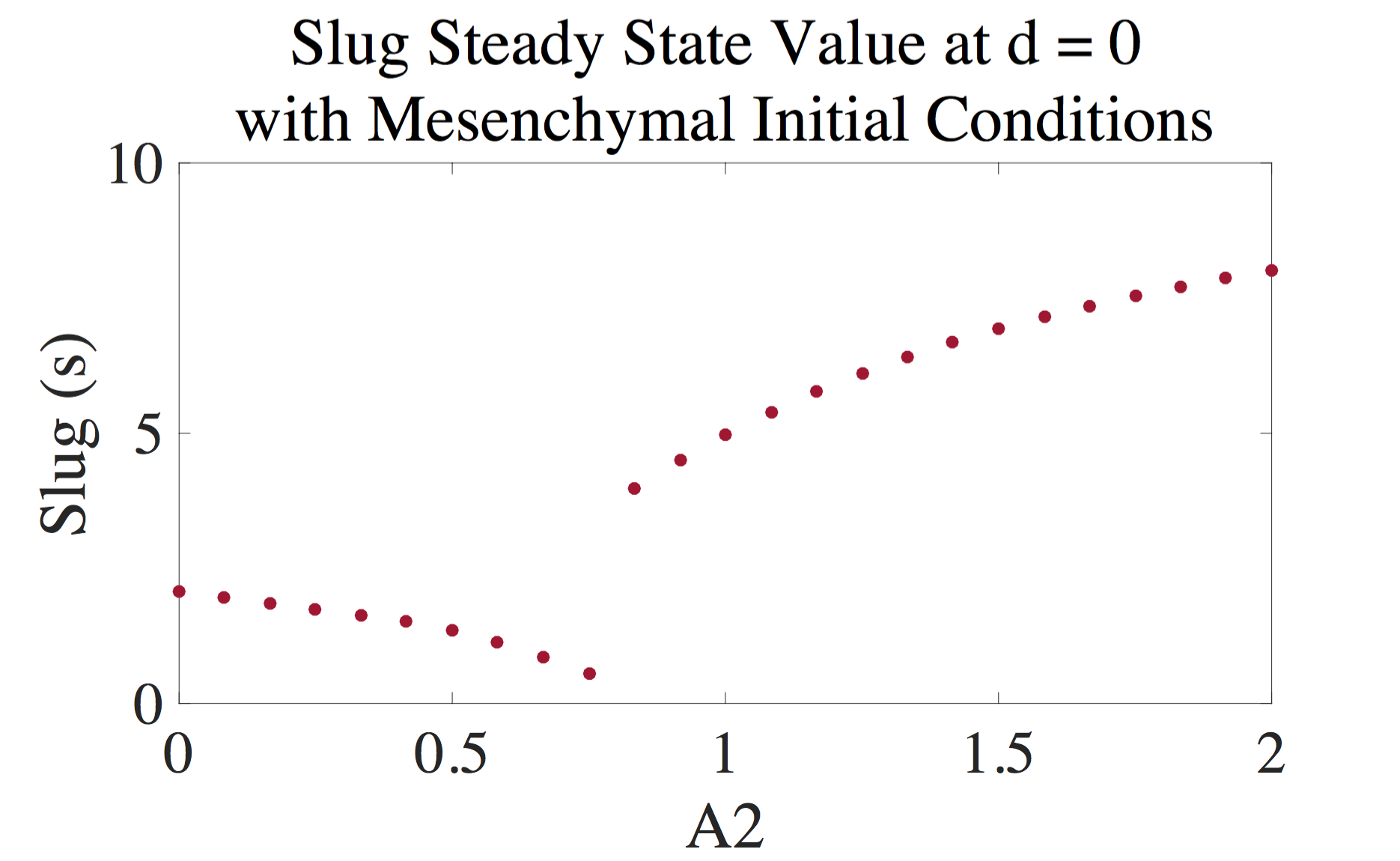 |
| Figure S1AE | Figure S1AF |
|  |  |
| 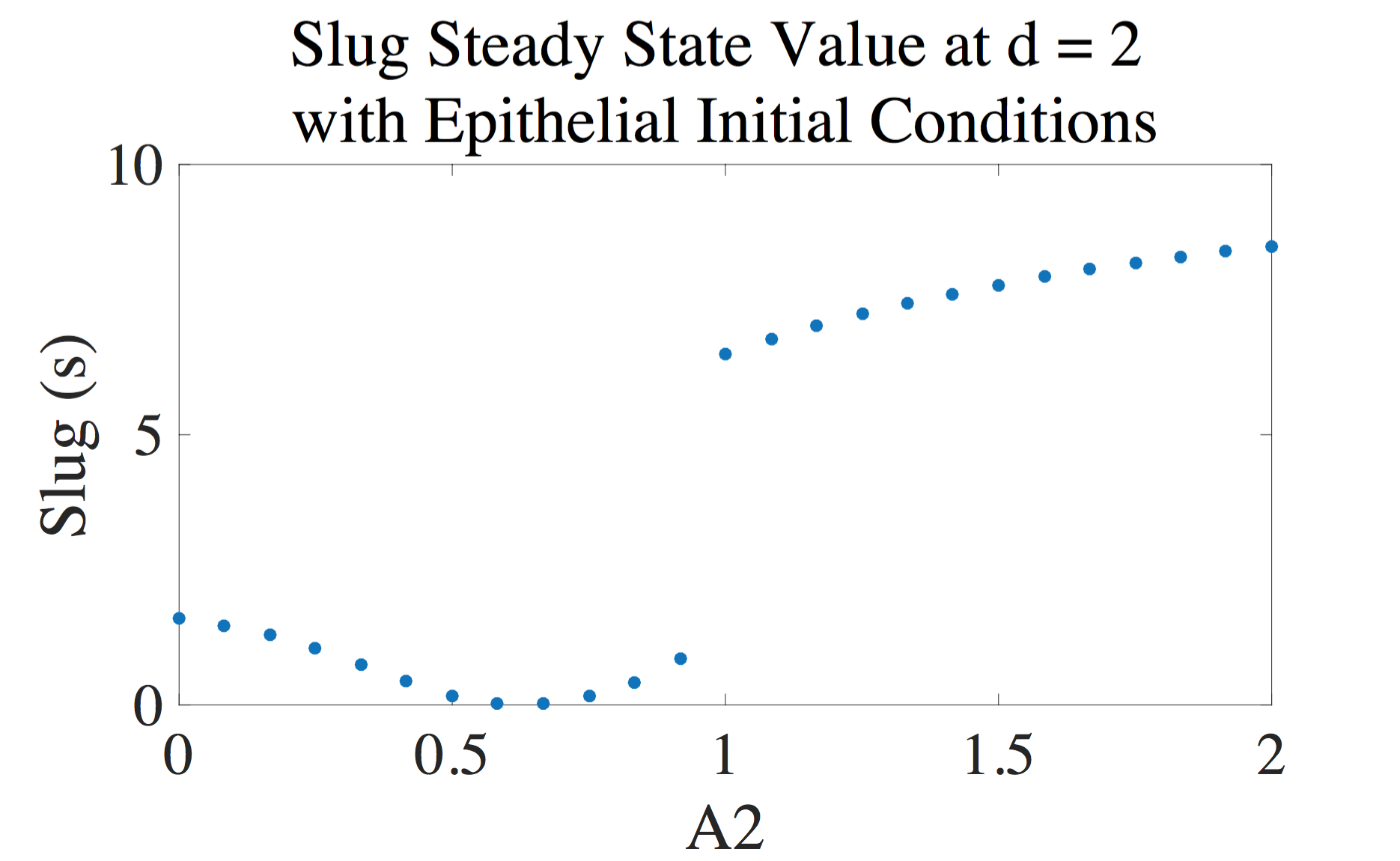 | 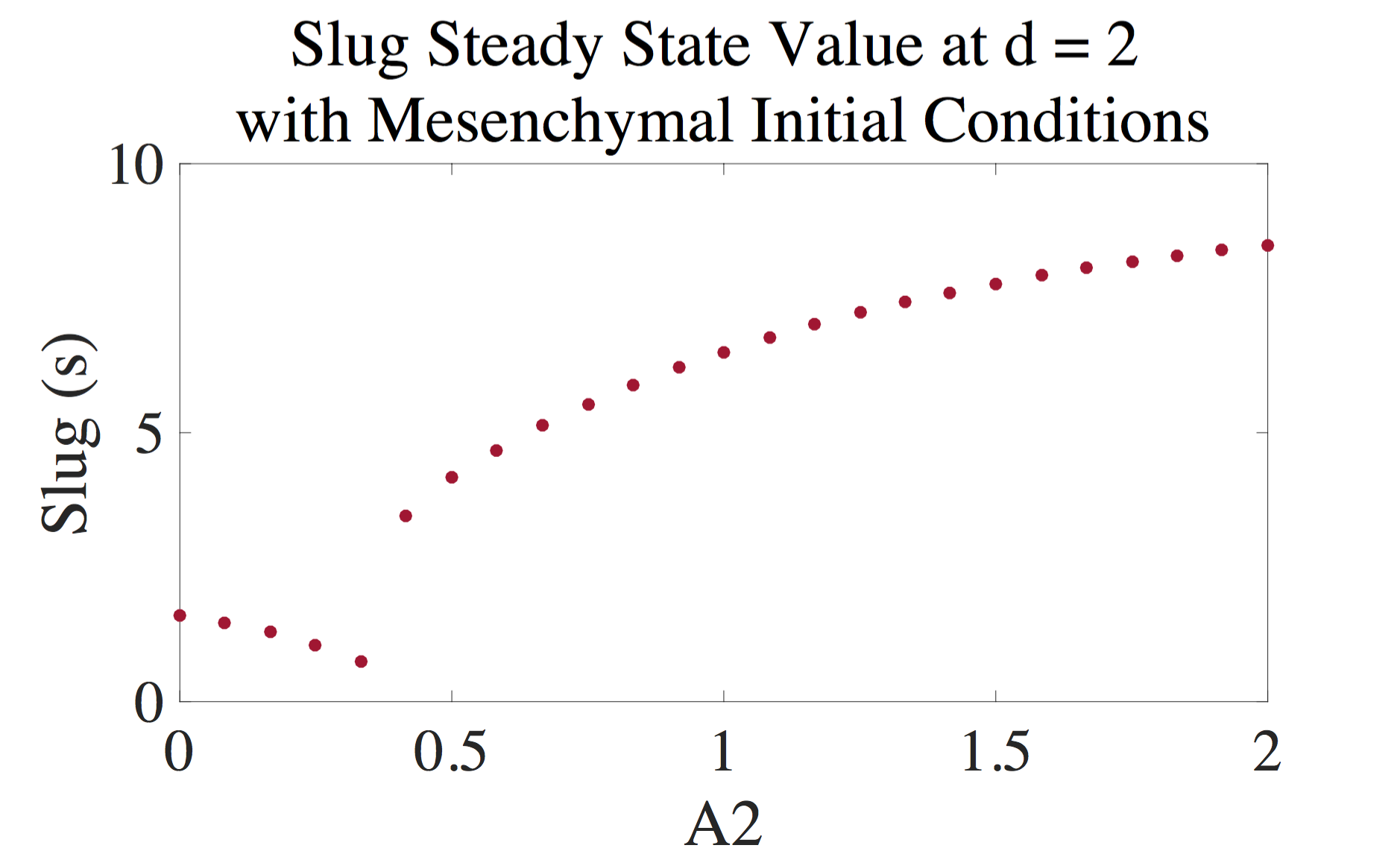 |
| Figure S1AG | Figure S1AH |
|  |  |
| 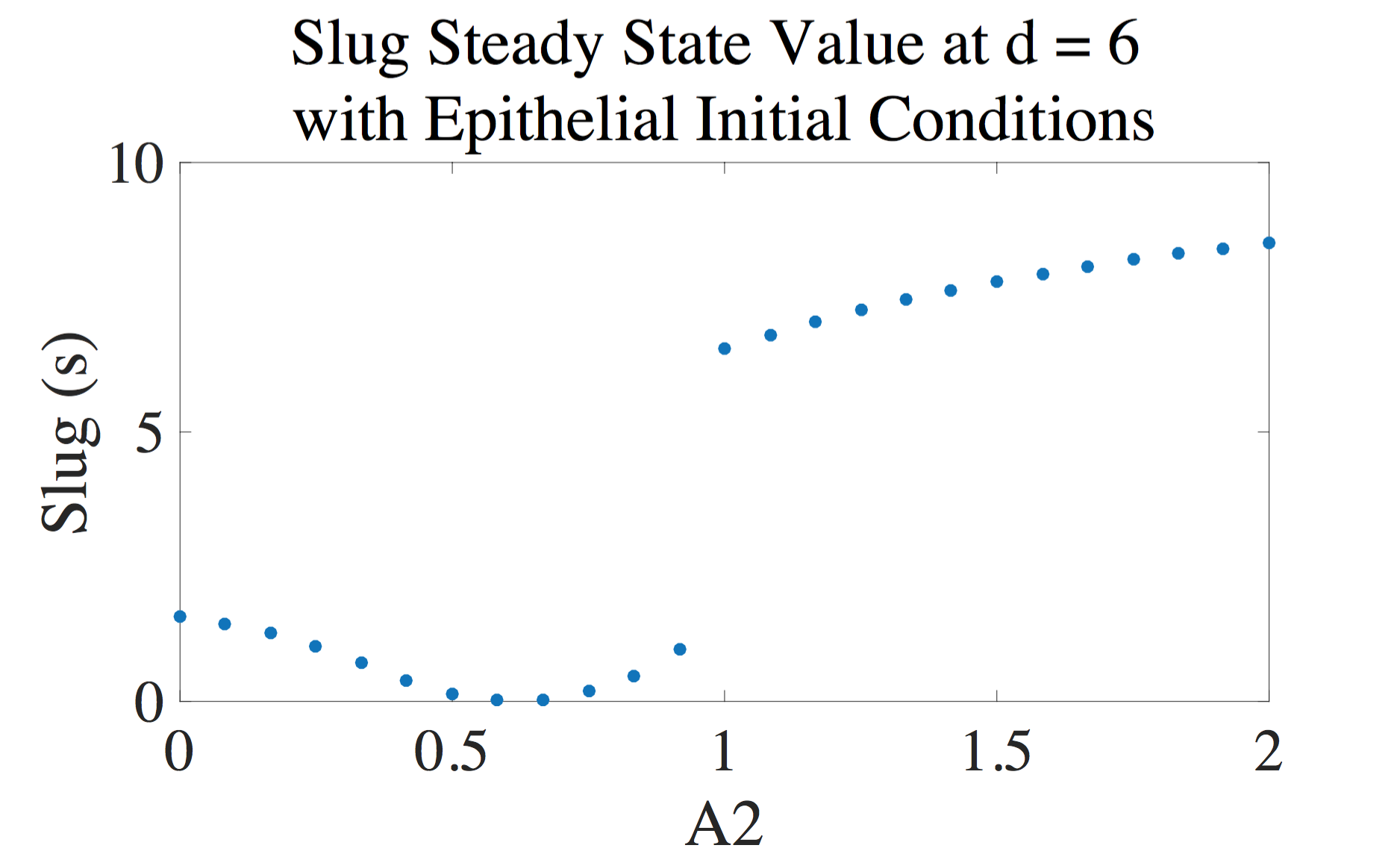 | 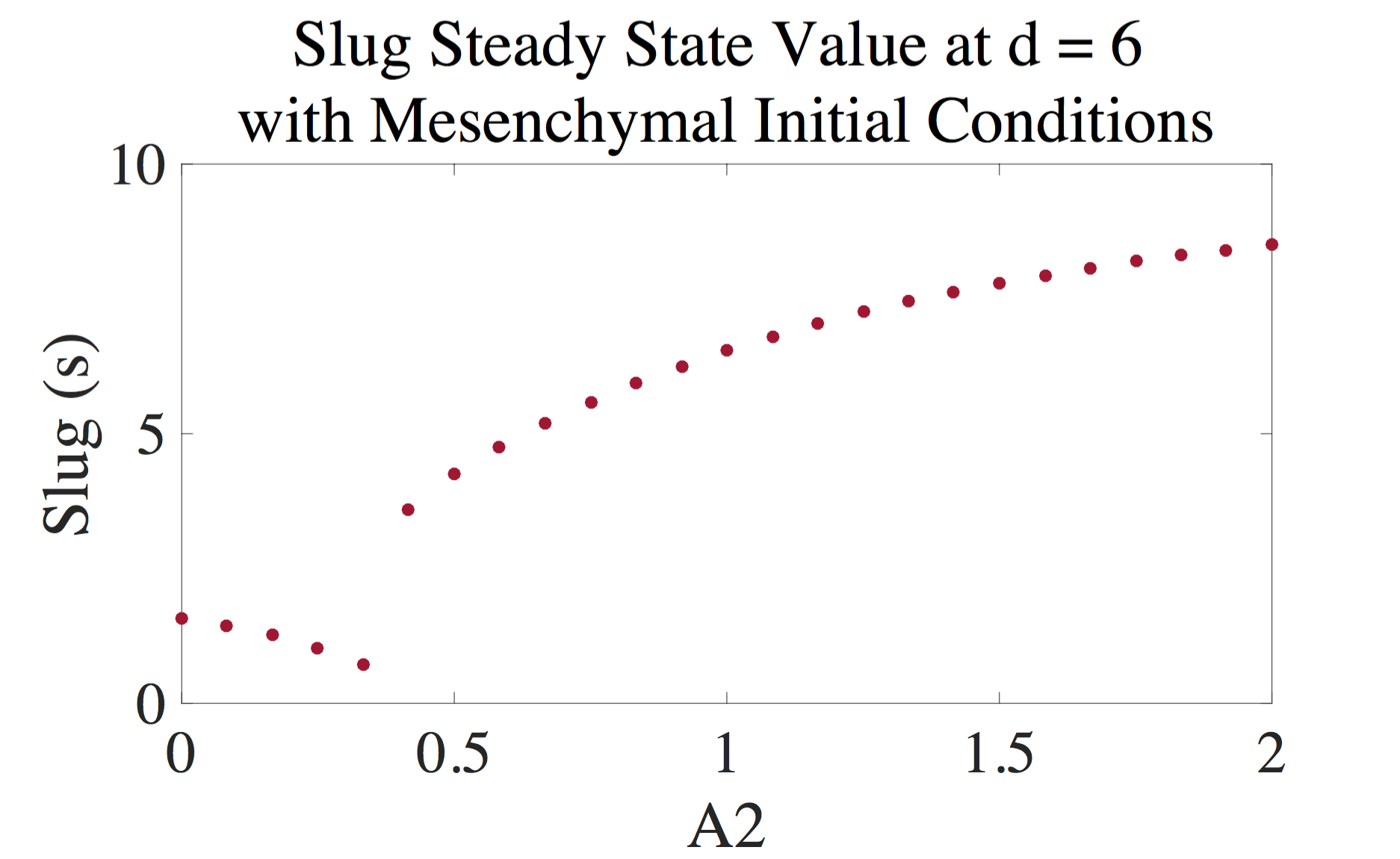 |
| Figure S1AI | Figure S1AJ |
|  |  |
| 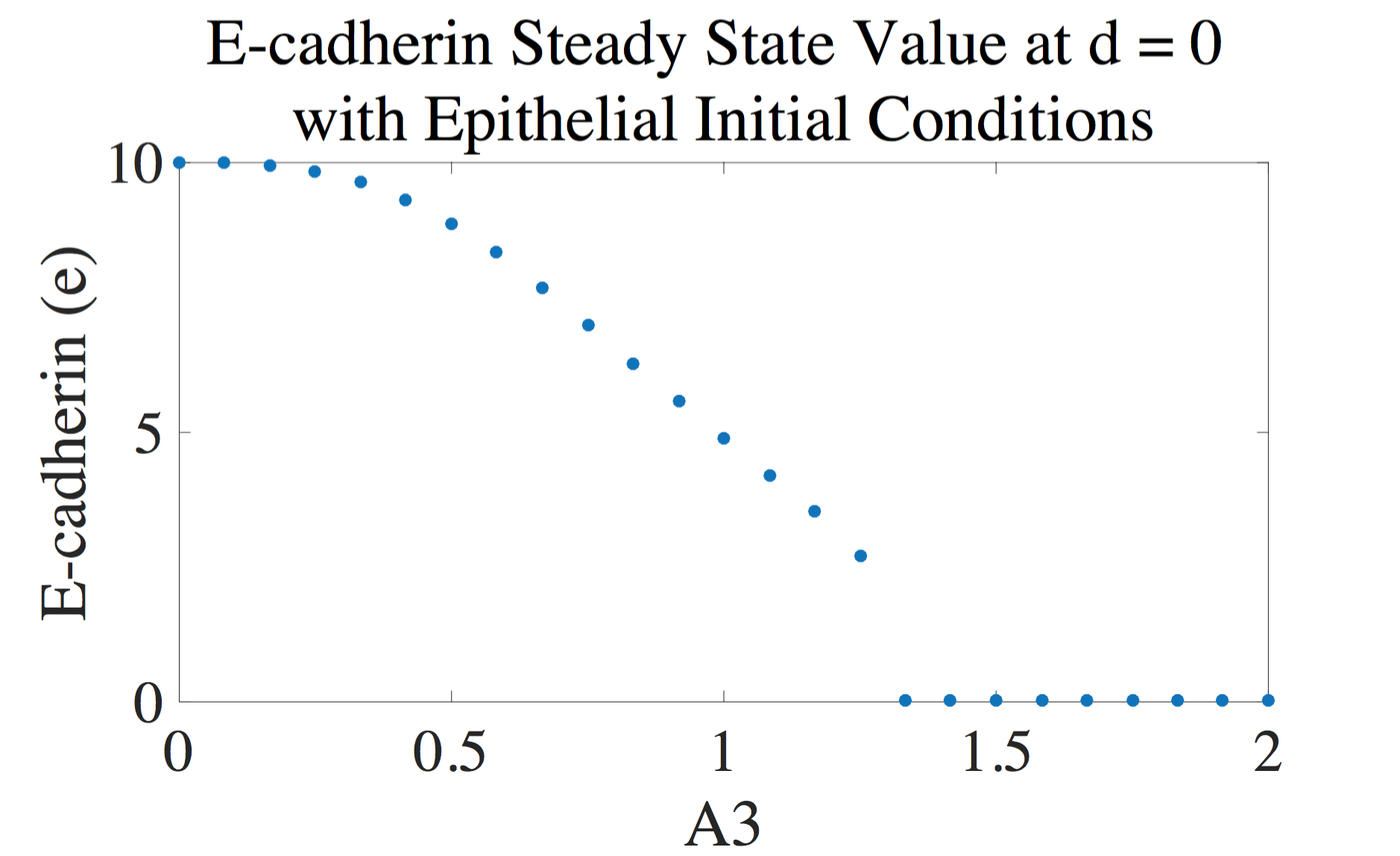 | 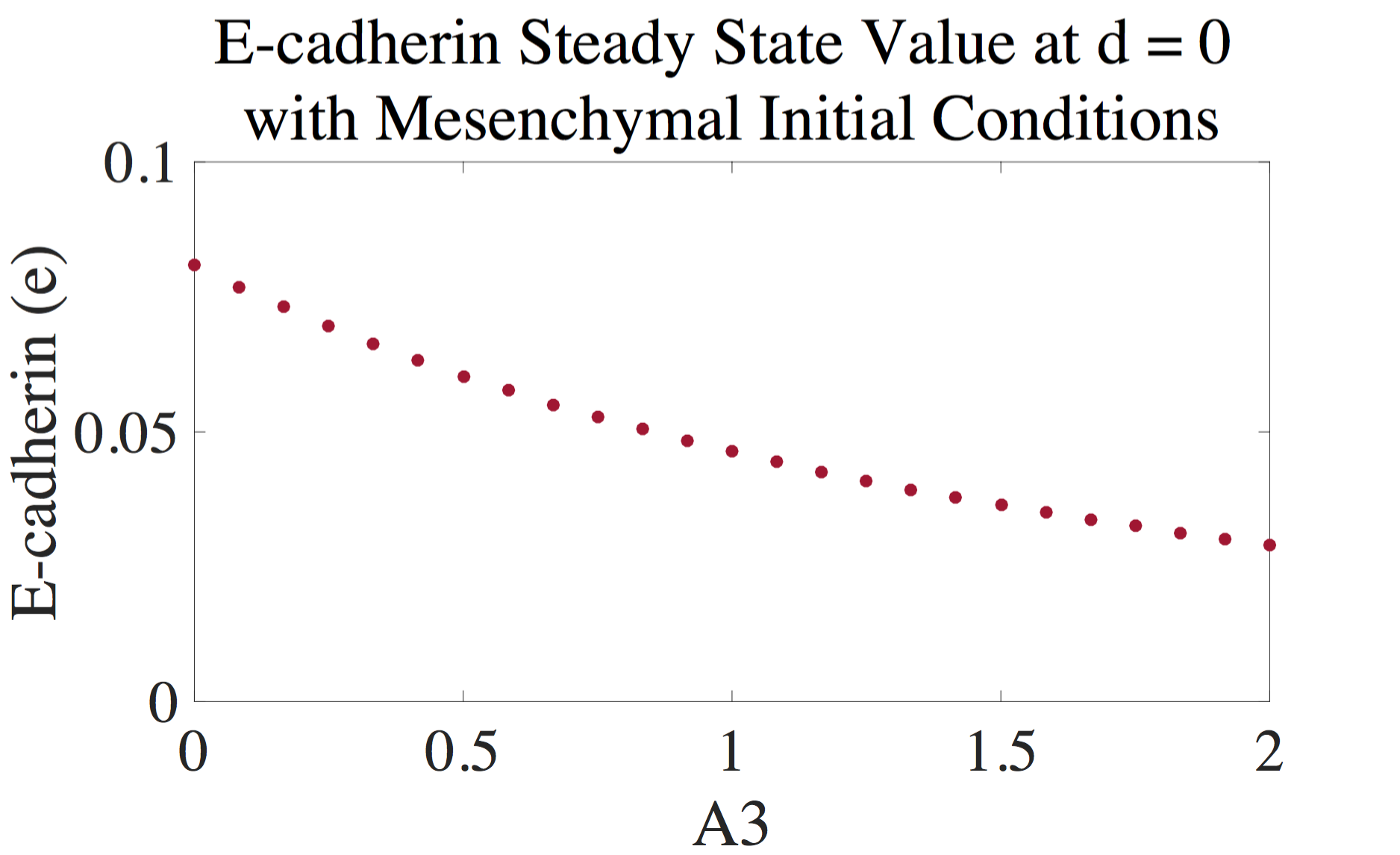 |
| Figure S1AK | Figure S1AL |
|  |  |
| 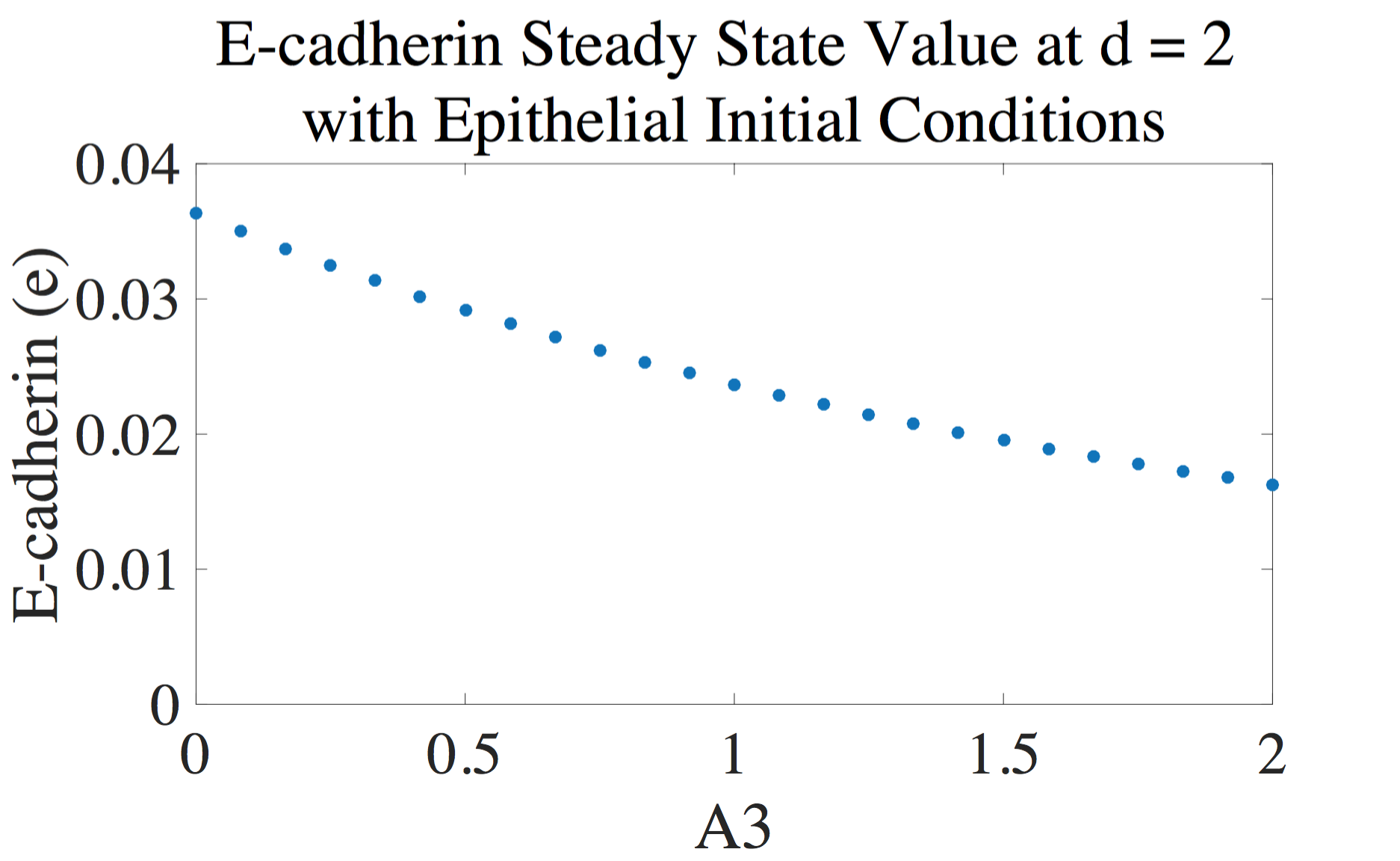 | 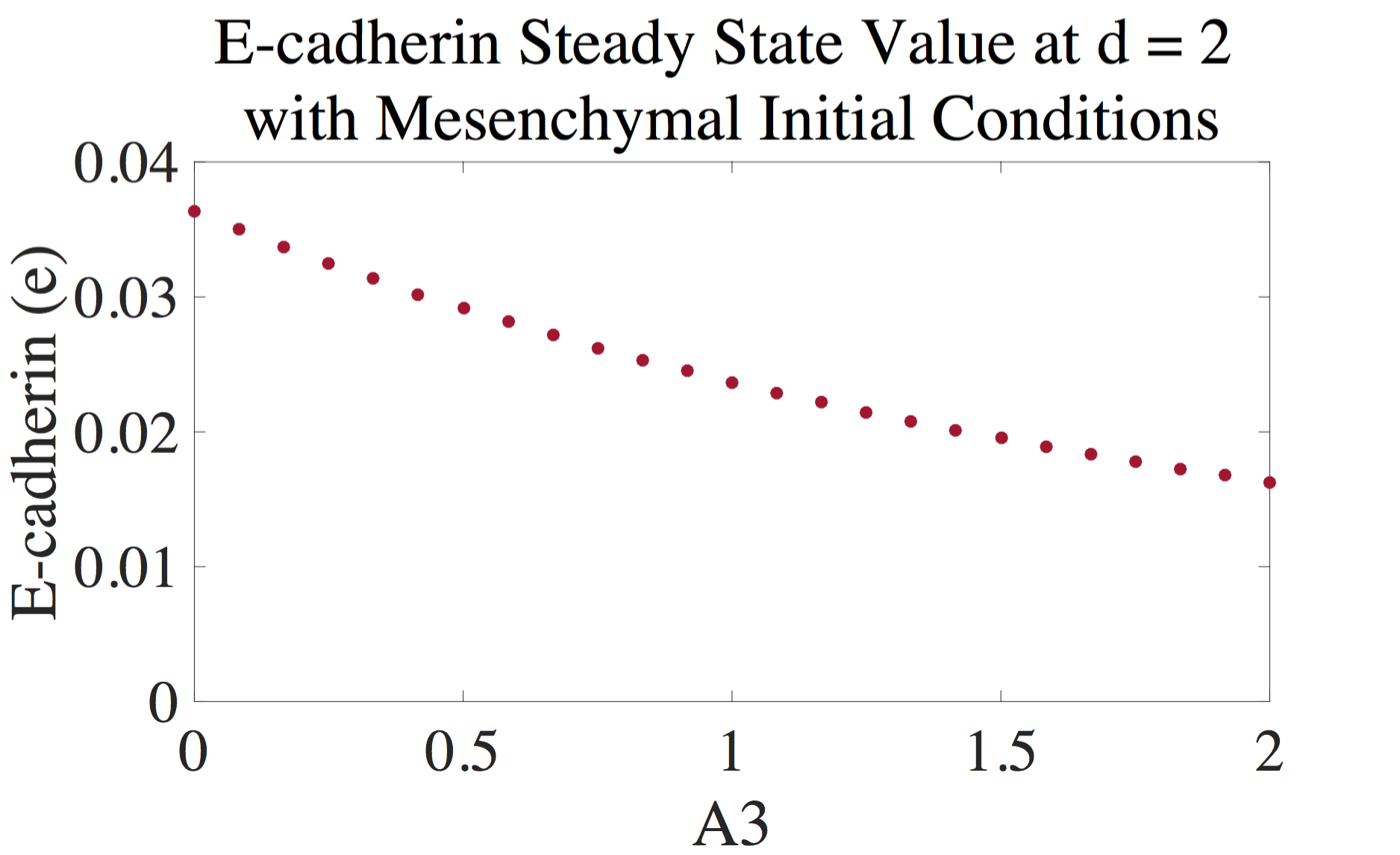 |
| Figure S1AM | Figure S1AN |
|  |  |
| 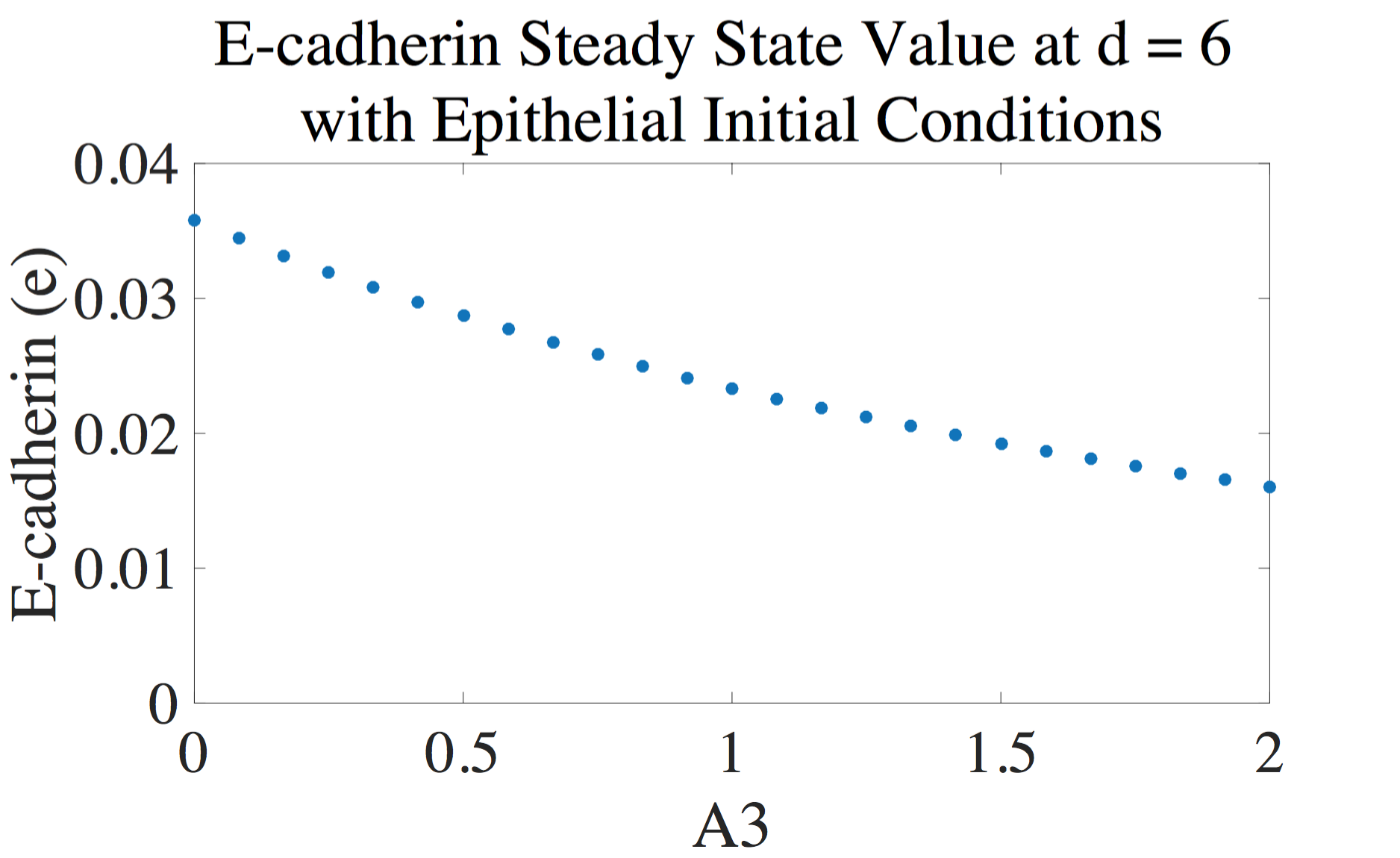 | 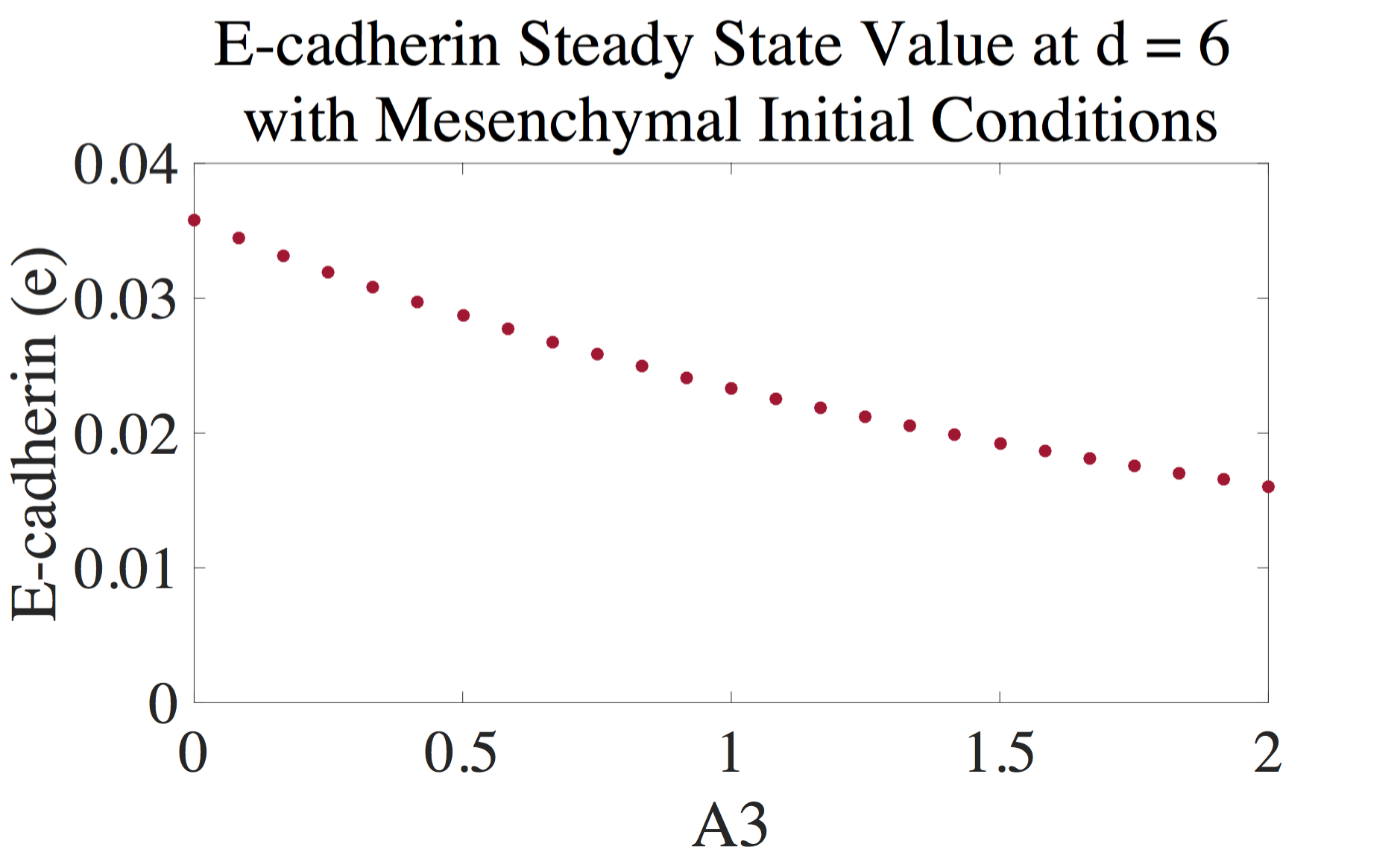 |
| Figure S1AO | Figure S1AP |
|  |  |
| 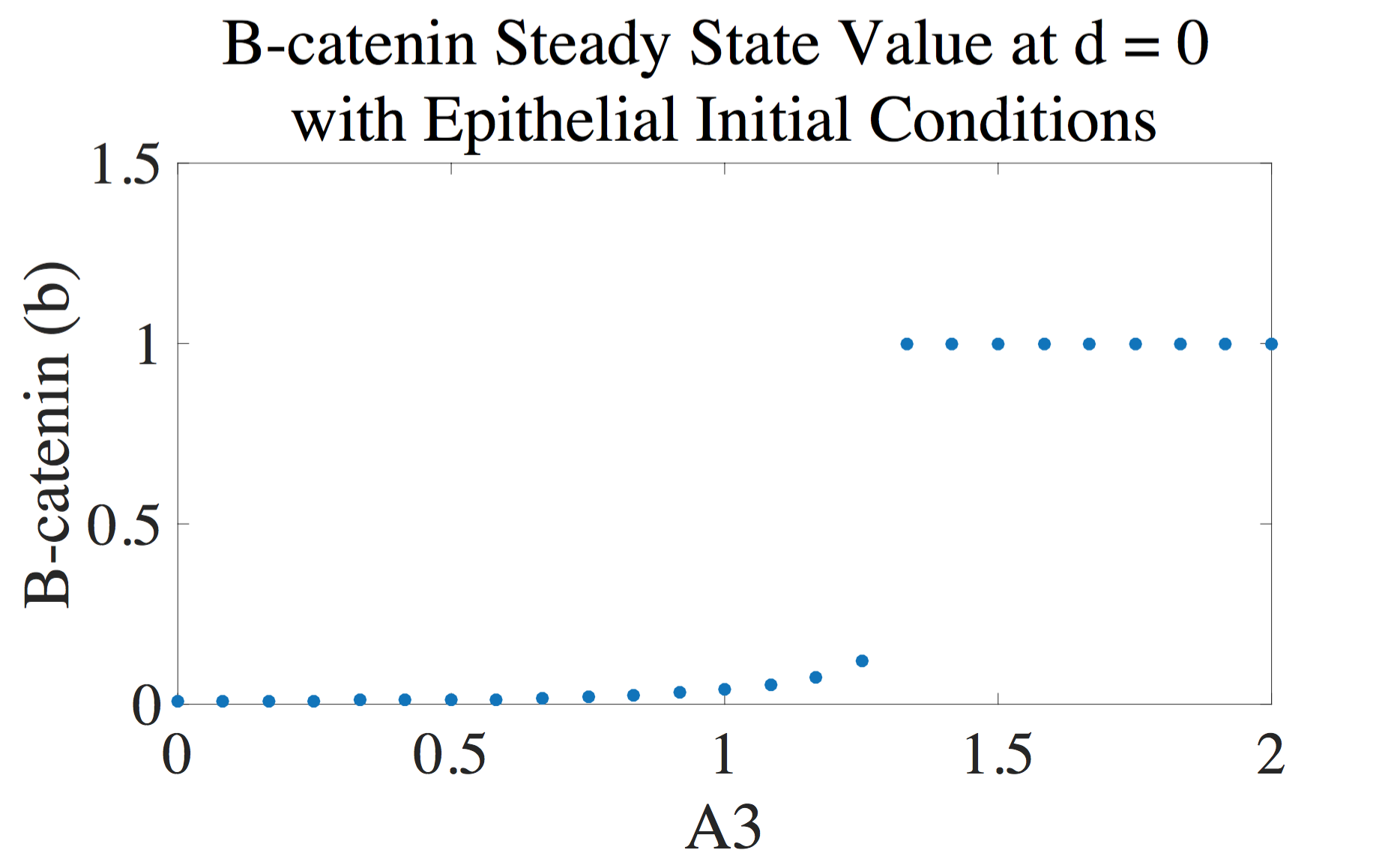 | 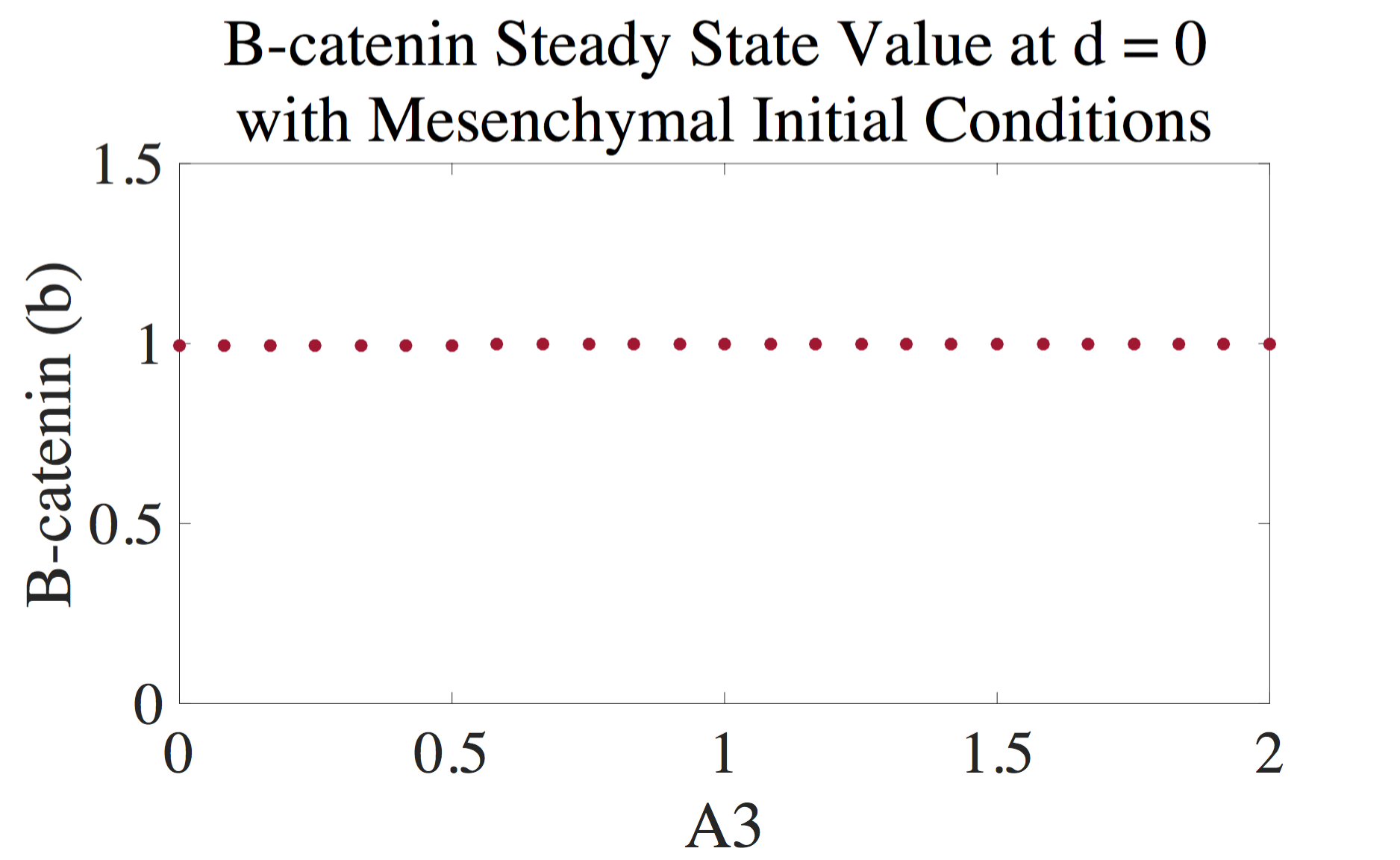 |
| Figure S1AQ | Figure S1R |
|  |  |
| 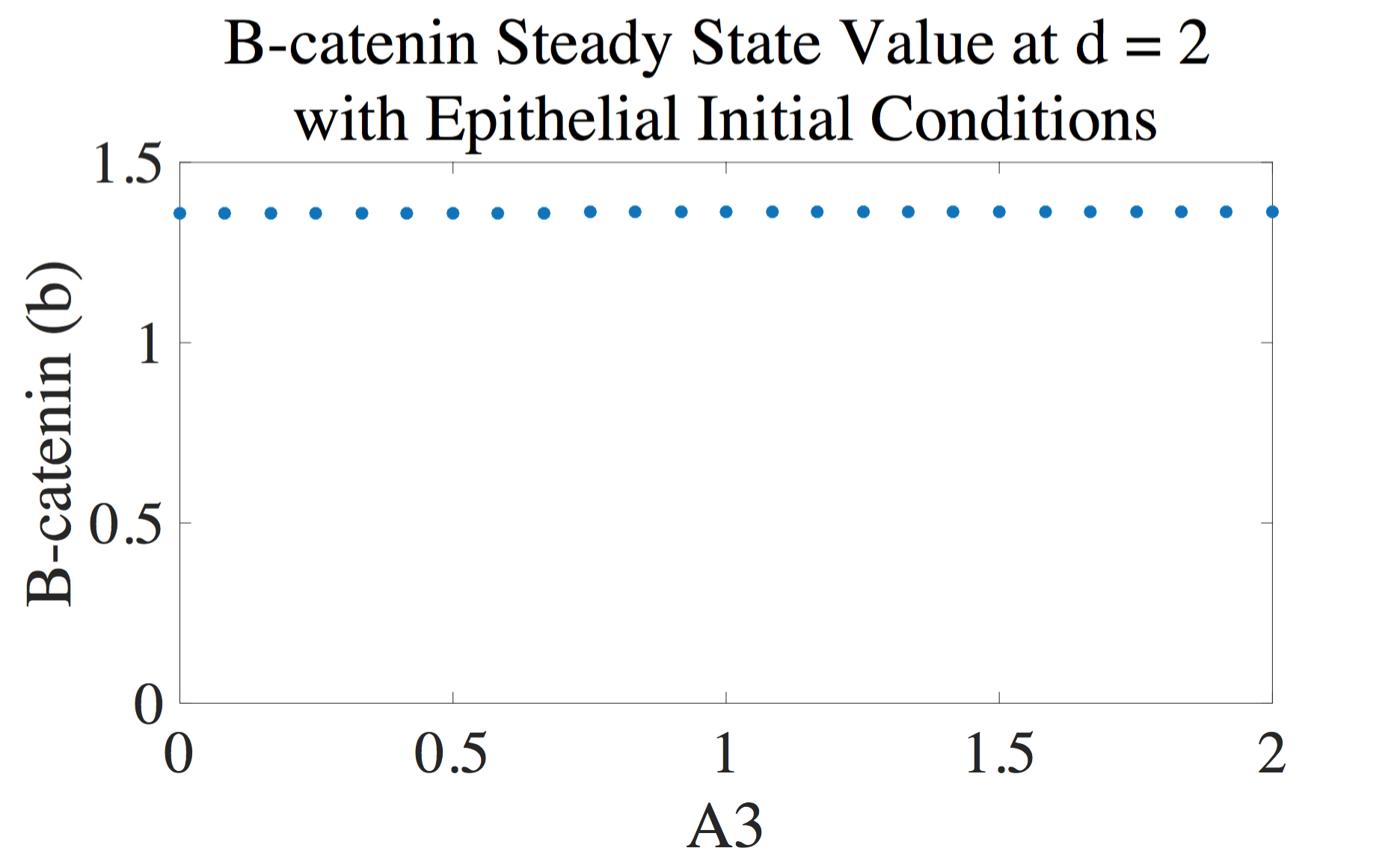 | 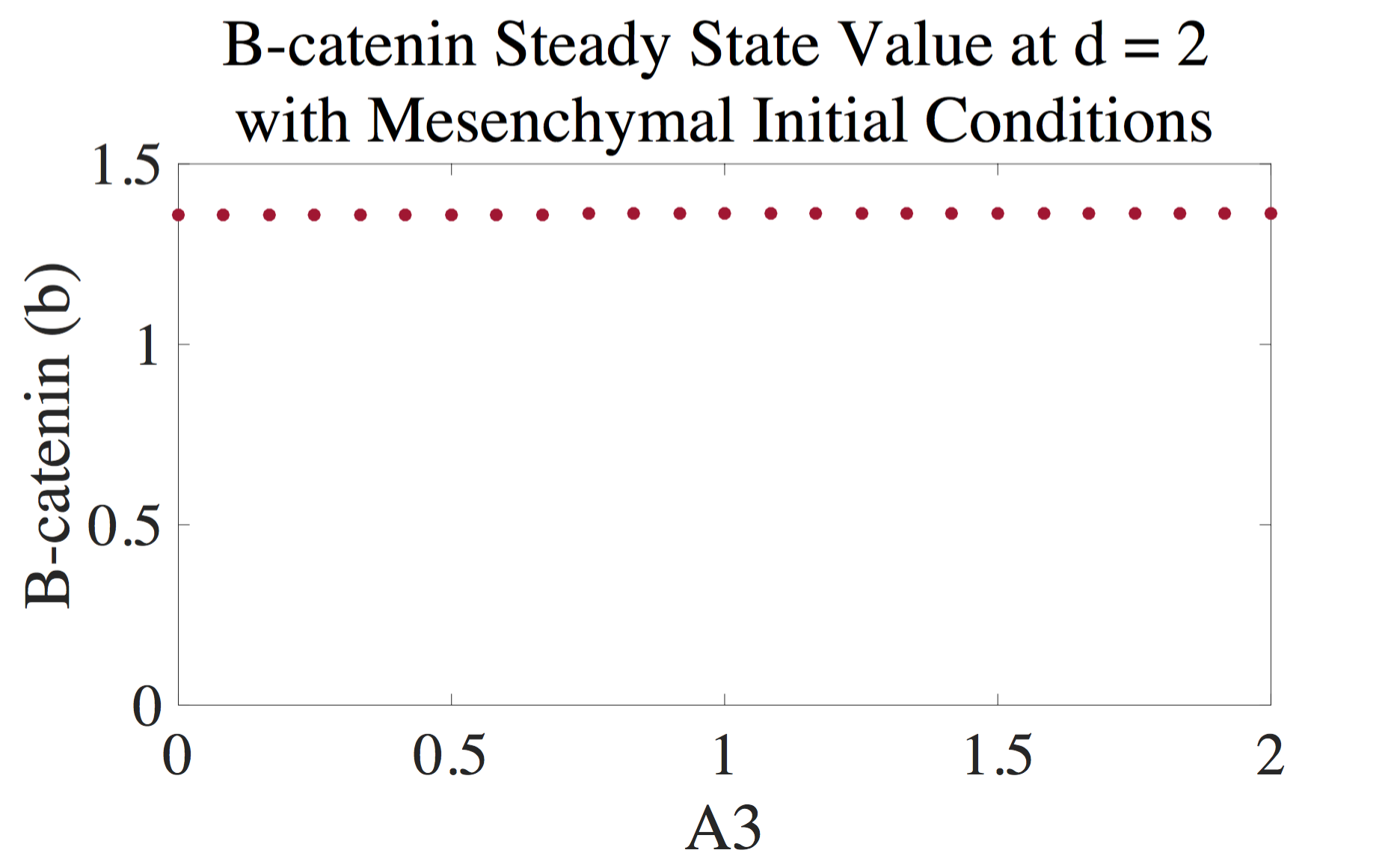 |
| Figure S1AS | Figure S1AT |
|  |  |
| 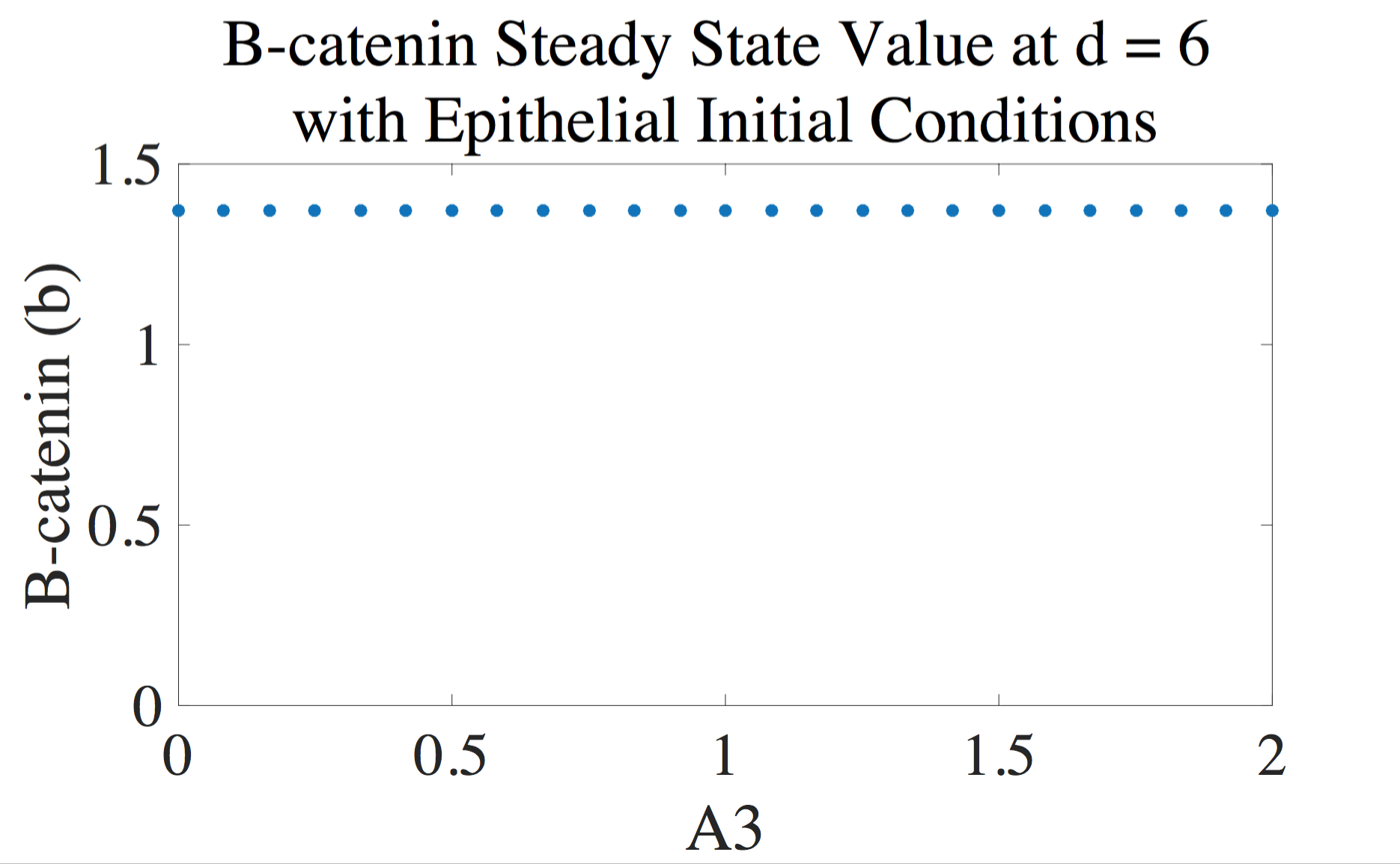 | 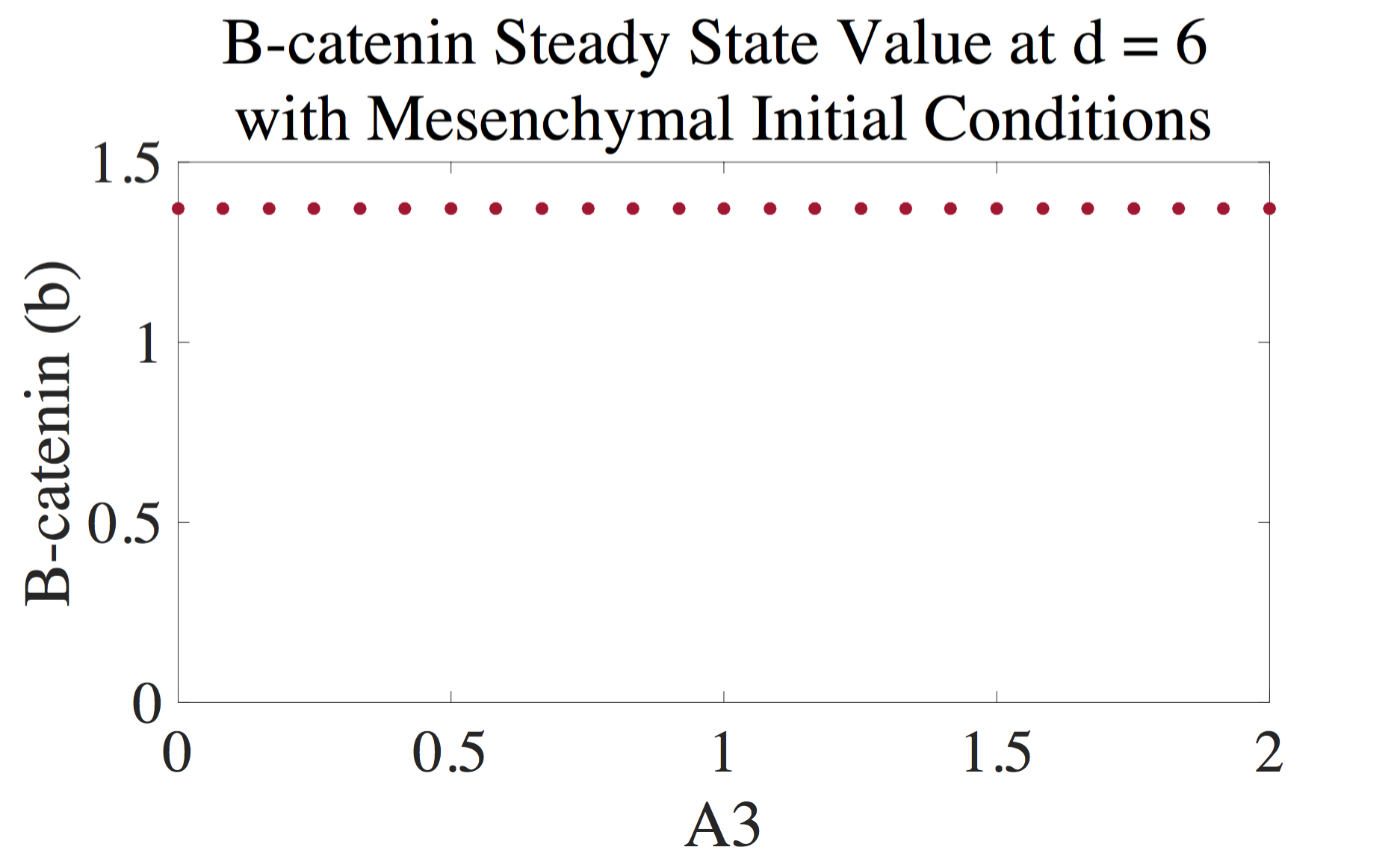 |
| Figure S1AU | Figure S1AV |
|  |  |
| 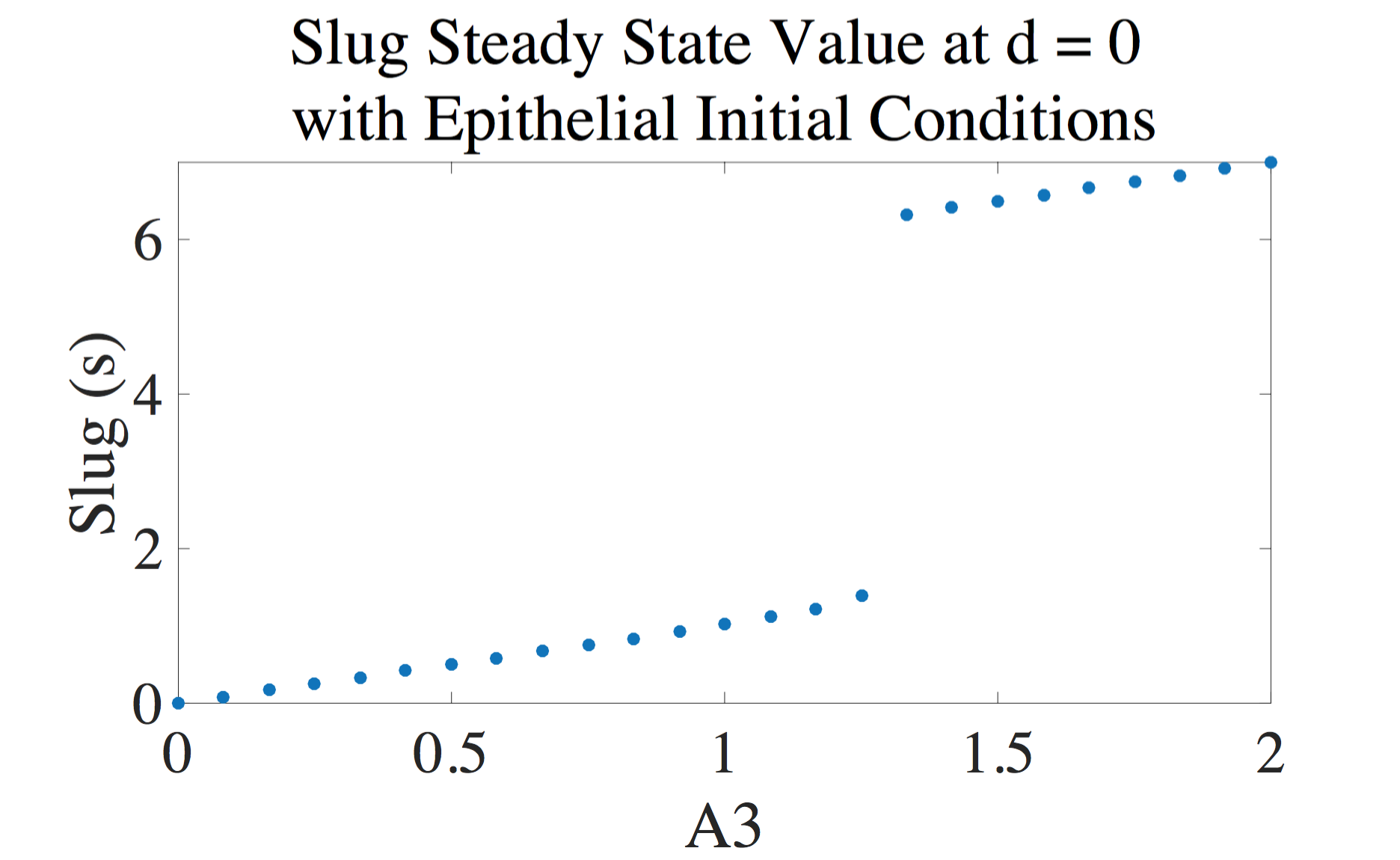 | 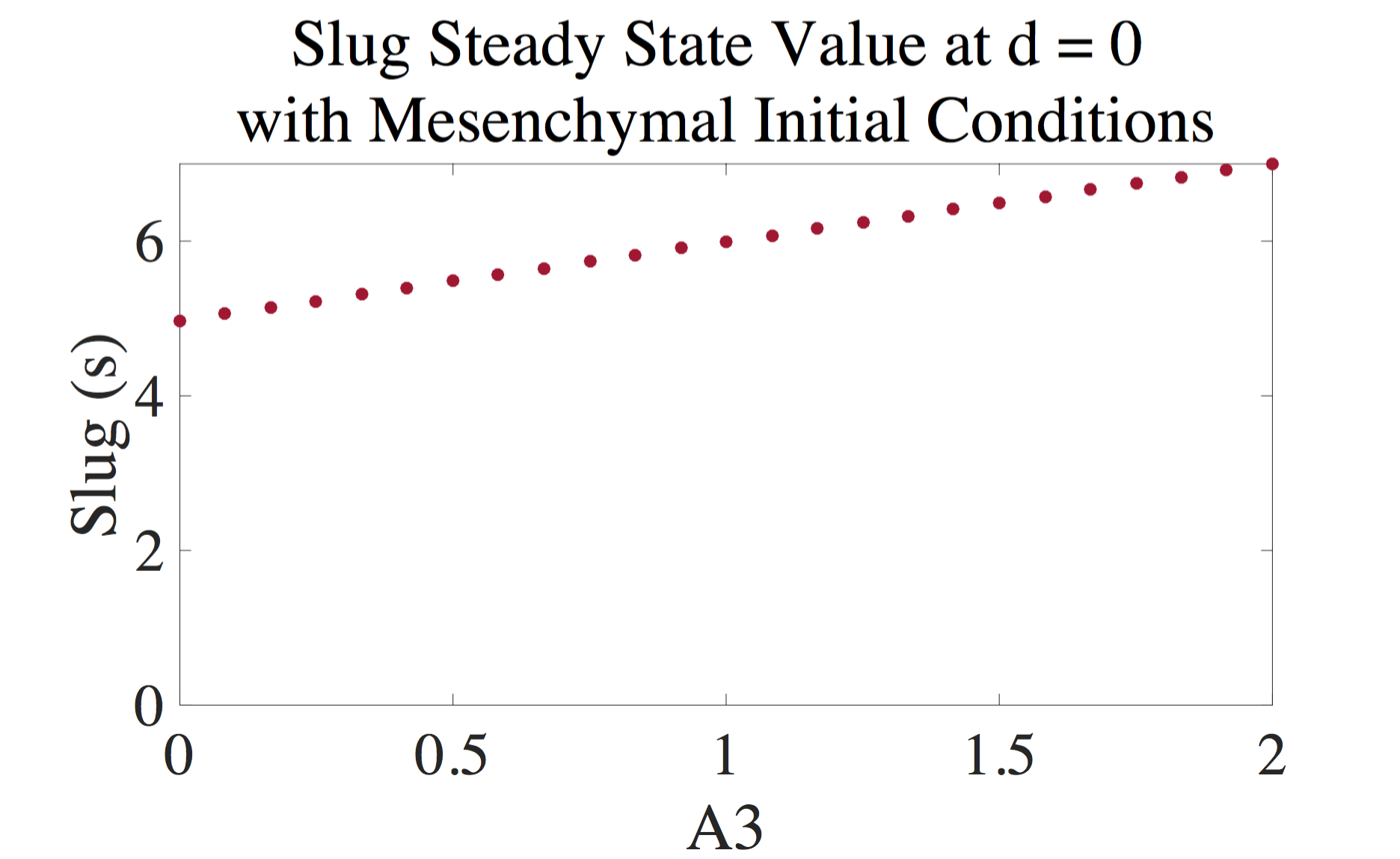 |
| Figure S1AW | Figure S1AX |
|  |  |
| 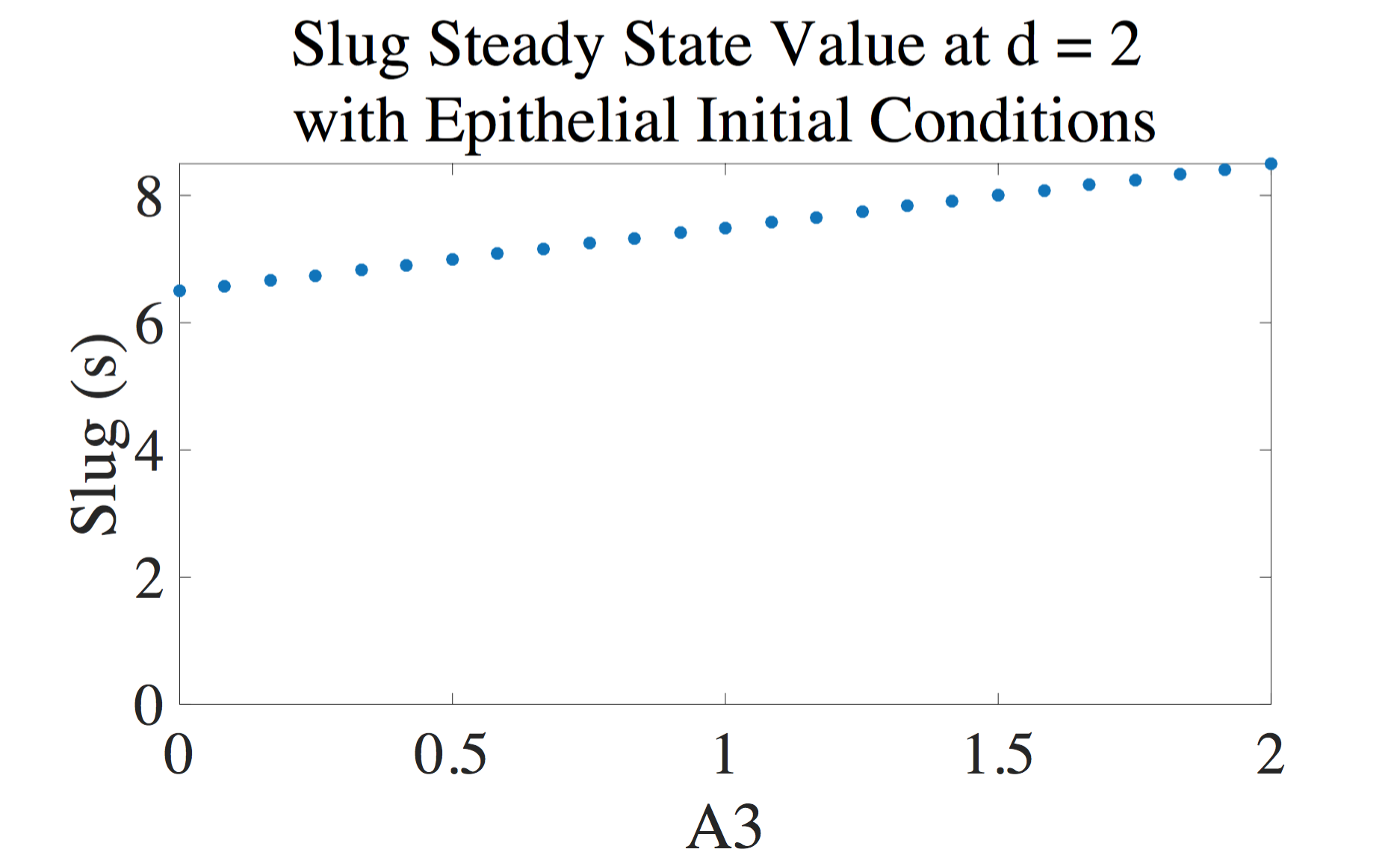 | 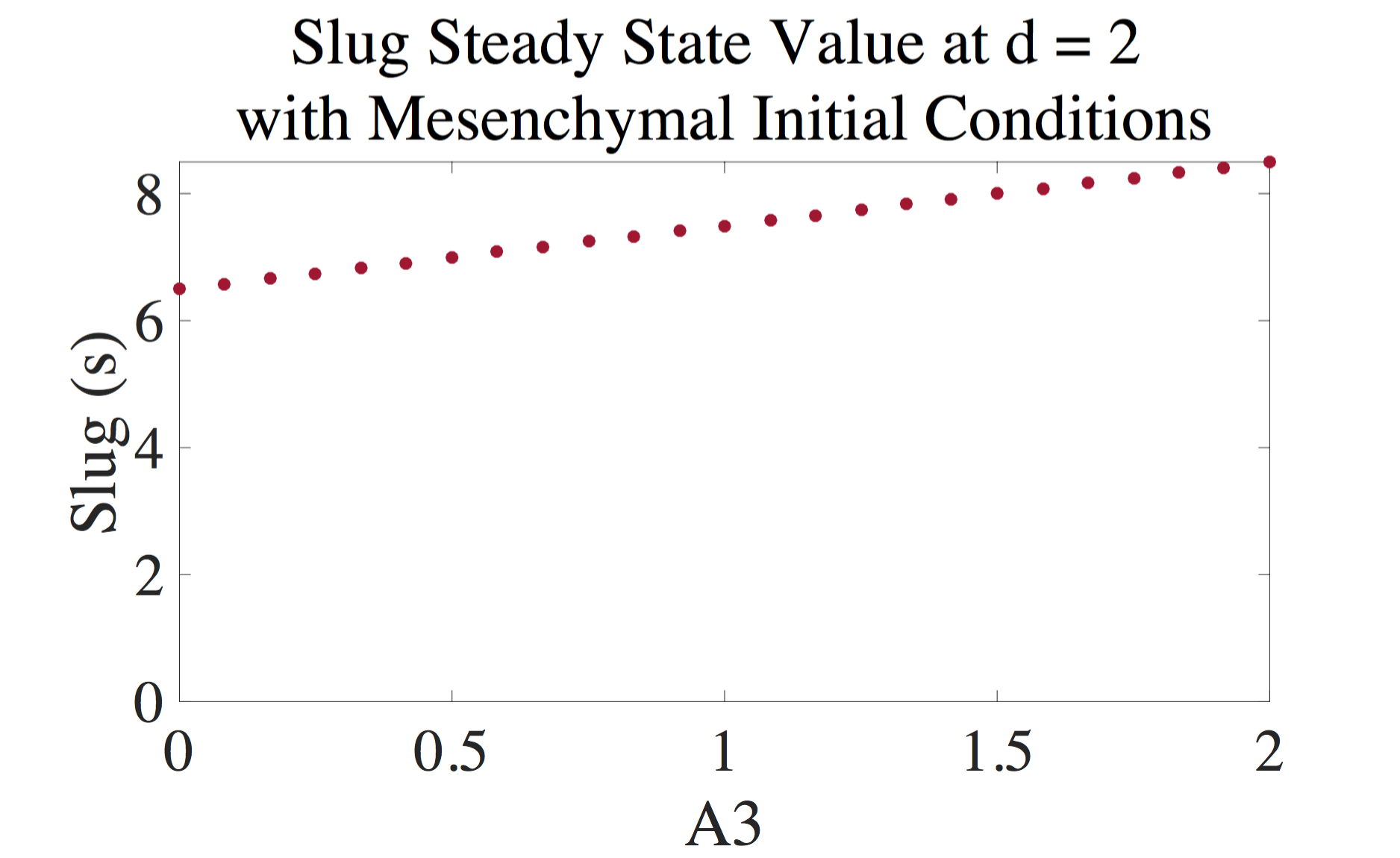 |
| Figure S1AY | Figure S1AZ |
|  |  |
| 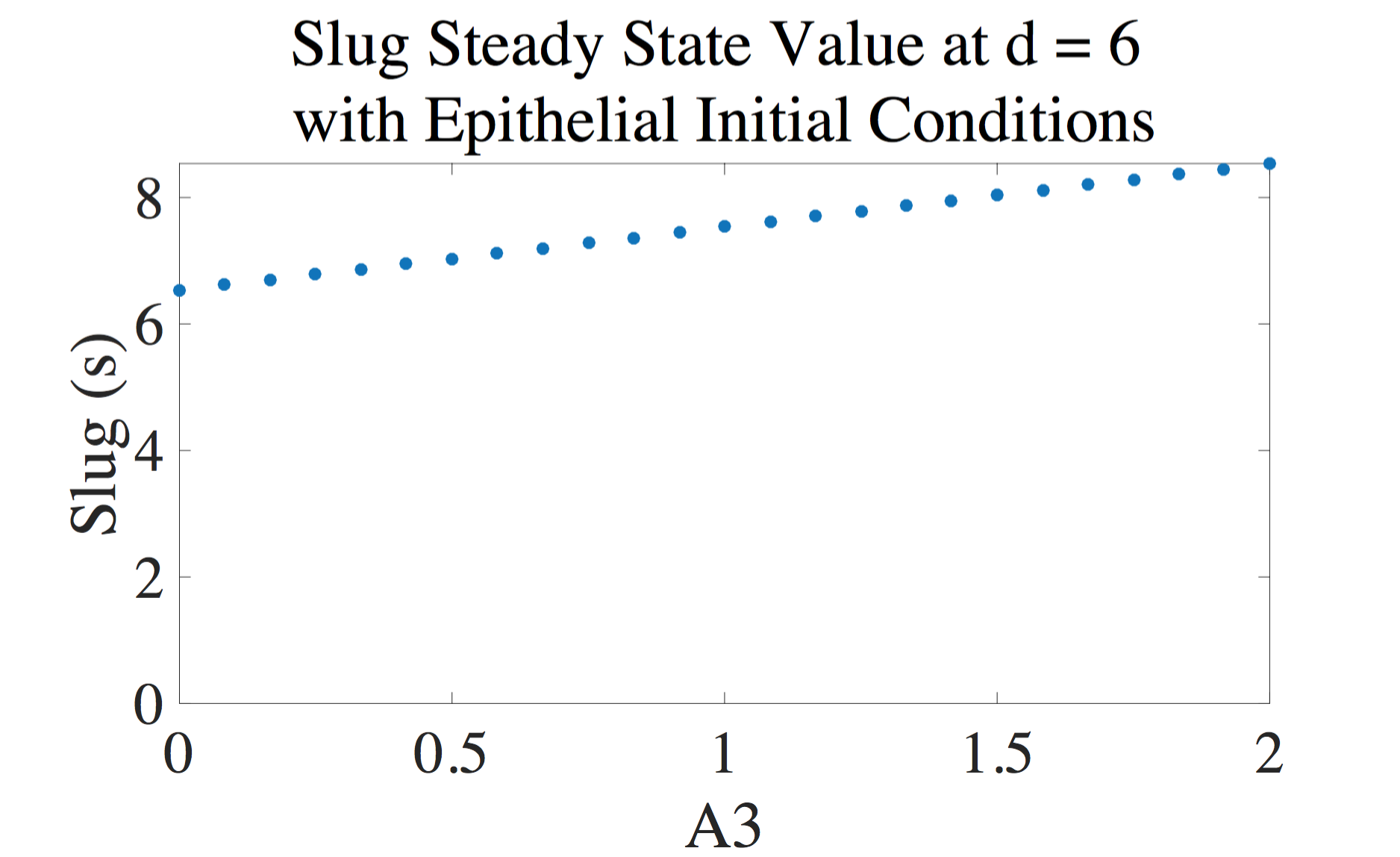 | 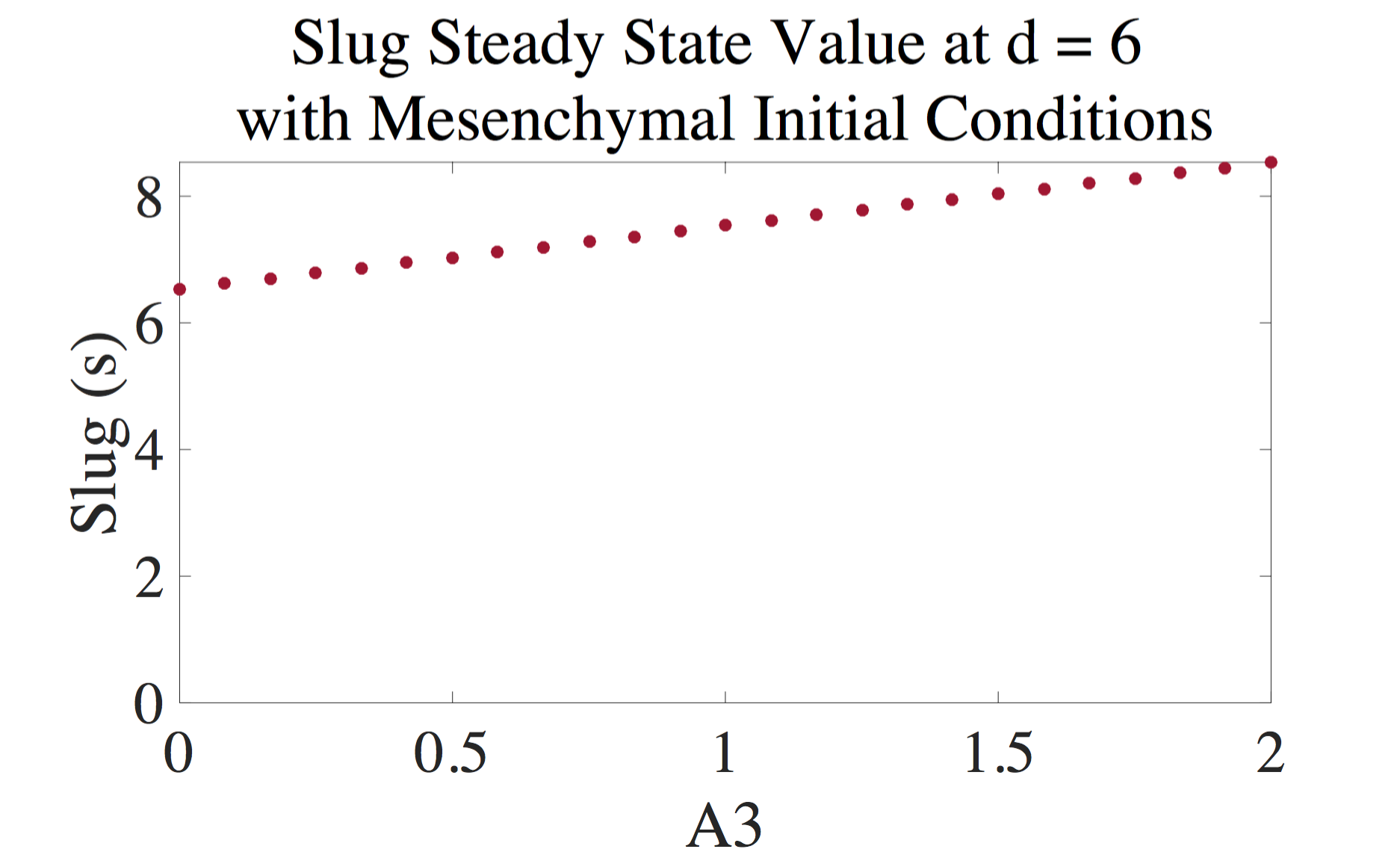 |
| Figure S1BA | Figure S1BB |
|  |  |
| 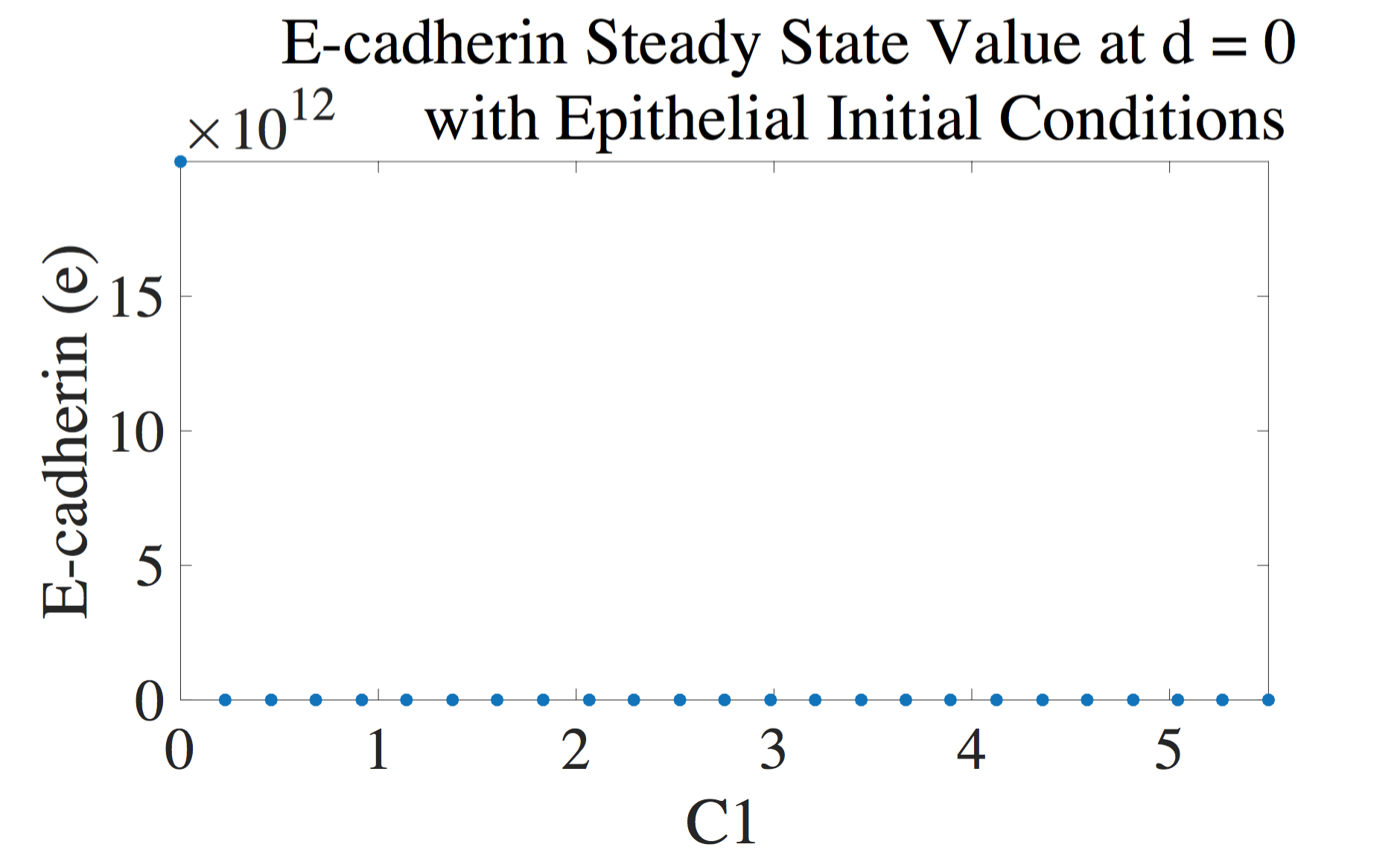 | 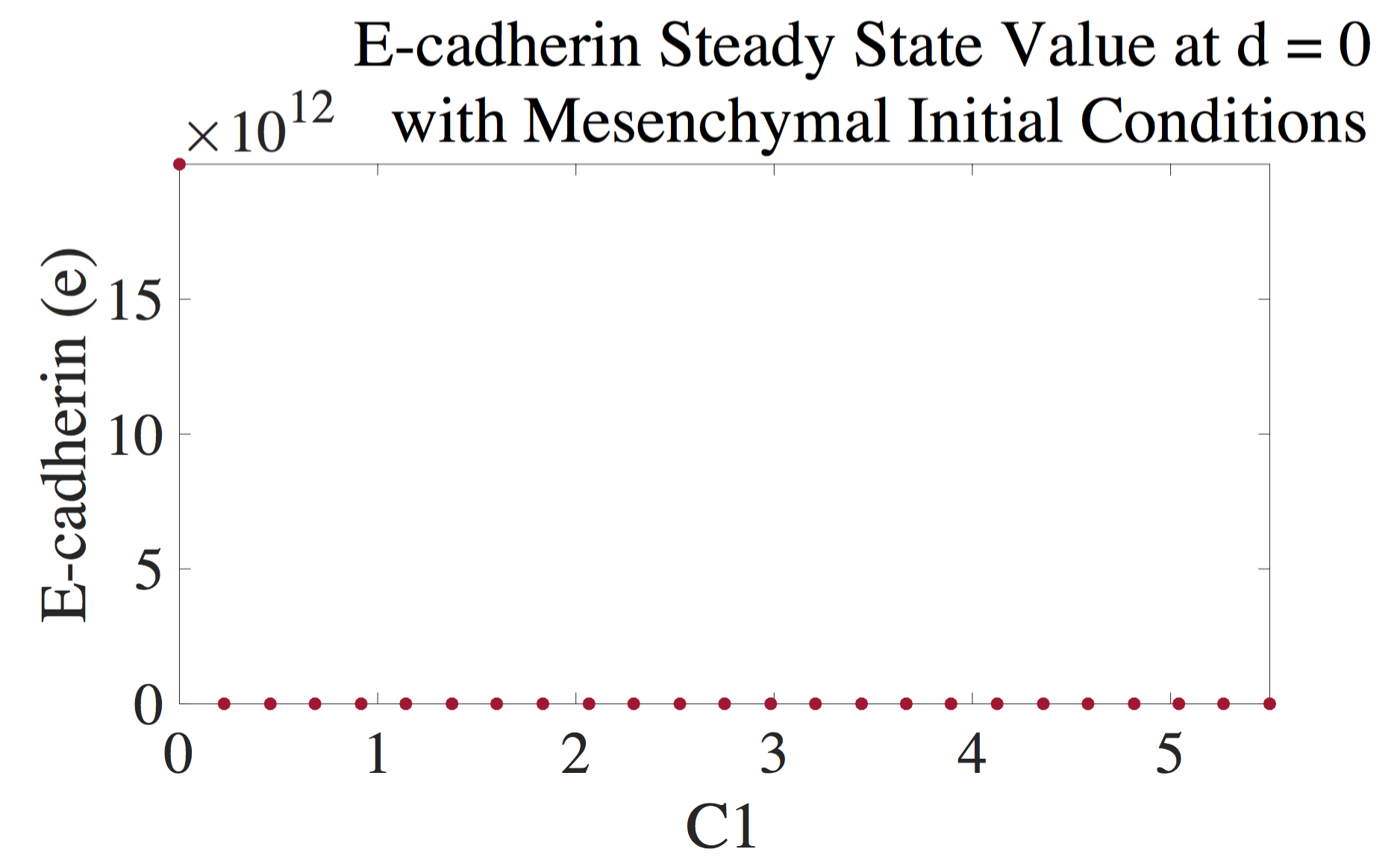 |
| Figure S1BC | Figure S1BD |
|  |  |
| 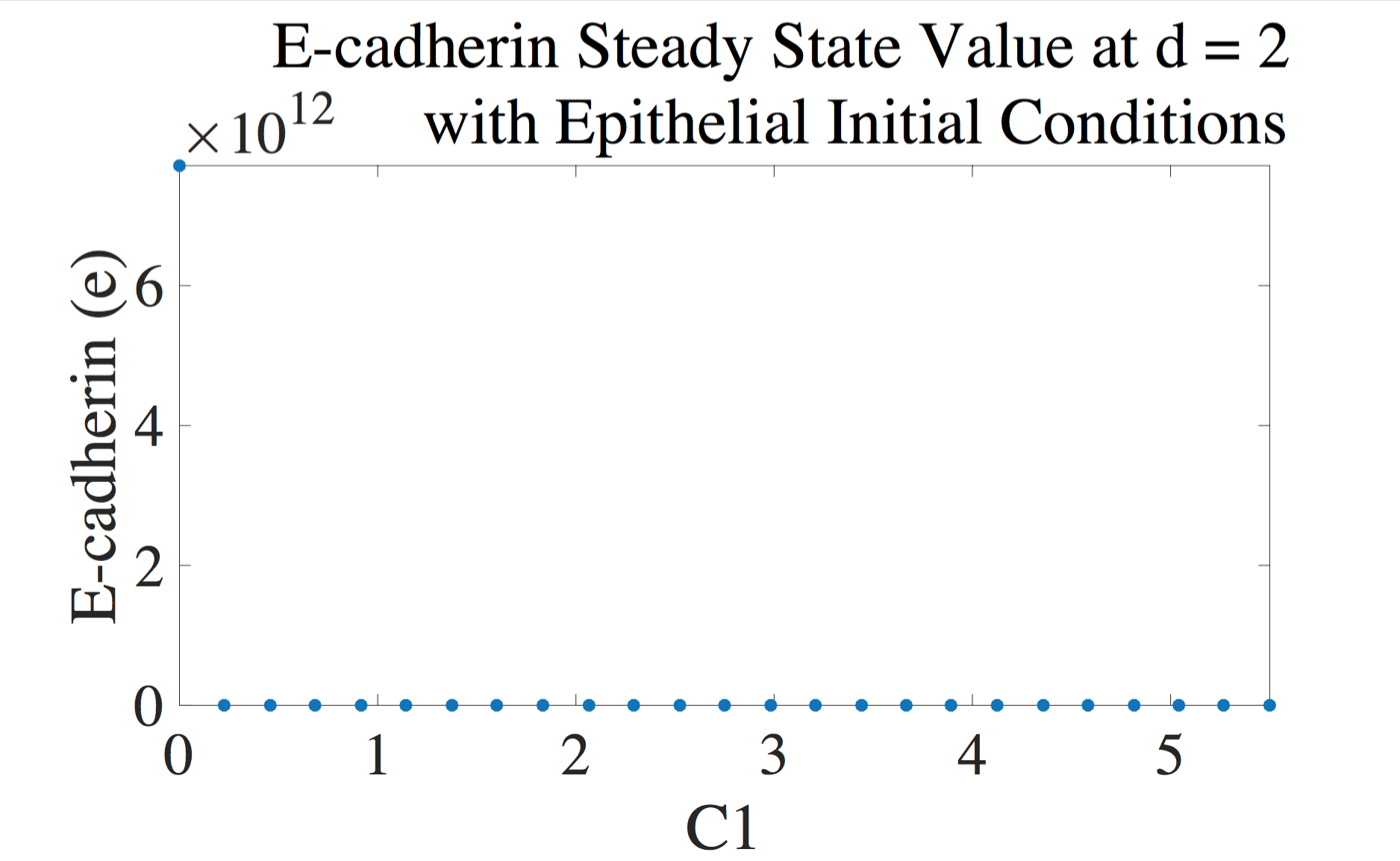 | 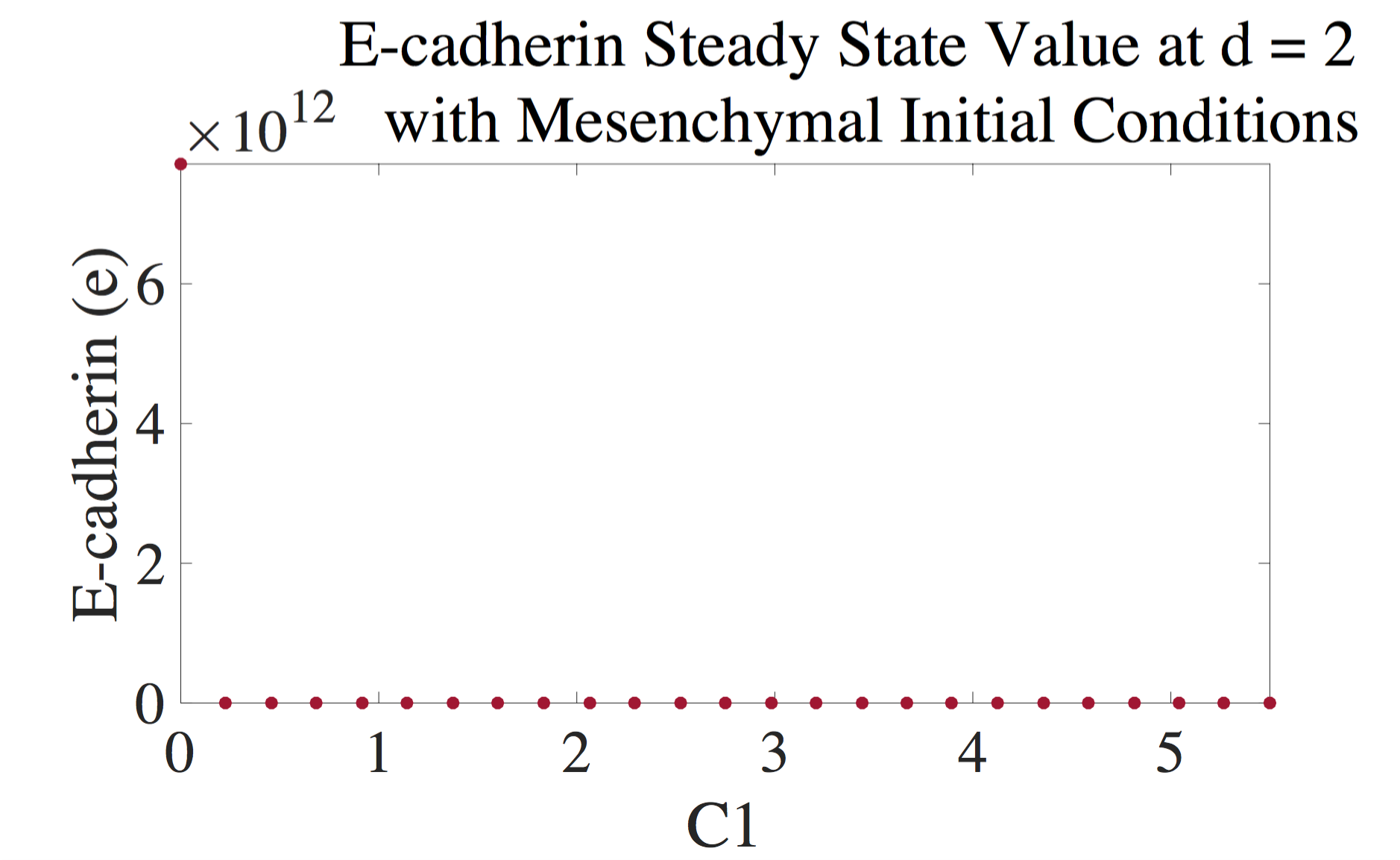 |
| Figure S1BE | Figure S1BF |
|  |  |
| 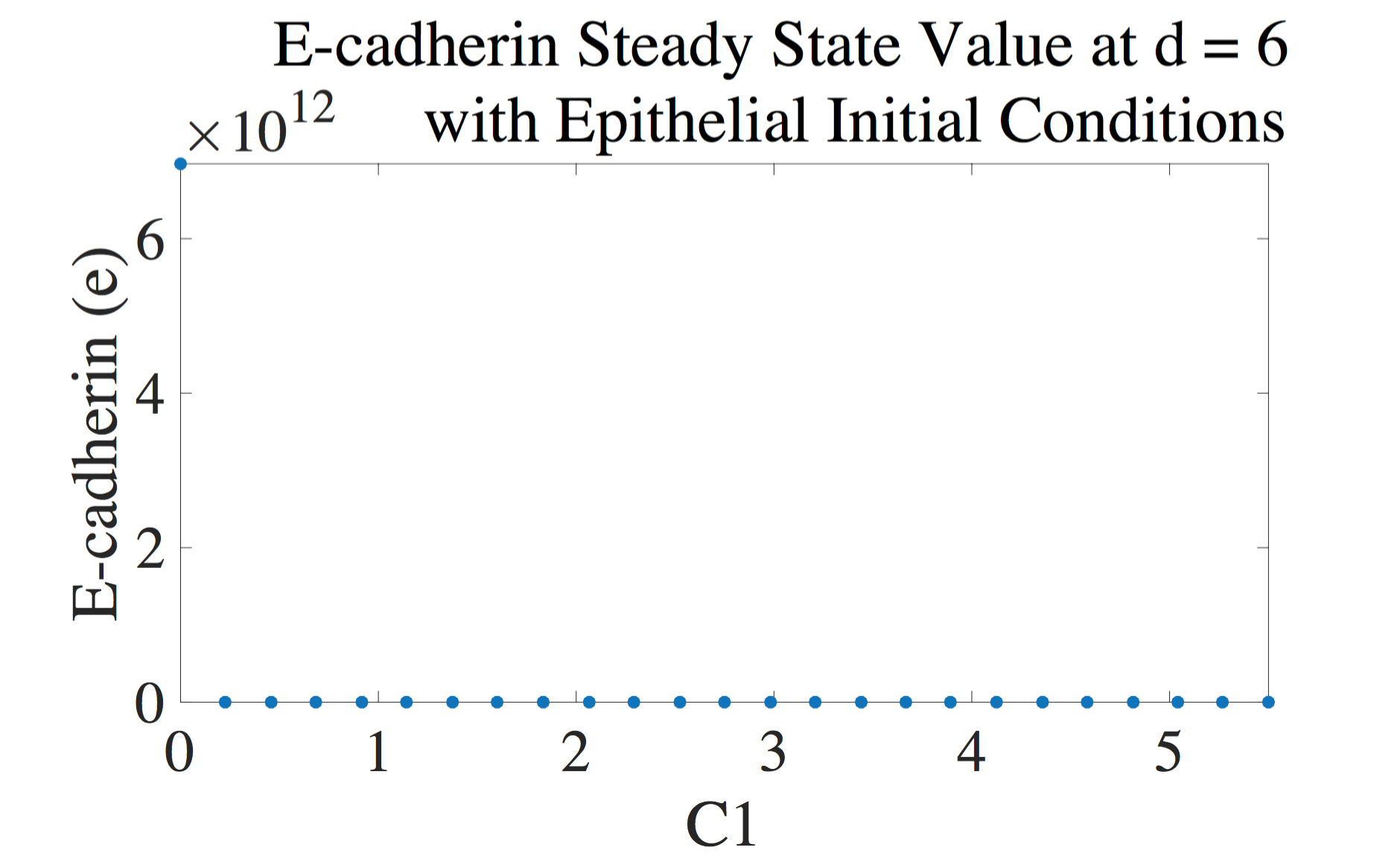 | 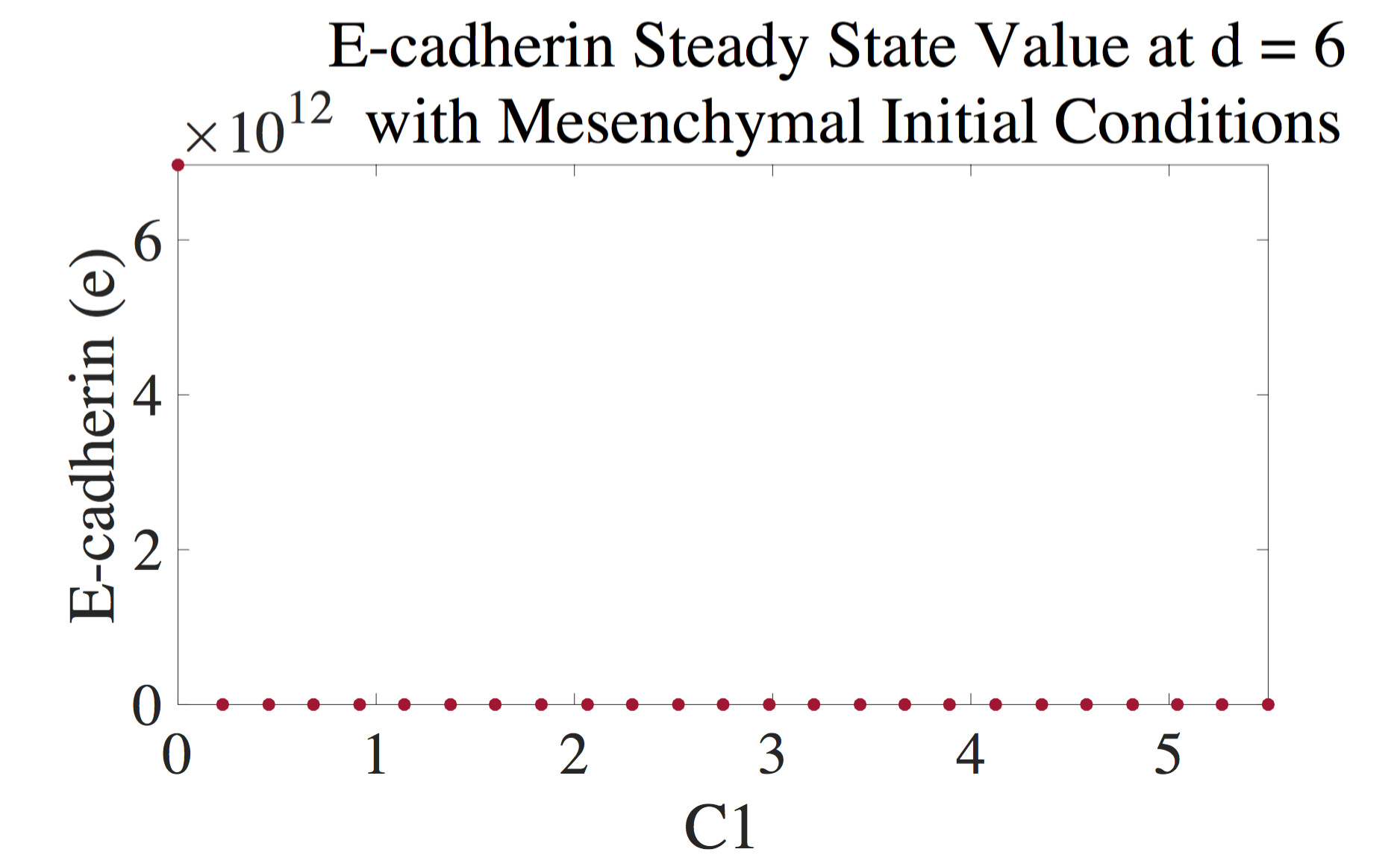 |
| Figure S1BG | Figure S1BH |
|  |  |
| 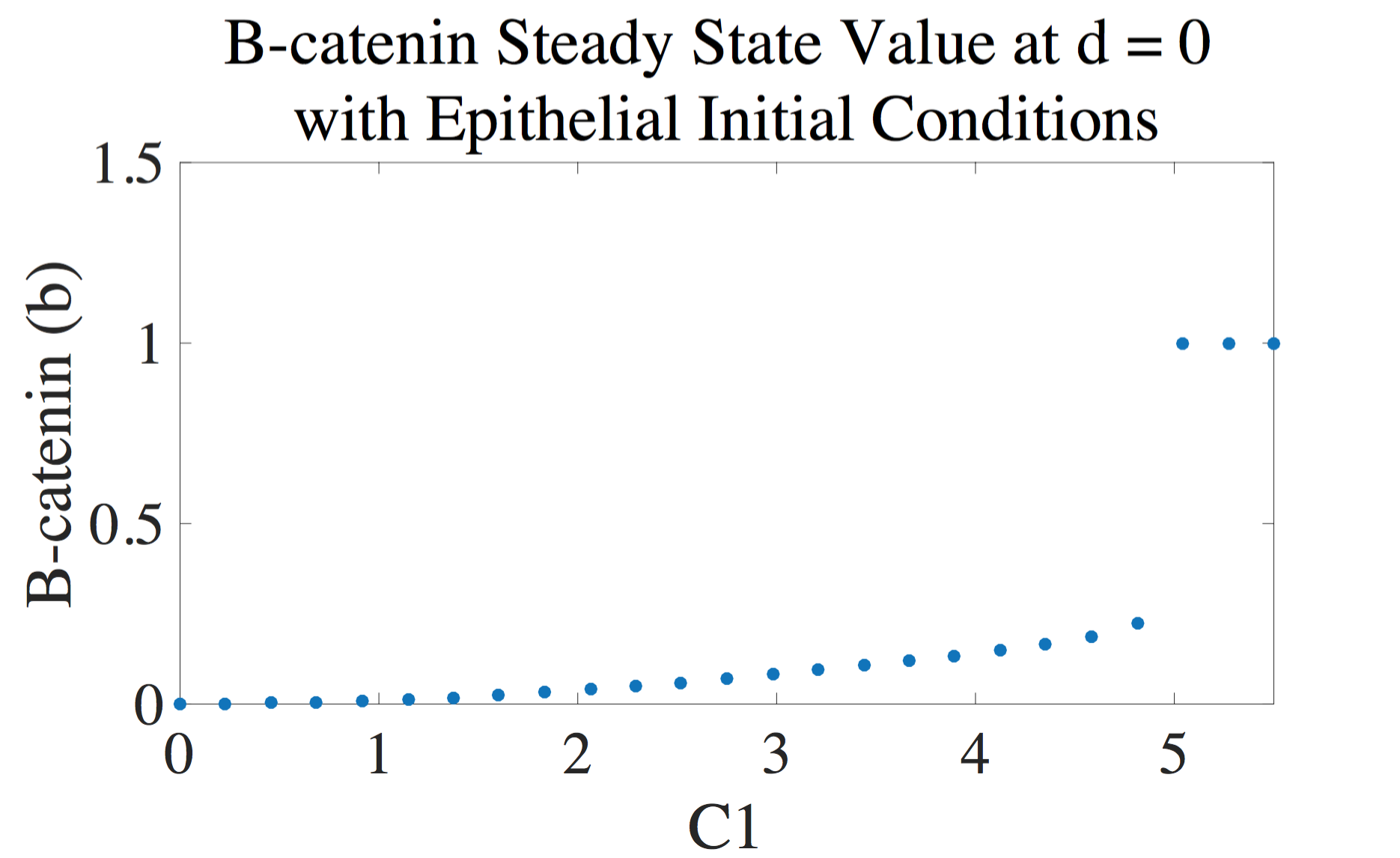 | 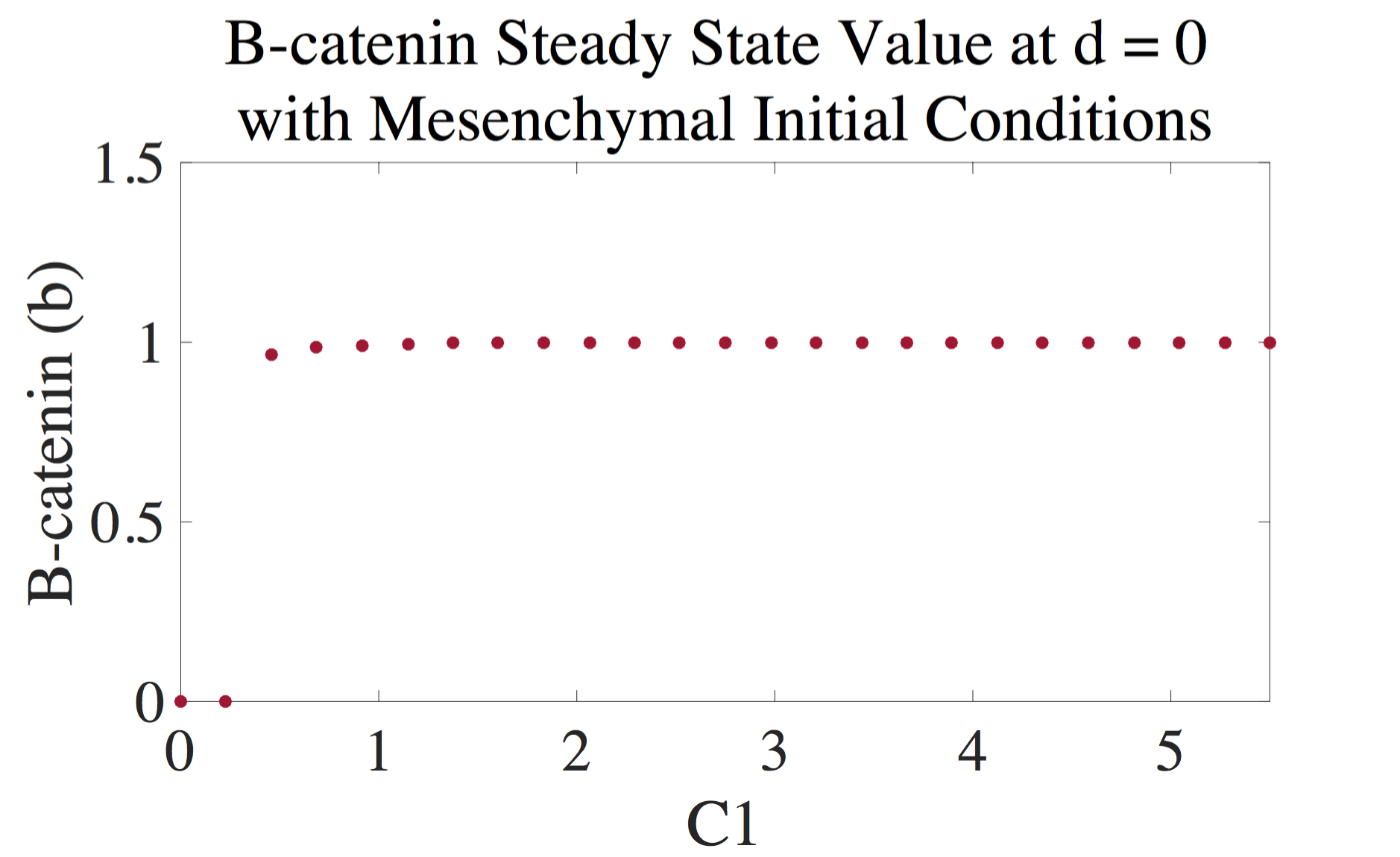 |
| Figure S1BI | Figure S1BJ |
|  |  |
| 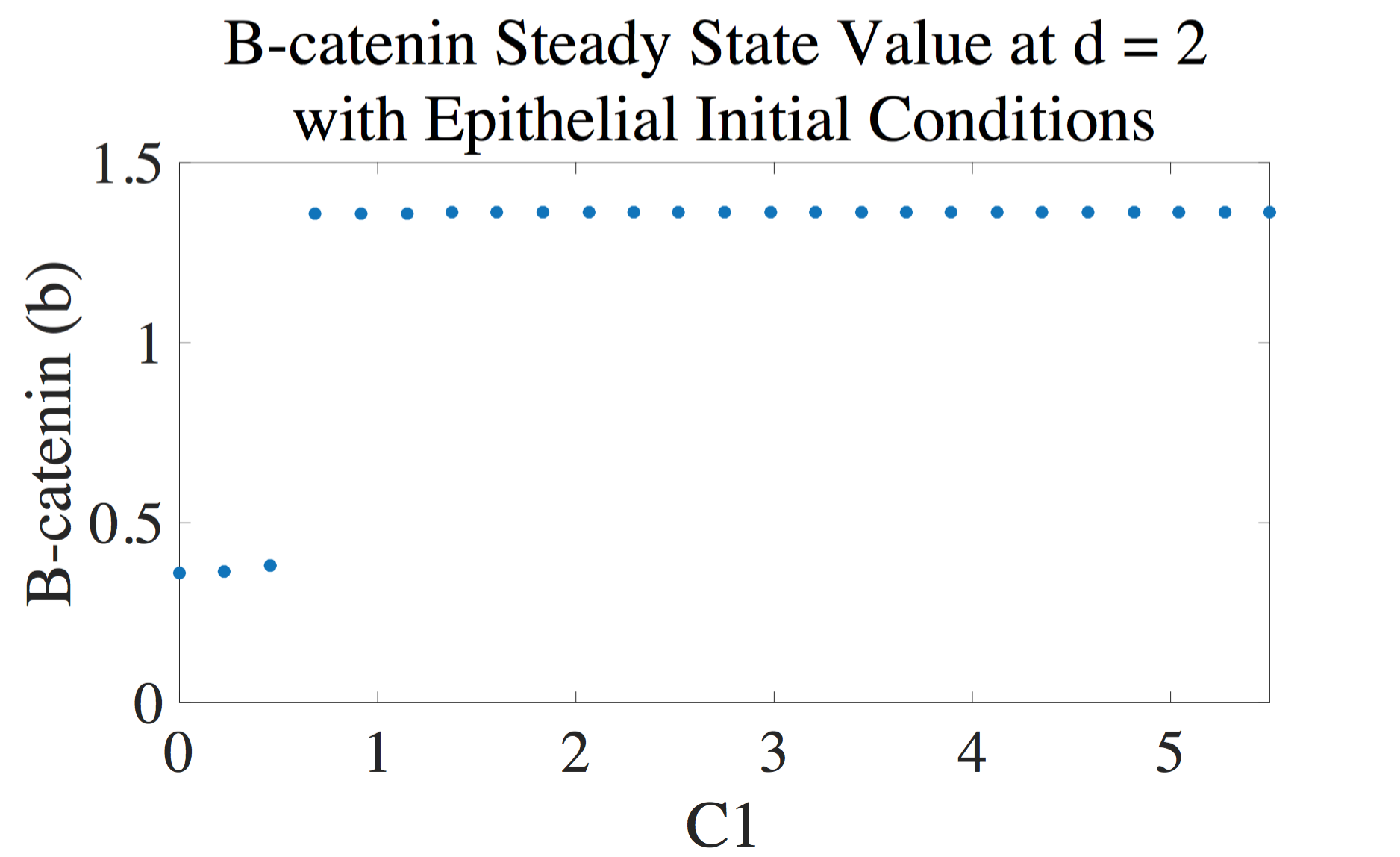 | 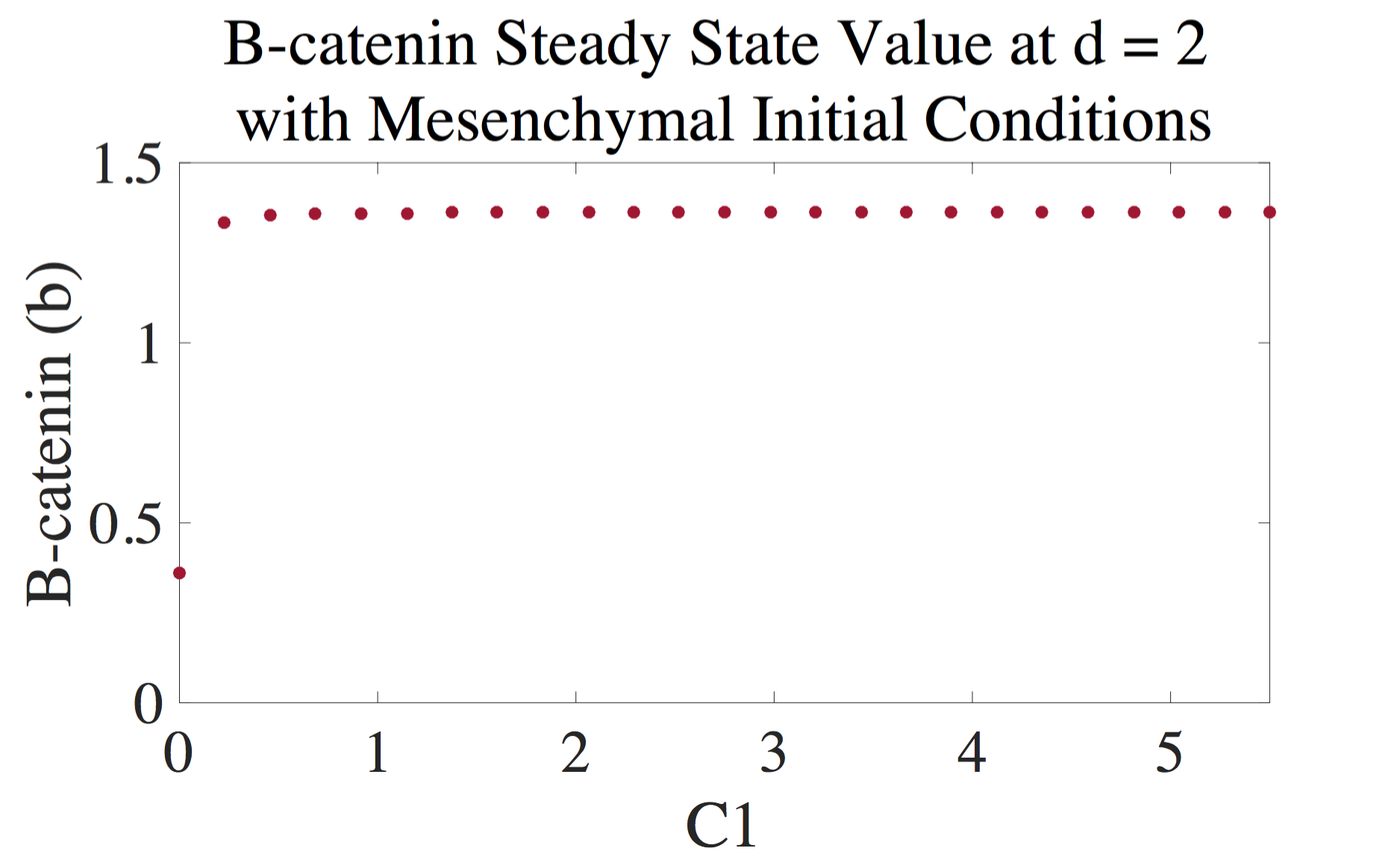 |
| Figure S1BK | Figure S1BL |
|  |  |
| 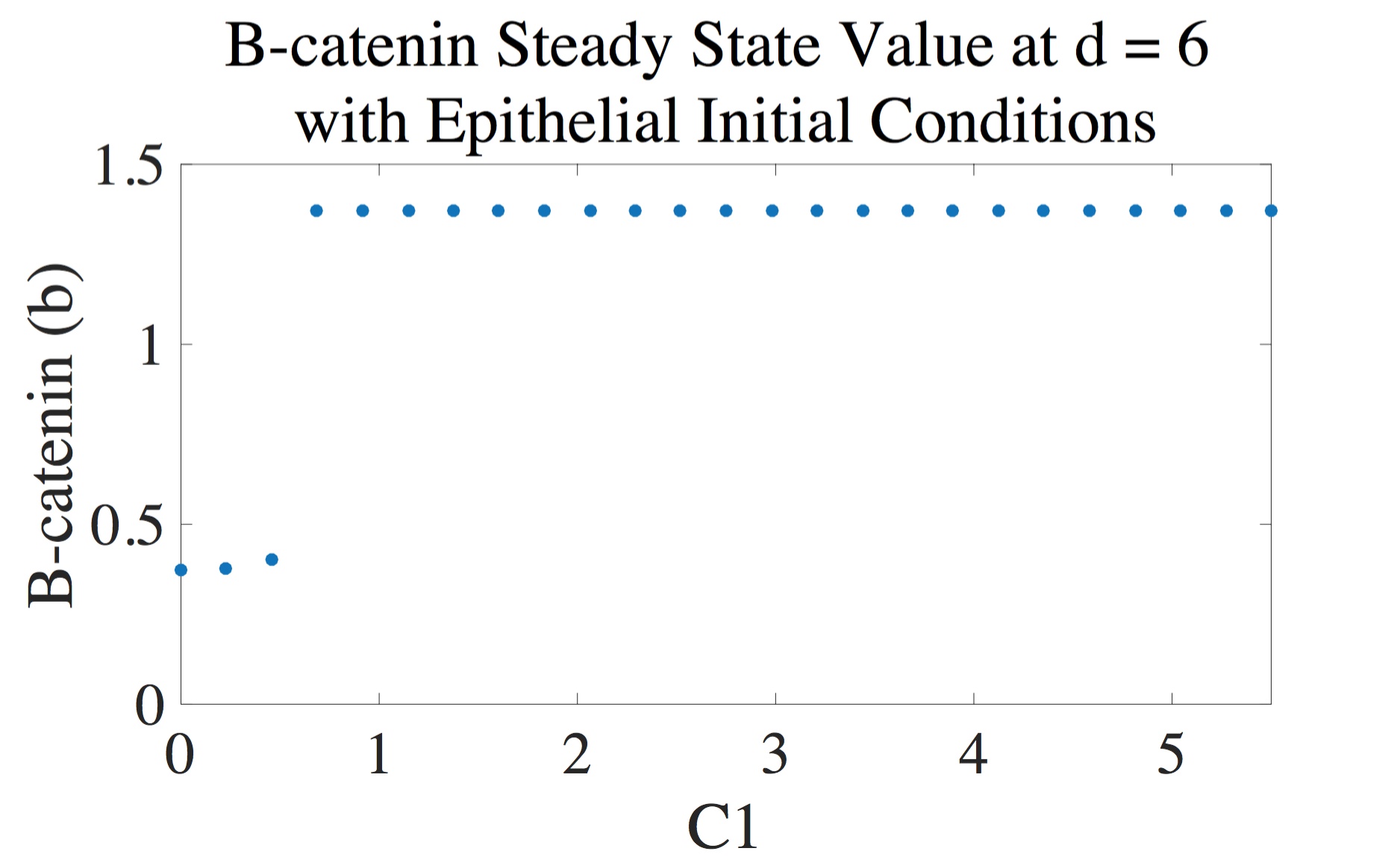 | 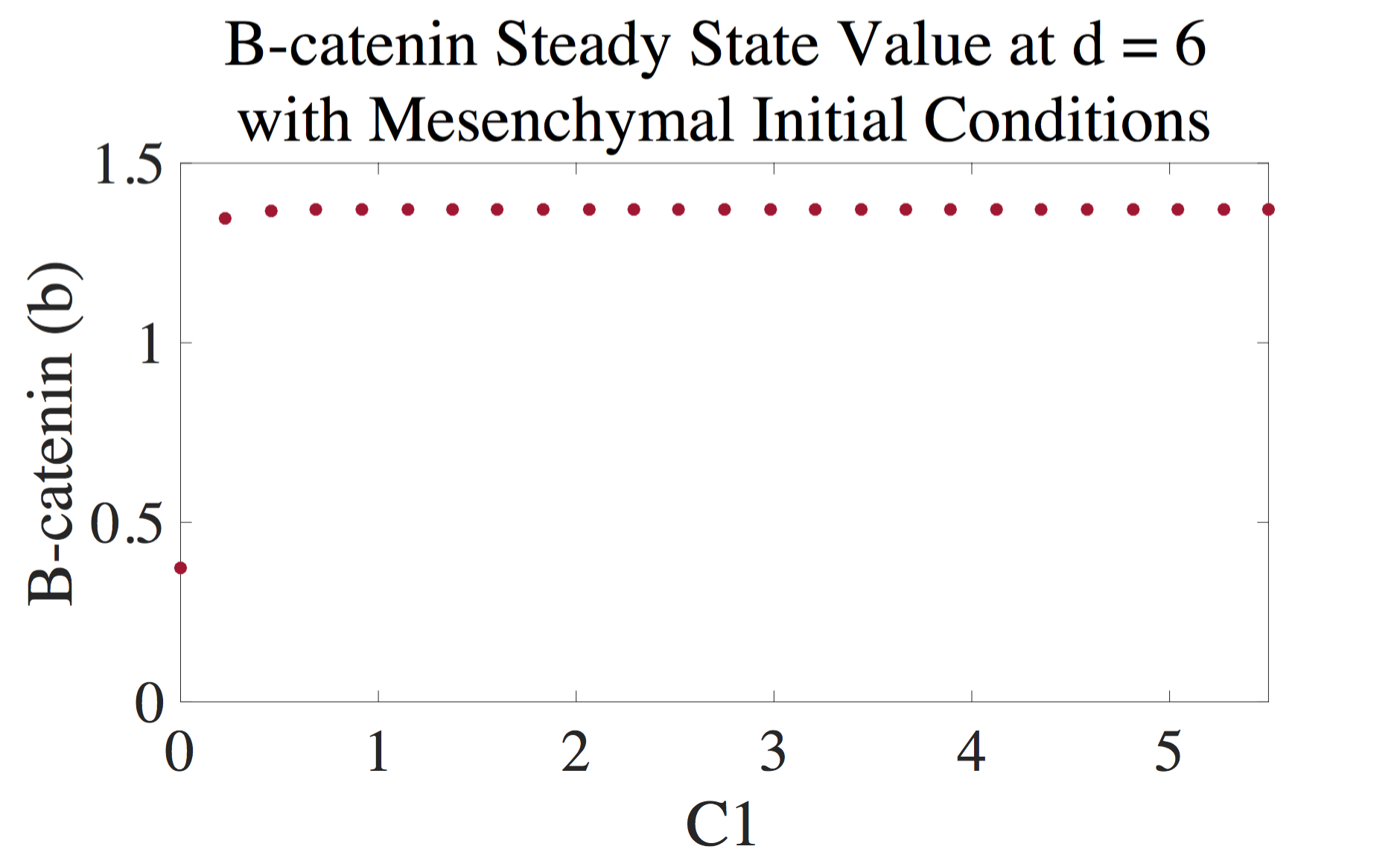 |
| Figure S1BM | Figure S1BN |
|  |  |
| 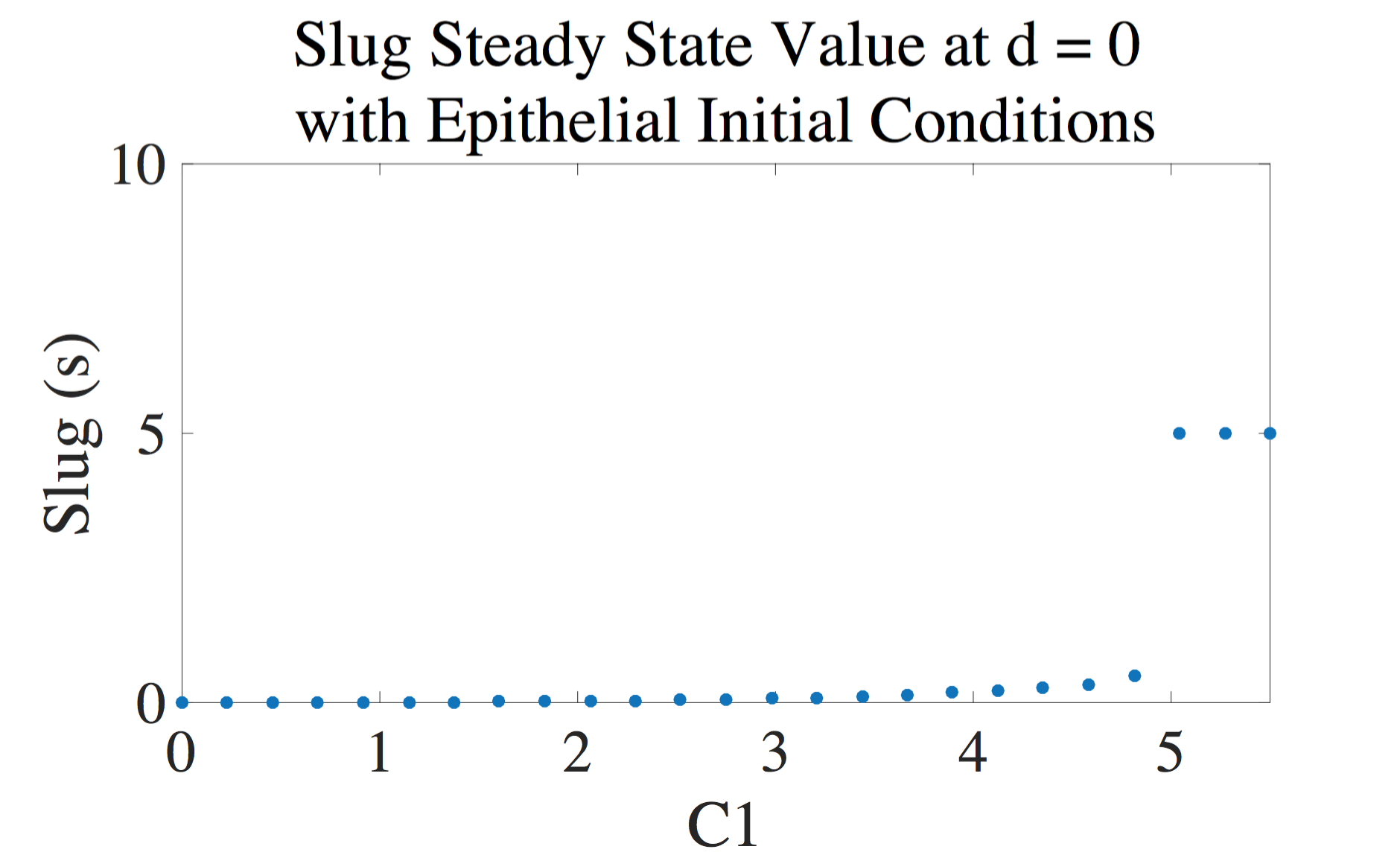 | 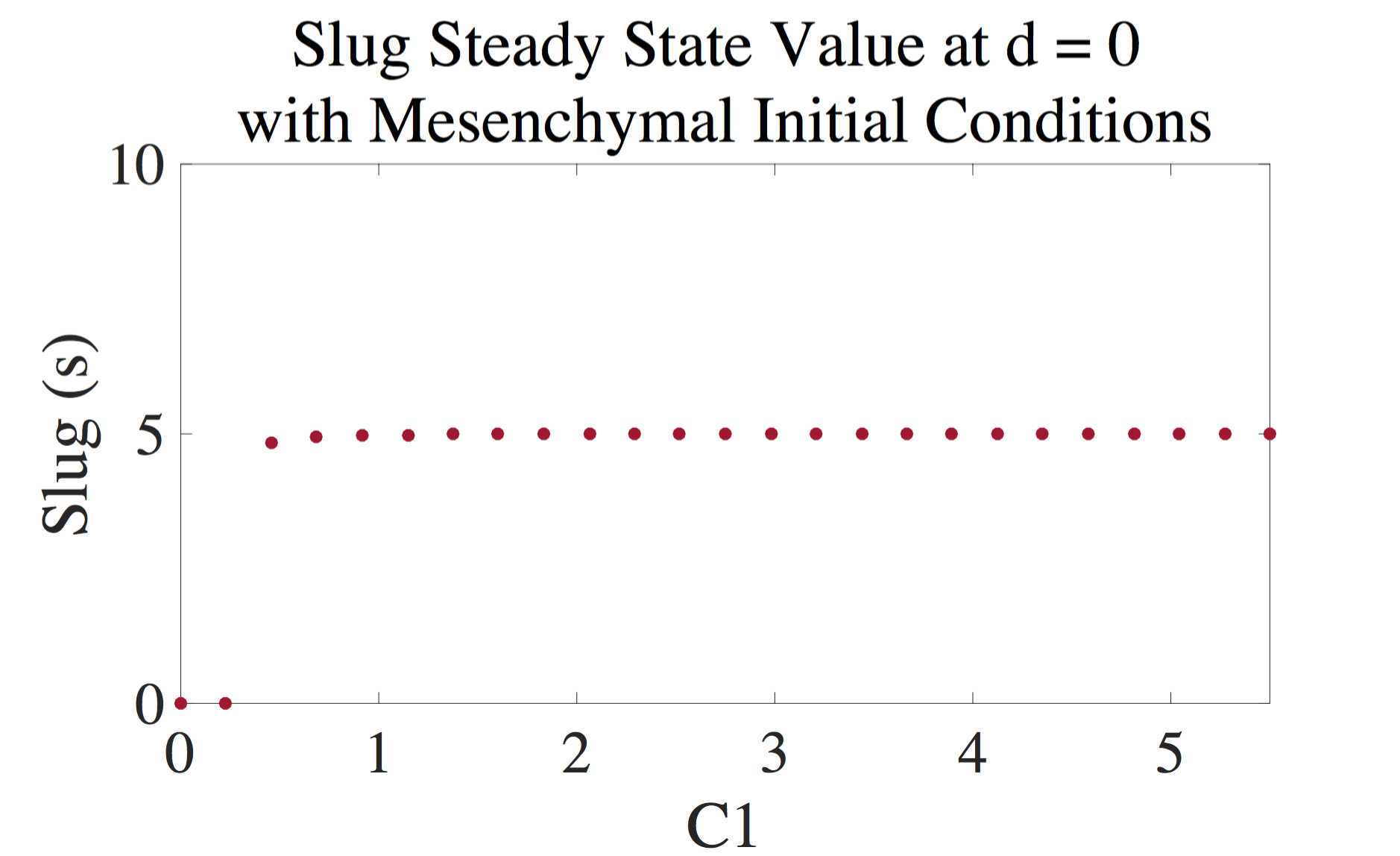 |
| Figure S1BO | Figure S1BP |
|  |  |
| 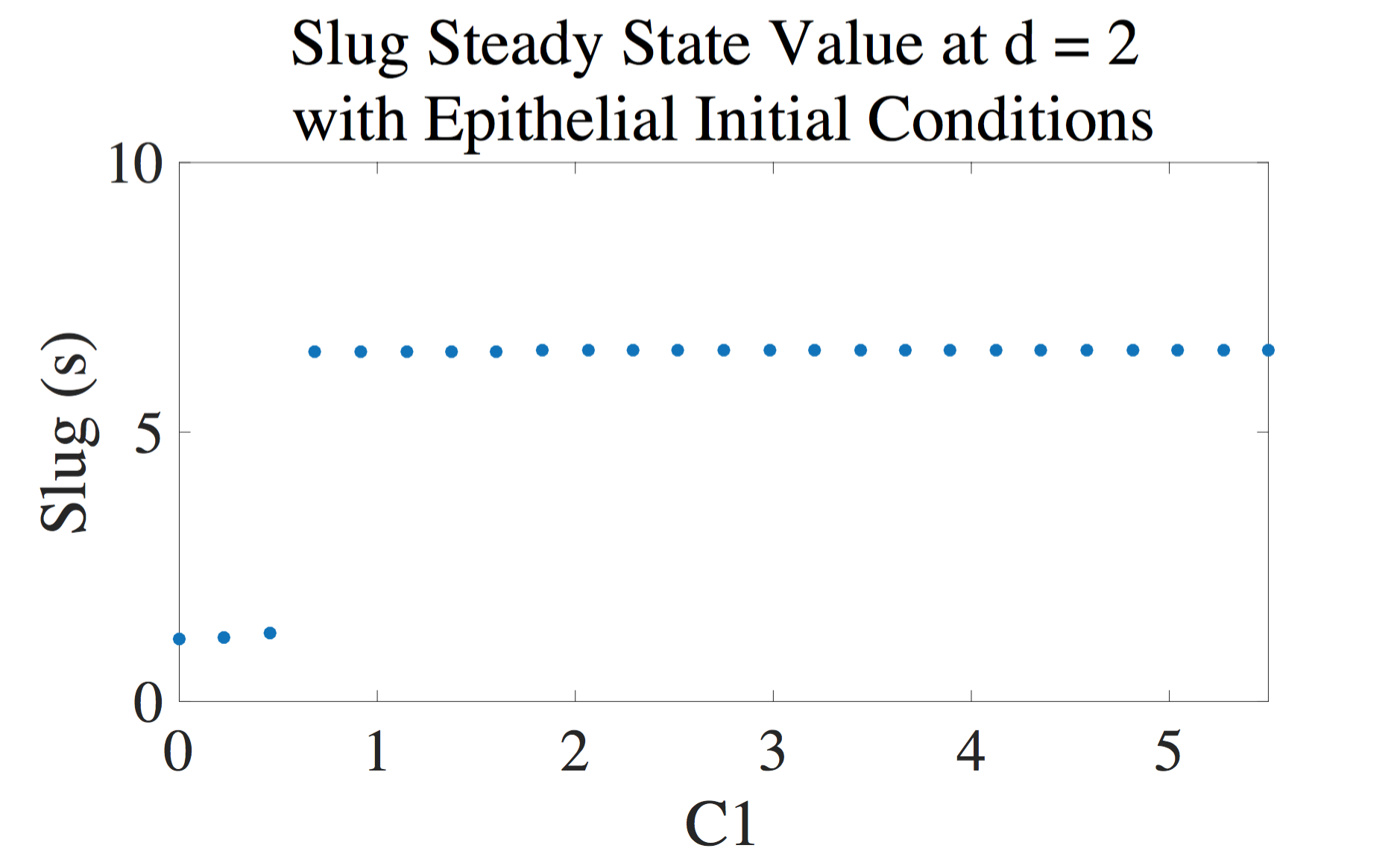 | 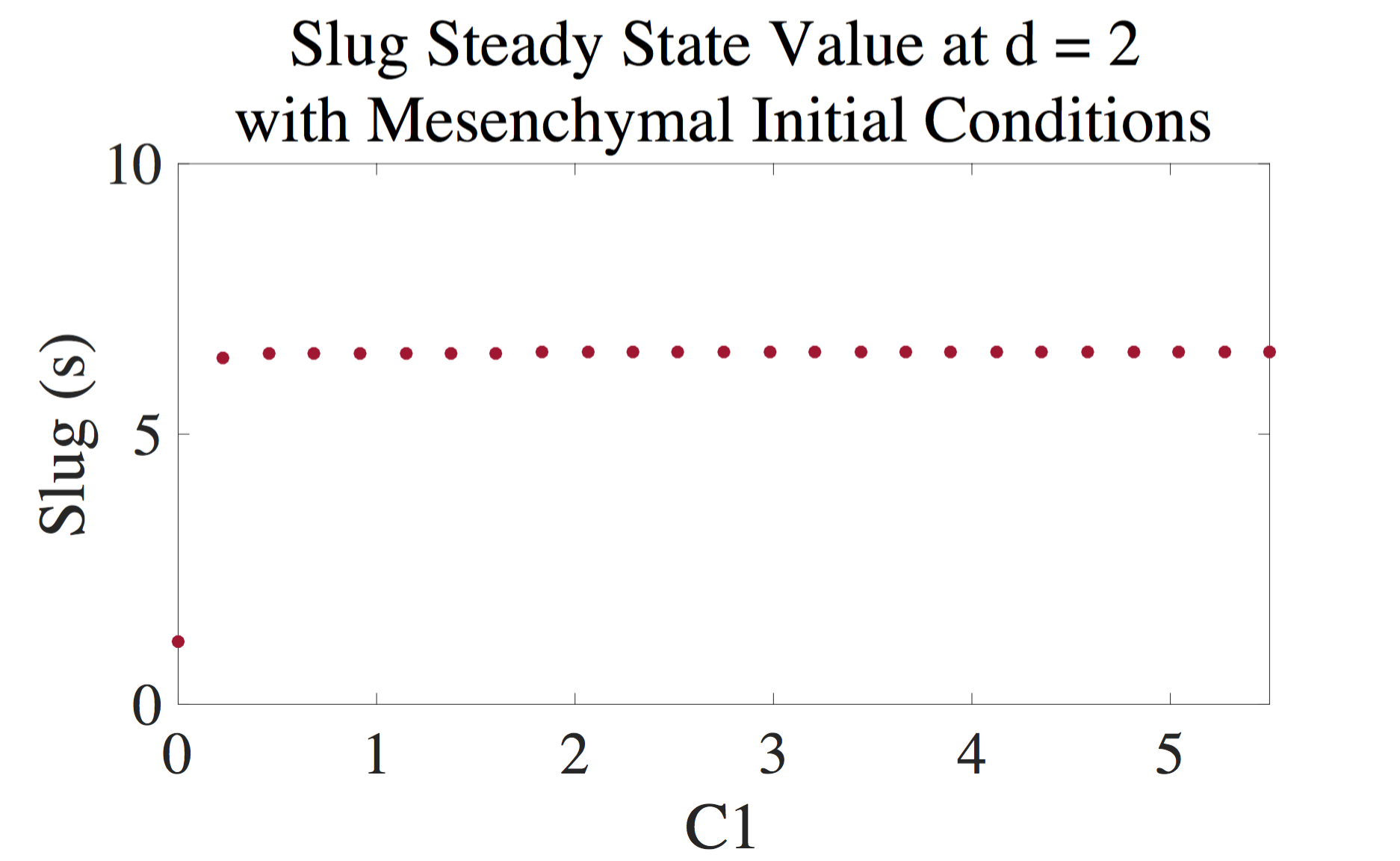 |
| Figure S1BQ | Figure S1BR |
|  |  |
| 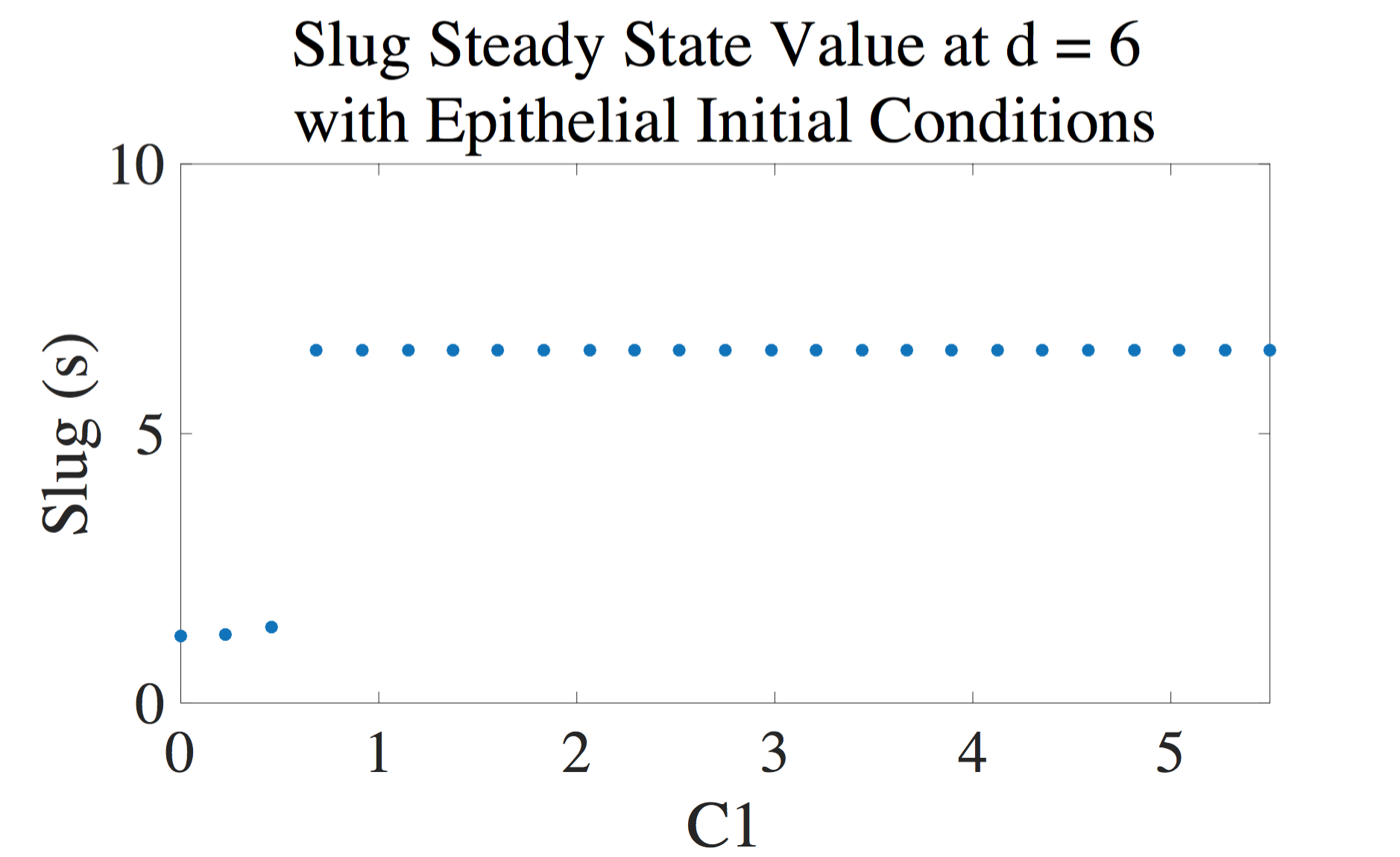 | 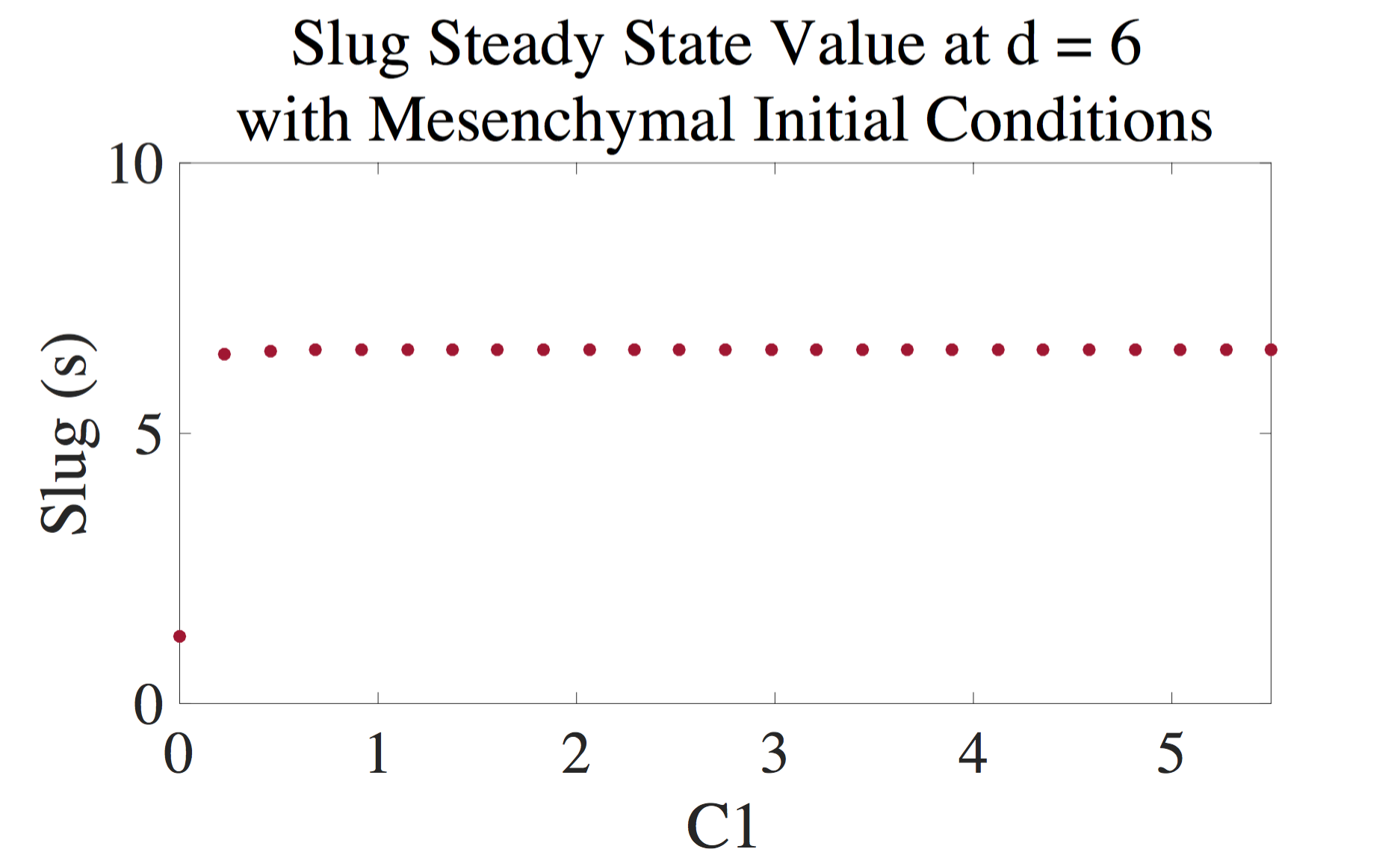 |
| Figure S1BS | Figure S1BT |
|  |  |
| 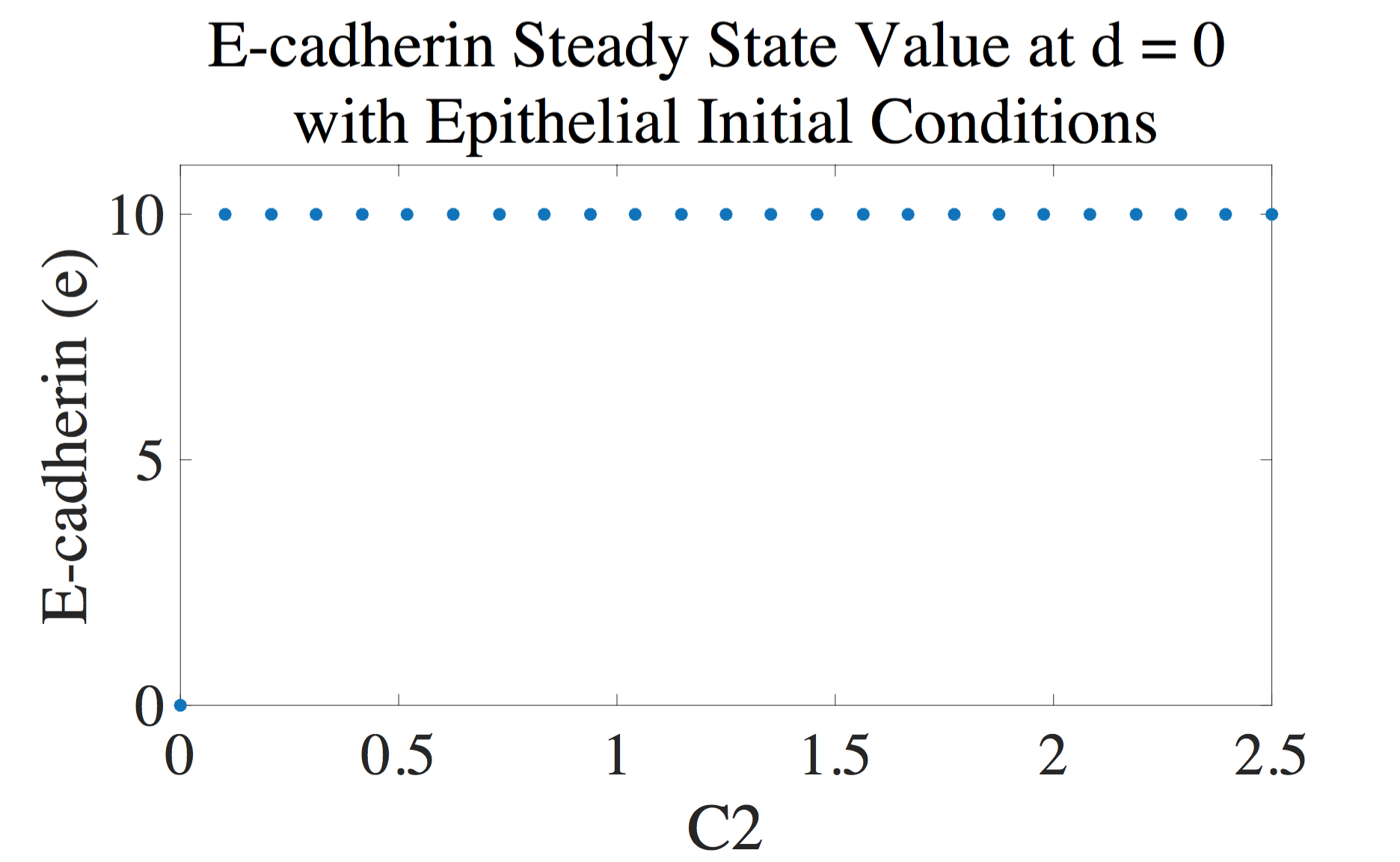 | 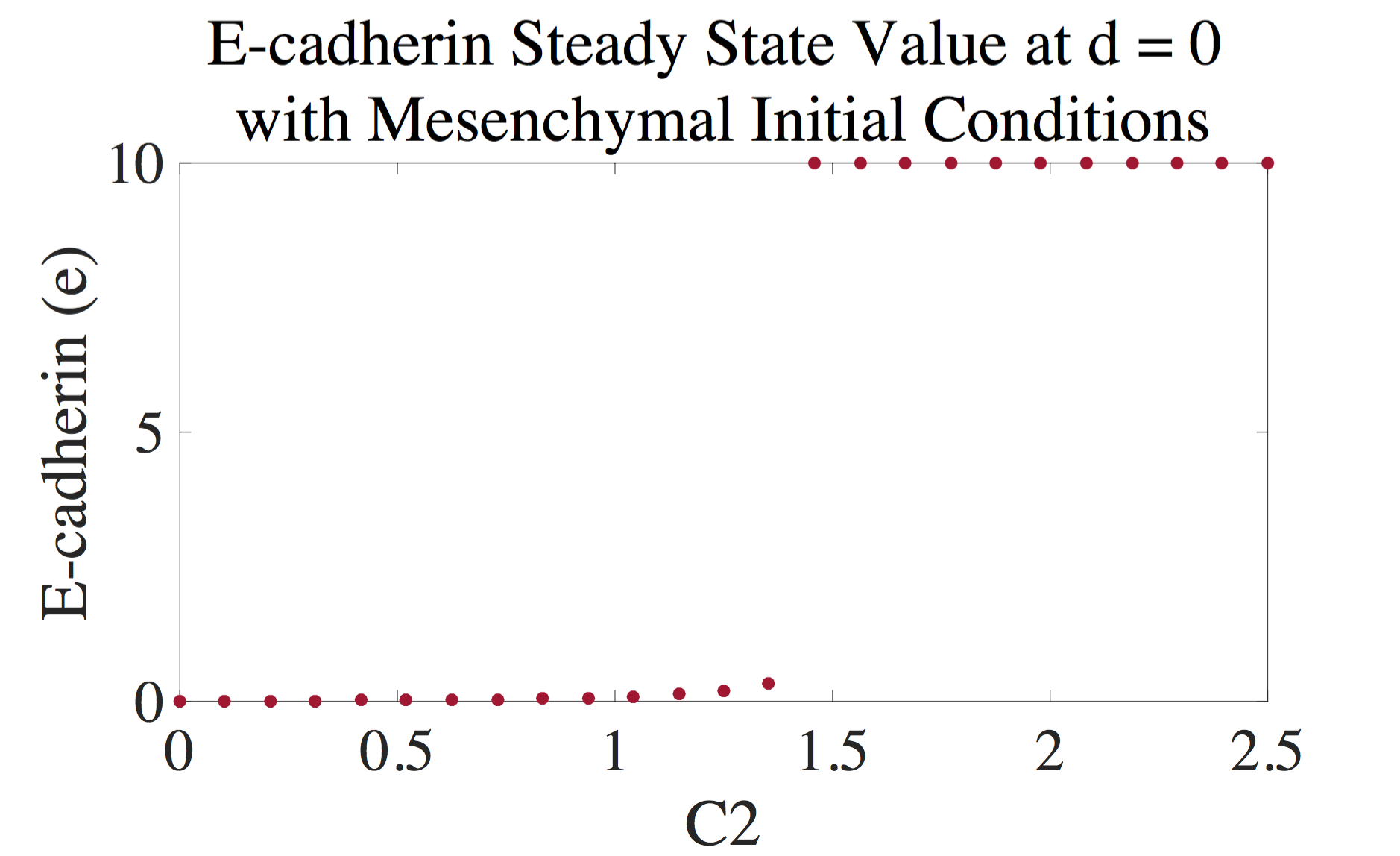 |
| Figure S1BU | Figure S1BV |
|  |  |
| 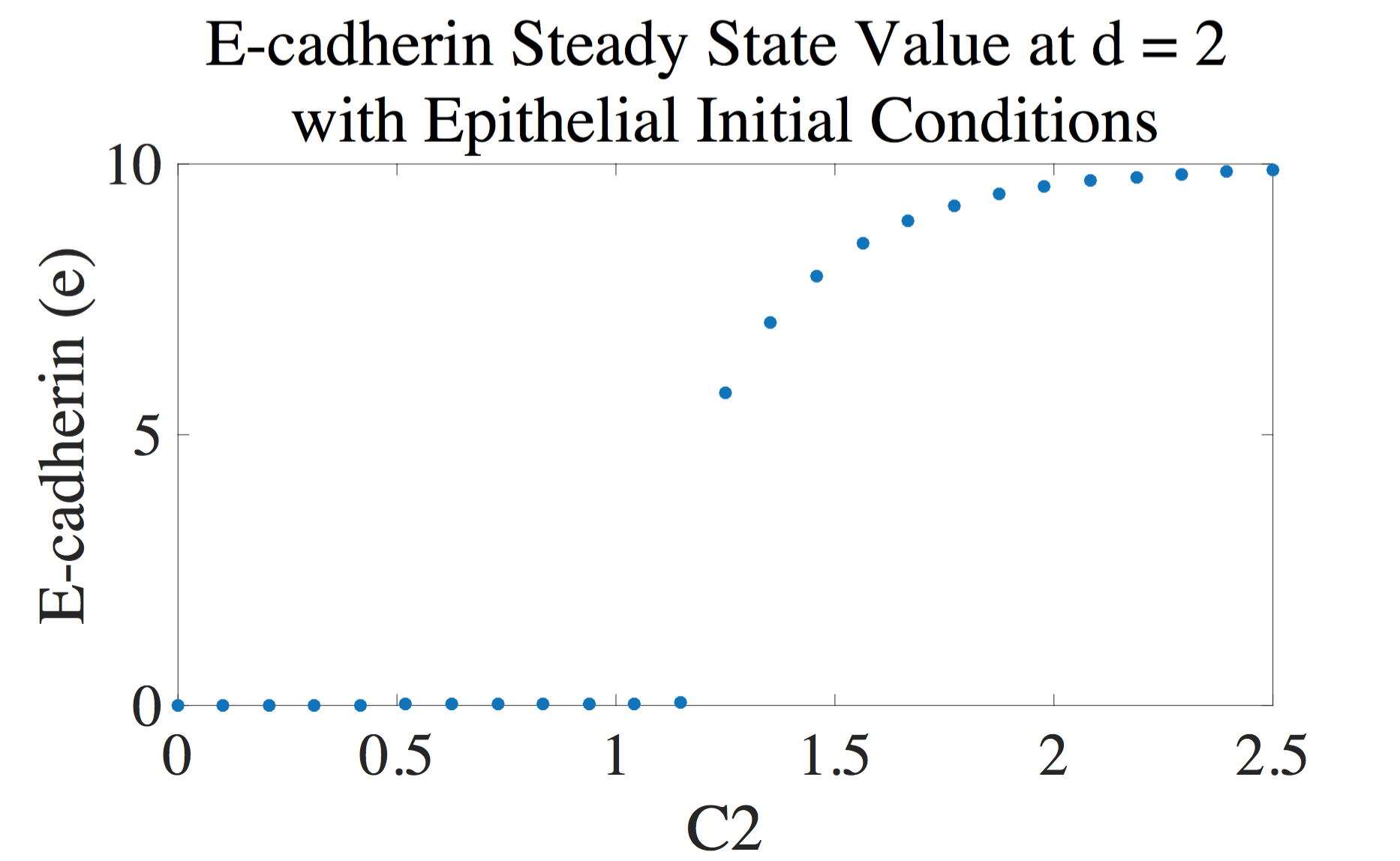 | 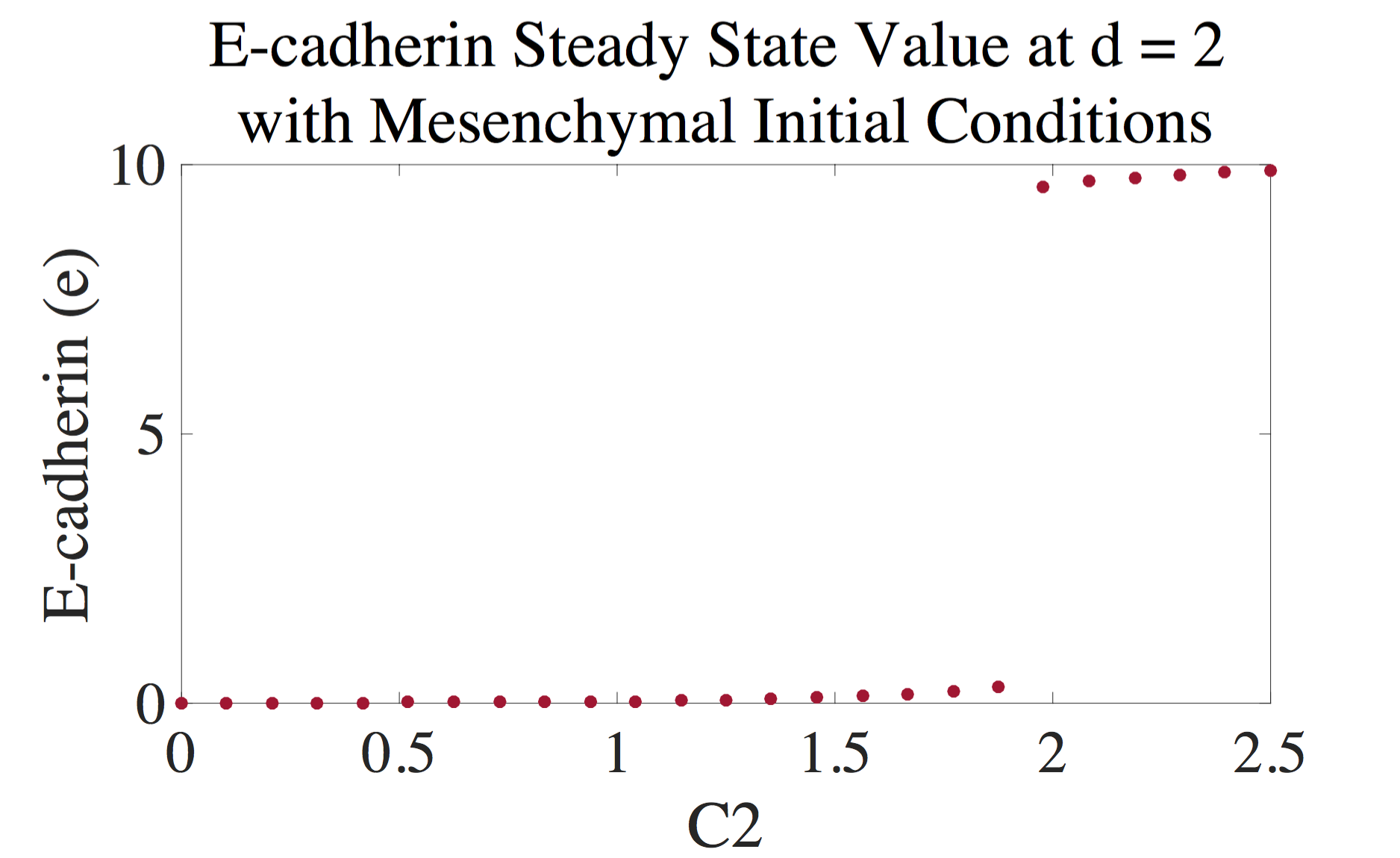 |
| Figure S1BW | Figure S1BX |
|  |  |
| 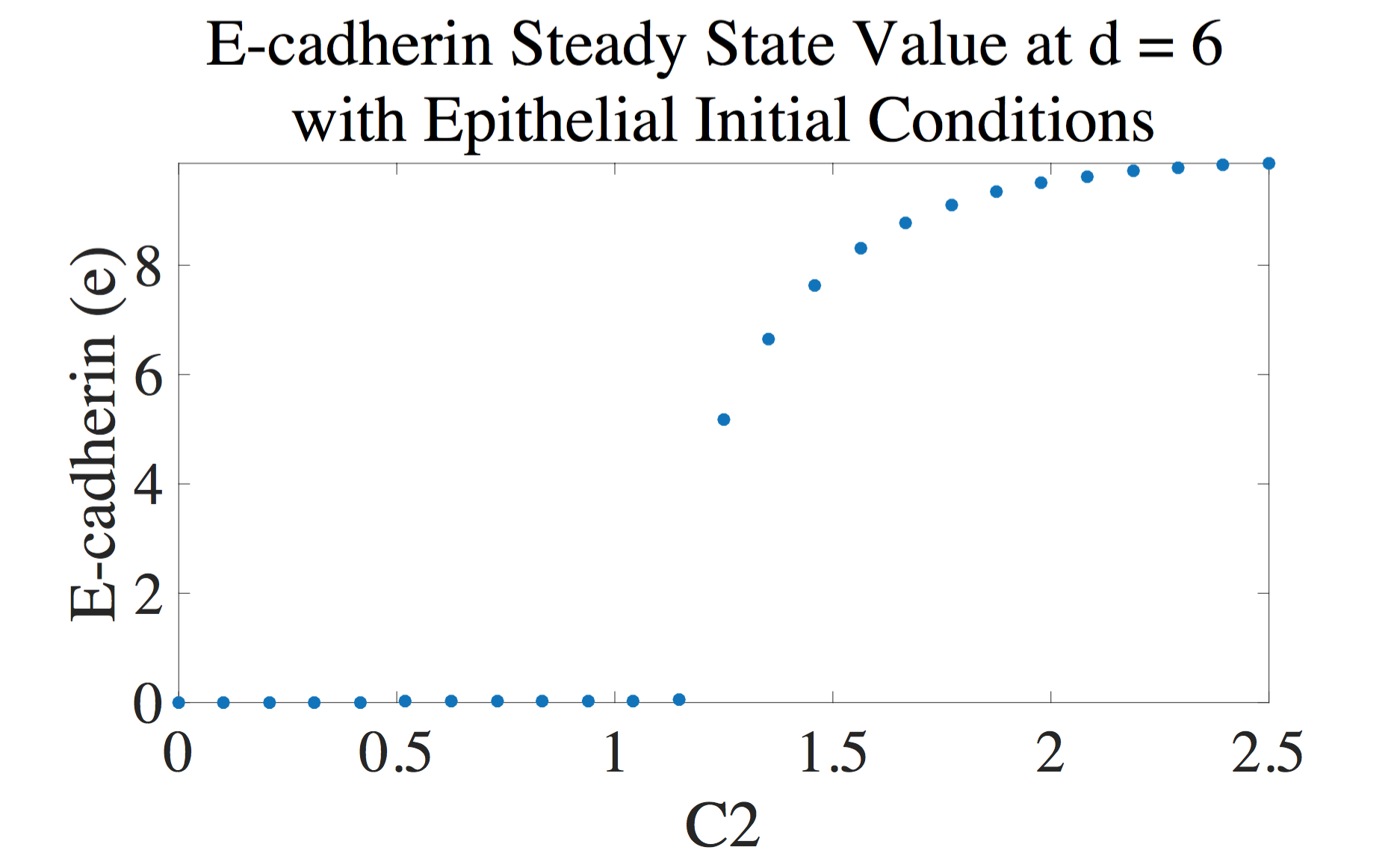 | 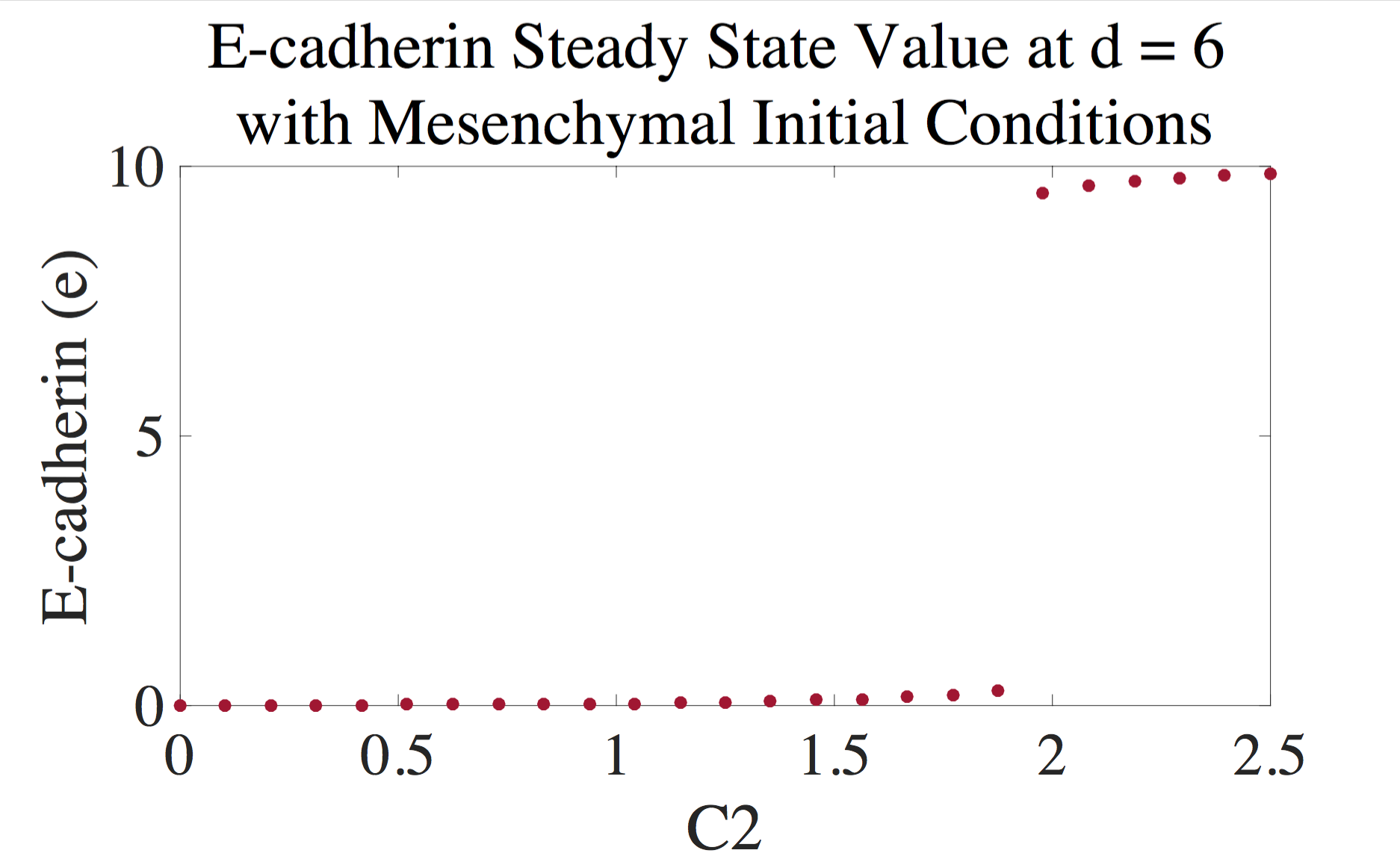 |
| Figure S1BY | Figure S1BZ |
|  |  |
| 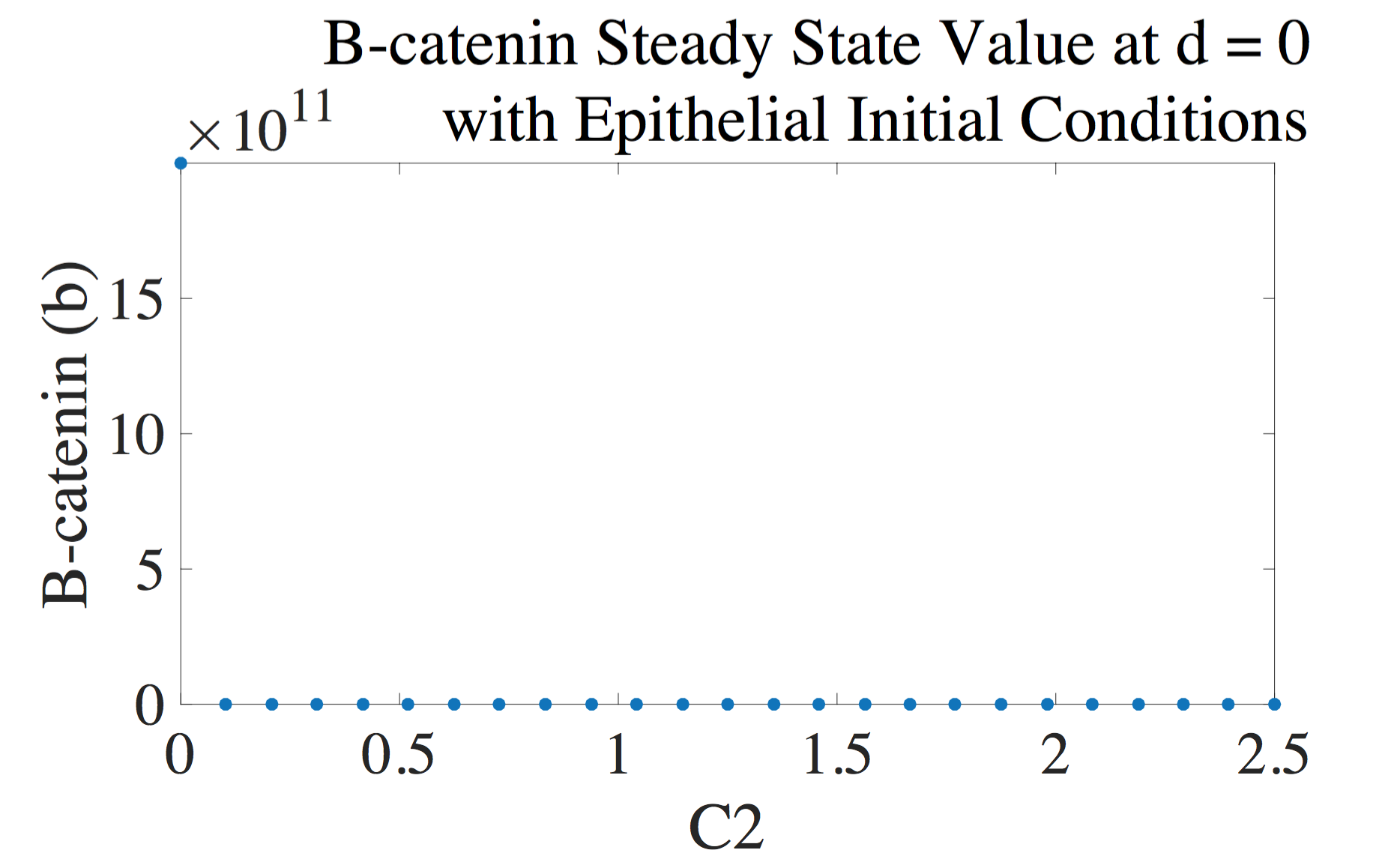 | 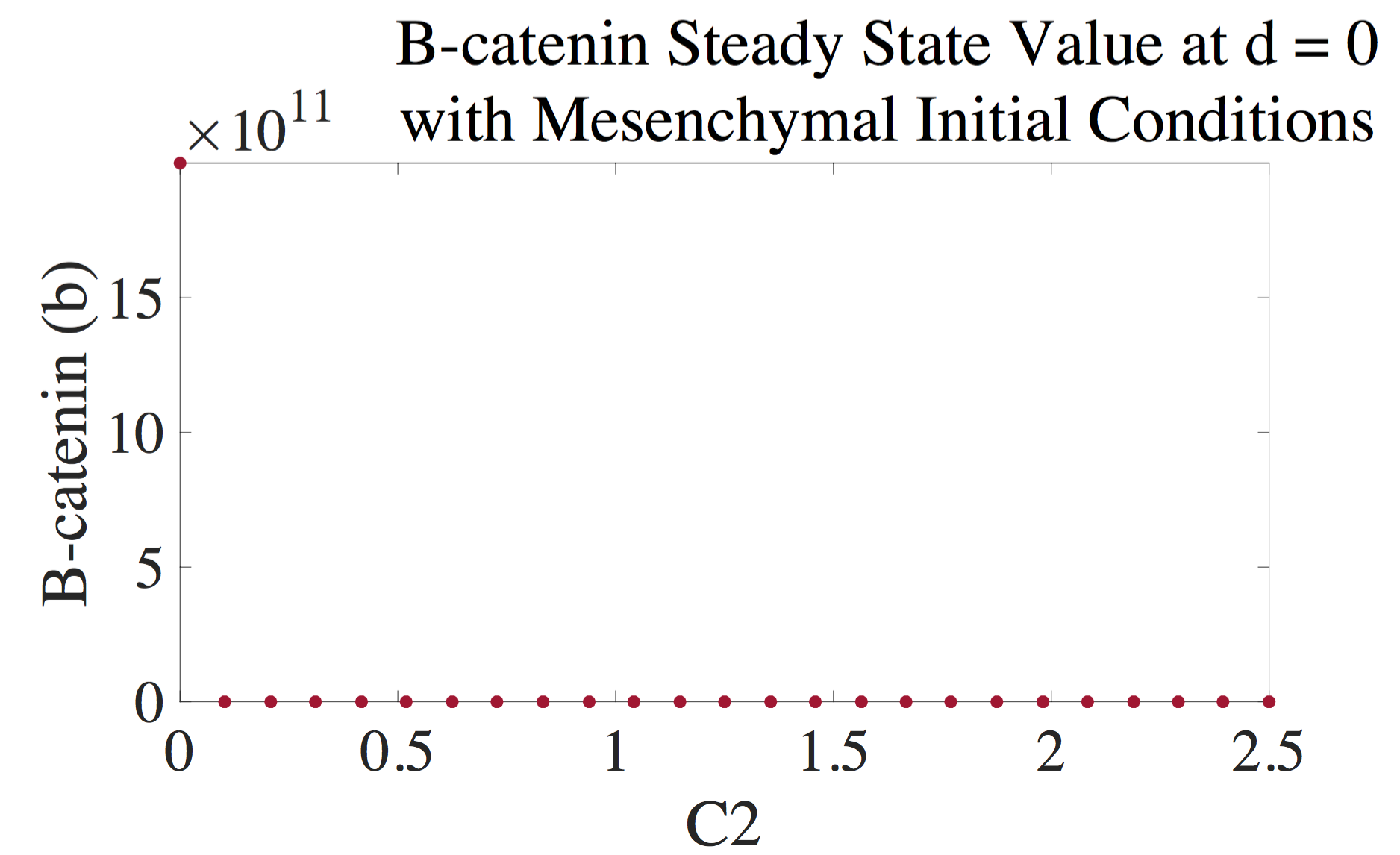 |
| Figure S1CA | Figure S1CB |
|  |  |
| 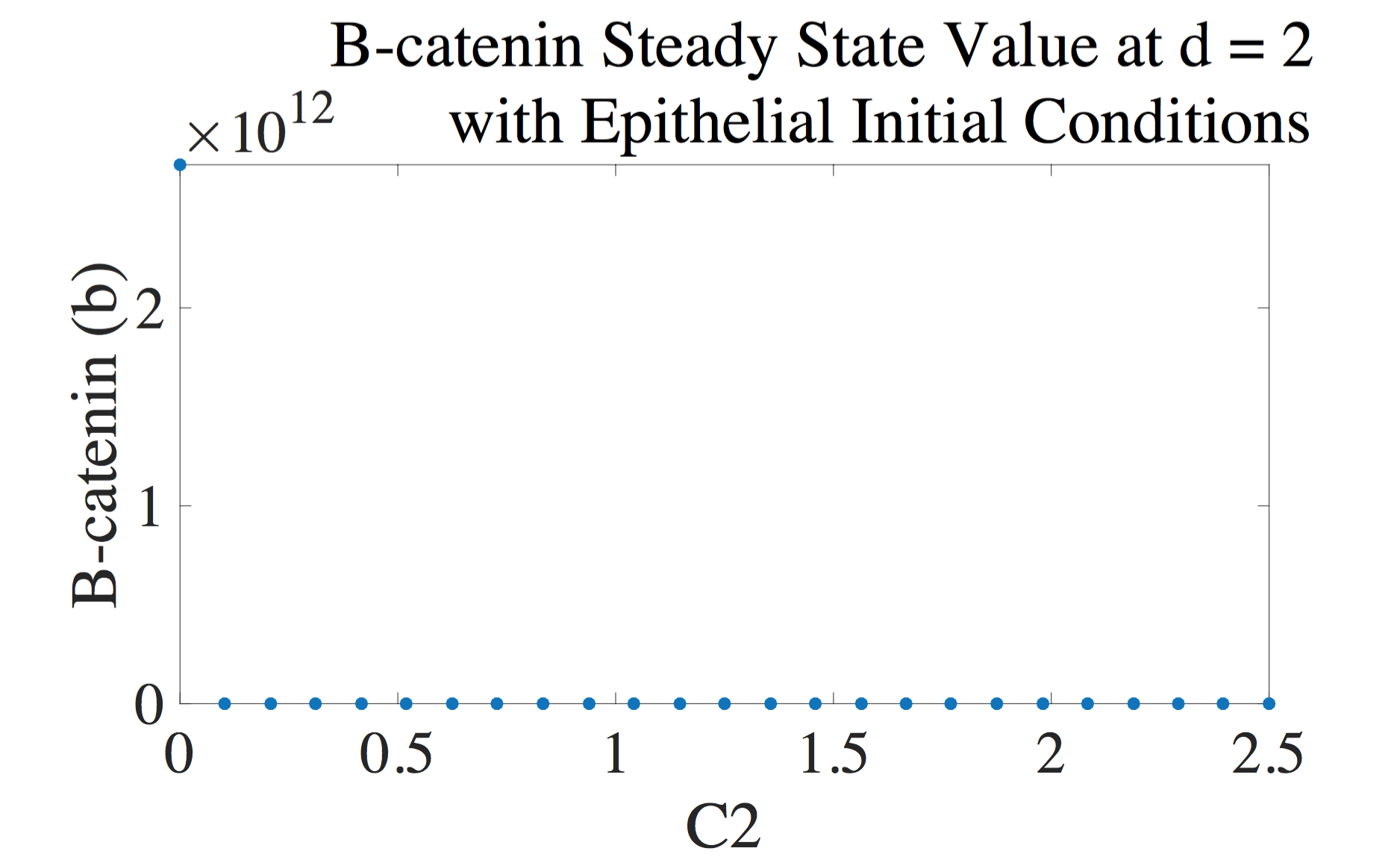 | 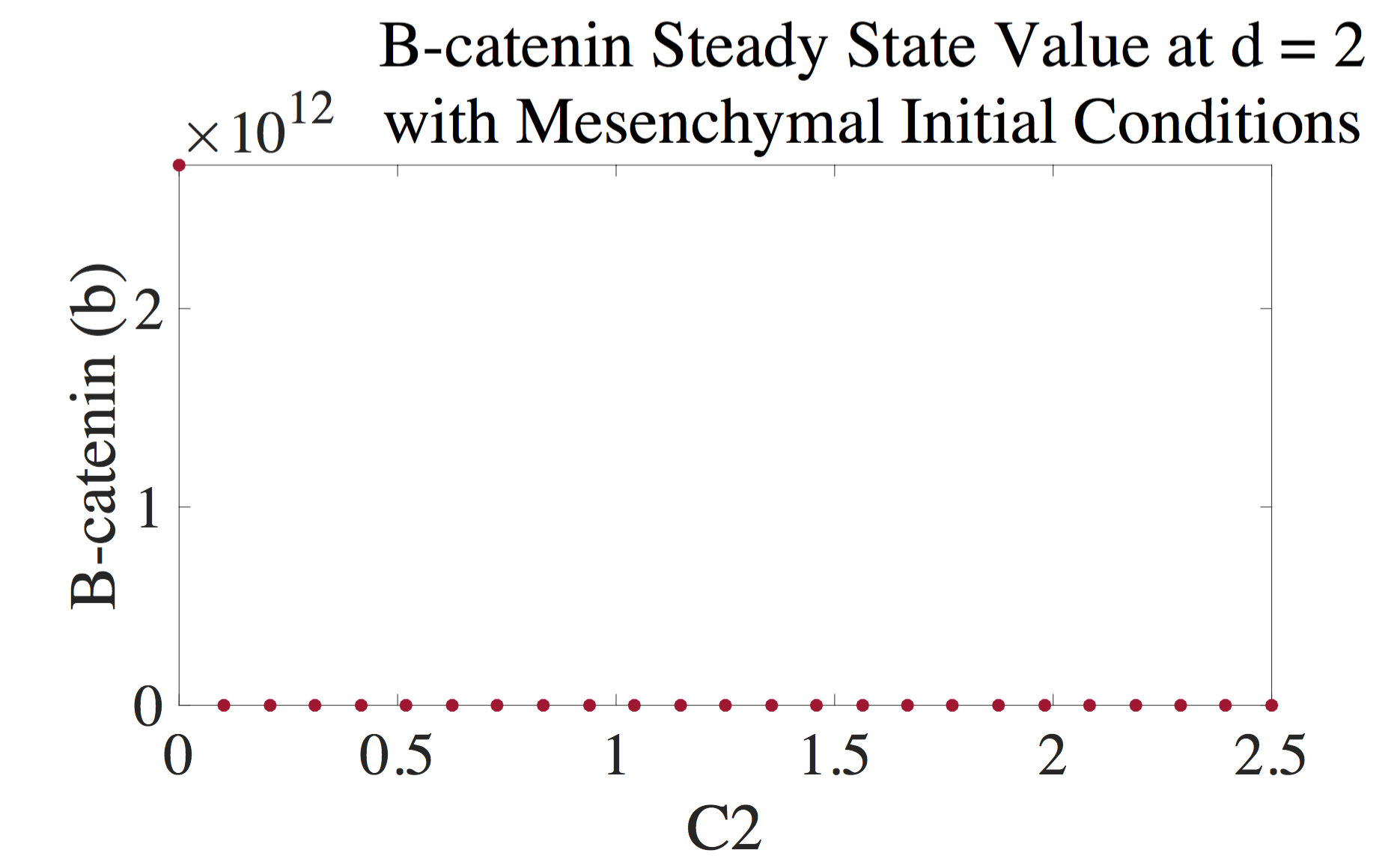 |
| Figure S1CC | Figure S1CD |
|  |  |
| 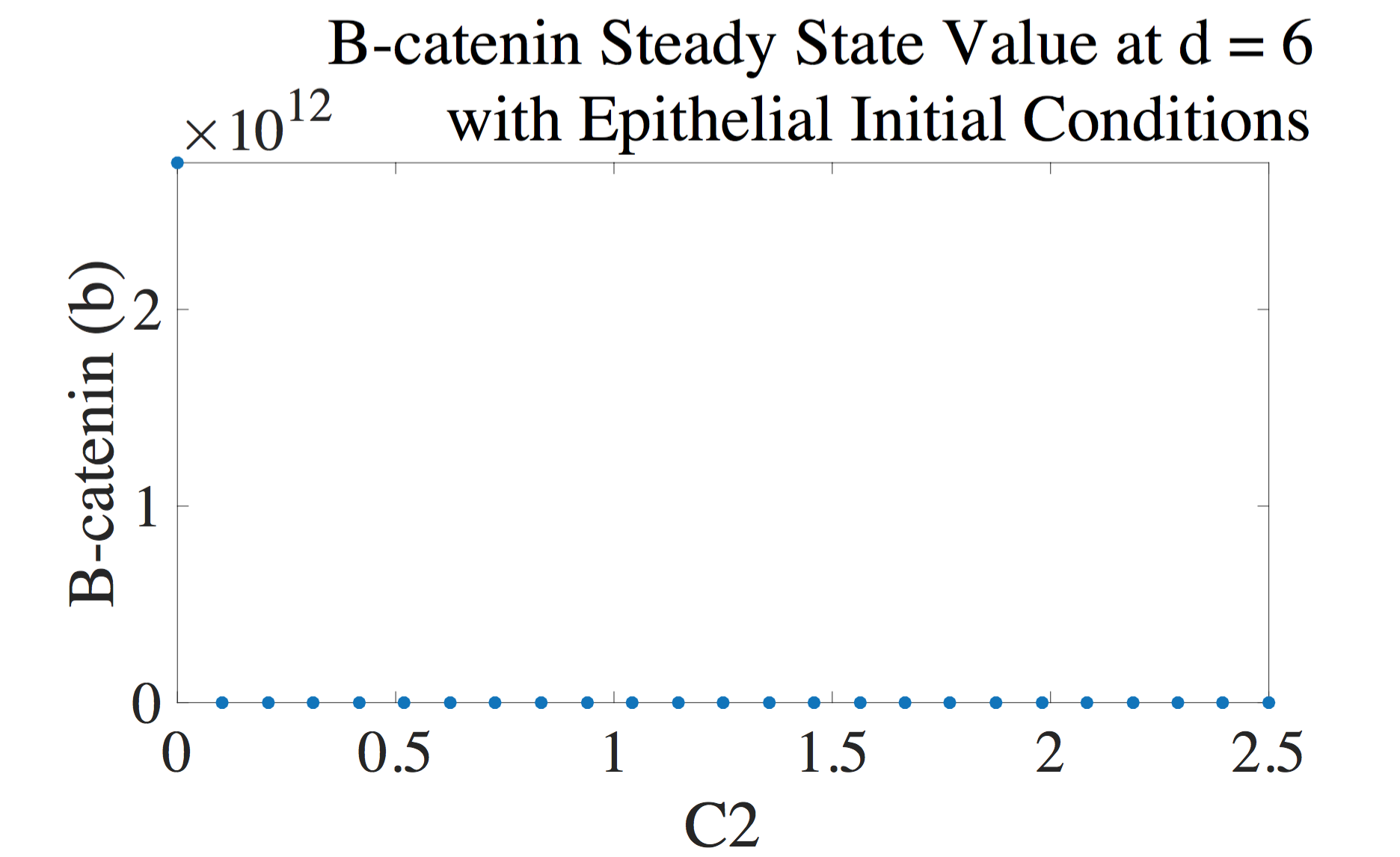 | 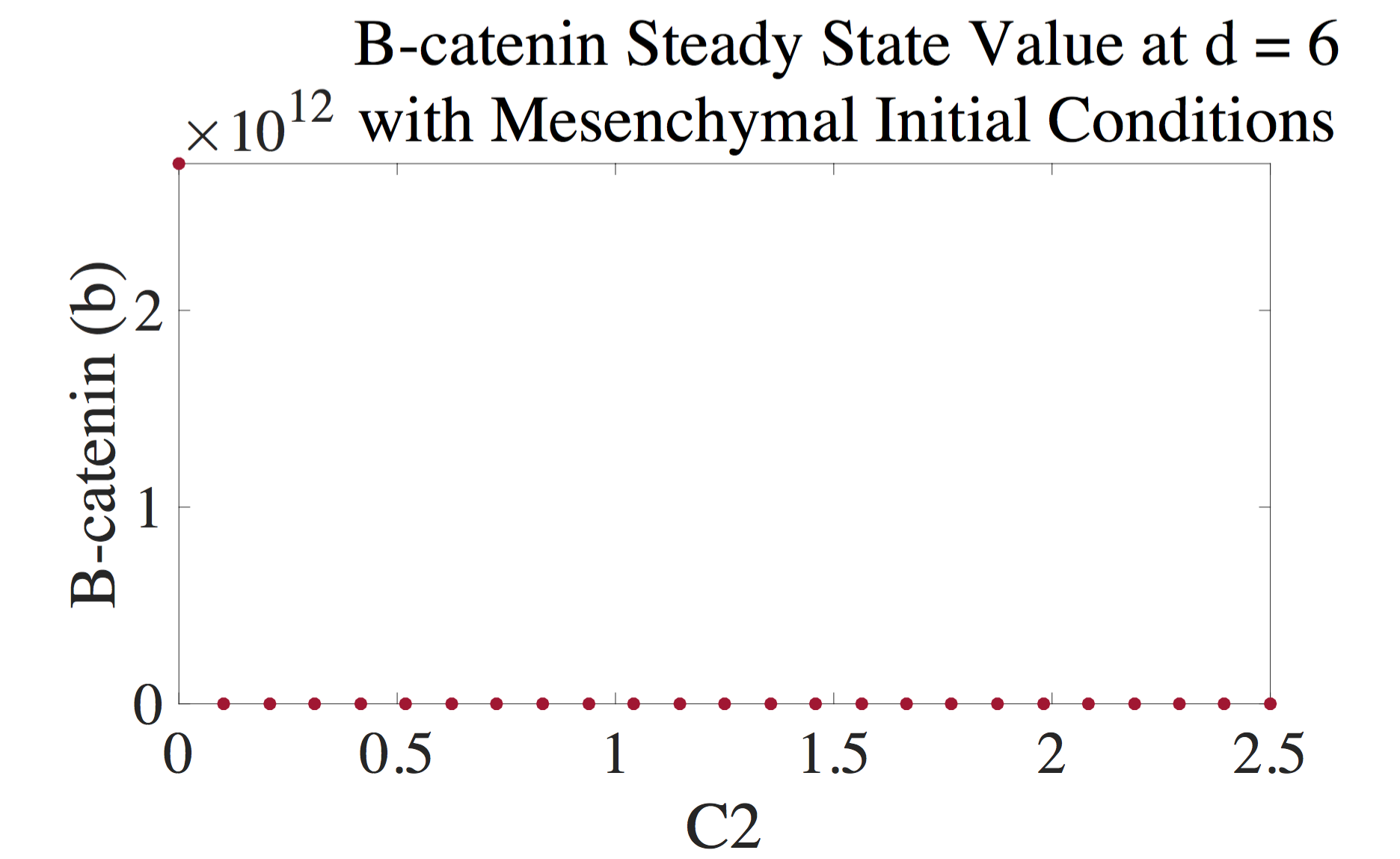 |
| Figure S1CE | Figure S1CF |
|  |  |
| 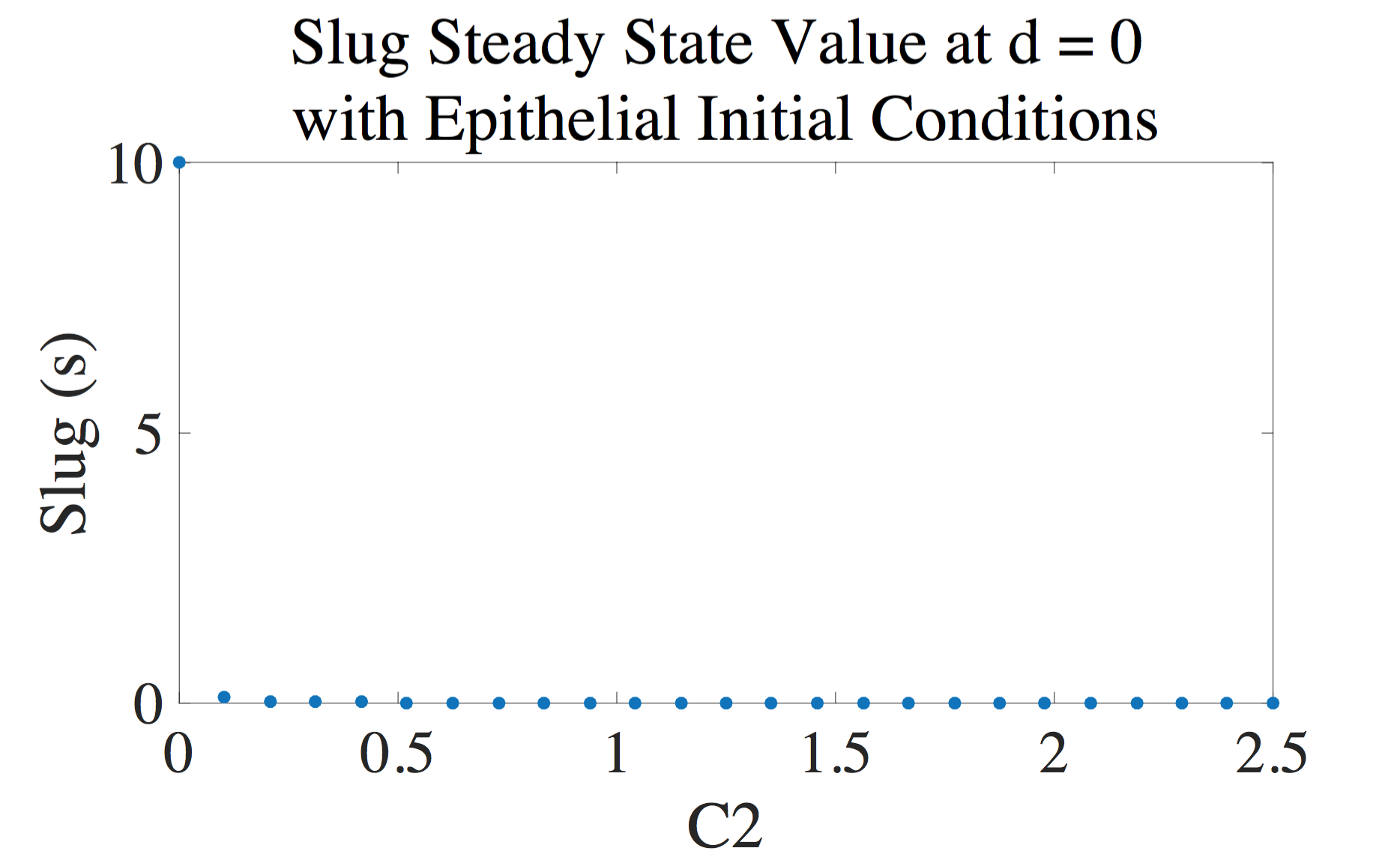 | 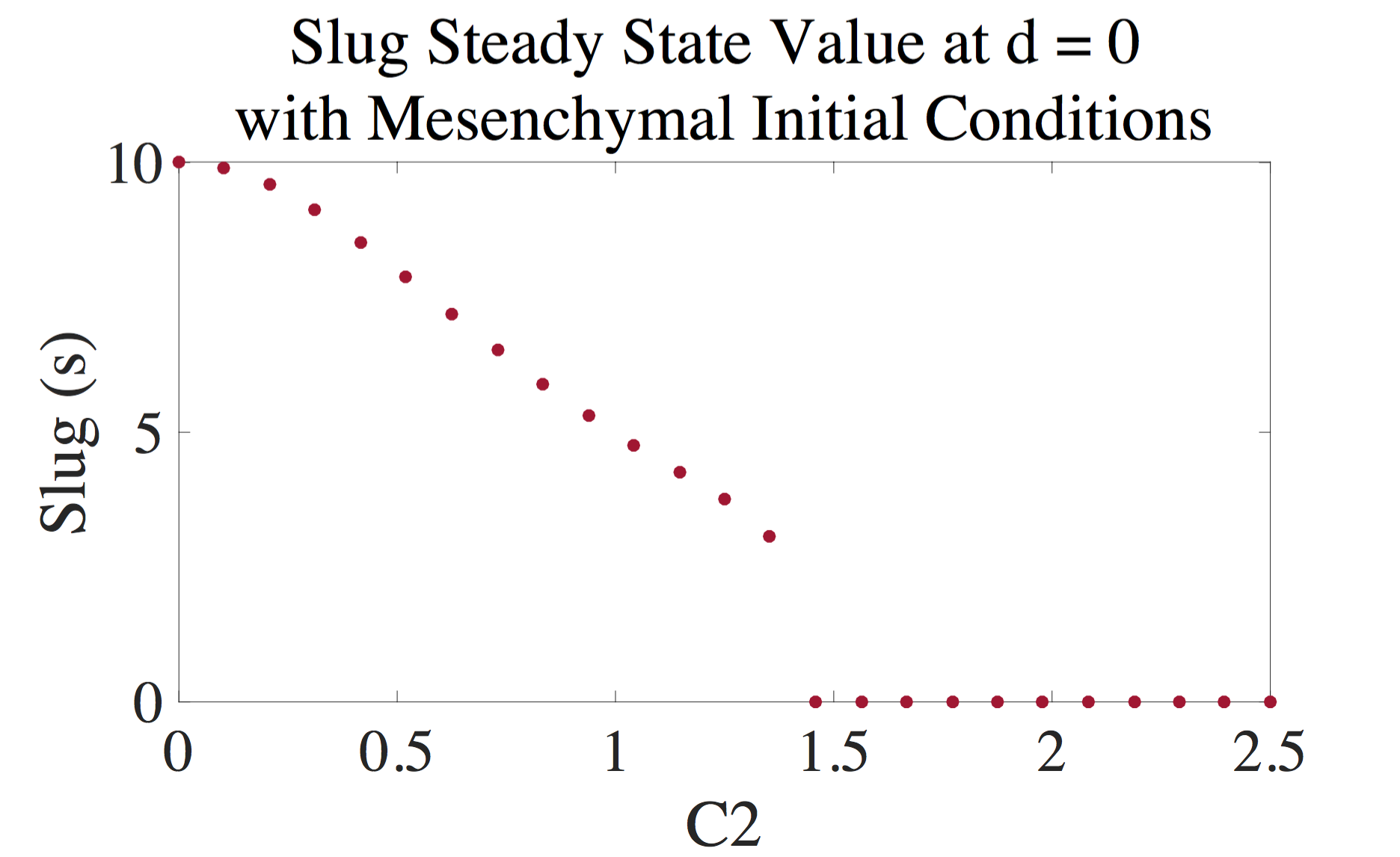 |
| Figure S1CG | Figure S1CH |
|  |  |
| 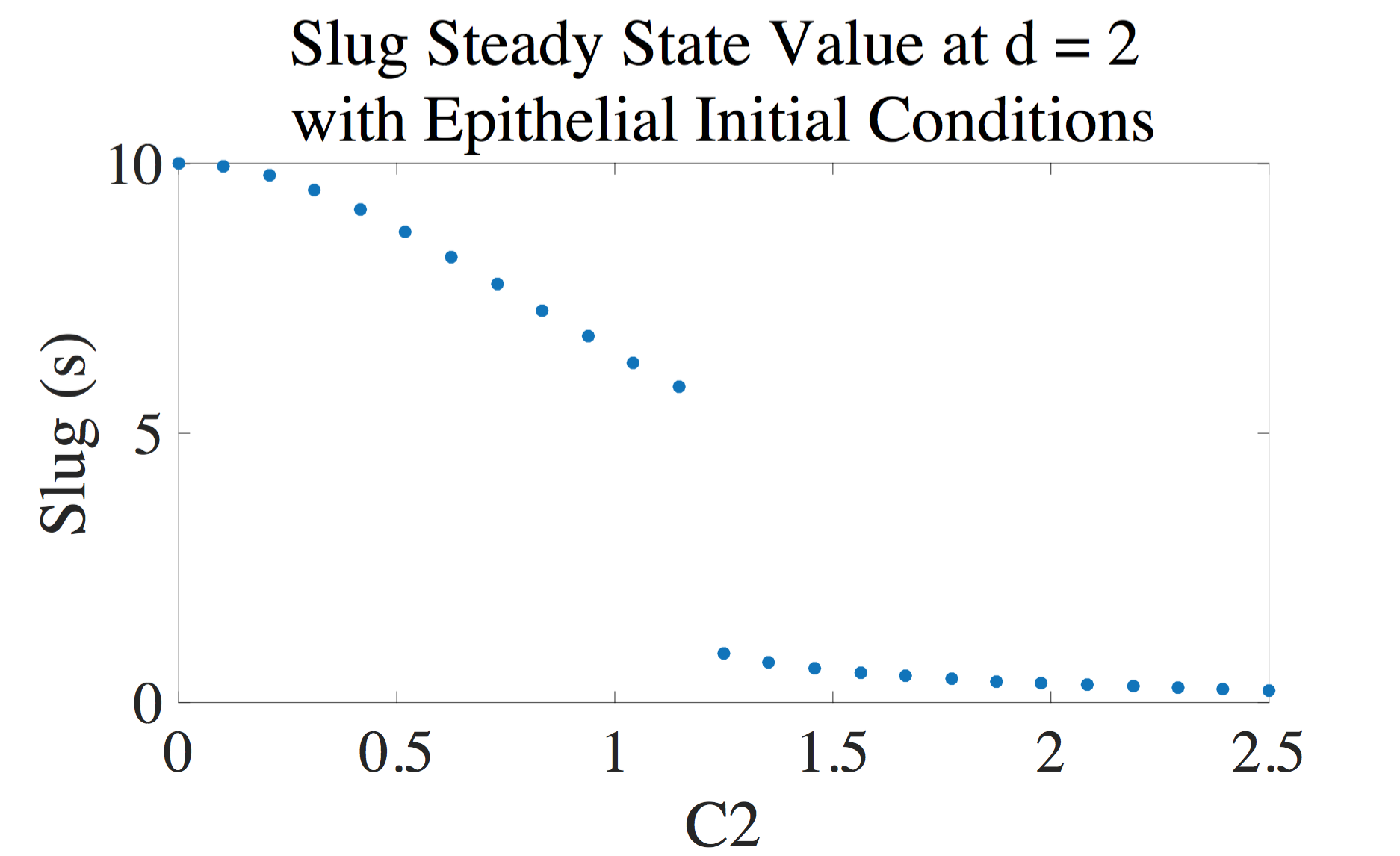 | 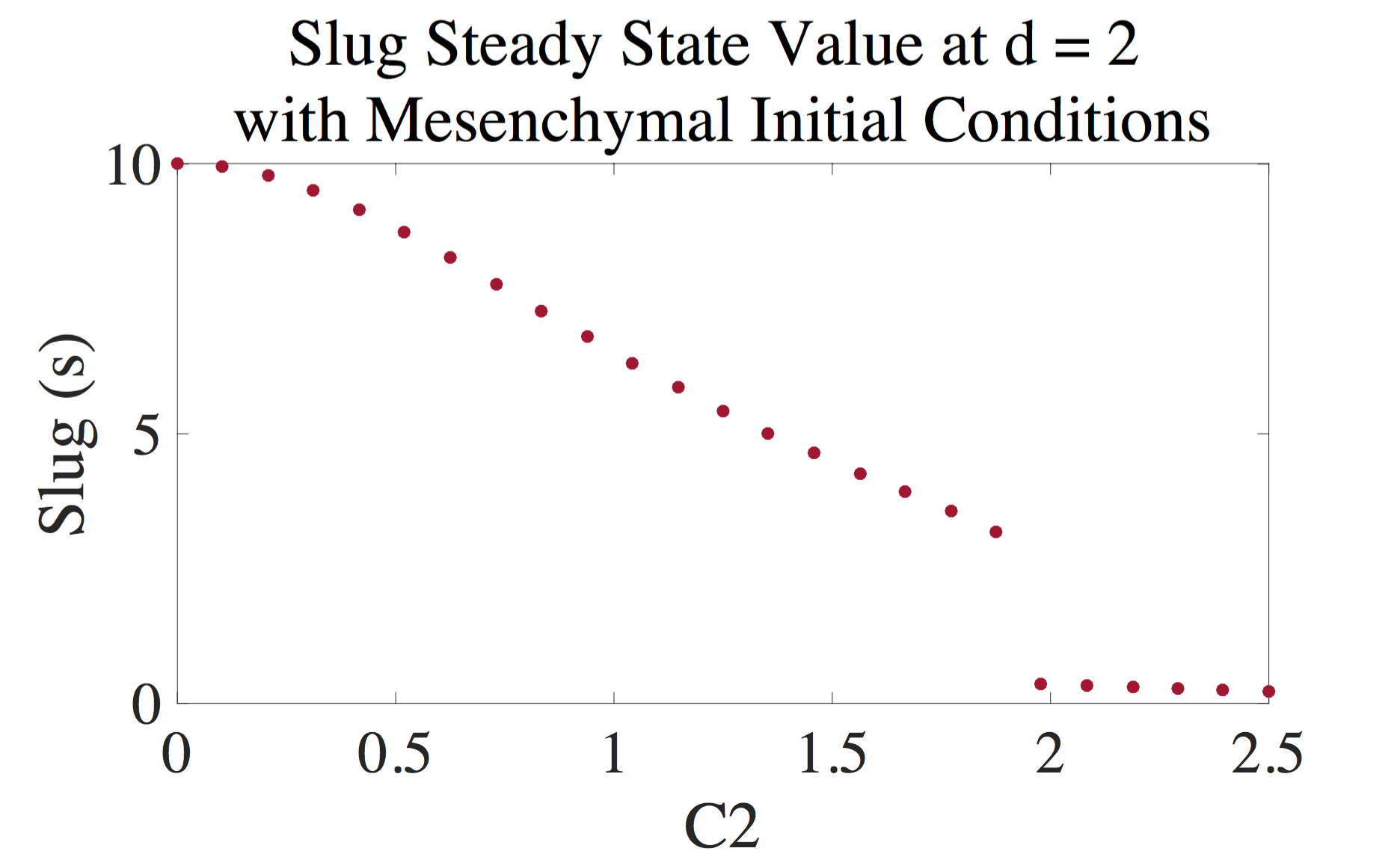 |
| Figure S1CI | Figure S1CJ |
|  |  |
| 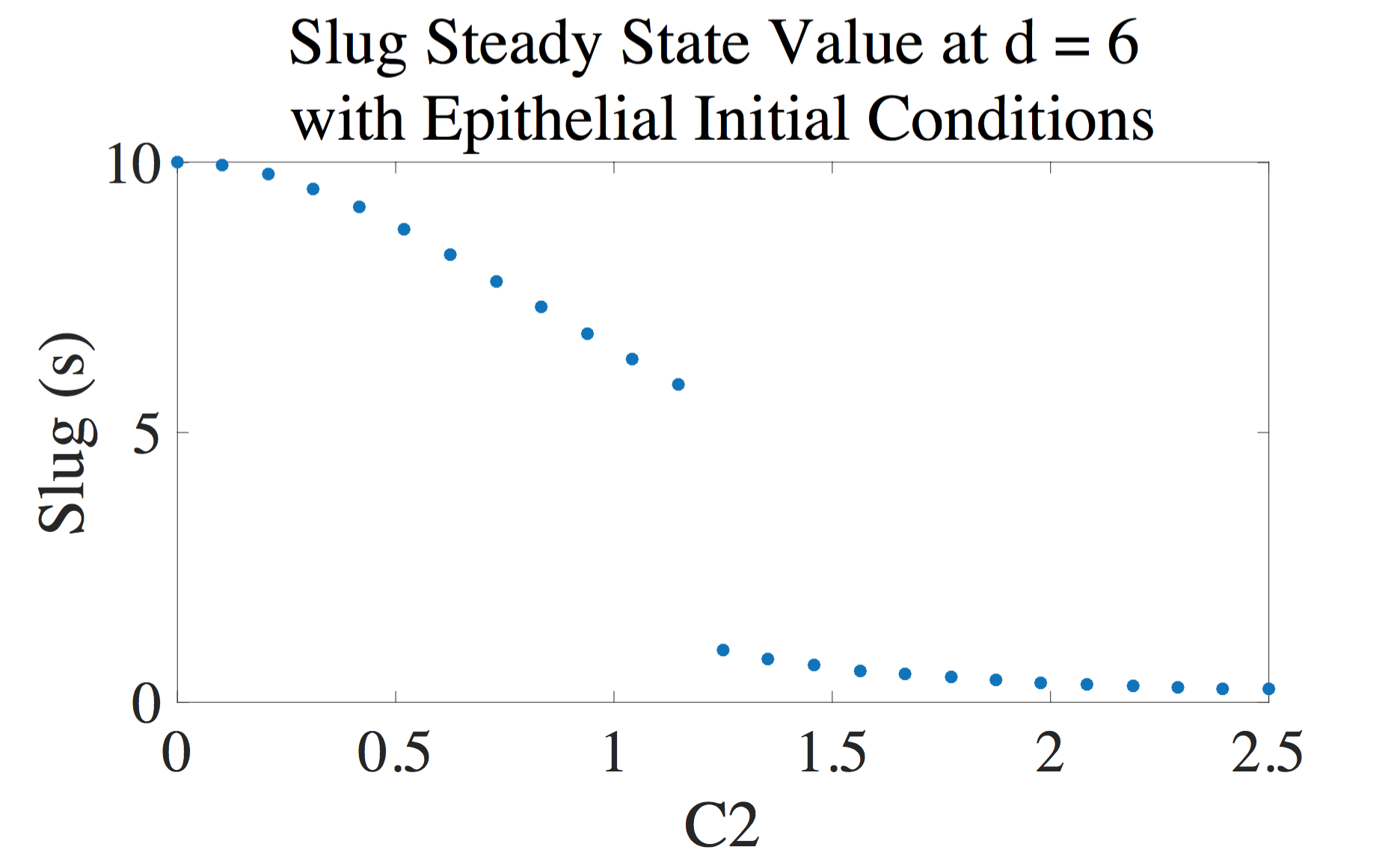 | 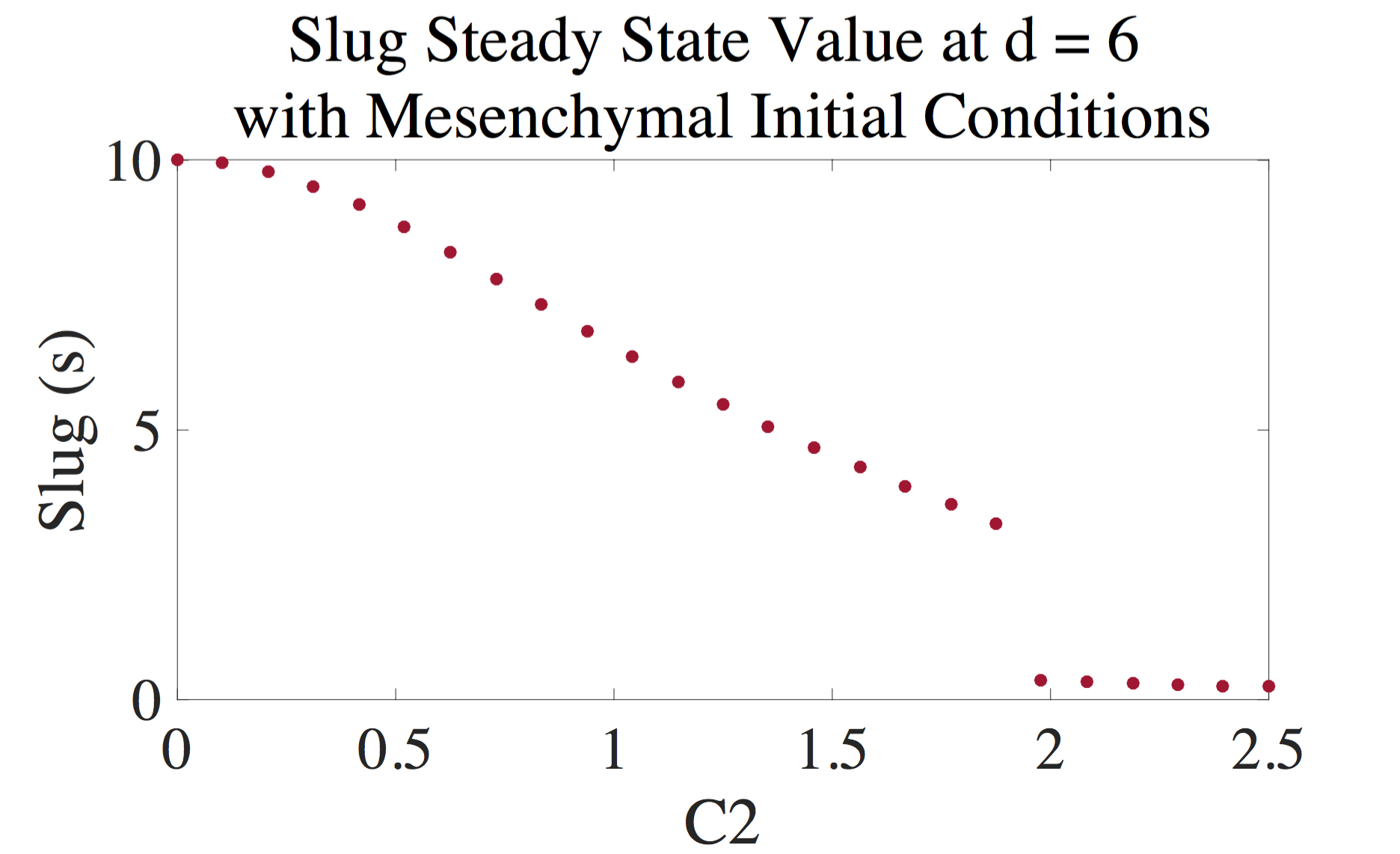 |
| Figure S1CK | Figure S1CL |
|  |  |
| 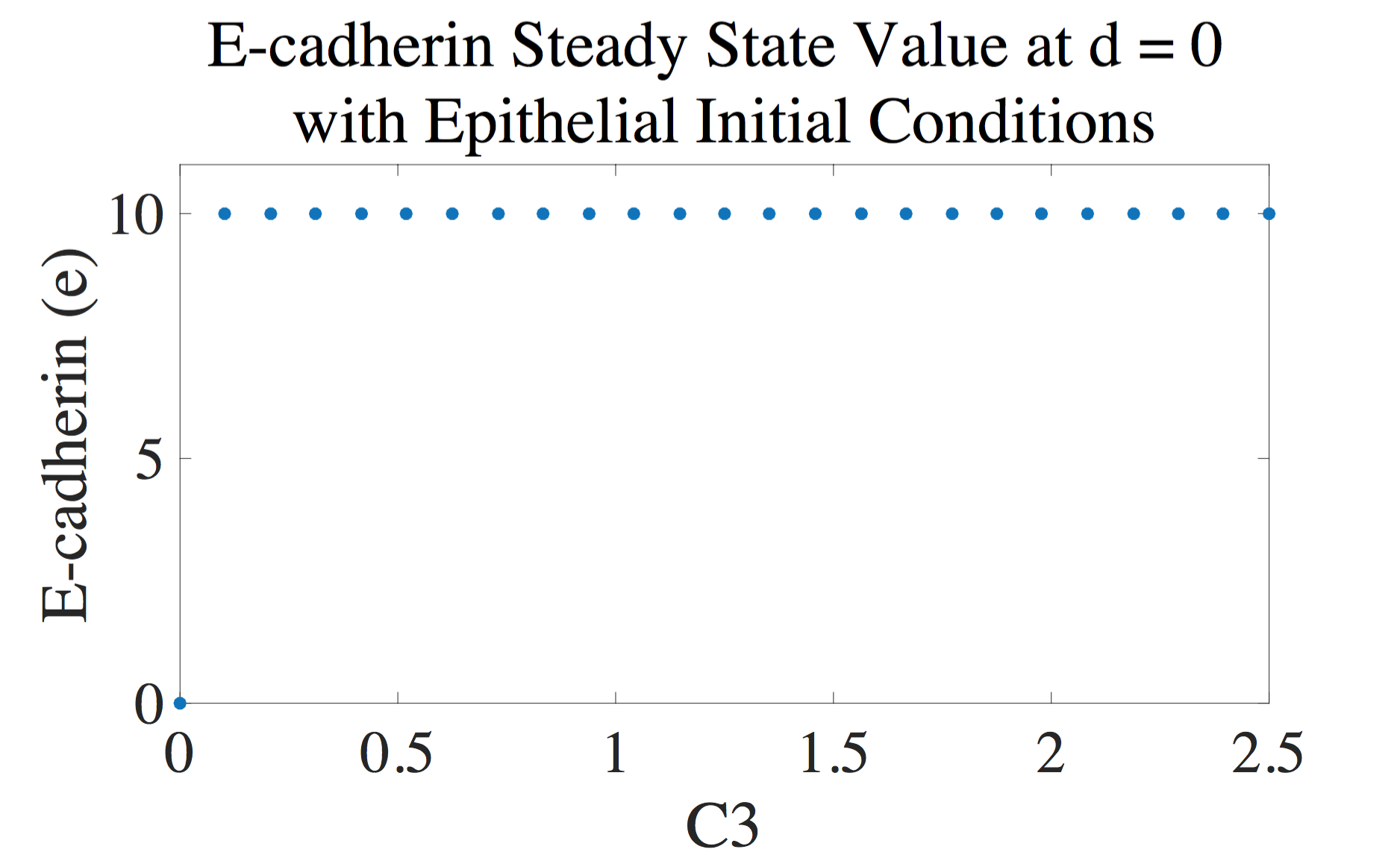 | 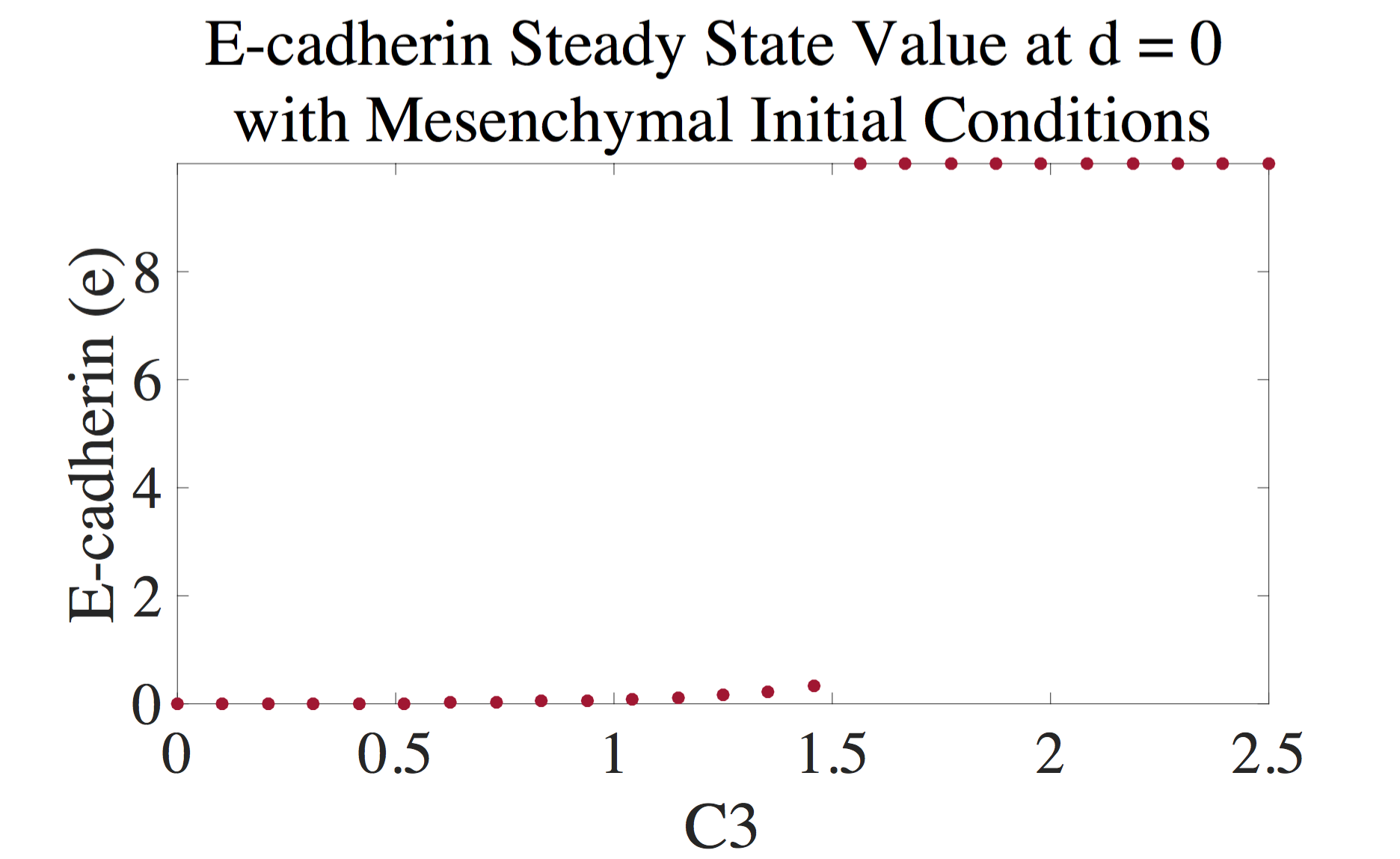 |
| Figure S1CM | Figure S1CN |
|  |  |
| 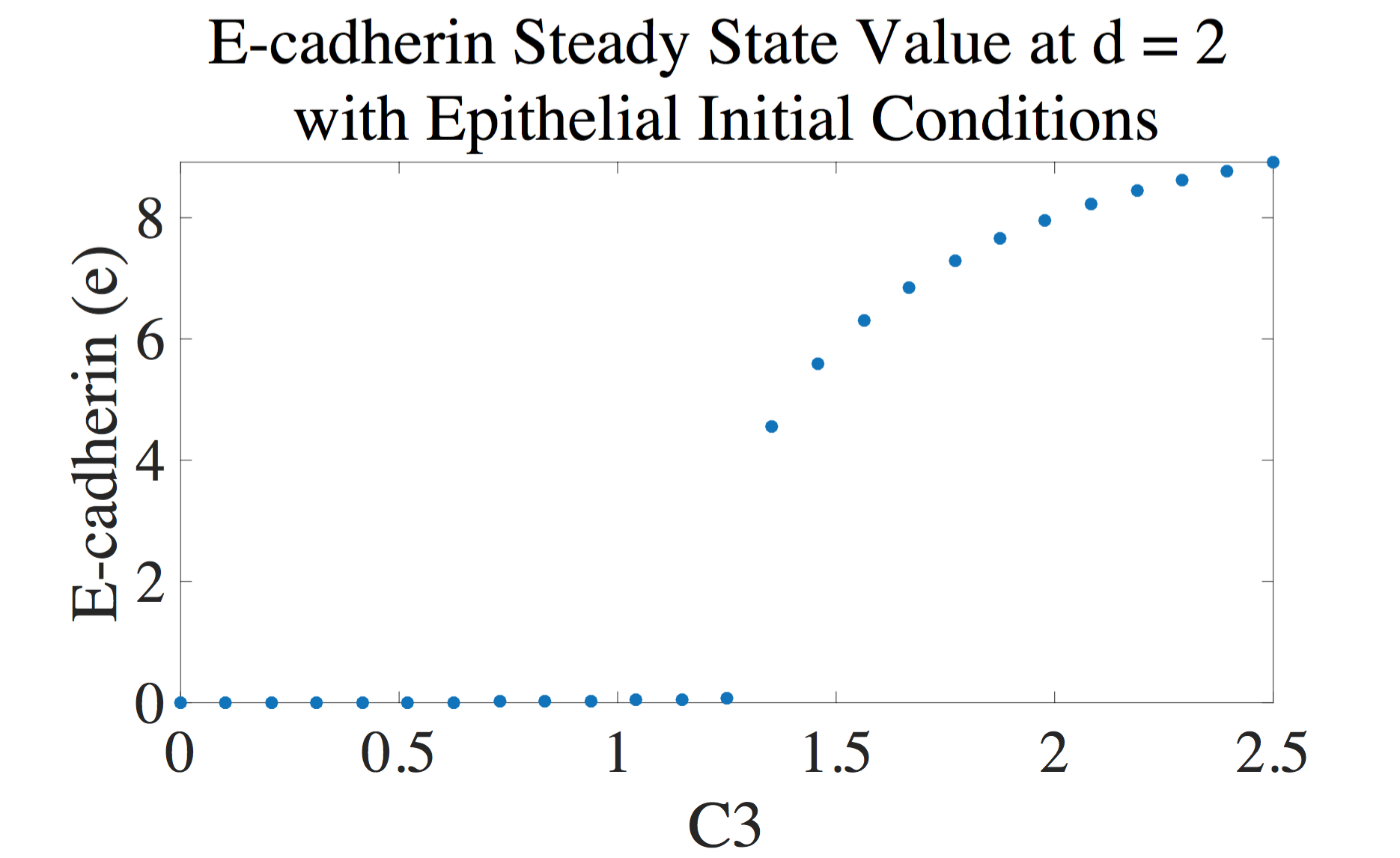 | 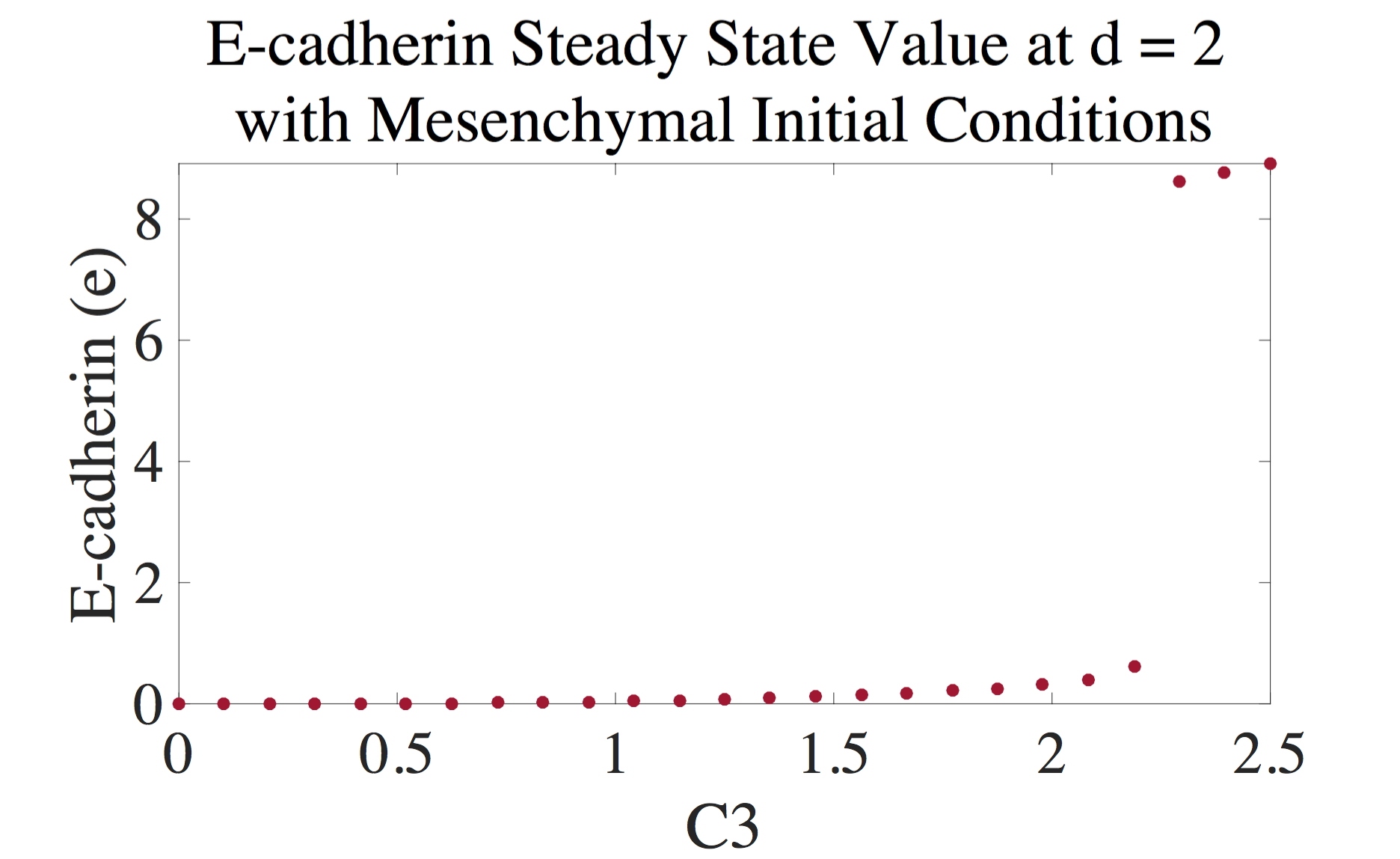 |
| Figure S1CO | Figure S1CP |
|  |  |
| 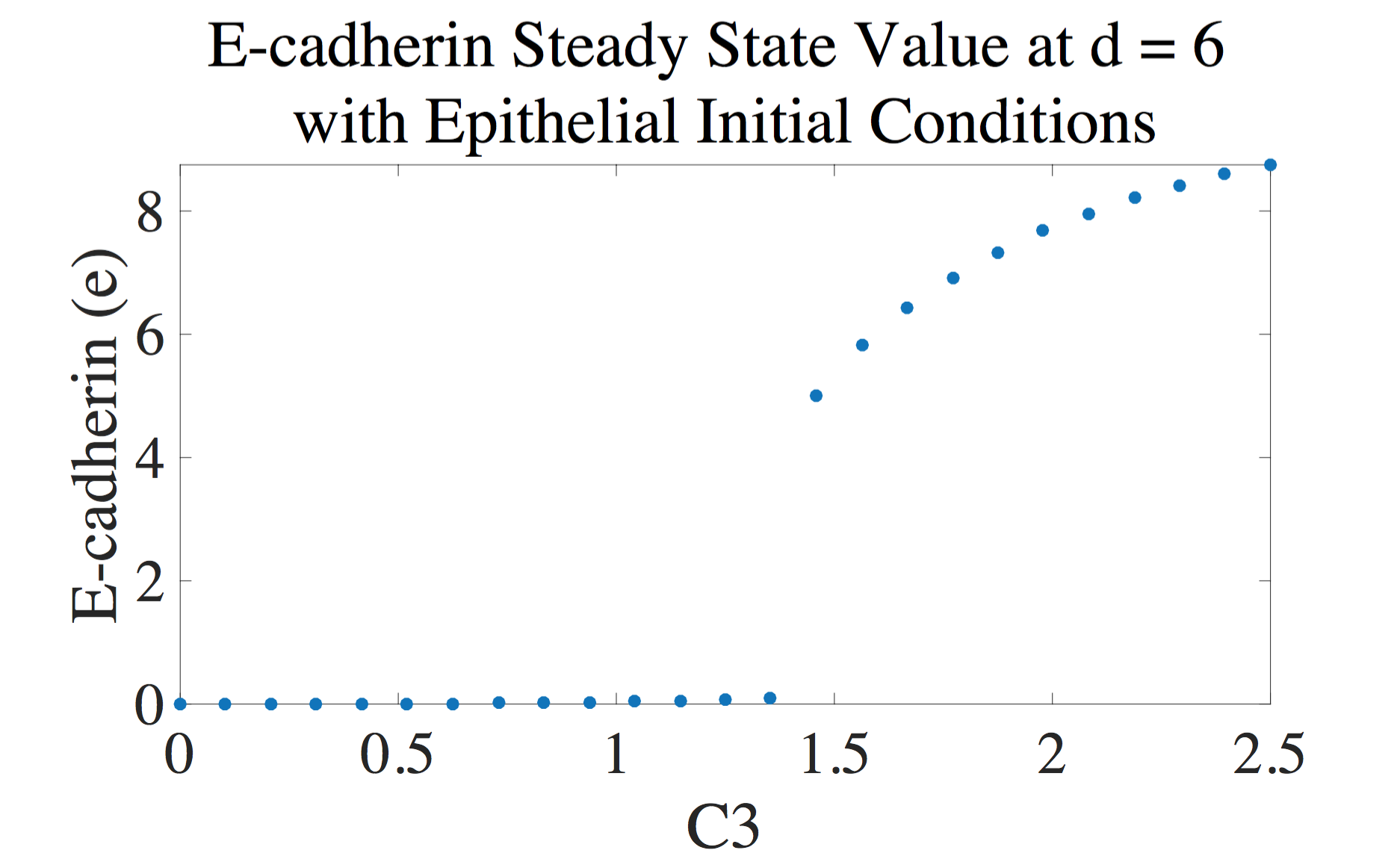 | 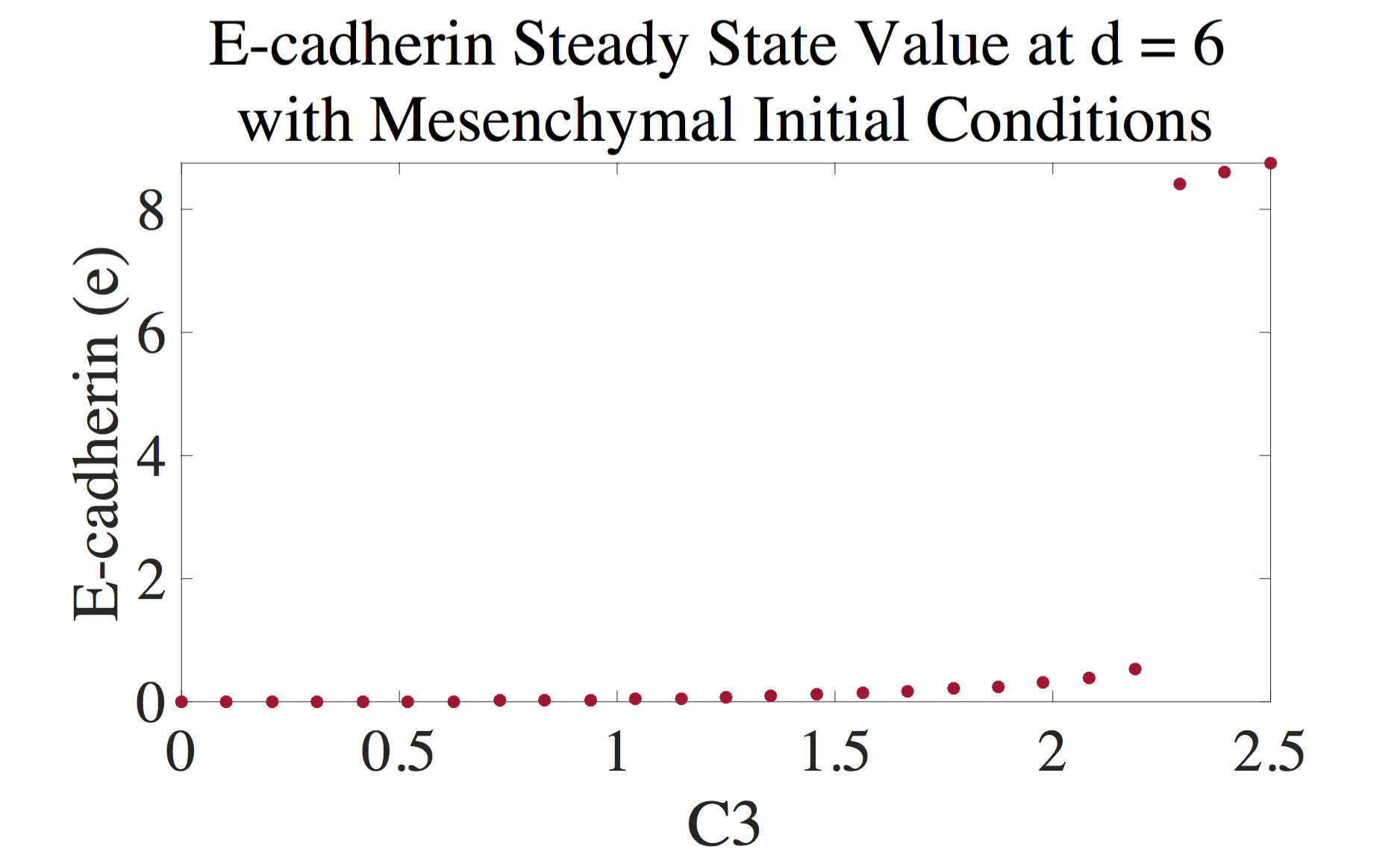 |
| Figure S1CQ | Figure S1CR |
|  |  |
| 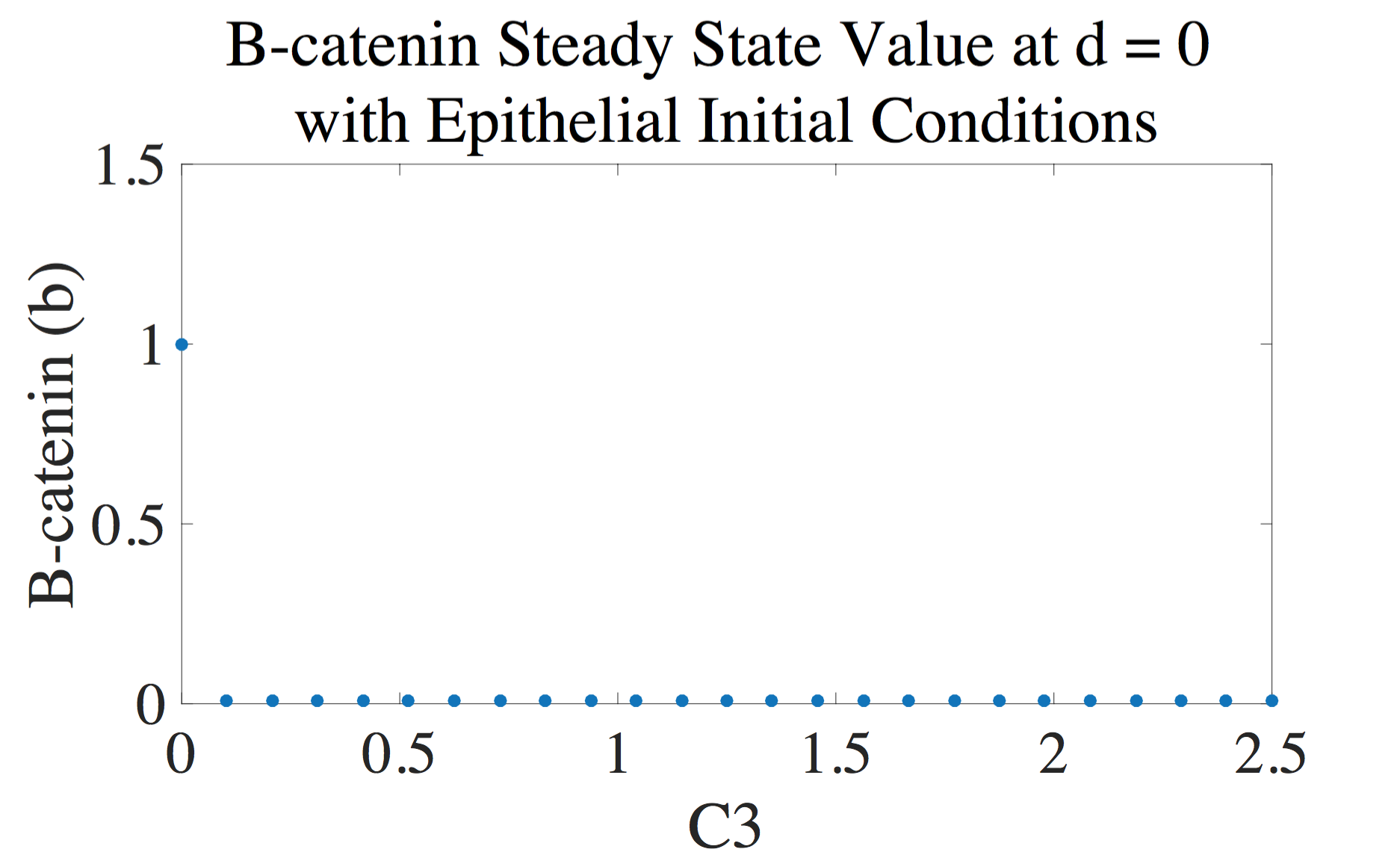 | 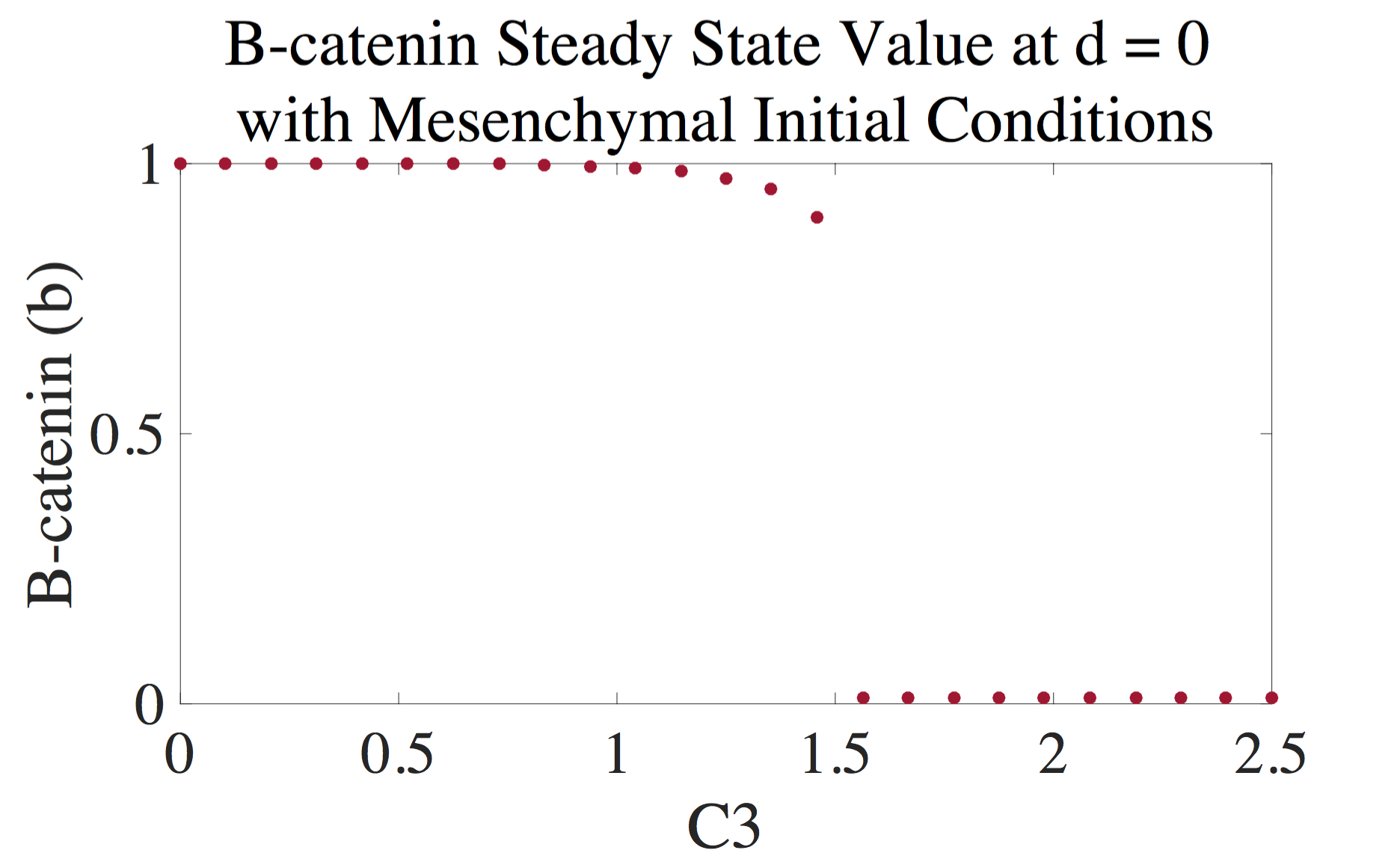 |
| Figure S1CS | Figure S1CT |
|  |  |
| 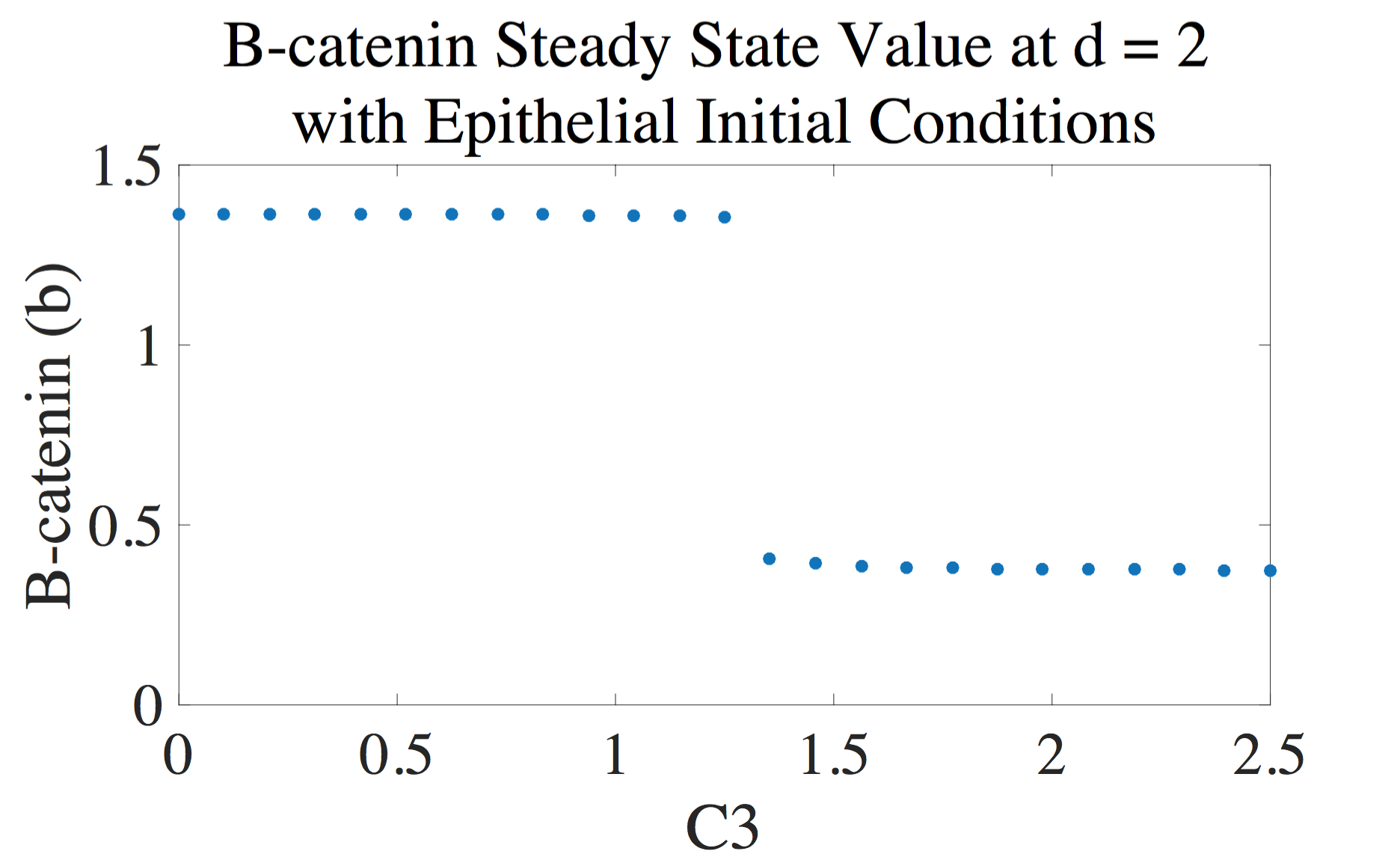 | 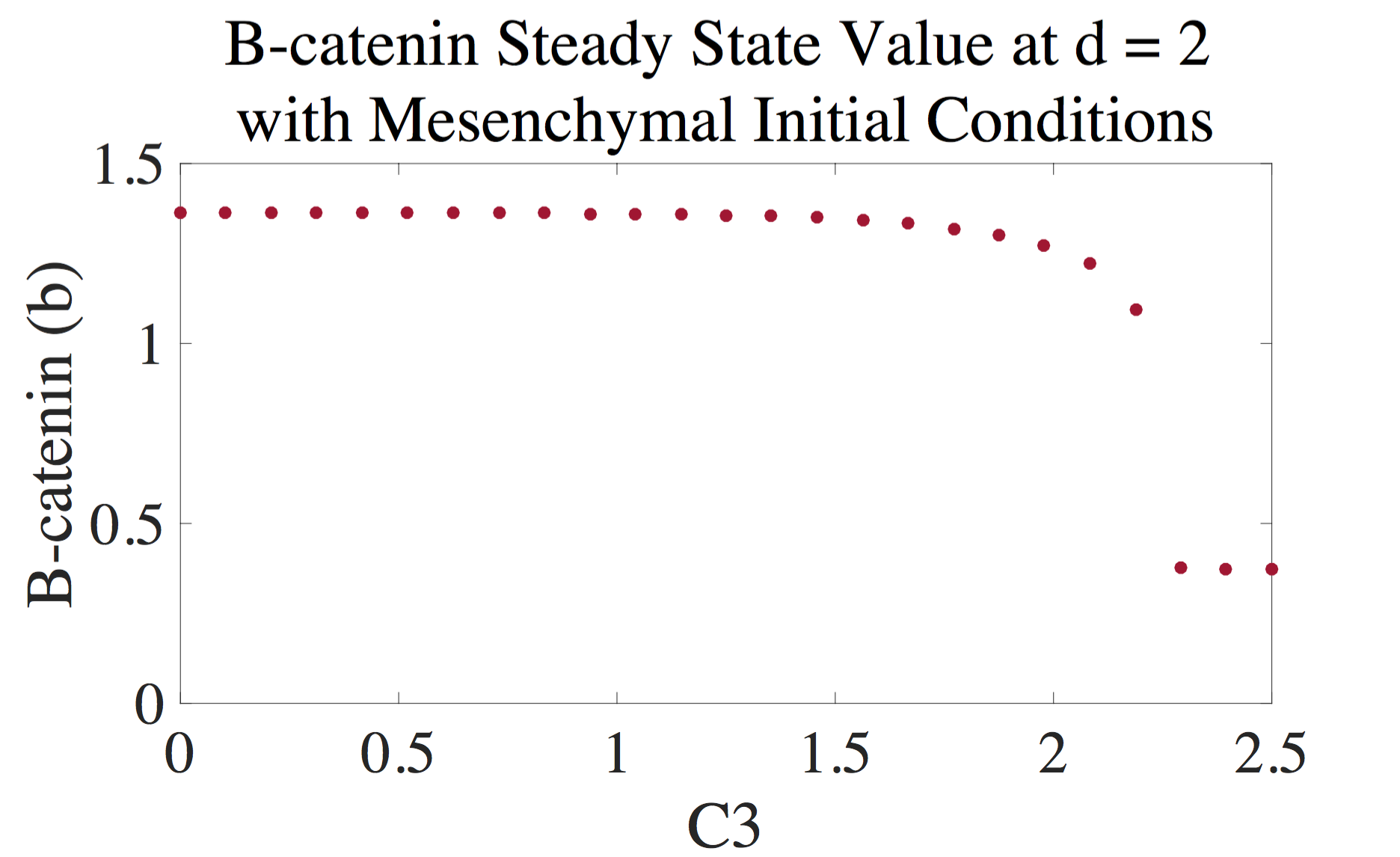 |
| Figure S1CU | Figure S1CV |
|  |  |
|  |  |
| Figure S1CW | Figure S1CX |
|  |  |
|  |  |
| Figure S1CY | Figure S1CZ |
|  |  |
|  |  |
| Figure S1DA | Figure S1DB |
|  |  |
|  |  |
| Figure S1DC | Figure S1DD |
|  |  |
|  |  |
| Figure S1DE | Figure S1DF |
|  |  |
|  |  |
| Figure S1DG | Figure S1DH |
|  |  |
|  |  |
| Figure S1DI | Figure S1DJ |
|  |  |
|  |  |
| Figure S1DK | Figure S1DL |
|  |  |
|  |  |
| Figure S1DM | Figure S1DN |
|  |  |
|  |  |
| Figure S1DO | Figure S1DP |
|  |  |
|  |  |
| Figure S1DQ | Figure S1DR |
|  |  |
|  |  |
| Figure S1DS | Figure S1DT |
|  |  |
|  |  |
| Figure S1DU | Figure S1DV |
|  |  |
|  |  |
| Figure S1DW | Figure S1DX |
|  |  |
|  |  |
| Figure S1DY | Figure S1DZ |
|  |  |
|  |  |
| Figure S1EA | Figure S1EB |
|  |  |
|  |  |
| Figure S1EC | Figure S1ED |
|  |  |
|  |  |
| Figure S1EE | Figure S1EF |
|  |  |
|  |  |
| Figure S1EG | Figure S1EH |
|  |  |
|  |  |
| Figure S1EI | Figure S1EJ |
|  |  |
|  |  |
| Figure S1EK | Figure S1EL |
|  |  |
|  |  |
| Figure S1EM | Figure S1EN |
|  |  |
|  | |
